# Global, regional and national temporal trends in incidence and mortality of non-Hodgkin lymphoma from 1992 to 2021: an age-period-cohort analysis

**DOI:** 10.1007/s00277-025-06261-w

**Published:** 2025-02-24

**Authors:** Shanshan Tang, Jin Cao, Denan Jiang, Longzhu Zhu, Zeyu Luo, Shiyi Shan, Jiali Zhou, Jiayao Ying, Jing Wu, Peige Song, Wei Li

**Affiliations:** 1 The Fourth Affiliated Hospital of School of Medicine, and International School of Medicine, International Institutes of Medicine, Zhejiang University, Yiwu, China; 2 Department of Big Data in Health Science, School of Public Health, The Second Affiliated Hospital, Zhejiang University School of Medicine, Hangzhou, Zhejiang China; 3 Center for Clinical Big Data and Statistics of the Second Affiliated Hospital Zhejiang University School of Medicine, School of Public Health, Zhejiang University School of Medicine, Hangzhou, China

**Keywords:** Non-Hodgkin lymphoma, Global burden of disease, Incidence, Mortality, Age-period-cohort effects

## Abstract

**Objectives:** This study aimed to provide an overview of temporal trends in incidence and mortality of non-Hodgkin lymphoma (NHL) from 1992 to 2021 at global, regional, and national levels, with a special focus on their associations with age, period and cohort. **Methods:** Data were obtained from the Global Burden of Disease Study 2021. We presented temporal trends in NHL incidence and mortality for the world and 204 countries and territories from 1992 to 2021. An age-period-cohort (APC) model was adopted to estimate net drifts (overall annual percentage change), local drifts (annual percentage change in each age group), longitudinal age curves (expected longitudinal age-specific rate), and period (cohort) relative risks. **Results:** The global age-standardized incidence rate (ASIR) and age-standardized mortality rate (ASMR) for NHL were 7.14 (95% uncertainty interval [UI]: 6.58, 7.66) and 3.19 (95% UI: 2.93, 3.44) per 100,000 population in 2021, respectively. From 1992 to 2021, the global net drift of incidence rate was 0.11% (95% confidential intervals [CI]: 0.07%, 0.15%) per year, ranging from - 0.60% (95% CI: -0.66%, -0.54%) in high socio-demographic index (SDI) region to 1.51% (95% CI: 1.46%, 1.57%) in middle SDI region, with 100 countries and territories presenting increasing trends. Similar patterns can be found in the net drift of mortality rate, with 19 countries and territories showing upward trends. Age effects illustrated that incidence and mortality risks progressively increased with advancing age across different SDI regions. Period effects on incidence presented rising risks in high-middle, middle and low-middle SDI regions, whereas on mortality exhibited persistent downward trends in high and high-middle SDI regions. High-middle and middle SDI regions presented initially unfavourable and then favourable trends in incidence and mortality risks across successive birth cohorts. A strong heterogeneity was found in age, period and cohort effects on incidence and mortality across countries. **Conclusions:** Despite observing an increasing temporal trend in NHL incidence, coupled with a declining trend in mortality, NHL represented a substantial public health challenge worldwide. Temporal trends in NHL were not completely commensurate with socioeconomic development and varied widely across countries. Timely intervention should be conducted, especially for middle-aged and aged individuals.

**Supplementary Information:**

The online version contains supplementary material available at 10.1007/s00277-025-06261-w.

## Introduction

Non-Hodgkin lymphoma (NHL) is one of the dominant malignancies in the hematological system that typically infiltrates both lymphoid and hematopoietic tissues, and can extend to other organs, accounting for 90% of all lymphoma incidences [1, 2]. NHL survivors are at significant risk of late mortality from secondary neoplasms, recurrent or progressive disease and chronic health conditions, and late morbidity of multiple organ systems, which collectively contribute to a poor health-related quality of life [3, 4]. Despite significant improvements in prognosis over the past few decades, particularly in high-income countries where survival rates for children with lymphoma exceed 80%, NHL remains a significant public health concern [5, 6]. Globally, NHL ranked as the 11th most commonly diagnosed cancer and the 11th leading cause of cancer-related death in 2020, representing 2.8% of total cancer new cases and 2.6% of total deaths [7]. Besides, NHL-related healthcare expenditure has notably contributed to the economic burden in recent years [8]. For example, rituximab expenditures for targeted therapy reached €263.4 million in 2017, nearly 8% of the total expenditure on high-cost antineoplastic drugs for inpatients care in France [9]. The incidence and mortality of NHL have persistently remained high and even displayed an upward trend in some regions, such as Europe and North America [10]. To inform effective prevention, screening, and management strategies, a comprehensive analysis of NHL incidence and mortality for all countries is warranted. Incidence rates indicate the frequency of new cases and can highlight changes in risk factors, diagnostic practices, and demographic shifts. Mortality rates reveal the lethality of the disease and the effectiveness of treatment protocols over time. Together, these metrics provide a comprehensive view of NHL’s impact on populations and can help identify critical areas for intervention. Notably, the temporal trends of NHL incidence and mortality reflect multidimensional changes and help us understand the evolving dynamics of the disease, which necessitates an in-depth analysis of age, period, and cohort effects. The Age-Period-Cohort (APC) model is a robust statistical framework that allows for this type of analysis. Age effects capture the physiological changes and increased risks associated with aging [11]. Period effects account for external factors affecting all age groups, such as advancements in medical technology, changes in diagnostic criteria, and public health policies [12]. Cohort effects reflect the unique experiences of specific birth cohorts, including early-life exposures, lifestyle changes, and chronic stress [13, 14]. By disentangling these effects, the APC model provides a nuanced understanding of how these factors independently and interactively influence NHL trends. This approach can identify age-specific vulnerabilities, the impact of historical events and technological advancements, and the long-term consequences of early-life exposures.

The Global Burden of Disease (GBD) study estimates a variety of metrics of diseases recorded annually, dating back to 1990, providing a unique opportunity to assess the long-term trends of the disease burden. Based on the GBD data, the vast majority of previous studies have only been conducted at a global or region-specific level for NHL. However, a comprehensive analysis of the NHL burden for all countries is scarce [15–17], let alone the effects of age, period, and cohort on the temporal trends. To address these gaps, our study utilized data from GBD 2021 and described incidence and mortality data by different locations and years for the period 1992–2021. APC model was then used to further explore the age, period, and cohort effects on incidence and mortality of NHL at global, regional and national levels over the previous three decades.

## Methods

### Data sources

GBD 2021 covered 204 countries and territories with comprehensive assessment information on health losses from 371 diseases and injuries [18]. It also produced socio-demographic index (SDI) for each country or territory serving as a comprehensive indicator of income, education and fertility conditions [19]. The SDI values ranged from 0 to 1, with higher values suggesting higher socioeconomic development levels. Based on the SDI values of GBD 2021, the 204 countries and territories were categorized into five quintiles including high, high-middle, middle, low-middle and low SDI. All estimates were reported in 95% uncertainty intervals (UIs), which were obtained by repeatedly sampling the sample 1000 times, whose upper and lower bounds were derived based on the 2.5th and 97.5th percentiles of the uncertainty distribution [20].

In this study, the data on NHL incidence and mortality were derived from the Global Health Data Exchange query tool (https://vizhub.healthdata.org/gbd-results/). NHL was defined according to an approved standard of the Ninth Revision of the International Classification of Diseases (ICD-9) and ICD-10 codes pertaining to NHL (200-200.9, 202-202.98 and C82-C85.29, C85.7-C86.6, C96-C96.9, respectively). We obtained the crude incidence rate, crude mortality rate, age-standardized incidence rate (ASIR), and age-standardized mortality rate (ASMR) of NHL by location (global, SDI regions and 204 countries and territories), age (5–89 years old), year (from 1992 to 2021), and SDI from GBD 2021.

### Statistical analysis

#### Descriptive analyses of NHL incidence and mortality at global, regional and national levels

Descriptive analyses were conducted on NHL incidence and mortality at global, regional and national levels in 1992 and 2021. Age-standardized rate (ASR) was an important indicator comparing incidence and mortality that effectively excluded the influence of age factors between regions or countries with different age structures, thus ASIR and ASMR were employed to quantify the burden of NHL in this study. The age distribution of the global population from the GBD 2021 study was utilized to standardize incidence and mortality rates per 100,000 person years [21]. Each rate was reported per 100,000 population and the corresponding 95% uncertainty interval (UI) was provided simultaneously.

#### APC analysis of NHL incidence and mortality at global, regional and national levels

The APC model was a statistical method used to extract and reveal possible information about illness patterns and to assess the contributions of age, period, and cohort effects on the outcomes. In this study, we mainly focused on the following estimable functions. Net drift reflected the overall log-linear trend by period and birth cohort, indicating the overall annual percentage change of the expected age-adjusted rate. Local drifts represented the log-linear trends for each age group by period and birth cohort, indicating the annual percentage change of the expected age-specific rate over time. Longitudinal age curve indicated the fitted longitudinal age-specific rates in the reference cohort adjusted for period deviations. Period (or cohort) rate ratios (RR) represented the ratio of age-specific rates in each period (or cohort) relative to the reference one. The RR value more than 1.0 suggested that the factor increased the risk of NHL incidence or mortality, otherwise it indicated that the factor decreased the risk of NHL incidence or mortality. To address the identification problem caused by linear relationships between age, period, and cohort, the intrinsic estimator (IE) method associated with the APC model was used, overcoming the drawback of model parameters being unpredictable.

The incidence and mortality estimates for NHL and population data of each region/country from the GBD 2021 were used as data inputs for the APC model with IE method. Notably, 11 countries and territories (including American Samoa, Cook Islands, Kiribati, Marshall Islands, Micronesia (Federated States of), Nauru, Niue, Northern Mariana Islands, Palau, Tokelau and Tuvalu) were excluded from the analyses of APC model due to the rarity of NHL incidence number. Meanwhile, 17 countries and territories (including American Samoa, Antigua and Barbuda, Cook Islands, Greenland, Kiribati, Maldives, Marshall Islands, Micronesia (Federated States of Micronesia), Nauru, Niue, Northern Mariana Islands, Palau, Saint Kitts and Nevis, Sao Tome and Principe, Tokelau, Tuvalu and Vanuatu) were excluded due to the rarity of NHL mortality number. For APC analysis, age intervals must be equal to period intervals, which means 5-year age groups should correspond to 5-year calendar periods. Thus, we divided the population aged 5–89 years into 17 age groups (5–9, 10–14, 15–19, 20–24, 25–29, 30–34, 35–39, 40–44, 45–49, 50–54, 55–59, 60–64, 65–69, 70–74, 75–79, 80–84, 85–89 years) with 5-year age intervals. Those younger than five years old and older than 90 years old were excluded from this study due to the absence or rarity of NHL events. Correspondingly, the whole study period (1992–2021) was divided into six consecutive time periods (1992–1996, 1997–2001, 2002–2006, 2007–2011, 2012–2016, 2017–2021). The sample consisted of 22 partially overlapping 10-year birth cohorts, which included those born between 1902–1911 and 2007–2016. For relative rate measurements, the reference period interval was from 2002 to 2006, and the reference birth cohort interval was from 1947 to 1956. Wald chi-square tests were used to calculate the significance of the function and estimable parameters. All statistical tests were two-sided, and *p* < 0.05 was considered statistically significant differences. The APC model was perfomed using the APC Web Tool (http://analysistools.nci.nih.gov/apc/) from the National Cancer Institute [22]. All graphics were generated via R software (version 4.0.3).

## Results

### Trends in NHL incidence and mortality, 1992–2021

Table 1 showed the global and regional ASIR, ASMR, as well as net drifts of NHL incidence and mortality rate. The global ASIR and ASMR for NHL were 7.14 (95% UI: 6.58, 7.66) and 3.19 (95% UI: 2.93, 3.44) per 100,000 population in 2021, respectively. At the SDI regions level, the ASIR for NHL decreased from 12.96 (95% UI: 12.03, 13.68) per 100,000 population in high SDI region to 3.54 (95% UI: 3.15, 4.41) per 100,000 population in low-middle SDI region in 2021. The ASMR was persistently highest in the high SDI region (3.97, 95% UI: 3.60, 4.19 per 100,000 population) and lowest in the middle SDI region (2.54, 95% UI: 2.28, 2.82 per 100,000 population) in 2021. The APC model estimated a global net drift of incidence rate at 0.11% (95% CI: 0.07%, 0.15%) per year, ranging from -0.60% (95% CI: -0.66%, -0.54%) in high SDI region to 1.51% (95% CI: 1.46%, 1.57%) in middle SDI region. Similar patterns can be found in the net drift of mortality rate (from -2.19%, 95% CI: -2.27%, -2.11% in high SDI region to -0.39%, 95% CI: -0.44%, -0.34% in middle SDI region).

**Fig. 1:**
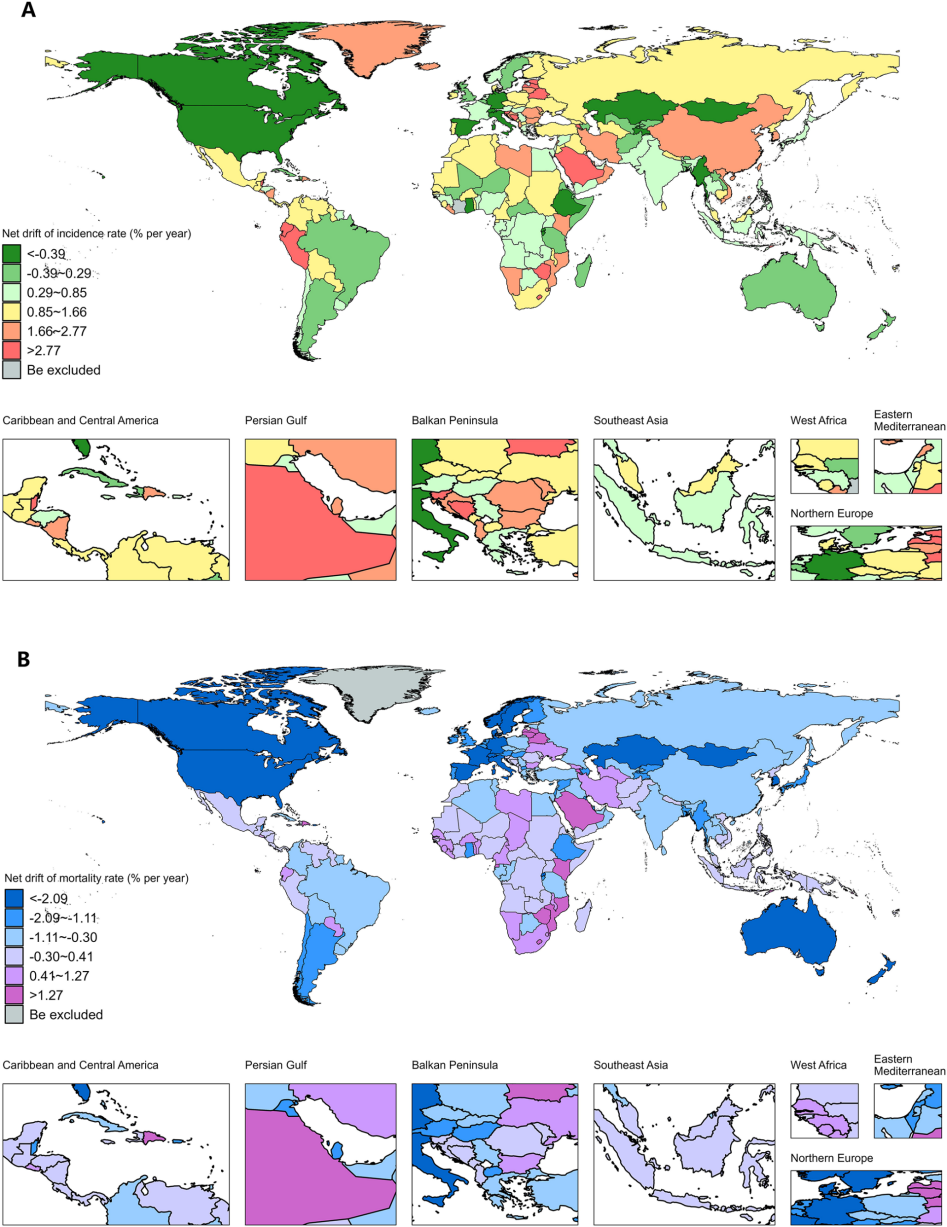
The net drifts of incidence and mortality rate from 1992 to 2021 for NHL in 204 countries and territories. (**A**)World map of the net drift of incidence rate from 1992 to 2021 for NHL.(**B**) World map of the net drift of mortality rate from 1992 to 2021 for NHL. NHL, non-Hodgkin lymphoma

National ASIR, ASMR in 1992 and 2021, as well as net drifts of incidence and mortality rate from 1992 to 2021 for NHL were shown in Fig. 1, supplemental Figure S1-S4, and supplemental Table S1-S2. In 2021, in terms of NHL ASIR, the top five countries and territories were Peru (24.00, 95% UI: 17.61, 31.10 per 100,000 people), Slovenia (23.82, 95% UI: 19.43, 28.99 per 100,000 people), Iceland (23.81, 95% UI: 20.33, 28.43 per 100,000 people), Andorra (22.85, 95% UI: 15.28, 30.64 per 100,000 people) and Estonia (21.70, 95% UI: 18.16, 25.77 per 100,000 people). Meanwhile, for the ASMR of NHL, Malawi (13.00, 95% UI: 9.05, 18.24 per 100,000 people), Grenada (10.00, 95% UI: 8.70, 11.41 per 100,000 people), Zimbabwe (9.46, 95% UI: 6.35, 12.04 per 100,000 people), Uganda (9.05, 95% UI: 6.80, 12.03 per 100,000 people) and Zambia (6.93, 95% UI: 4.59, 11.43 per 100,000 people) were the top five countries and territories. Notably, Kiribati recorded the lowest ASIR and ASMR, with 0.82 (95% UI: 0.47, 1.11) per 100,000 people and 0.66 (95% UI: 0.38, 0.88) per 100,000 people, respectively. The net drift of incidence rate suggested 100 countries and territories with increasing trends (net drifts and its 95% CI were > 0) and 14 countries and territories with decreasing trends (net drifts and its 95% CI were < 0) estimated from APC model. In comparison, the net drift of mortality rate presented 19 countries and territories with upward trend and 55 countries and territories with downward trend.

**Fig. 2:**
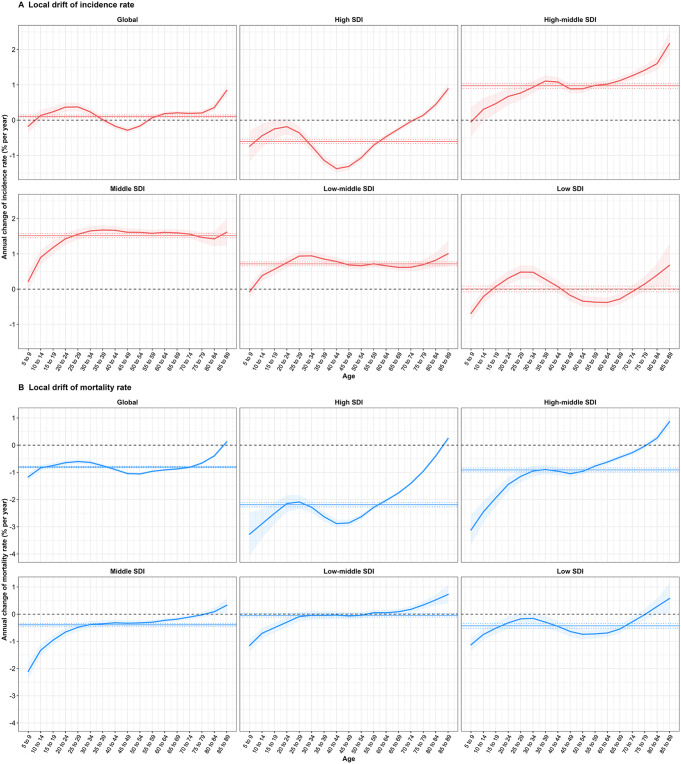
The local drifts of incidence and mortality rate from 1992 to 2021 for NHL across SDI quintiles. (**A**) Local drift of incidence rate from 1992 to 2021 for NHL for seventeen age groups. (**B**) Local drift of mortality rate from 1992 to 2021 for NHL for seventeen age groups. The dots and shaded areas indicate the annual percentage change of incidence and mortality rate (% per year) and the corresponding 95% CIs. NHL, non-Hodgkin lymphoma; SDI, socio-demographic index; CI, confidential interval

The local drifts of incidence and mortality rate calculated from APC model was presented in Fig. 2. Globally, NHL incidence rate demonstrated decreasing trends from 40–44 to 50–54 years group, and increasing trends from 15–19 to 30–34 years group and 55–59 to 85–89 years group. The increasing trends intensified from 15–19 to 25–29 years group and 70–74 to 85–89 years group, being highest in 85–89 years group (0.84%, 95% CI: 0.70%, 0.98%). However, NHL incidence rate remained nearly constant in 5–9, 10–14 and 35–39 years group from 1992 to 2021.Values of local drift were mainly above 0 for most age groups in high-middle, middle and low-middle SDI regions, indicating a general increase in NHL incidence rate. Globally, the NHL mortality rate showed a decreasing trend from 5–9 to 80–84 years group, followed by an increase in 85–89 years group, a pattern that was also observed in high SDI region. In the age group from 5–9 to 70–74 years, NHL mortality rate decreased in middle and low SDI regions, 75–79 to 80–84 years kept flat, and 85–89 years increased. Notably, in low-middle SDI region, the mortality rate presented a flat trend in the age group from 25–29 to 65–69 years. The local drifts of incidence and mortality rate for each country was demonstrated in supplemental Table S3.

**Table 1:** Trends of incidence and mortality of non-Hodgkin lymphoma from 1992 to 2021 by SDI quintiles

	Global		High SDI		High-middle SDI		Middle SDI		Low-middle SDI		Low SDI	
	1992	2021	1992	2021	1992	2021	1992	2021	1992	2021	1992	2021
**ASIR**												
Rate per 100,000	6.37 (6.04,6.77)	7.14 (6.58,7.66)	12.66 (12.19,12.97)	12.96 (12.03,13.68)	5.49 (5.23,5.88)	7.39 (6.68,8.09)	3.43 (3.17,3.97)	5.12 (4.53,5.73)	2.87 (2.51,3.47)	3.54 (3.15,4.41)	3.98 (3.21,4.71)	4.00 (3.41,4.74)
**ASMR**												
Rate per 100,000	3.69 (3.44,4.02)	3.19 (2.93,3.44)	5.24 (4.98,5.40)	3.97 (3.60,4.19)	3.06 (2.86,3.33)	2.69 (2.42,2.95)	2.70 (2.49,3.15)	2.54 (2.28,2.82)	2.57 (2.25,3.14)	2.70 (2.41,3.36)	3.79 (3.06,4.48)	3.50 (3.01,4.14)
**APC model estimates**												
Net drift of incidence rate (% per year)	0.11 (0.07,0.15)		-0.60 (-0.66,-0.54)		0.97 (0.9,1.04)		1.51 (1.46,1.57)		0.72 (0.66,0.77)		0.00 (-0.08,0.09)	
Net drift of mortality rate (% per year)	-0.81 (-0.83,-0.78)		-2.19 (-2.27,-2.11)		-0.91 (-0.98,-0.84)		-0.39 (-0.44,-0.34)		-0.04 (-0.11,0.02)		-0.43 (-0.52,-0.34)	

Parentheses for GBD estimates denote 95% uncertainty intervals (UIs) and parentheses for net drifts denote 95% confidential intervals (CIs)

Abbreviations: ASIR, age-standardized incidence rates; ASMR, age-standardized mortality rates; APC, age-period-cohort; GBD, Global Burden of Diseases; SDI, socio-demographic index

### Age, period and cohort effects on NHL incidence and mortality

#### Age-period-cohort effects at global and SDI region levels

**Fig. 3:**
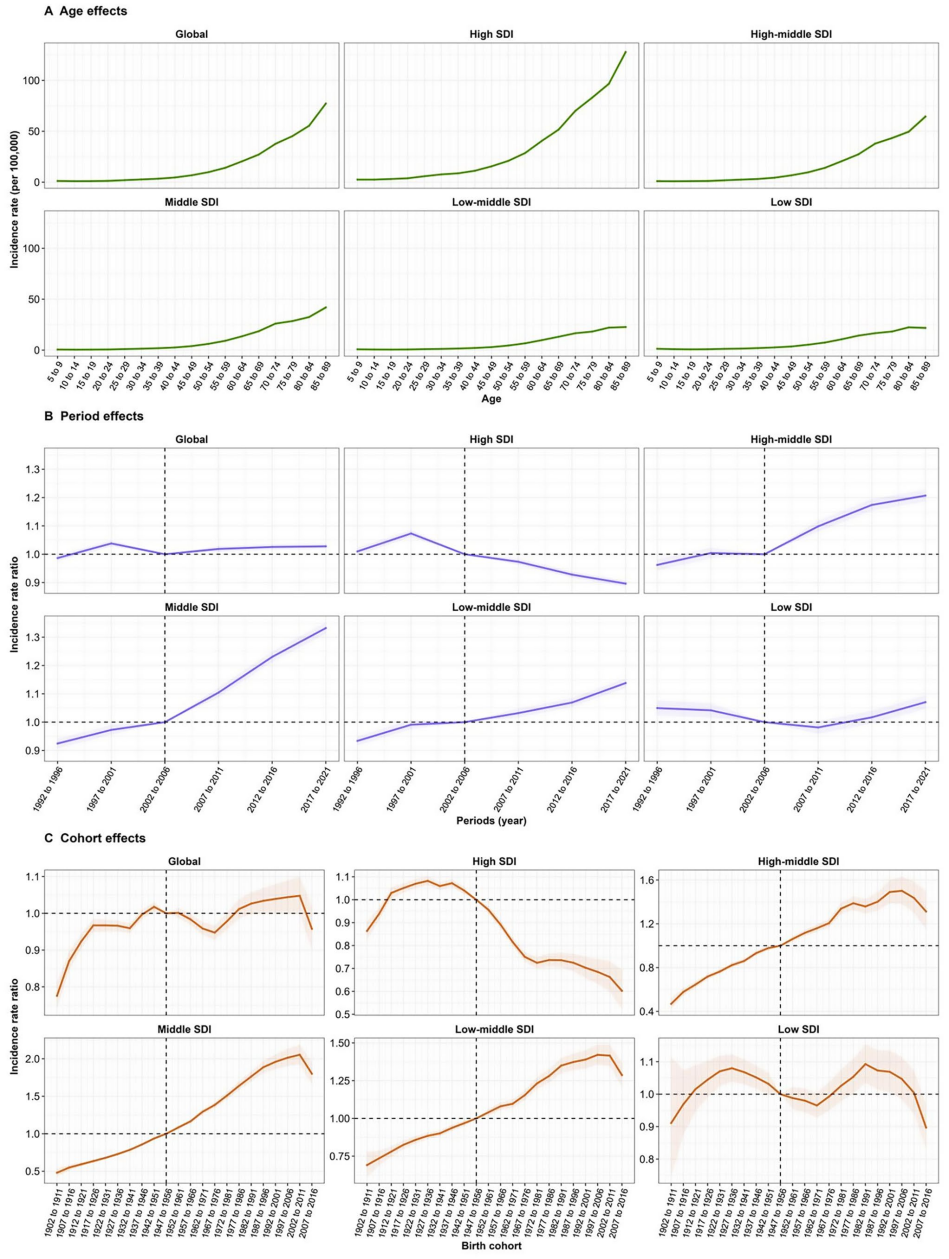
Age, period and cohort effects on NHL incidence across SDI quintiles. (**A**) Age effects are illustrated by the fitted longitudinal age-specific rates for a given number of birth cohorts adjusted for period deviations.(**B**) Period effects are illustrated by the period relative risk of incidence (incidence rate ratio) and calculated as the ratio of age-specific rates from 1992–1996 period to 2017–2021 period, with the reference period set at 2002–2006. (**C**) Cohort effects are illustrated by the cohort relative risk of incidence (incidence rate ratio) and calculated as the ratio of age-specific rates from 1902–1911 cohort to 2007–2016 cohort, with the reference cohort set at 1947–1956. The dots and shaded areas denote the incidence rates or rate ratios and their corresponding 95% CIs. NHL, non-Hodgkin lymphoma; SDI, socio-demographic index; CI, confidential interval

**Fig. 4:**
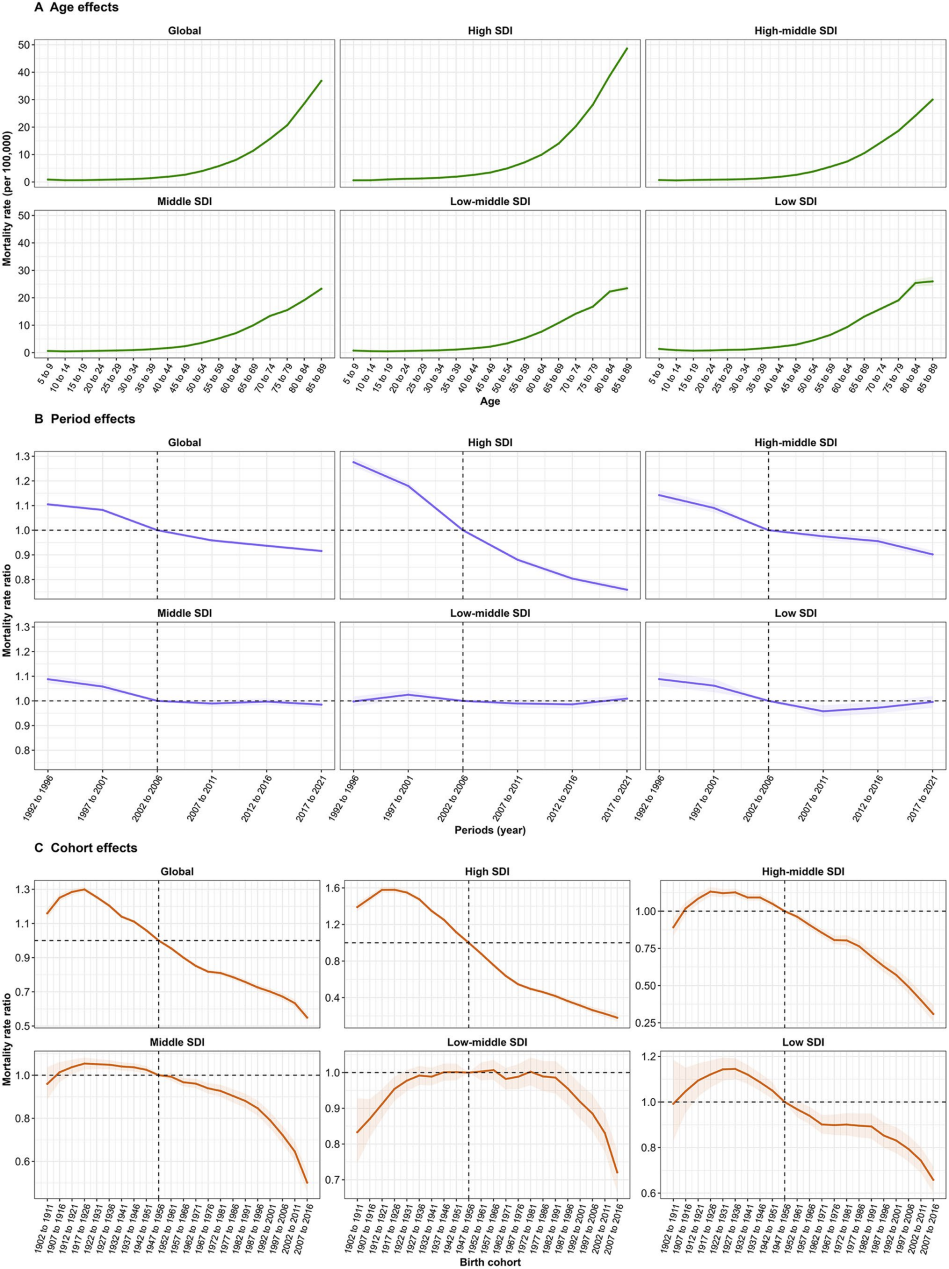
Age, period and cohort effects on NHL mortality across SDI quintiles. (**A**) Age effects are illustrated by the fitted longitudinal age-specific rates for a given number of birth cohorts adjusted for period deviations.(**B**) Period effects are illustrated by the period relative risk of mortality (mortality rate ratio) and calculated as the ratio of age- specific rates from 1992–1996 period to 2017–2021 period, with the reference period set at 2002–2006. (**C**) Cohort effects are illustrated by the cohort relative risk of mortality (mortality rate ratio) and calculated as the ratio of age-specific rates from 1902–1911 cohort to 2007–2016 cohort, with the reference cohort set at 1947–1956. The dots and shaded areas denote the incidence rates or rate ratios and their corresponding 95% CIs. NHL, non-Hodgkin lymphoma; SDI, socio-demographic index; CI, confidential interval

The age, period and birth cohort effects by SDI quintile derived from APC model were demonstrated in Figs. 3 and 4. Generally, there were similar patterns in age effects on NHL incidence rate across different SDI regions. The lowest risk was noted in the 5–44 year age group, with the risk increasing progressively with age. Moreover, similar patterns of age effects were observed on NHL mortality rate across all SDI quintiles. Age effects in NHL incidence and mortality rate were almost completely commensurate with socioeconomic development, that was, higher SDI regions displayed an overall higher incidence and mortality rate and faster risk increases as compared to other SDI regions.

Globally, period effects revealed an increase in incidence risks before 1997–2001 and after 2002–2006, and a decline from 1997–2001 to 2002–2006 period. Overall, period effects presented rising risks in incidence across different SDI regions except in high and low SDI regions. However, period effects on mortality at global level exhibited a persistent downward trend, aligning with trends observed in high and high-middle SDI regions. Period effects on NHL mortality in middle SDI region showed a slightly decline, in low-middle SDI region kept relatively stable and in low SDI region presented a declining first and then rising risks.

Regarding birth cohort effects, there was an increase in the cohort born before 1927 to 1936, and a decline in 1962 to 1971 and 1967 to 1976 in incidence risks globally. In high-middle, middle and low-middle SDI regions, cohort effects showed initially rising and then declining incidence risks across successive birth cohorts. Especially, in low SDI region, the relative risk of incidence remained relatively stable across all birth cohorts. Globally, cohort effects displayed a pattern in which the mortality risks initially increased and subsequently decreased across successive birth cohorts, with a highest risk of NHL mortality in 1917–1926, a trend that was similar in high, high-middle and middle SDI regions. Low-middle SDI region had an increase of mortality risks in the cohort born before 1917 to 1926, and a decline after 1992 to 2001. In low SDI region, the mortality risks exhibited a pattern of firstly increasing followed by a decline, with the cohort inflection point situated within the birth cohort of 1927 to 1936.

### Age-period-cohort effects in representative countries

**Fig. 5:**
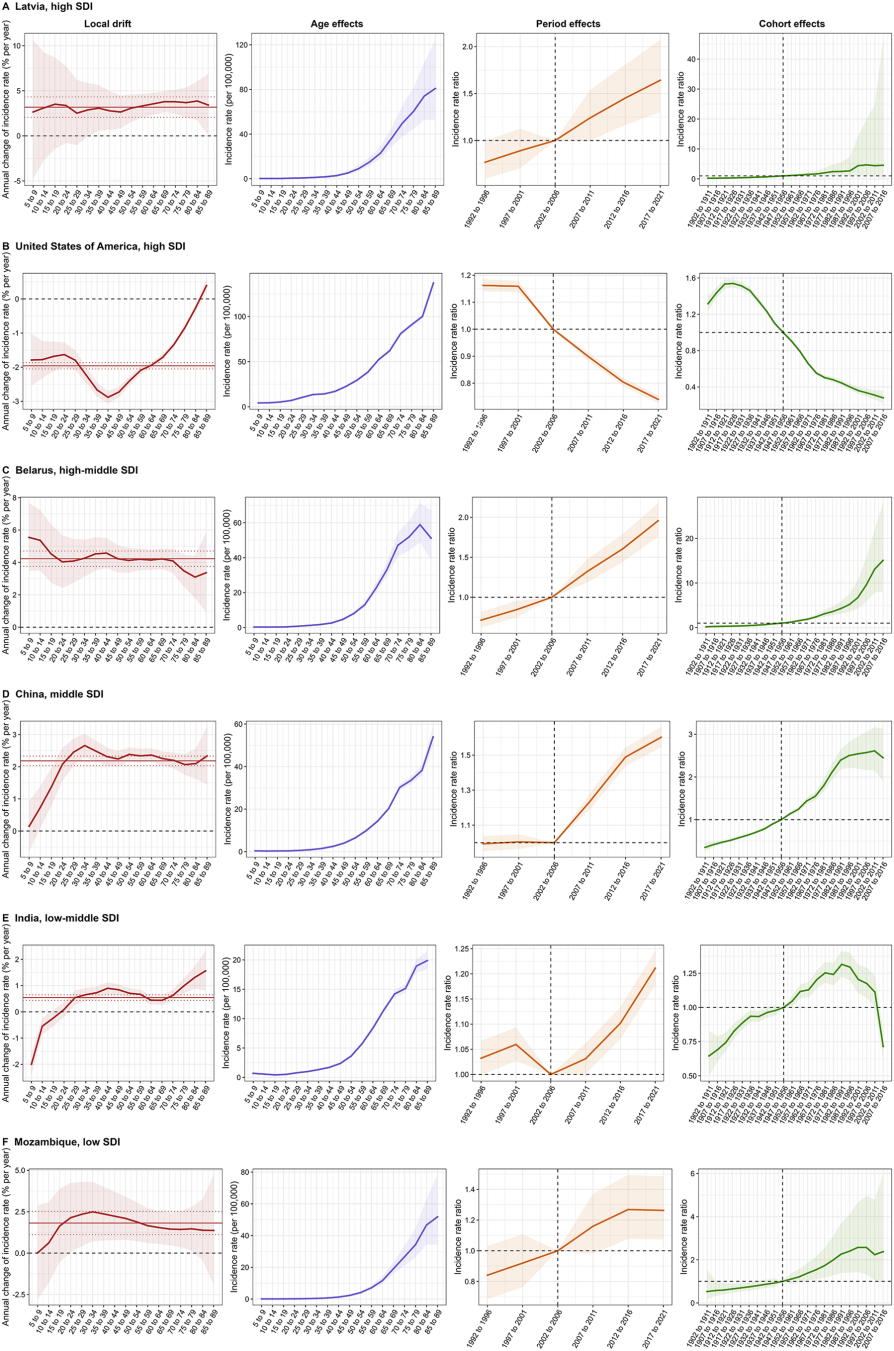
Age, period and cohort effects on NHL incidence in representative countries. Local drift denotes the annual percentage change of age-specific incidence (% per year) from 1992 to 2021. Age effects are illustrated by the fitted longitudinal age-specific rates for a given number of birth cohorts adjusted for period deviations. Period effects are illustrated by the period relative risk of incidence (incidence rate ratio) and calculated as the ratio of age-specific rates from 1992–1996 period to 2017–2021 period, with the reference period set at 2002–2006. Cohort effects are illustrated by the cohort relative risk of incidence (incidence rate ratio) and calculated as the ratio of age-specific rates from 1902–1911 cohort to 2007–2016 cohort, with the reference cohort set at 1947–1956. The dots and shaded areas denote the incidence rates or rate ratios and their corresponding 95% CIs. NHL, non-Hodgkin lymphoma; SDI, socio-demographic index; CI, confidential interval

**Fig. 6:**
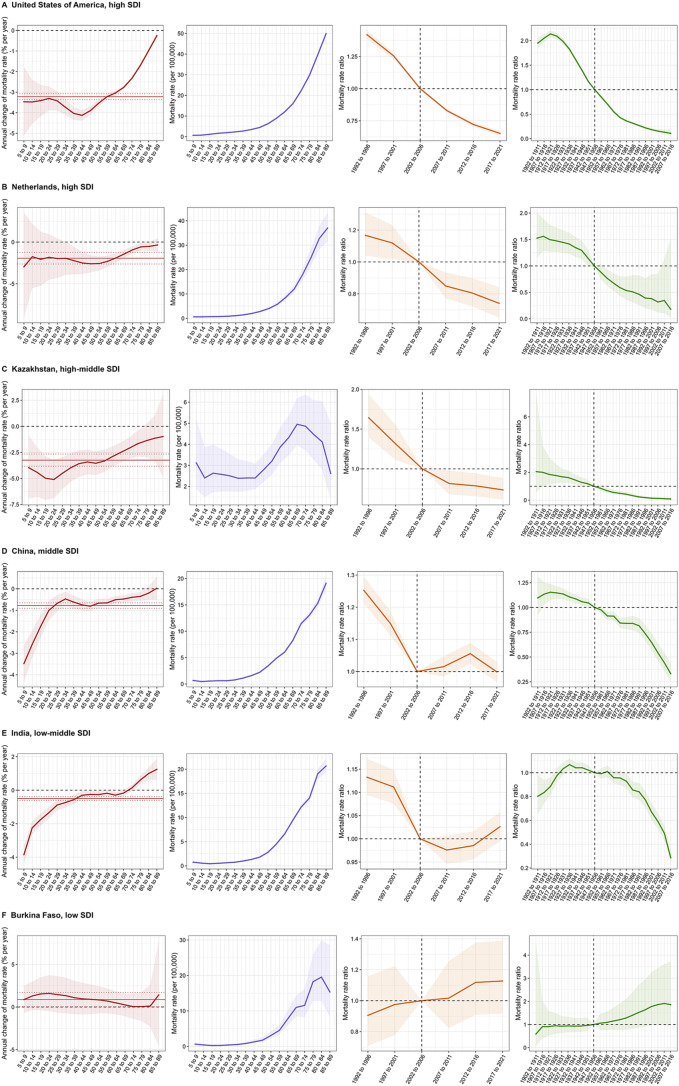
Age, period and cohort effects on NHL mortality in representative countries. Local drift denotes the annual percentage change of age-specific mortality (% per year) from 1992 to 2021. Age effects are illustrated by the fitted longitudinal age-specific rates for a given number of birth cohorts adjusted for period deviations. Period effects are illustrated by the period relative risk of mortality (mortality rate ratio) and calculated as the ratio of age-specific rates from 1992–1996 period to 2017–2021 period, with the reference period set at 2002–2006. Cohort effects are illustrated by the cohort relative risk of mortality (mortality rate ratio) and calculated as the ratio of age-specific rates from 1902–1911 cohort to 2007–2016 cohort, with the reference cohort set at 1947–1956. The dots and shaded areas denote the mortality rates or rate ratios and their corresponding 95% CIs. NHL, non-Hodgkin lymphoma; SDI, socio-demographic index; CI, confidential interval

The age, period, and cohort effects on NHL incidence and mortality in each country were shown in supplemental Table S4-S6. To better characterize the temporal trends in NHL incidence and mortality worldwide, Figs. 5 and 6 presented several representative countries across different SDI quintiles mainly selected by net drifts and local drifts of incidence and mortality rate, with relatively favorable and unfavorable age, period and birth cohort effects.

In terms of age-period-cohort effects on NHL incidence, Latvia represented a typical trend in high SDI countries with unfavorable manifestations, where incidence increases were observed across almost all age groups with both period and cohort risks worsening in recent years. In contrast, the USA had extremely favorable trends in NHL incidence among high SDI countries, with local drift < 0% per year for almost all age groups and noticeable declining risks as periods progressed and in successive birth cohorts after 1917 to 1926. Belarus showed significant incidence increases for all age groups in high-middle SDI countries, with continuous deteriorations in period and cohort risks. China and India were examples of highly populous middle and low-middle SDI countries, with increasing incidence across almost all age groups. Period effects exhibited increasing risks in periods after 2007 to 2011 for both China and India. Cohort effects showed similar patterns in these two countries, with rising risks in the early stage and declining risks in the later stage of the study period. Mozambique represented low SDI countries with local drift > 0% per year in nearly all age groups and showed gradual increasing risks in periods after 2012 to 2016.

With regard to age-period-cohort effects on NHL mortality, the USA stood out for the lowest net drift in high SDI countries, with period effects dropping constantly. In Netherlands, NHL mortality exhibited a flat trend in the age groups from 5–9 to 30–34 years and 80–84 to 85–89 years, showing significantly decreasing risks over the periods. For the cohort effects, the risk of mortality dropped dramatically before 1997–2006. Kazakhstan was a high-middle SDI country with the lowest net drift, and the mortality rate presented a steep decline after 65–69 years. Additionally, a notable gradual attenuation in risk was observed across periods and birth cohorts after 1907 to 1916. China, a highly populous middle SDI country, demonstrated a significant decline in period risk before 2002–2006 and in cohort risk since 1907**–**1916. Among low-middle SDI countries, India exhibited a decreasing trend of mortality in most age groups, with more favorable period effects before 2002–2006. Burkina Faso was notable for its relatively stable risks in both period and birth cohort effects in low SDI countries.

## Discussion

In this study, we comprehensively analyzed the current trends in the NHL incidence and mortality. Additionally, we used APC model to reflect the substantial health disparities setting of NHL incidence and mortality in the three dimensions of age, period and birth cohort at the global, regional, and national level. Findings from this study will provide a factual basis for preventing NHL in the future.

After eliminating the inconsistency in age composition, the global ASIR and ASMR for NHL were 7.14 (95% UI: 6.58, 7.66) and 3.19 (95% UI: 2.93, 3.44) per 100,000 population in 2021, respectively. Compared with lower SDI regions, the ASIR and ASMR of NHL were higher in the developed regions. This was similar to previous studies suggesting that higher SDI countries shouldered disproportionately higher burden of NHL [15, 17]. The heterogeneity in NHL burden between different regions may be ascribed to risk factor exposures, such as the different pathological subtypes of lymphoma, relatively high incidence of invasive NHL in the developed regions, local economic development and local health systems [16]. Our further analyses estimated the local drift to capture temporal trends in NHL incidence and mortality for each age group. Generally, higher SDI regions had more age groups showing declining mortality whereas lower SDI regions had more age groups showing rising mortality, suggesting a potential SDI-related distributive inequity. The NHL mortality changes observed in this study may be fully commensurate with the general assumption that higher quality in the healthcare system and better medical service were correlated with higher SDI conditions leading to lower disease burden [23].

The relative effects of age, period and birth cohort contributed to incidence and mortality trends have been explored here. Age effects showed a monotonic pattern at global level, with the risk of NHL incidence and mortality rising as the age progressed forward, and similar patterns were found in most regions and countries. The above phenomenon can be partly attributed to population growth and aging. A study showed that the changing age structures and population growth meant that NHL cases and deaths continued to increase across the world [24]. Additionally, we found that the incidence and mortality of NHL in all the SDI regions were lower in young-aged group than those in the old-aged group. NHL occurred more commonly in the elderly due to the association with the aging process that led to changes in the immune system alterations and the long-term latency of EBV infection [25]. HIV infection was another possible driver of the rapidly increasing incidence and mortality of NHL across different countries. Lymphomas remained a leading cause of cancer morbidity and mortality for HIV-infected patients, and an elevated risk of developing NHL persisted among HIV-infected individuals compared to the general population despite the advent of effective antiretroviral therapy [26]. The relative risks of NHL ranged from 15 for low-grade NHL in the general population to 400 for high-grade NHL in patients with acquired immune deficiency syndrome [27]. In countries with a high prevalence of HIV, the incidence of NHL was anticipated to increase. For example, the increased morbidity in Belarus may be related to the high prevalence of HIV and the rapid spread of HIV variants in recent years [28]. However, due to highly active antiretroviral therapy in developed countries, the incidence of NHL has significantly declined, such as Australia [29].

Period effects were defined as the risks of incidence or mortality caused by changes in natural conditions or social environment during specific periods of time [12]. Globally, period effects showed an increase in incidence risks before 1997–2001, followed by a decline between 1997–2001 and 2002–2006. However, period effects for mortality at global level exhibited a persistent downward trend. The period effects on incidence and mortality showed high heterogeneity across most regions and countries. In recent years, increased incidence in NHL has been largely attributed to the continuous improvement of NHL diagnosis technology, leading to increased detection rates and fewer misdiagnoses and misses. For example, advances in laboratory techniques have had a significant impact on the diagnosis and management of hematologic malignancies during the past study period [30]. With the development of new technologies, such as next-generation sequencing, flow cytometry, and molecular genetics, it was now possible to identify specific genetic mutations and biomarkers that can be used to classify and prognosticate hematologic malignancies and contribute to better detection of NHL [31]. Advances in treatments were other possible contributing factors to changes in mortality. Innovations such as stem cell transplantation and new drugs like rituximab and immune checkpoint inhibitors may offer new options for patients with refractory or recurrent NHL [32, 33]. With the development of new treatment strategies and palliative medicine, the survival period of patients with NHL has been extended. However, disparities in access to healthcare persisted. Compared to patients in higher SDI countries, patients in lower often faced challenges in sufficient access to timely treatment, leading to higher mortality risks [34]. The poor medical source, misdiagnosis and omission by lacking high quality pathological diagnosis of NHL, and the heavy burden of chemotherapy drugs on the masses had brought the worse prognosis of NHL patients in Sub-Saharan Africa, such as Malawi [35]. Notably, the rapidly increasing incidence risks and declining mortality risks in China was deeply influenced by local healthcare infrastructure and health systems [36]. Since 1983, China has been emphasizing health information construction, and the addition of medical information systems in each region, and the establishment and improvement of disease registration and reporting systems have expanded the coverage of patients with NHL [37]. These developments may contribute to the increase in incidence risks to levels almost similar to that in high-middle and high SDI countries. The gradual implementation of universal coverage of residents’ basic health insurance nationwide have enhanced the accessibility and utilization of healthcare resources, which may lead to the observed increase in the burden of NHL in China [37].

Cohort effects were defined as the risks of incidence or mortality for people in the same birth cohort at different ages or for people in different cohorts who received exposure to a factor at the same age [38]. Overall, there was an increase in the cohort born before 1927 to 1936, and a decline in 1962 to 1971 and 1967 to 1976 in incidence risks globally. Cohort effects displayed a global pattern of initially rising and then declining mortality risks across successive birth cohorts, with a highest risk of NHL mortality in 1917–1926. One possible reason was the change in dietary habits and lifestyle. According to previous studies, vegetable, fish and alcohol intake have been shown to reduce the risk of NHL, whereas meat and fat intake and obesity were thought to increase the risk [39, 40]. Research has discovered that the westernization of lifestyle may cause the distribution of lymphoma subtypes, indicating the impact of lifestyle changes on the incidence pattern [14]. Another reason may be the increased social stress experienced by the later-born cohort. Given that chronic stress can weaken the original anti-tumor environment of immune cells and accelerate tumor progression, the risks of incidence and mortality of NHL increased to a certain extent [41]. Our analyses found higher peaks of mortality risks existed in higher SDI regions, except in low SDI region. This phenomenon may be attributed to higher BMI in higher SDI regions, due to the proportion of NHL mortality attributed to high BMI had a strong positive correlation with SDI level [42]. A pooled study also illustrated that obesity was causally linked to the development of NHL, especially for the diffuse large B cell lymphoma subtype [43]. However, the cohort risks of NHL incidence and mortality varied among countries, which may be mainly due to imbalances in socio-economic development and insufficient access in health care. Notably, our study showed that there was an overall decreasing trend of birth cohorts since 1907 to 1916 in China. The Chinese government has made substantial progress by enhancing financial protection and reforming the healthcare system to improve access to health services, such as universal health insurance programs, which were likely to be contributing factors [44].

Here, for the first time, we used APC model to comprehensively analyse the temporal trends of NHL incidence and mortality on a global scale from 1992 to 2021. Specifically, we can distinguish and capture the independent effects of age, period, and cohort effects on disease incidence and mortality trends at the global, regional, and national levels. Our findings provided evidence for the evaluation of NHL-related prevention, management and treatment programmes and supported horizontal comparisons between different regions and countries. Despite these advantages, this study has several limitations. First, although GBD 2021 has made several adjusted methods to improve the comparability of data, it was inevitable to introduce bias affecting the completeness and accuracy of the data, such as reporting bias and over-diagnosis bias. Second, our study only analyzed the incidence and mortality of NHL at global, regional and national levels, lacking a more detailed analysis to capture subnational differences. Due to the subnational disparities in health access to healthcare providers and services, evidence-based health decision making at the subnational level was critical for every country. Therefore, future research should incorporate subnational-level research data to comprehensively understand the disease burden of NHL. Finally, APC analysis with the IE method was an ecological study, for which we were unable to make casual inference. Some assumptions can only be made based on the present data and literature. Therefore, further research on the contributing factors should be conducted.

## Conclusions

In conclusion, an increasing overall temporal trend in NHL incidence, coupled with a declining trend in NHL mortality were observed worldwide, highlighting that NHL burden remained a substantial public health challenge. Meanwhile, there was a strong heterogeneity in age, period and birth cohort effects on NHL incidence and mortality across regions and countries, indicating distinct disease patterns worldwide. The scope of prevention and therapy guidelines can be extended to all age groups, with particular attention to vulnerable populations, including middle-aged and aged individuals. Future efforts should be directed toward tailoring NHL control actions following regional risk factor assessments and cancer burden profiles. More detailed investigations into the reasons behind temporal trends and the epidemiology are warranted to optimize the allocation of public health resources and formulate policies more rationally.

## Electronic supplementary material

Below is the link to the electronic supplementary material.

Supplementary Material 1

## Data Availability

The datasets generated during and/or analyzed during the current study are available from the Global Health Data Exchange query tool (https://vizhub.healthdata.org/gbd-results/).
