# Supplementary material for: Global, regional and national temporal trends in incidence and mortality of non-Hodgkin lymphoma from 1992 to 2021: an age-period-cohort analysis
Source: Ann Hematol. 2025 Feb 24;104(3):1679–96. doi: 10.1007/s00277-025-06261-w (PMC12031885; doi:10.1007/s00277-025-06261-w)
Supplement: Supplementary file 1 — Supplementary Material 1 [file 277_2025_6261_MOESM1_ESM.docx]

**Supplementary Material**

**Contents**

**Figure S1 The age-standardized incidence rate in 1992 for non-Hodgkin lymphoma in 204 countries and territories**

**Figure S2 The age-standardized mortality rate in 1992 for non-Hodgkin lymphoma in 204 countries and territories**

**Figure S3 The age-standardized incidence rate in 2021 for non-Hodgkin lymphoma in 204 countries and territories**

**Figure S4 The age-standardized mortality rate in 2021 for non-Hodgkin lymphoma in 204 countries and territories**

**Table S1 Temporal trends in non-Hodgkin lymphoma incidence for both sexes in 204 countries, 1992-2021**

**Table S2 Temporal trends in non-Hodgkin lymphoma mortality for both sexes in 204 countries, 1992-2021**

**Table S3 The local drift of incidence and mortality from 1992 to 2021 for non-Hodgkin lymphoma for different age groups across 204 countries and territories**

**Table S4 Age effects on non-Hodgkin lymphoma incidence and mortality across 204 countries and territories**

**Table S5 Period effects on non-Hodgkin lymphoma incidence and mortality across 204 countries and territories**

**Table S6 Cohort effects on non-Hodgkin lymphoma incidence and mortality across 204 countries and territories**

**Figure S1 The age-standardized incidence rate in 1992 for non-Hodgkin lymphoma in 204 countries and territories**

**
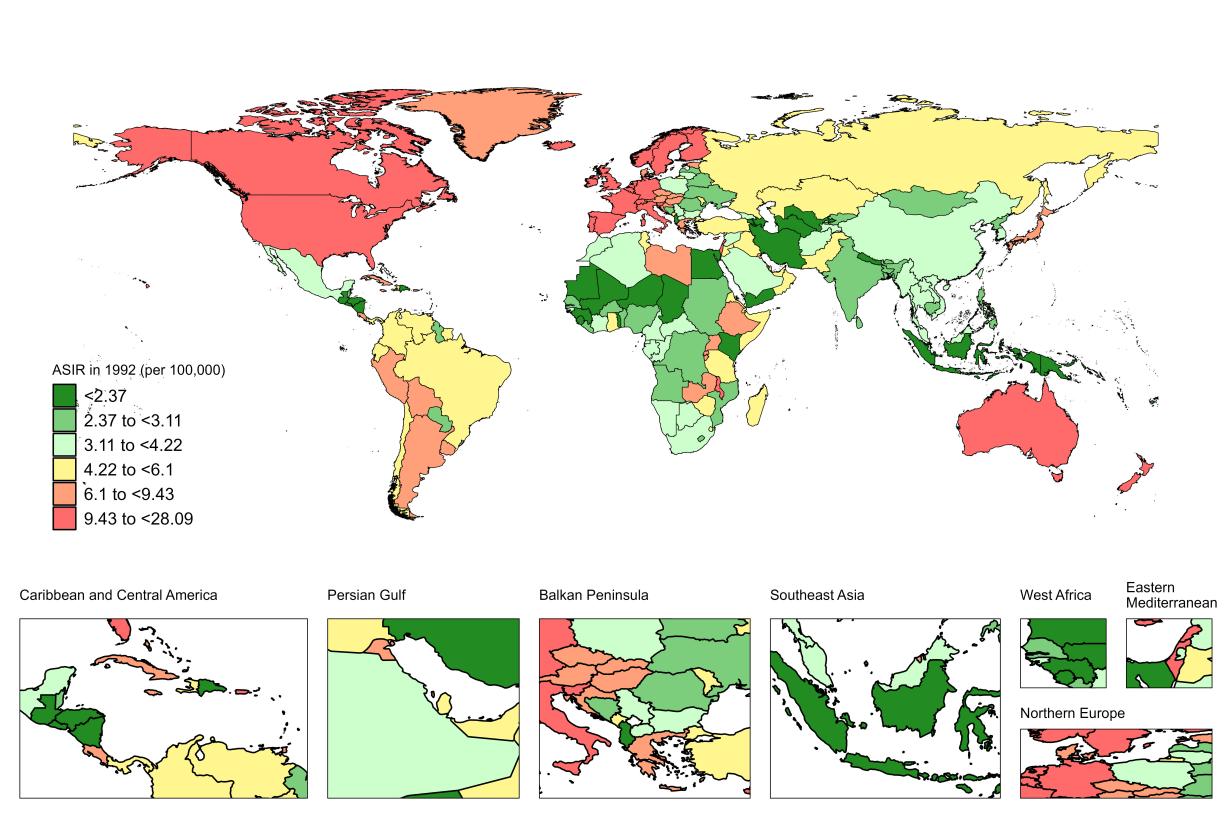
**

**Figure S2 The age-standardized mortality rate in 1992 for non-Hodgkin lymphoma in 204 countries and territories**

**
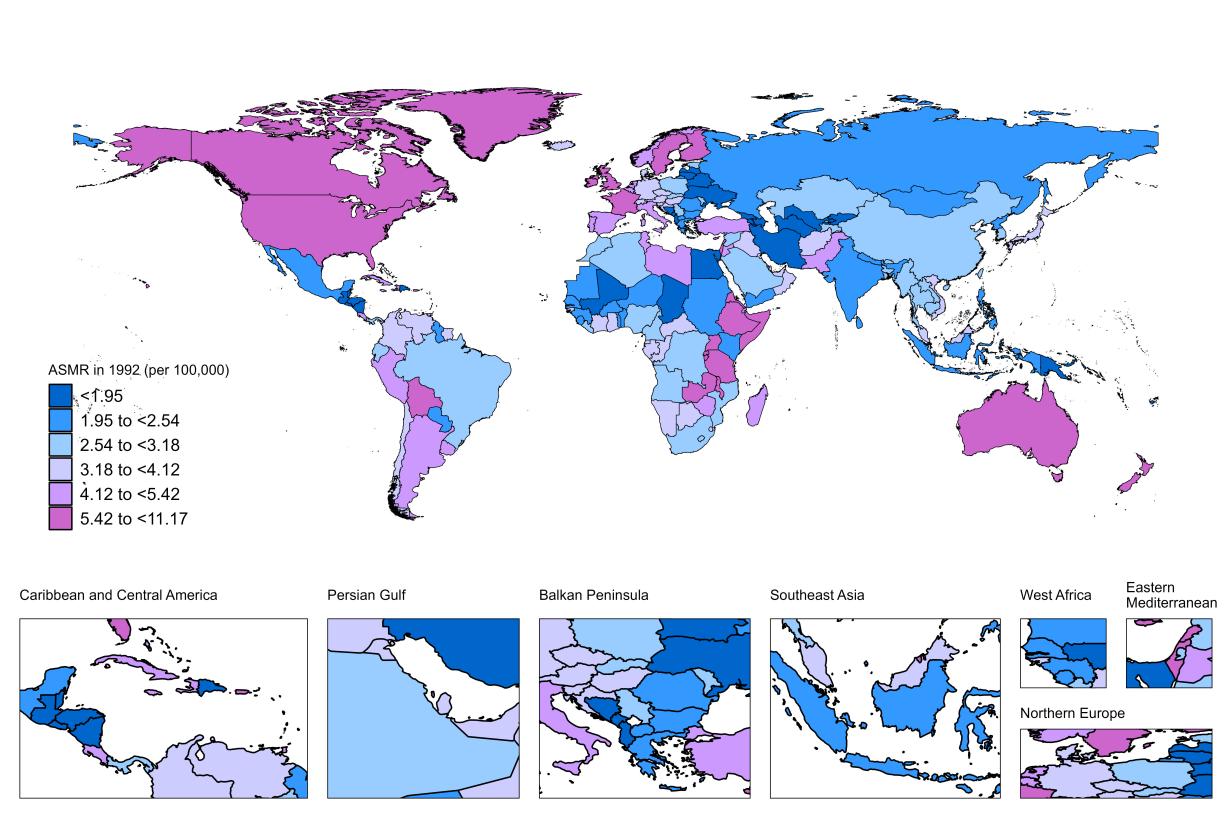
**

**Figure S3 The age-standardized incidence rate in 2021 for non-Hodgkin lymphoma in 204 countries and territories**

**
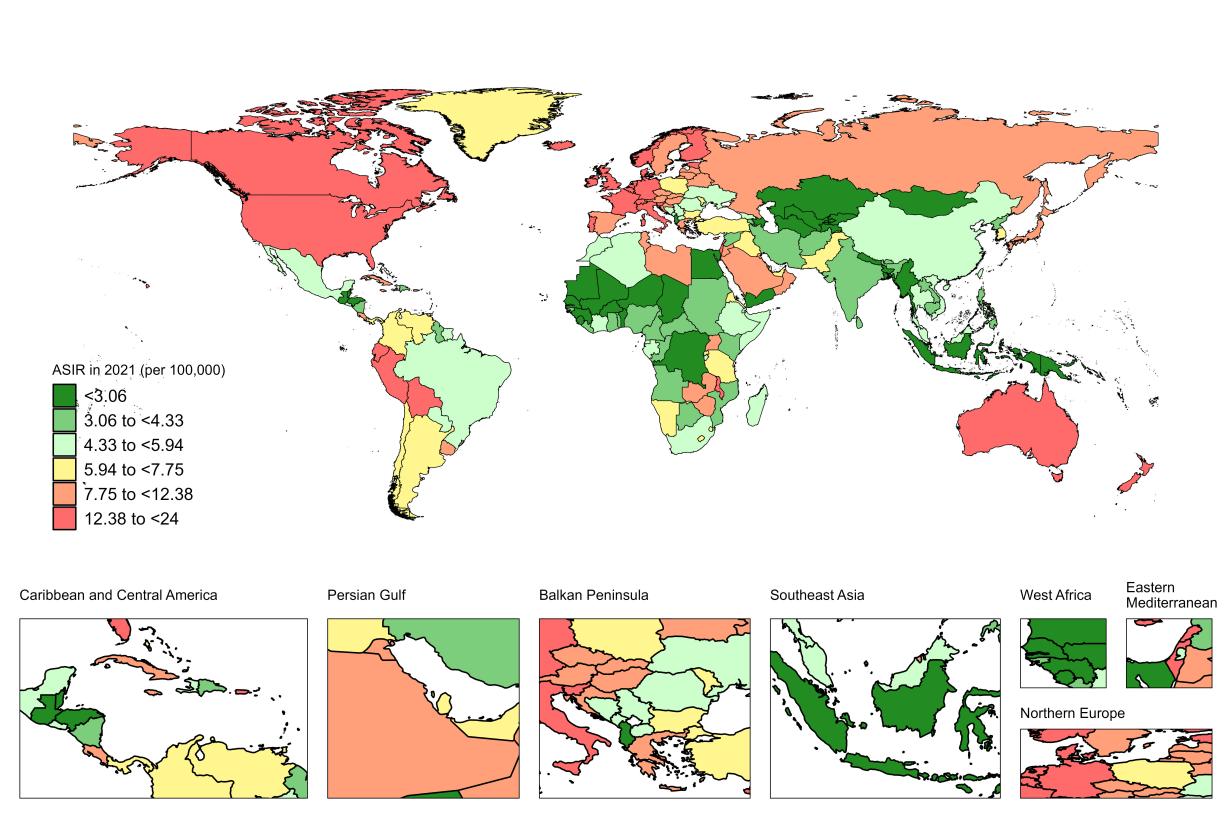
**

**Figure S4 The age-standardized mortality rate in 2021 for non-Hodgkin lymphoma in 204 countries and territories**

**
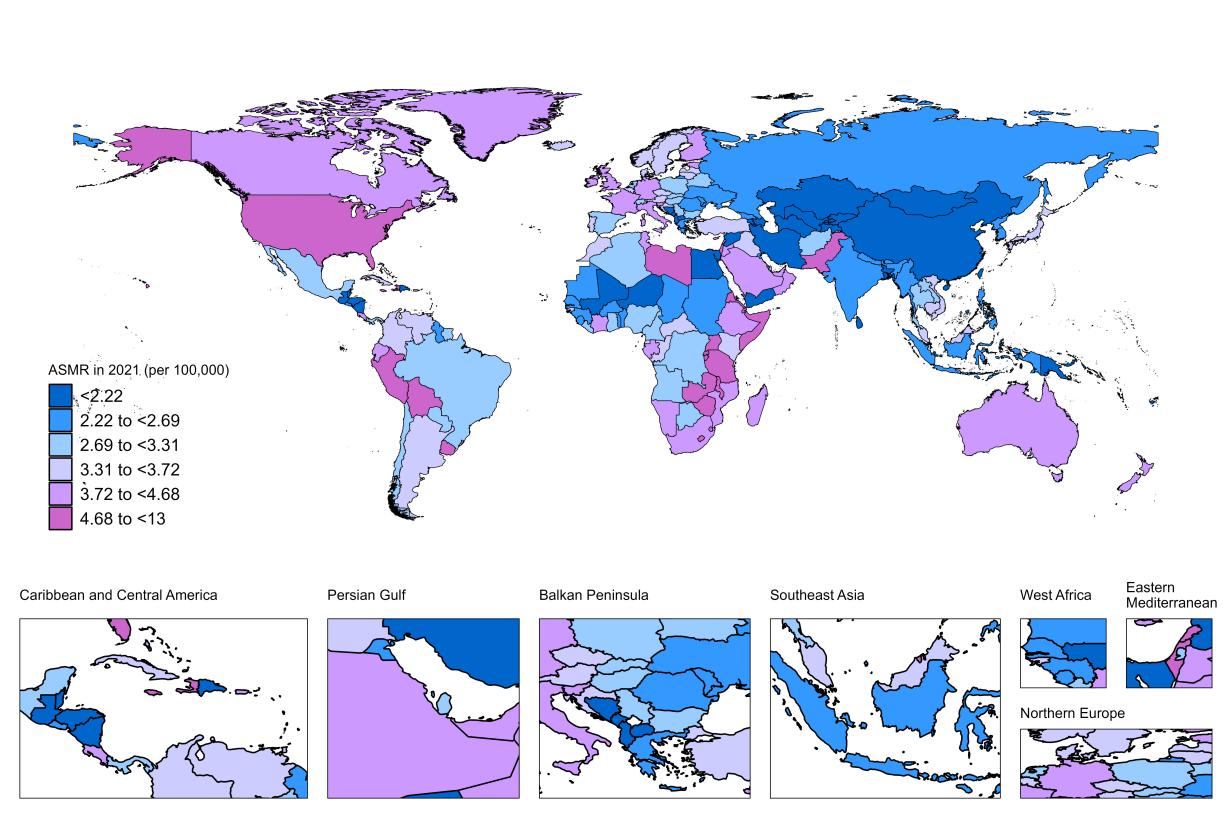
**

**Table S1 Temporal trends in non-Hodgkin lymphoma incidence for both sexes in 204 countries, 1992-2021**

| **SDI** | | **Age-standardized incidence rate** | | **Net drift of incidence from APC model (% per year)** |
| --- | --- | --- | --- | --- |
| **Quintile** | **Country** | **Rate in 1992** | **Rate in 2021** |
| High SDI | Andorra | 25.57 (18.47,34.71) | 22.85 (15.28,30.64) | 0.42 (-2.28,3.19) |
| High SDI | Australia | 14.55 (13.57,15.58) | 15.9 (13.8,18.2) | -0.35 (-0.55,-0.14) |
| High SDI | Austria | 9.35 (8.52,10.22) | 12.01 (10.56,13.46) | 0.5 (0.18,0.83) |
| High SDI | Belgium | 12.43 (11.21,13.74) | 13.93 (12.32,15.94) | 0 (-0.28,0.27) |
| High SDI | Bermuda | 14.35 (13,15.82) | 14.51 (11.84,18.01) | 0.32 (-4.56,5.46) |
| High SDI | Canada | 20.67 (18.57,22.94) | 17.16 (14.7,19.64) | -1.61 (-1.8,-1.43) |
| High SDI | Cyprus | 12.7 (10.79,16.14) | 16.83 (13.31,20.18) | 2.01 (1.13,2.89) |
| High SDI | Czechia | 7.34 (6.57,8.31) | 11.41 (9.41,13.85) | 1.57 (1.23,1.91) |
| High SDI | Denmark | 7.9 (7.37,8.56) | 13.69 (11.99,15.77) | 0.98 (0.5,1.47) |
| High SDI | Estonia | 8.48 (7.18,10) | 21.7 (18.16,25.77) | 2.97 (2.19,3.76) |
| High SDI | Finland | 11.41 (10.59,12.2) | 14.69 (12.89,16.72) | 0.87 (0.46,1.27) |
| High SDI | France | 12.62 (11.45,13.86) | 15.24 (13.22,17.59) | 0.46 (0.34,0.57) |
| High SDI | Germany | 9.89 (9.11,10.77) | 12.97 (11.18,14.67) | -0.45 (-0.57,-0.33) |
| High SDI | Greenland | 8.22 (7.27,9.98) | 7.27 (5.95,9.59) | 2.19 (-4.54,9.4) |
| High SDI | Iceland | 11.14 (9.91,12.35) | 23.81 (20.33,28.43) | 2.48 (1.11,3.86) |
| High SDI | Ireland | 13.02 (11.88,14.4) | 15.95 (13.49,18.35) | 1.35 (0.93,1.78) |
| High SDI | Japan | 7.31 (6.8,7.78) | 10.38 (9.08,11.76) | 0.44 (0.26,0.62) |
| High SDI | Kuwait | 6.37 (5.72,7.14) | 8 (6.49,9.56) | 0.7 (-0.2,1.61) |
| High SDI | Latvia | 2.86 (2.53,3.22) | 7.77 (6.43,9.15) | 3.18 (2.05,4.32) |
| High SDI | Lithuania | 3.53 (3.17,3.96) | 8.49 (7.22,9.96) | 2.53 (1.79,3.27) |
| High SDI | Luxembourg | 11.48 (10.48,12.55) | 13.52 (11.66,15.61) | -0.21 (-1.51,1.1) |
| High SDI | Monaco | 14.37 (9.92,19.15) | 21.01 (14.66,28.22) | 0.32 (-4.64,5.52) |
| High SDI | Netherlands | 14.78 (13.4,16.3) | 17.89 (15.23,20.59) | 0.56 (0.32,0.8) |
| High SDI | New Zealand | 15.88 (14.36,17.45) | 20.34 (17.71,22.78) | 0.22 (-0.17,0.62) |
| High SDI | Norway | 10.92 (9.94,11.93) | 12.75 (11.42,14.4) | 0.34 (-0.09,0.77) |
| High SDI | Poland | 4.1 (3.91,4.3) | 7 (6.32,7.83) | 1.46 (1.23,1.69) |
| High SDI | Puerto Rico | 11.16 (10.28,12.23) | 12.79 (10.55,15.39) | 0.45 (-0.05,0.95) |
| High SDI | Qatar | 4.7 (3.75,6.38) | 7.02 (5.17,9.09) | 1.98 (-0.49,4.52) |
| High SDI | Republic of Korea | 3.58 (3.2,4.98) | 7.13 (4.9,8.51) | 1.87 (1.64,2.1) |
| High SDI | San Marino | 28.09 (22.98,34.26) | 21 (13.83,30.4) | -0.18 (-4.67,4.53) |
| High SDI | Saudi Arabia | 3.55 (2.71,4.49) | 8.89 (6.5,11.08) | 3.86 (3.47,4.25) |
| High SDI | Singapore | 5.47 (4.99,5.94) | 7.86 (6.86,8.99) | 1.11 (0.41,1.82) |
| High SDI | Slovakia | 6.38 (5.61,8.17) | 9.5 (7.15,11.56) | 1.48 (0.95,2.02) |
| High SDI | Slovenia | 11.26 (10,12.6) | 23.82 (19.43,28.99) | 2.9 (2.32,3.49) |
| High SDI | Sweden | 11.04 (10.17,12.07) | 9.66 (8.43,10.83) | -0.19 (-0.56,0.19) |
| High SDI | Switzerland | 11.27 (10.34,12.38) | 12.88 (11.27,14.59) | -0.46 (-0.75,-0.17) |
| High SDI | Taiwan (Province of China) | 5.33 (5.02,5.65) | 7 (6.16,7.87) | 0.4 (0.12,0.69) |
| High SDI | United Arab Emirates | 5.27 (3.81,7.45) | 6.09 (4.76,7.48) | 0.61 (-0.51,1.74) |
| High SDI | United Kingdom | 13.11 (12.64,13.48) | 15.03 (14.18,15.86) | 0.17 (0.04,0.3) |
| High SDI | United States of America | 20.51 (19.53,21.17) | 15.82 (14.53,16.64) | -1.96 (-2.05,-1.87) |
| High SDI | United States Virgin Islands | 7.61 (6.2,9.1) | 7.22 (5.4,9.59) | 0.81 (-3.39,5.19) |
| High-middle SDI | American Samoa | 2.83 (2.33,3.55) | 4.79 (3.61,6.1) | - |
| High-middle SDI | Antigua and Barbuda | 6.7 (6.14,7.33) | 6.31 (5.72,6.88) | 2.77 (-3.82,9.82) |
| High-middle SDI | Argentina | 6.23 (5.84,6.63) | 6.59 (5.92,7.27) | 0.09 (-0.11,0.29) |
| High-middle SDI | Bahamas | 6.58 (6.05,7.15) | 7.4 (5.87,9.25) | 0.64 (-1.82,3.17) |
| High-middle SDI | Bahrain | 6.86 (5.61,8.03) | 6.92 (5.44,8.59) | 0.14 (-1.69,2) |
| High-middle SDI | Barbados | 11.36 (10.51,12.29) | 11.84 (9.16,15.25) | 0.37 (-1.55,2.33) |
| High-middle SDI | Belarus | 3.04 (2.65,3.44) | 9.31 (7.64,11.49) | 4.23 (3.76,4.7) |
| High-middle SDI | Brunei Darussalam | 7.9 (6.71,9.45) | 8.54 (6.96,9.97) | 0.23 (-2.4,2.93) |
| High-middle SDI | Bulgaria | 4.03 (3.71,4.38) | 7.55 (6.39,9.02) | 2.48 (1.98,2.99) |
| High-middle SDI | Chile | 5.15 (4.81,5.52) | 6.53 (5.8,7.37) | 0.82 (0.45,1.19) |
| High-middle SDI | Cook Islands | 2.04 (1.63,2.57) | 3.84 (2.79,5.16) | - |
| High-middle SDI | Croatia | 6.43 (5.79,7.16) | 10.33 (8.7,12.12) | 1.86 (1.3,2.43) |
| High-middle SDI | Dominica | 9.27 (7.81,10.61) | 10.3 (8.17,13.65) | 0.91 (-4.87,7.03) |
| High-middle SDI | Georgia | 3.67 (2.86,4.56) | 4.94 (4.09,5.95) | 1.22 (0.51,1.93) |
| High-middle SDI | Greece | 6.96 (6.31,7.77) | 9.36 (8.35,10.57) | 0.73 (0.38,1.08) |
| High-middle SDI | Guam | 5.85 (4.8,6.6) | 6.26 (5.25,7.73) | 3.17 (-1.02,7.53) |
| High-middle SDI | Hungary | 6.7 (5.96,7.58) | 9.01 (7.58,10.61) | 0.78 (0.4,1.15) |
| High-middle SDI | Israel | 13.29 (12.16,14.52) | 17.44 (15.09,19.67) | 0.54 (0.21,0.86) |
| High-middle SDI | Italy | 15.54 (14.16,17.08) | 16.85 (14.69,18.91) | -0.42 (-0.52,-0.31) |
| High-middle SDI | Jordan | 5.95 (4.64,7.62) | 8.64 (6.76,10.79) | 1.48 (0.85,2.1) |
| High-middle SDI | Kazakhstan | 4.37 (4.03,4.77) | 2.67 (2.27,3.14) | -1.56 (-1.98,-1.13) |
| High-middle SDI | Lebanon | 9.81 (7.8,12.18) | 14.09 (10.54,18.45) | 1.97 (1.43,2.52) |
| High-middle SDI | Libya | 7.01 (5.19,8.84) | 11.77 (8.71,15.36) | 2.54 (1.97,3.11) |
| High-middle SDI | Malaysia | 3.54 (2.95,4.74) | 4.81 (3.92,6.56) | 1.17 (0.8,1.54) |
| High-middle SDI | Malta | 11.74 (10.33,13.09) | 20.36 (17.2,24.04) | 1.66 (0.44,2.89) |
| High-middle SDI | Montenegro | 4.65 (3.9,5.66) | 5.94 (4.66,7.56) | 0.98 (-0.83,2.81) |
| High-middle SDI | Niue | 3.2 (2.48,4.17) | 8.97 (6.84,12) | - |
| High-middle SDI | North Macedonia | 3.11 (2.71,4.12) | 4.43 (3.43,5.5) | 1.39 (0.2,2.6) |
| High-middle SDI | Northern Mariana Islands | 2.56 (1.93,3.94) | 4.44 (3.62,5.11) | - |
| High-middle SDI | Oman | 5.2 (3.56,7.17) | 7.79 (6.07,10.04) | 1.87 (0.87,2.89) |
| High-middle SDI | Palau | 2.14 (1.64,2.73) | 2.83 (2.2,3.53) | - |
| High-middle SDI | Portugal | 10.72 (9.75,11.86) | 15.83 (13.84,18.09) | 1.01 (0.75,1.27) |
| High-middle SDI | Republic of Moldova | 4.83 (4.4,5.34) | 6.44 (5.58,7.52) | 1.89 (1.16,2.63) |
| High-middle SDI | Romania | 2.71 (2.53,2.92) | 5.15 (4.43,5.99) | 1.85 (1.48,2.23) |
| High-middle SDI | Russian Federation | 5.77 (5.63,5.92) | 8.82 (8.13,9.5) | 1.66 (1.49,1.82) |
| High-middle SDI | Saint Kitts and Nevis | 6.86 (6.35,7.36) | 6.71 (5.52,8.08) | 0.61 (-6.19,7.9) |
| High-middle SDI | Serbia | 4.19 (3.39,5.08) | 5.4 (4.33,6.41) | 0.62 (0.1,1.14) |
| High-middle SDI | Seychelles | 4.9 (4.29,6.27) | 6.04 (5.06,7) | 2.91 (-3.4,9.62) |
| High-middle SDI | Spain | 10.96 (9.99,11.98) | 11.54 (9.97,13.35) | -0.74 (-0.93,-0.56) |
| High-middle SDI | Trinidad and Tobago | 6.68 (6.26,7.1) | 6.69 (5.05,8.41) | 0.47 (-0.62,1.58) |
| High-middle SDI | Ukraine | 3.06 (2.76,3.48) | 4.36 (3.25,5.67) | 1.13 (0.91,1.36) |
| Low SDI | Afghanistan | 3.47 (2.16,6.24) | 3.58 (2.35,5.92) | 0.21 (-0.35,0.77) |
| Low SDI | Angola | 3.05 (2.1,4.33) | 3.17 (2.08,4.58) | 0.53 (-0.11,1.18) |
| Low SDI | Benin | 2.38 (1.96,3.01) | 2.7 (2.01,3.4) | 0.61 (-0.42,1.65) |
| Low SDI | Bhutan | 2.94 (2.07,4.14) | 3.17 (2.08,5.42) | 0.28 (-2.83,3.48) |
| Low SDI | Burkina Faso | 2.47 (1.9,3.07) | 2.9 (2.14,3.71) | 1.08 (0.33,1.84) |
| Low SDI | Burundi | 7.13 (4.92,9.92) | 5.12 (3.75,6.94) | -1.33 (-1.96,-0.69) |
| Low SDI | Cambodia | 2.9 (2.17,3.93) | 3.81 (2.82,5.57) | 0.85 (0.24,1.47) |
| Low SDI | Cameroon | 3.15 (2.63,3.8) | 3.91 (2.81,5.18) | 1.04 (0.48,1.61) |
| Low SDI | Central African Republic | 3.8 (2.88,5.1) | 3.67 (2.74,5.01) | -0.21 (-1.45,1.05) |
| Low SDI | Chad | 1.81 (1.38,2.31) | 2.44 (1.66,3.39) | 1.12 (0.07,2.19) |
| Low SDI | Comoros | 5.75 (4.02,7.76) | 6.14 (4.23,8.58) | -0.47 (-2.65,1.75) |
| Low SDI | Côte d'Ivoire | 4.18 (3.38,5.11) | 4.91 (3.62,6.35) | 0.68 (0.17,1.19) |
| Low SDI | Democratic Republic of the Congo | 2.7 (1.91,3.78) | 2.97 (2.06,4.26) | 0.62 (0.26,0.99) |
| Low SDI | Djibouti | 5.06 (3.67,6.77) | 5.92 (4.09,8.41) | 0.03 (-2.28,2.39) |
| Low SDI | Eritrea | 6.03 (4.72,7.35) | 6.15 (4.51,8.31) | 0.17 (-0.81,1.16) |
| Low SDI | Ethiopia | 7.03 (4.92,9.33) | 5.21 (3.84,7.11) | -1.35 (-1.57,-1.13) |
| Low SDI | Gambia | 3.3 (2.55,4.09) | 4.34 (3.09,5.69) | 1.06 (-0.89,3.04) |
| Low SDI | Guinea | 2.36 (1.95,2.85) | 2.57 (1.87,3.4) | 0.83 (-0.17,1.84) |
| Low SDI | Guinea-Bissau | 3.45 (2.62,4.41) | 3.71 (2.81,4.68) | 0.77 (-1.3,2.89) |
| Low SDI | Haiti | 5.76 (4.23,8.15) | 5.45 (3.82,7.91) | 0.12 (-0.45,0.7) |
| Low SDI | Liberia | 2.81 (2.26,3.42) | 3.36 (2.36,4.39) | 1.87 (0.3,3.45) |
| Low SDI | Madagascar | 4.5 (3.56,5.52) | 4.43 (3.17,6.07) | 0.15 (-0.35,0.65) |
| Low SDI | Malawi | 11.74 (9.65,14.07) | 14.72 (10.09,20.54) | 0.9 (0.55,1.25) |
| Low SDI | Mali | 1.76 (1.44,2.09) | 1.69 (1.29,2.13) | 0.26 (-0.76,1.28) |
| Low SDI | Mozambique | 2.8 (1.59,3.41) | 3.93 (2.06,5.21) | 1.82 (1.11,2.52) |
| Low SDI | Nepal | 2.37 (1.77,3.3) | 3.02 (2.2,4.81) | 1.15 (0.63,1.66) |
| Low SDI | Niger | 2.14 (1.61,2.71) | 1.89 (1.2,2.7) | 0.16 (-0.9,1.23) |
| Low SDI | Papua New Guinea | 1.79 (1.21,2.72) | 2.11 (1.45,3.12) | 0.61 (-0.67,1.89) |
| Low SDI | Rwanda | 8.83 (6.58,11.38) | 6.48 (4.86,8.42) | -1.72 (-2.24,-1.2) |
| Low SDI | Senegal | 2.44 (2,2.98) | 2.86 (2.14,3.74) | 1.08 (0.26,1.91) |
| Low SDI | Sierra Leone | 2.35 (1.81,2.96) | 2.7 (2,3.57) | 1.3 (0.08,2.53) |
| Low SDI | Solomon Islands | 2.01 (1.24,3) | 2.91 (2.13,3.97) | 4.92 (-0.31,10.43) |
| Low SDI | Somalia | 5.96 (4.21,7.93) | 5.5 (3.83,7.42) | -0.1 (-0.69,0.5) |
| Low SDI | South Sudan | 5.65 (4.07,7.46) | 6.54 (4.52,8.94) | 0.73 (0.03,1.43) |
| Low SDI | Timor-Leste | 1.81 (1.29,2.51) | 2.17 (1.61,3.07) | 4.01 (-0.23,8.43) |
| Low SDI | Togo | 2.33 (1.87,2.88) | 3.12 (2.23,4.12) | 1.51 (0.25,2.78) |
| Low SDI | Uganda | 7.98 (6.46,9.76) | 10.26 (7.67,13.71) | 0.73 (0.42,1.04) |
| Low SDI | United Republic of Tanzania | 5.9 (4.59,7.4) | 5.94 (4.53,7.57) | 0.03 (-0.26,0.33) |
| Low SDI | Vanuatu | 2.04 (1.43,2.92) | 2.76 (2.16,3.56) | -1.9 (-8.93,5.67) |
| Low SDI | Yemen | 2.22 (1.29,3.88) | 2.54 (1.69,3.87) | 0.59 (-0.07,1.26) |
| Low SDI | Zimbabwe | 5.21 (4.11,6.44) | 10.75 (7.28,13.83) | 2.92 (2.46,3.38) |
| Low-middle SDI | Bangladesh | 2.84 (2.24,3.71) | 2.7 (1.98,3.92) | -0.14 (-0.35,0.07) |
| Low-middle SDI | Belize | 2.53 (2.34,2.72) | 2.9 (2.56,3.35) | 4.82 (-1.16,11.18) |
| Low-middle SDI | Bolivia (Plurinational State of) | 8.64 (6.64,11.03) | 13.88 (9.99,18.96) | 1.31 (0.87,1.76) |
| Low-middle SDI | Cabo Verde | 0.84 (0.65,1.49) | 4.4 (2.53,5.8) | 5.92 (0.92,11.16) |
| Low-middle SDI | Congo | 4.12 (3.26,5.66) | 4.31 (3.2,6.04) | 0.36 (-0.81,1.54) |
| Low-middle SDI | Democratic People's Republic of Korea | 2.58 (1.82,3.56) | 3.11 (2.29,4.51) | 0.86 (0.44,1.28) |
| Low-middle SDI | Dominican Republic | 2.14 (1.82,2.48) | 3.09 (2.35,3.99) | 1.84 (1.1,2.58) |
| Low-middle SDI | Egypt | 2.03 (1.66,3.93) | 2.73 (1.92,5.51) | 0.45 (0.17,0.73) |
| Low-middle SDI | El Salvador | 2.32 (2.11,2.77) | 4.6 (3.46,5.79) | 2.48 (1.65,3.31) |
| Low-middle SDI | Eswatini | 4.61 (3.57,6.29) | 7.47 (4.75,10.83) | 1.77 (-0.04,3.62) |
| Low-middle SDI | Ghana | 4.24 (2.35,5.6) | 3.59 (2.56,4.62) | -1.6 (-2.05,-1.16) |
| Low-middle SDI | Guatemala | 1.73 (1.65,1.82) | 2.21 (1.87,2.57) | 1.31 (0.46,2.18) |
| Low-middle SDI | Honduras | 1.58 (1.3,1.89) | 2.37 (1.69,3.6) | 0.43 (-0.7,1.58) |
| Low-middle SDI | India | 2.55 (2.1,3.02) | 3.08 (2.72,3.53) | 0.54 (0.44,0.65) |
| Low-middle SDI | Kenya | 2.29 (1.62,3.08) | 3.83 (3.03,4.77) | 1.96 (1.49,2.43) |
| Low-middle SDI | Kiribati | 0.69 (0.44,0.83) | 0.82 (0.47,1.11) | - |
| Low-middle SDI | Kyrgyzstan | 2.43 (1.98,2.92) | 1.82 (1.4,2.3) | 0.02 (-1.04,1.09) |
| Low-middle SDI | Lao People's Democratic Republic | 3.2 (2.15,4.76) | 3.09 (2.22,4.75) | 0.02 (-1.01,1.06) |
| Low-middle SDI | Lesotho | 2.84 (2.05,3.91) | 6.38 (4.4,8.69) | 3.35 (1.91,4.81) |
| Low-middle SDI | Marshall Islands | 2.54 (2.08,3.32) | 3.54 (2.5,4.86) | - |
| Low-middle SDI | Mauritania | 2.36 (1.84,2.91) | 2.94 (2.07,3.87) | 1.06 (-0.64,2.78) |
| Low-middle SDI | Micronesia (Federated States of) | 2.95 (2.28,4) | 3.99 (2.88,5.29) | - |
| Low-middle SDI | Mongolia | 2.79 (1.96,3.62) | 2.1 (1.6,2.77) | -0.72 (-2.2,0.78) |
| Low-middle SDI | Morocco | 3.59 (2.88,4.36) | 5 (3.75,6.3) | 1.1 (0.75,1.45) |
| Low-middle SDI | Myanmar | 3.23 (2.17,4.71) | 2.98 (2.14,4.31) | -0.51 (-0.8,-0.21) |
| Low-middle SDI | Namibia | 3.62 (2.95,4.86) | 5.97 (4.39,8.1) | 1.84 (0.38,3.32) |
| Low-middle SDI | Nauru | 3.84 (2.82,5.18) | 5.32 (3.61,7.25) | - |
| Low-middle SDI | Nicaragua | 2.29 (2.04,2.73) | 3.34 (2.73,4.08) | 1.87 (0.82,2.93) |
| Low-middle SDI | Nigeria | 2.86 (2.18,3.57) | 3.39 (2.36,4.38) | 0.81 (0.57,1.05) |
| Low-middle SDI | Pakistan | 5.09 (4.39,6.25) | 6.87 (5.31,8.97) | 0.8 (0.67,0.94) |
| Low-middle SDI | Palestine | 3.97 (2.79,5.22) | 4.91 (3.94,5.86) | 0.86 (-0.27,2) |
| Low-middle SDI | Samoa | 6.28 (5.15,8.02) | 7.05 (5.5,9.4) | 3.68 (-1.17,8.77) |
| Low-middle SDI | Sao Tome and Principe | 2.29 (1.86,2.81) | 3.38 (2.52,4.43) | -2.43 (-9.57,5.27) |
| Low-middle SDI | Sudan | 2.72 (1.59,4.94) | 3.21 (2.25,4.84) | 0.99 (0.53,1.45) |
| Low-middle SDI | Syrian Arab Republic | 3.33 (2.38,4.27) | 3.8 (2.75,5.33) | 0.3 (-0.22,0.81) |
| Low-middle SDI | Tajikistan | 3.65 (2.79,4.79) | 2.8 (2.12,3.64) | -1.21 (-2,-0.41) |
| Low-middle SDI | Tonga | 6.58 (5.32,8.21) | 8.74 (6.49,12.08) | 1.59 (-5.04,8.68) |
| Low-middle SDI | Tuvalu | 2.5 (1.98,3.3) | 3.44 (2.72,4.36) | - |
| Low-middle SDI | Venezuela (Bolivarian Republic of) | 5.22 (4.9,5.55) | 6.67 (5.11,8.58) | 0.97 (0.67,1.27) |
| Low-middle SDI | Viet Nam | 3.55 (2.84,4.47) | 4.99 (3.78,7.03) | 1.71 (1.5,1.92) |
| Low-middle SDI | Zambia | 6.73 (5.27,8.27) | 7.97 (5.17,13.76) | 0.72 (0.23,1.22) |
| Middle SDI | Albania | 1.55 (1.27,1.91) | 2.37 (1.76,3.1) | 2.23 (0.83,3.66) |
| Middle SDI | Algeria | 3.59 (2.98,4.36) | 4.94 (3.92,6.27) | 1.27 (0.93,1.61) |
| Middle SDI | Armenia | 2.08 (1.74,2.43) | 3.44 (2.78,4.08) | 3.04 (1.83,4.27) |
| Middle SDI | Azerbaijan | 1.91 (1.41,2.46) | 1.85 (1.33,2.49) | 0.15 (-0.68,0.99) |
| Middle SDI | Bosnia and Herzegovina | 2.74 (2.28,3.48) | 5.08 (3.57,6.46) | 2.86 (1.98,3.76) |
| Middle SDI | Botswana | 3.78 (2.59,5.26) | 4.12 (3.08,5.67) | 0.36 (-1.11,1.84) |
| Middle SDI | Brazil | 4.41 (4.23,4.56) | 5.22 (4.9,5.5) | 0.26 (0.15,0.38) |
| Middle SDI | China | 3.38 (2.93,4.08) | 5.53 (4.36,6.68) | 2.18 (2.04,2.33) |
| Middle SDI | Colombia | 5.41 (5.03,5.74) | 7.74 (6.46,9.42) | 1.31 (1.09,1.54) |
| Middle SDI | Costa Rica | 8.6 (7.77,9.43) | 12.31 (10.56,14.08) | 1.38 (0.79,1.96) |
| Middle SDI | Cuba | 8.79 (8.09,9.55) | 8.96 (7.62,10.4) | 0.23 (-0.1,0.56) |
| Middle SDI | Ecuador | 5.8 (5.29,6.35) | 16.26 (12.17,21.09) | 3.06 (2.71,3.4) |
| Middle SDI | Equatorial Guinea | 3.28 (2.35,4.53) | 4.43 (2.83,6.48) | 0.88 (-2.46,4.32) |
| Middle SDI | Fiji | 2.07 (1.7,2.73) | 3.28 (2.35,4.27) | 0.93 (-1.57,3.5) |
| Middle SDI | Gabon | 4.19 (3.29,5.53) | 5.22 (3.8,7.19) | 0.79 (-0.87,2.47) |
| Middle SDI | Grenada | 14.43 (13.12,15.86) | 16.49 (14.08,18.92) | 0.21 (-2.85,3.36) |
| Middle SDI | Guyana | 2.82 (2.46,3.16) | 3.64 (2.76,4.64) | 1.44 (-1,3.93) |
| Middle SDI | Indonesia | 2.36 (1.8,3.22) | 2.87 (2.12,4.16) | 0.61 (0.46,0.76) |
| Middle SDI | Iran (Islamic Republic of) | 2.23 (1.85,2.72) | 4.09 (3.2,4.7) | 2.76 (2.5,3.03) |
| Middle SDI | Iraq | 4.69 (3.47,5.94) | 6.14 (4.5,7.89) | 0.94 (0.61,1.28) |
| Middle SDI | Jamaica | 6.93 (6.14,7.92) | 9.28 (7.18,12.19) | 0.3 (-0.42,1.04) |
| Middle SDI | Maldives | 2.63 (1.89,3.47) | 2.19 (1.75,2.65) | 2.31 (-3.55,8.53) |
| Middle SDI | Mauritius | 1.7 (1.61,1.8) | 3.74 (3.4,4.03) | 2.13 (-0.06,4.37) |
| Middle SDI | Mexico | 3.54 (3.44,3.64) | 5.7 (5.03,6.35) | 1.22 (1.06,1.39) |
| Middle SDI | Panama | 4.58 (4.19,4.98) | 7.46 (5.93,9.07) | 1.66 (0.81,2.52) |
| Middle SDI | Paraguay | 3.1 (2.5,3.9) | 5.08 (3.83,6.55) | 1.59 (0.75,2.44) |
| Middle SDI | Peru | 8.37 (7.15,9.96) | 24 (17.61,31.1) | 3.44 (3.23,3.65) |
| Middle SDI | Philippines | 2.53 (2.25,2.92) | 3.07 (2.57,3.88) | 0.51 (0.27,0.75) |
| Middle SDI | Saint Lucia | 9.95 (9.27,10.72) | 8.94 (7.28,10.82) | -1.31 (-4.17,1.64) |
| Middle SDI | Saint Vincent and the Grenadines | 11.15 (10.21,12.17) | 10.48 (9.14,12.15) | -1.01 (-4.19,2.27) |
| Middle SDI | South Africa | 3.75 (3.25,4.35) | 5.46 (4.33,6.08) | 1.5 (1.2,1.8) |
| Middle SDI | Sri Lanka | 2.59 (2.3,3.25) | 3.38 (2.3,4.67) | 0.93 (0.49,1.37) |
| Middle SDI | Suriname | 5.14 (4.45,5.77) | 4.89 (3.68,6.23) | 0.54 (-1.68,2.81) |
| Middle SDI | Thailand | 3.7 (3.09,5.03) | 5.19 (3.96,7.35) | 0.57 (0.38,0.76) |
| Middle SDI | Tokelau | 2.56 (1.88,3.39) | 7.65 (5.48,10.91) | - |
| Middle SDI | Tunisia | 5.78 (4.9,6.81) | 8.19 (5.85,11.21) | 1.56 (1.12,2) |
| Middle SDI | Türkiye | 5.92 (4.93,7.2) | 7.58 (5.99,9.72) | 1.05 (0.89,1.21) |
| Middle SDI | Turkmenistan | 0.83 (0.72,0.97) | 1.31 (0.99,1.72) | 1.57 (-0.27,3.45) |
| Middle SDI | Uruguay | 7.94 (7.37,8.63) | 11.24 (10.1,12.41) | 0.77 (0.19,1.35) |
| Middle SDI | Uzbekistan | 2.3 (1.88,2.79) | 2.32 (1.85,2.86) | -0.1 (-0.55,0.35) |

**Table S2 Temporal trends in non-Hodgkin lymphoma mortality for both sexes in 204 countries, 1992-2021**

| **SDI** | | **Age-standardized mortality rate** | | **Net drift of mortality from APC model (% per year)** |
| --- | --- | --- | --- | --- |
| **Quintile** | **Country** | **Rate in 1992** | **Rate in 2021** |
| High SDI | Andorra | 8.65 (6.45,11.65) | 4.94 (3.42,6.59) | -0.99 (-6.84,5.22) |
| High SDI | Australia | 6.45 (6.09,6.8) | 4.31 (3.83,4.7) | -2.86 (-3.41,-2.3) |
| High SDI | Austria | 3.99 (3.76,4.19) | 3.51 (3.14,3.77) | -1.26 (-2.17,-0.35) |
| High SDI | Belgium | 4.96 (4.59,5.26) | 3.64 (3.24,4.06) | -2 (-2.78,-1.22) |
| High SDI | Bermuda | 7.23 (6.74,7.82) | 3.74 (3.14,4.6) | 0.29 (-6.45,7.52) |
| High SDI | Canada | 6.35 (5.97,6.7) | 4.49 (3.97,4.95) | -2.62 (-3.07,-2.18) |
| High SDI | Cyprus | 6.44 (5.25,8.2) | 3.86 (3.18,4.52) | -1.01 (-1.57,-0.44) |
| High SDI | Czechia | 3.89 (3.55,4.29) | 3.47 (2.9,4.12) | -1.66 (-4.57,1.34) |
| High SDI | Denmark | 3.46 (3.26,3.64) | 3.56 (3.19,3.88) | -2.46 (-3.89,-1.01) |
| High SDI | Estonia | 2.61 (2.35,2.9) | 4.02 (3.45,4.56) | -0.36 (-2.66,2) |
| High SDI | Finland | 5.56 (5.24,5.89) | 4.12 (3.66,4.53) | -1.63 (-2.73,-0.52) |
| High SDI | France | 5.44 (5.11,5.73) | 3.98 (3.54,4.43) | -2.2 (-2.52,-1.88) |
| High SDI | Germany | 4.04 (3.78,4.29) | 3.83 (3.37,4.18) | -2.1 (-2.42,-1.77) |
| High SDI | Greenland | 6.72 (5.93,8.34) | 4.39 (3.64,5.84) | - |
| High SDI | Iceland | 3.27 (3,3.54) | 3.4 (2.98,3.8) | -0.74 (-6.11,4.94) |
| High SDI | Ireland | 5.6 (5.3,5.94) | 3.76 (3.29,4.15) | -1.79 (-3.01,-0.55) |
| High SDI | Japan | 3.72 (3.5,3.84) | 3.61 (3.18,3.86) | -1.73 (-1.99,-1.46) |
| High SDI | Kuwait | 3.32 (3,3.67) | 2.63 (2.17,3.12) | -1.56 (-3.02,-0.09) |
| High SDI | Latvia | 1.78 (1.61,1.97) | 3.63 (3.12,4.18) | 1.75 (0,3.52) |
| High SDI | Lithuania | 1.84 (1.69,2.01) | 3.44 (3.01,3.9) | 1.3 (-0.2,2.83) |
| High SDI | Luxembourg | 5.1 (4.83,5.38) | 3.52 (3.12,3.86) | -2.69 (-7.01,1.82) |
| High SDI | Monaco | 4.54 (3.16,5.96) | 4.77 (3.38,6.26) | -0.35 (-7.16,6.95) |
| High SDI | Netherlands | 4.35 (4.08,4.61) | 3.19 (2.85,3.49) | -1.94 (-2.63,-1.24) |
| High SDI | New Zealand | 6.28 (5.83,6.71) | 4.64 (4.17,5.1) | -2.66 (-3.94,-1.36) |
| High SDI | Norway | 5.07 (4.8,5.31) | 3.53 (3.19,3.8) | -2.63 (-3.99,-1.25) |
| High SDI | Poland | 2.8 (2.72,2.88) | 3.2 (2.93,3.46) | -0.71 (-1.09,-0.33) |
| High SDI | Puerto Rico | 5.71 (5.4,6.02) | 4.07 (3.41,4.82) | -1.56 (-2.57,-0.54) |
| High SDI | Qatar | 3.34 (2.67,4.4) | 2.72 (2.04,3.48) | -1.9 (-5.36,1.68) |
| High SDI | Republic of Korea | 2.68 (2.4,3.84) | 2.51 (1.67,2.97) | -2.13 (-2.52,-1.74) |
| High SDI | San Marino | 9.04 (7.49,10.98) | 4.68 (3.13,6.59) | -1.03 (-7.78,6.21) |
| High SDI | Saudi Arabia | 2.89 (2.22,3.65) | 4.38 (3.13,5.37) | 1.32 (0.82,1.82) |
| High SDI | Singapore | 3.68 (3.43,3.94) | 2.88 (2.55,3.14) | -2.1 (-3.37,-0.82) |
| High SDI | Slovakia | 3.14 (2.81,4.06) | 3.12 (2.36,3.78) | -0.5 (-1.52,0.54) |
| High SDI | Slovenia | 3.9 (3.63,4.16) | 4.64 (3.91,5.34) | -0.17 (-2.15,1.85) |
| High SDI | Sweden | 5.61 (5.25,5.9) | 3.53 (3.09,3.93) | -2.3 (-3.35,-1.23) |
| High SDI | Switzerland | 3.7 (3.46,4.03) | 3.02 (2.67,3.37) | -2.57 (-3.55,-1.58) |
| High SDI | Taiwan (Province of China) | 3.77 (3.57,3.95) | 3.62 (3.26,4.02) | -1.11 (-1.56,-0.65) |
| High SDI | United Arab Emirates | 4.07 (2.91,5.81) | 3.85 (2.97,4.71) | -0.74 (-2.06,0.6) |
| High SDI | United Kingdom | 5.54 (5.34,5.65) | 4.23 (3.89,4.4) | -2.08 (-2.39,-1.76) |
| High SDI | United States of America | 7.38 (6.93,7.63) | 4.69 (4.21,4.93) | -3.22 (-3.37,-3.08) |
| High SDI | United States Virgin Islands | 4.54 (3.7,5.34) | 3.38 (2.55,4.42) | 1.2 (-5.39,8.25) |
| High-middle SDI | American Samoa | 1.96 (1.64,2.45) | 2.75 (2.11,3.43) | - |
| High-middle SDI | Antigua and Barbuda | 4.18 (3.85,4.47) | 3.21 (3.01,3.41) | - |
| High-middle SDI | Argentina | 4.51 (4.27,4.74) | 3.65 (3.33,3.99) | -1.11 (-1.42,-0.81) |
| High-middle SDI | Bahamas | 4.59 (4.26,4.94) | 4.15 (3.37,5.12) | -0.98 (-3.93,2.06) |
| High-middle SDI | Bahrain | 5.41 (4.47,6.36) | 3.35 (2.68,4.1) | -2.5 (-5.16,0.24) |
| High-middle SDI | Barbados | 6.85 (6.43,7.27) | 5.81 (4.64,7.2) | -1.05 (-4.03,2.03) |
| High-middle SDI | Belarus | 1.61 (1.44,1.78) | 2.9 (2.38,3.54) | 1.7 (0.81,2.59) |
| High-middle SDI | Brunei Darussalam | 6.98 (5.89,8.21) | 6.2 (5.03,7.21) | -1.04 (-4.49,2.53) |
| High-middle SDI | Bulgaria | 2 (1.9,2.1) | 2.8 (2.45,3.2) | 0.94 (0.06,1.83) |
| High-middle SDI | Chile | 3.95 (3.72,4.17) | 3.03 (2.74,3.29) | -1.49 (-2.05,-0.92) |
| High-middle SDI | Cook Islands | 1.23 (1,1.52) | 1.31 (1,1.65) | - |
| High-middle SDI | Croatia | 3.34 (3.03,3.66) | 3.65 (3.13,4.22) | 0.2 (-0.36,0.76) |
| High-middle SDI | Dominica | 6.8 (5.82,7.74) | 6.77 (5.46,8.88) | 1.41 (-4.96,8.2) |
| High-middle SDI | Georgia | 1.94 (1.53,2.36) | 2.68 (2.23,3.22) | 1.12 (0.07,2.18) |
| High-middle SDI | Greece | 2.47 (2.33,2.61) | 2.67 (2.41,2.87) | -0.36 (-1.46,0.75) |
| High-middle SDI | Guam | 3.14 (2.59,3.51) | 2.48 (2.12,2.92) | 1.61 (-5.2,8.9) |
| High-middle SDI | Hungary | 3.79 (3.37,4.22) | 3.15 (2.66,3.67) | -1.18 (-1.92,-0.45) |
| High-middle SDI | Israel | 6.68 (6.16,7.09) | 5.42 (4.71,5.95) | -1.83 (-2.6,-1.05) |
| High-middle SDI | Italy | 5.11 (4.79,5.31) | 3.85 (3.43,4.11) | -2.36 (-2.67,-2.05) |
| High-middle SDI | Jordan | 4.35 (3.39,5.63) | 3.97 (3.03,4.97) | -0.98 (-1.81,-0.15) |
| High-middle SDI | Kazakhstan | 2.77 (2.59,2.96) | 1.29 (1.1,1.48) | -3.24 (-3.82,-2.65) |
| High-middle SDI | Lebanon | 7.24 (5.86,8.9) | 6.32 (4.75,8.01) | -0.45 (-1.31,0.43) |
| High-middle SDI | Libya | 5.04 (3.71,6.38) | 6.72 (5.03,8.89) | 1.16 (0.38,1.95) |
| High-middle SDI | Malaysia | 3.21 (2.66,4.38) | 3.41 (2.74,4.78) | -0.05 (-0.49,0.4) |
| High-middle SDI | Malta | 4.27 (3.88,4.6) | 3.62 (3.16,4.05) | 0.67 (-3.84,5.4) |
| High-middle SDI | Montenegro | 1.94 (1.65,2.32) | 2.11 (1.71,2.56) | -0.28 (-4.98,4.66) |
| High-middle SDI | Niue | 2.18 (1.71,2.79) | 3.04 (2.44,3.65) | - |
| High-middle SDI | North Macedonia | 2.08 (1.82,2.73) | 2.08 (1.63,2.54) | -1.85 (-3.93,0.28) |
| High-middle SDI | Northern Mariana Islands | 1.51 (1.19,2.38) | 2.23 (1.83,2.52) | - |
| High-middle SDI | Oman | 3.84 (2.6,5.35) | 3.74 (2.85,4.66) | -0.38 (-1.81,1.07) |
| High-middle SDI | Palau | 1.41 (1.12,1.77) | 1.44 (1.14,1.78) | - |
| High-middle SDI | Portugal | 4.14 (3.92,4.36) | 3.72 (3.38,4.05) | -1.82 (-2.57,-1.07) |
| High-middle SDI | Republic of Moldova | 2.58 (2.43,2.73) | 2.59 (2.29,2.98) | 0.37 (-0.79,1.55) |
| High-middle SDI | Romania | 1.97 (1.85,2.09) | 2.56 (2.23,2.91) | 0.09 (-0.43,0.61) |
| High-middle SDI | Russian Federation | 2.27 (2.22,2.33) | 2.56 (2.38,2.77) | -0.3 (-0.5,-0.1) |
| High-middle SDI | Saint Kitts and Nevis | 5.33 (4.98,5.68) | 4.02 (3.32,4.74) | - |
| High-middle SDI | Serbia | 2.76 (2.25,3.47) | 2.71 (2.19,3.22) | -0.9 (-1.77,-0.03) |
| High-middle SDI | Seychelles | 4.32 (3.79,5.49) | 4.46 (3.74,5.13) | 2.28 (-4.3,9.3) |
| High-middle SDI | Spain | 4.17 (3.91,4.39) | 3.15 (2.82,3.48) | -2.49 (-2.89,-2.09) |
| High-middle SDI | Trinidad and Tobago | 4.74 (4.51,4.97) | 3.69 (2.83,4.73) | -0.52 (-2.11,1.09) |
| High-middle SDI | Ukraine | 1.72 (1.57,1.93) | 2.24 (1.66,2.88) | 0.59 (0.25,0.92) |
| Low SDI | Afghanistan | 3.3 (2.09,5.86) | 3.09 (2.01,5.04) | -0.25 (-0.85,0.36) |
| Low SDI | Angola | 2.95 (2.02,4.22) | 2.82 (1.84,4.16) | 0.12 (-0.57,0.82) |
| Low SDI | Benin | 2.23 (1.84,2.8) | 2.33 (1.76,2.94) | 0.47 (-0.68,1.63) |
| Low SDI | Bhutan | 2.72 (1.91,3.86) | 2.44 (1.62,4.07) | -0.69 (-5.77,4.68) |
| Low SDI | Burkina Faso | 2.33 (1.78,2.89) | 2.57 (1.92,3.29) | 0.87 (0.04,1.72) |
| Low SDI | Burundi | 6.94 (4.81,9.65) | 4.81 (3.55,6.45) | -1.59 (-2.27,-0.91) |
| Low SDI | Cambodia | 2.85 (2.14,3.82) | 3.33 (2.5,4.84) | 0.13 (-0.52,0.79) |
| Low SDI | Cameroon | 2.92 (2.45,3.53) | 3.29 (2.4,4.36) | 0.68 (0.05,1.31) |
| Low SDI | Central African Republic | 3.71 (2.81,4.93) | 3.52 (2.66,4.83) | -0.01 (-1.29,1.29) |
| Low SDI | Chad | 1.73 (1.33,2.2) | 2.23 (1.54,3.05) | 0.95 (-0.19,2.11) |
| Low SDI | Comoros | 5.49 (3.92,7.37) | 5.48 (3.79,7.69) | -1.35 (-3.62,0.97) |
| Low SDI | Côte d'Ivoire | 3.98 (3.24,4.84) | 4.25 (3.18,5.48) | -0.62 (-1.38,0.15) |
| Low SDI | Democratic Republic of the Congo | 2.61 (1.82,3.71) | 2.69 (1.83,3.94) | 0.34 (-0.06,0.73) |
| Low SDI | Djibouti | 4.81 (3.54,6.45) | 5.27 (3.62,7.53) | -0.48 (-2.97,2.08) |
| Low SDI | Eritrea | 5.85 (4.57,7.12) | 5.73 (4.24,7.67) | -0.07 (-1.11,0.97) |
| Low SDI | Ethiopia | 6.88 (4.75,9.1) | 4.62 (3.42,6.34) | -1.95 (-2.18,-1.71) |
| Low SDI | Gambia | 3.03 (2.37,3.73) | 3.75 (2.61,4.94) | 0 (-2.08,2.12) |
| Low SDI | Guinea | 2.25 (1.87,2.71) | 2.36 (1.74,3.1) | 0.47 (-0.63,1.58) |
| Low SDI | Guinea-Bissau | 3.31 (2.56,4.24) | 3.37 (2.57,4.23) | 0.21 (-1.93,2.41) |
| Low SDI | Haiti | 5.32 (3.92,7.46) | 4.76 (3.33,6.84) | -0.18 (-0.79,0.43) |
| Low SDI | Liberia | 2.64 (2.14,3.23) | 2.8 (2,3.66) | 1.26 (-0.45,2.99) |
| Low SDI | Madagascar | 4.3 (3.39,5.29) | 4.01 (2.89,5.54) | -0.11 (-0.65,0.43) |
| Low SDI | Malawi | 11.17 (9.26,13.34) | 13 (9.05,18.24) | 0.52 (0.14,0.9) |
| Low SDI | Mali | 1.68 (1.38,2.01) | 1.54 (1.18,1.92) | 0.12 (-1,1.26) |
| Low SDI | Mozambique | 2.87 (1.56,3.52) | 3.86 (1.94,5.14) | 1.65 (0.87,2.43) |
| Low SDI | Nepal | 2.21 (1.63,3.09) | 2.45 (1.82,3.85) | 0.27 (-0.31,0.87) |
| Low SDI | Niger | 2.03 (1.54,2.58) | 1.73 (1.12,2.46) | -0.11 (-1.16,0.95) |
| Low SDI | Papua New Guinea | 1.4 (0.97,2.16) | 1.52 (1.04,2.37) | 0.27 (-1.18,1.74) |
| Low SDI | Rwanda | 8.57 (6.42,10.98) | 5.79 (4.33,7.52) | -2.3 (-2.86,-1.74) |
| Low SDI | Senegal | 2.25 (1.86,2.74) | 2.43 (1.83,3.18) | 0.79 (-0.13,1.72) |
| Low SDI | Sierra Leone | 2.2 (1.71,2.76) | 2.34 (1.76,3.07) | 1.02 (-0.34,2.39) |
| Low SDI | Solomon Islands | 1.74 (1.09,2.55) | 2.23 (1.63,3.05) | 5.5 (-0.6,11.97) |
| Low SDI | Somalia | 5.84 (4.11,7.83) | 5.31 (3.71,7.13) | -0.18 (-0.8,0.44) |
| Low SDI | South Sudan | 5.42 (3.87,7.2) | 5.93 (4.1,8.16) | 0.48 (-0.28,1.25) |
| Low SDI | Timor-Leste | 1.77 (1.25,2.45) | 1.97 (1.45,2.78) | 1.65 (-3.04,6.58) |
| Low SDI | Togo | 2.15 (1.75,2.62) | 2.65 (1.91,3.51) | 1.02 (-0.33,2.38) |
| Low SDI | Uganda | 7.69 (6.19,9.48) | 9.05 (6.8,12.03) | 0.33 (-0.01,0.67) |
| Low SDI | United Republic of Tanzania | 5.56 (4.31,6.97) | 5.18 (4.03,6.61) | -0.31 (-0.64,0.02) |
| Low SDI | Vanuatu | 1.69 (1.18,2.44) | 2.11 (1.65,2.72) | - |
| Low SDI | Yemen | 1.97 (1.15,3.42) | 2.03 (1.36,3.11) | -0.14 (-0.9,0.63) |
| Low SDI | Zimbabwe | 4.71 (3.69,5.78) | 9.46 (6.35,12.04) | 3.09 (2.57,3.62) |
| Low-middle SDI | Bangladesh | 2.61 (2.04,3.42) | 1.95 (1.43,2.82) | -1.36 (-1.61,-1.11) |
| Low-middle SDI | Belize | 1.73 (1.63,1.84) | 1.76 (1.57,2) | -1.78 (-8.54,5.49) |
| Low-middle SDI | Bolivia (Plurinational State of) | 6.01 (4.66,7.59) | 6.17 (4.57,8.29) | -0.49 (-1.11,0.13) |
| Low-middle SDI | Cabo Verde | 0.65 (0.5,1.2) | 2.7 (1.68,3.51) | 3.5 (-2.13,9.45) |
| Low-middle SDI | Congo | 3.93 (3.1,5.35) | 3.72 (2.77,5.16) | -0.42 (-1.67,0.85) |
| Low-middle SDI | Democratic People's Republic of Korea | 2.36 (1.68,3.25) | 2.43 (1.8,3.51) | 0.21 (-0.27,0.69) |
| Low-middle SDI | Dominican Republic | 1.63 (1.4,1.89) | 1.95 (1.48,2.51) | 1.31 (0.4,2.22) |
| Low-middle SDI | Egypt | 1.69 (1.36,3.44) | 1.74 (1.23,3.62) | -0.8 (-1.14,-0.46) |
| Low-middle SDI | El Salvador | 1.72 (1.57,2.06) | 2.23 (1.71,2.78) | 0.81 (-0.32,1.94) |
| Low-middle SDI | Eswatini | 4.11 (3.22,5.62) | 6.04 (3.99,8.68) | 1.23 (-0.65,3.15) |
| Low-middle SDI | Ghana | 3.71 (2.13,4.88) | 2.95 (2.1,3.78) | -1.95 (-2.43,-1.47) |
| Low-middle SDI | Guatemala | 1.49 (1.43,1.56) | 1.47 (1.27,1.72) | 0.35 (-0.67,1.38) |
| Low-middle SDI | Honduras | 1.29 (1.06,1.56) | 1.77 (1.25,2.67) | -0.06 (-1.45,1.35) |
| Low-middle SDI | India | 2.31 (1.9,2.74) | 2.32 (2.06,2.69) | -0.5 (-0.62,-0.38) |
| Low-middle SDI | Kenya | 2.09 (1.47,2.85) | 3.32 (2.64,4.16) | 1.71 (1.17,2.25) |
| Low-middle SDI | Kiribati | 0.6 (0.38,0.73) | 0.66 (0.38,0.88) | - |
| Low-middle SDI | Kyrgyzstan | 1.81 (1.48,2.17) | 0.96 (0.78,1.19) | -1.14 (-2.52,0.27) |
| Low-middle SDI | Lao People's Democratic Republic | 3.17 (2.16,4.69) | 2.8 (2.01,4.36) | -0.55 (-1.55,0.45) |
| Low-middle SDI | Lesotho | 2.63 (1.91,3.64) | 5.6 (3.89,7.61) | 3.07 (1.48,4.69) |
| Low-middle SDI | Marshall Islands | 2.12 (1.76,2.77) | 2.62 (1.89,3.61) | - |
| Low-middle SDI | Mauritania | 2.23 (1.74,2.74) | 2.28 (1.63,3.01) | -0.3 (-2.12,1.55) |
| Low-middle SDI | Micronesia (Federated States of) | 2.46 (1.91,3.32) | 2.84 (2.08,3.83) | - |
| Low-middle SDI | Mongolia | 2.33 (1.64,3) | 1.34 (1.01,1.75) | -2.24 (-3.91,-0.54) |
| Low-middle SDI | Morocco | 3.15 (2.47,3.79) | 3.59 (2.64,4.39) | -0.02 (-0.46,0.43) |
| Low-middle SDI | Myanmar | 3.14 (2.13,4.57) | 2.56 (1.86,3.69) | -1.13 (-1.45,-0.81) |
| Low-middle SDI | Namibia | 3.3 (2.68,4.36) | 4.68 (3.49,6.32) | 1.11 (-0.5,2.75) |
| Low-middle SDI | Nauru | 3.05 (2.31,4.05) | 3.56 (2.38,4.84) | - |
| Low-middle SDI | Nicaragua | 1.61 (1.43,1.95) | 1.81 (1.48,2.25) | 0.18 (-1.18,1.56) |
| Low-middle SDI | Nigeria | 2.73 (2.05,3.41) | 2.93 (2.1,3.7) | 0.23 (-0.04,0.5) |
| Low-middle SDI | Pakistan | 4.75 (4.07,5.88) | 5.78 (4.51,7.64) | 0.31 (0.16,0.46) |
| Low-middle SDI | Palestine | 3.14 (2.23,4.09) | 2.84 (2.32,3.41) | -0.36 (-1.89,1.2) |
| Low-middle SDI | Samoa | 5.01 (4.14,6.24) | 4.73 (3.73,6.25) | 0.48 (-5.73,7.11) |
| Low-middle SDI | Sao Tome and Principe | 2.05 (1.69,2.53) | 2.58 (1.97,3.26) | - |
| Low-middle SDI | Sudan | 2.36 (1.39,4.31) | 2.26 (1.59,3.43) | 0.01 (-0.52,0.55) |
| Low-middle SDI | Syrian Arab Republic | 2.55 (1.86,3.2) | 1.94 (1.43,2.68) | -1.59 (-2.29,-0.88) |
| Low-middle SDI | Tajikistan | 2.66 (2.04,3.45) | 1.84 (1.41,2.35) | -1.7 (-2.62,-0.76) |
| Low-middle SDI | Tonga | 4.52 (3.66,5.68) | 5.05 (3.77,6.59) | -0.32 (-7.47,7.38) |
| Low-middle SDI | Tuvalu | 2.08 (1.66,2.7) | 2.34 (1.89,2.97) | - |
| Low-middle SDI | Venezuela (Bolivarian Republic of) | 3.45 (3.29,3.6) | 3.51 (2.71,4.48) | -0.14 (-0.57,0.29) |
| Low-middle SDI | Viet Nam | 3.31 (2.67,4.15) | 3.37 (2.56,4.67) | 0.31 (0.05,0.57) |
| Low-middle SDI | Zambia | 6.46 (5.07,7.83) | 6.93 (4.59,11.43) | 0.3 (-0.24,0.84) |
| Middle SDI | Albania | 1.10 (0.9,1.37) | 1.00 (0.76,1.28) | 0.22 (-1.88,2.36) |
| Middle SDI | Algeria | 2.96 (2.47,3.58) | 2.93 (2.31,3.63) | -0.44 (-0.89,0.01) |
| Middle SDI | Armenia | 1.25 (1.06,1.45) | 1.55 (1.28,1.83) | 1.66 (-0.04,3.39) |
| Middle SDI | Azerbaijan | 1.3 (0.98,1.67) | 0.97 (0.7,1.28) | -1.12 (-2.16,-0.06) |
| Middle SDI | Bosnia and Herzegovina | 1.75 (1.47,2.22) | 2.18 (1.61,2.72) | 0.35 (-1.24,1.97) |
| Middle SDI | Botswana | 3.37 (2.31,4.67) | 3.3 (2.51,4.47) | -0.46 (-2.09,1.21) |
| Middle SDI | Brazil | 3.07 (2.93,3.16) | 2.79 (2.61,2.91) | -0.85 (-1.01,-0.69) |
| Middle SDI | China | 2.67 (2.31,3.24) | 2.13 (1.68,2.57) | -0.78 (-0.91,-0.65) |
| Middle SDI | Colombia | 3.68 (3.5,3.83) | 3.31 (2.79,3.98) | -0.58 (-0.91,-0.25) |
| Middle SDI | Costa Rica | 4.3 (4.04,4.58) | 4.64 (4.1,5.2) | -0.18 (-1.18,0.83) |
| Middle SDI | Cuba | 4.49 (4.26,4.69) | 3.43 (2.99,3.9) | -0.14 (-1.25,0.97) |
| Middle SDI | Ecuador | 3.05 (2.86,3.22) | 4.55 (3.62,5.68) | 0.92 (0.34,1.5) |
| Middle SDI | Equatorial Guinea | 3.19 (2.28,4.44) | 3.41 (2.19,5.05) | 1.82 (-2.2,6.02) |
| Middle SDI | Fiji | 1.51 (1.24,2.05) | 2.13 (1.55,2.75) | 0.39 (-3.43,4.35) |
| Middle SDI | Gabon | 3.9 (3.05,5.15) | 4.2 (3.12,5.77) | -0.33 (-2.08,1.46) |
| Middle SDI | Grenada | 10.42 (9.5,11.32) | 10 (8.7,11.41) | -0.78 (-5.85,4.57) |
| Middle SDI | Guyana | 2.39 (2.11,2.67) | 2.69 (2.07,3.38) | 0.08 (-2.63,2.86) |
| Middle SDI | Indonesia | 2.23 (1.7,3.07) | 2.43 (1.8,3.53) | 0.03 (-0.14,0.19) |
| Middle SDI | Iran (Islamic Republic of) | 1.53 (1.28,1.9) | 1.73 (1.42,1.93) | 0.73 (0.36,1.1) |
| Middle SDI | Iraq | 3.6 (2.69,4.59) | 3.45 (2.52,4.37) | -0.9 (-1.33,-0.48) |
| Middle SDI | Jamaica | 4.25 (3.79,4.74) | 4.97 (3.86,6.35) | -0.27 (-1.33,0.79) |
| Middle SDI | Maldives | 2.49 (1.82,3.28) | 1.38 (1.12,1.67) | - |
| Middle SDI | Mauritius | 1.44 (1.37,1.51) | 2.5 (2.33,2.64) | 1.62 (-1.13,4.44) |
| Middle SDI | Mexico | 2.52 (2.45,2.58) | 3.07 (2.72,3.42) | 0.17 (-0.06,0.41) |
| Middle SDI | Panama | 2.77 (2.59,2.93) | 3.25 (2.59,3.91) | 0.41 (-0.86,1.69) |
| Middle SDI | Paraguay | 2.09 (1.68,2.61) | 2.86 (2.15,3.71) | 0.7 (-0.46,1.86) |
| Middle SDI | Peru | 4.27 (3.75,5.03) | 4.76 (3.5,6.13) | -0.19 (-0.55,0.18) |
| Middle SDI | Philippines | 2.26 (2.01,2.65) | 2.52 (2.12,3.19) | 0.16 (-0.1,0.43) |
| Middle SDI | Saint Lucia | 7.21 (6.79,7.66) | 5.03 (4.17,6) | 0.59 (-4.66,6.12) |
| Middle SDI | Saint Vincent and the Grenadines | 7.96 (7.37,8.54) | 6.54 (5.78,7.45) | 1.09 (-4.5,7) |
| Middle SDI | South Africa | 2.92 (2.53,3.39) | 3.73 (2.99,4.11) | 0.77 (0.47,1.07) |
| Middle SDI | Sri Lanka | 2.3 (2.05,2.88) | 2.06 (1.39,2.82) | -0.86 (-1.43,-0.29) |
| Middle SDI | Suriname | 3.97 (3.44,4.43) | 3.31 (2.46,4.24) | -0.38 (-3.14,2.46) |
| Middle SDI | Thailand | 3.13 (2.63,4.25) | 2.87 (2.2,3.95) | -1.11 (-1.36,-0.85) |
| Middle SDI | Tokelau | 1.96 (1.48,2.56) | 2.64 (2.05,3.4) | - |
| Middle SDI | Tunisia | 4.2 (3.59,4.93) | 3.99 (2.85,5.43) | -0.3 (-0.96,0.36) |
| Middle SDI | Türkiye | 4.72 (3.9,5.8) | 3.51 (2.79,4.41) | -1.68 (-1.91,-1.46) |
| Middle SDI | Turkmenistan | 0.59 (0.52,0.69) | 0.75 (0.57,0.97) | 0.86 (-1.28,3.05) |
| Middle SDI | Uruguay | 5.27 (4.97,5.61) | 5.47 (5.02,5.9) | -0.33 (-1.29,0.64) |
| Middle SDI | Uzbekistan | 1.55 (1.27,1.87) | 1.32 (1.06,1.64) | -0.93 (-1.47,-0.39) |

**Table S3 The local drift of incidence and mortality from 1992 to 2021 for non-Hodgkin lymphoma for different age groups across 204 countries and territories**

| **Quintile** | **Country** | **Age** | **Local drift of incidence**  **(% per year)** | **Local drift of mortality**  **(% per year)** |
| --- | --- | --- | --- | --- |
| High SDI | Andorra | 5 to 9 | -0.43 (-21.38,26.11) | -0.62 (-21.74,26.19) |
| High SDI | Andorra | 10 to 14 | -0.08 (-16.73,19.91) | -0.8 (-17.94,19.92) |
| High SDI | Andorra | 15 to 19 | 0.15 (-13.9,16.5) | -0.13 (-16.06,18.82) |
| High SDI | Andorra | 20 to 24 | 0.71 (-10.48,13.3) | 0.7 (-14.89,19.15) |
| High SDI | Andorra | 25 to 29 | 1.09 (-5.69,8.35) | 1.19 (-14.27,19.45) |
| High SDI | Andorra | 30 to 34 | 0.77 (-4.74,6.6) | 0.67 (-14.32,18.28) |
| High SDI | Andorra | 35 to 39 | 0.04 (-4.68,4.99) | -0.42 (-15.14,16.84) |
| High SDI | Andorra | 40 to 44 | 0.34 (-3.68,4.54) | -1.42 (-15.46,14.96) |
| High SDI | Andorra | 45 to 49 | 0.19 (-3.53,4.04) | -1.97 (-14.68,12.63) |
| High SDI | Andorra | 50 to 54 | 0.18 (-3.44,3.93) | -2.09 (-12.9,10.06) |
| High SDI | Andorra | 55 to 59 | 0.32 (-3.25,4.03) | -2.15 (-10.05,6.45) |
| High SDI | Andorra | 60 to 64 | 0.81 (-2.63,4.37) | -2.04 (-8.29,4.64) |
| High SDI | Andorra | 65 to 69 | 0.65 (-2.71,4.13) | -1.65 (-7.17,4.2) |
| High SDI | Andorra | 70 to 74 | 0.4 (-3.02,3.93) | -1.57 (-6.81,3.98) |
| High SDI | Andorra | 75 to 79 | 0.95 (-3.38,5.48) | -2.12 (-7.44,3.51) |
| High SDI | Andorra | 80 to 84 | 0.64 (-4.54,6.1) | -2.66 (-8.42,3.45) |
| High SDI | Andorra | 85 to 89 | -0.78 (-7.48,6.4) | 2.6 (-14.76,23.5) |
| High SDI | Australia | 5 to 9 | -2.44 (-4.02,-0.84) | -5.77 (-11.54,0.38) |
| High SDI | Australia | 10 to 14 | -1.36 (-2.46,-0.24) | -3.86 (-7.27,-0.33) |
| High SDI | Australia | 15 to 19 | -0.84 (-1.73,0.06) | -3.42 (-6.05,-0.72) |
| High SDI | Australia | 20 to 24 | -0.61 (-1.35,0.15) | -3.29 (-5.41,-1.12) |
| High SDI | Australia | 25 to 29 | -0.22 (-0.84,0.4) | -3.06 (-4.82,-1.27) |
| High SDI | Australia | 30 to 34 | 0.01 (-0.51,0.54) | -2.88 (-4.35,-1.39) |
| High SDI | Australia | 35 to 39 | -0.17 (-0.62,0.29) | -2.89 (-4.11,-1.65) |
| High SDI | Australia | 40 to 44 | -0.42 (-0.81,-0.02) | -3.1 (-4.13,-2.07) |
| High SDI | Australia | 45 to 49 | -0.58 (-0.92,-0.25) | -3.16 (-3.99,-2.32) |
| High SDI | Australia | 50 to 54 | -0.69 (-0.99,-0.39) | -3.2 (-3.89,-2.51) |
| High SDI | Australia | 55 to 59 | -0.59 (-0.86,-0.33) | -3.06 (-3.62,-2.5) |
| High SDI | Australia | 60 to 64 | -0.47 (-0.71,-0.22) | -2.9 (-3.37,-2.43) |
| High SDI | Australia | 65 to 69 | -0.21 (-0.43,0.02) | -2.59 (-3,-2.18) |
| High SDI | Australia | 70 to 74 | 0.21 (-0.02,0.44) | -2.04 (-2.41,-1.68) |
| High SDI | Australia | 75 to 79 | 0.56 (0.31,0.82) | -1.4 (-1.76,-1.04) |
| High SDI | Australia | 80 to 84 | 0.83 (0.51,1.16) | -0.7 (-1.11,-0.28) |
| High SDI | Australia | 85 to 89 | 1.2 (0.66,1.75) | 0.17 (-0.49,0.84) |
| High SDI | Austria | 5 to 9 | -0.33 (-2.69,2.08) | -0.04 (-7.21,7.69) |
| High SDI | Austria | 10 to 14 | -0.4 (-2.14,1.38) | -0.4 (-5.87,5.4) |
| High SDI | Austria | 15 to 19 | -0.32 (-1.67,1.05) | -1.14 (-5.56,3.48) |
| High SDI | Austria | 20 to 24 | -0.09 (-1.19,1.03) | -1.15 (-4.56,2.38) |
| High SDI | Austria | 25 to 29 | 0.41 (-0.49,1.32) | -1.54 (-4.4,1.41) |
| High SDI | Austria | 30 to 34 | 0.44 (-0.35,1.23) | -1.73 (-4.17,0.77) |
| High SDI | Austria | 35 to 39 | 0.06 (-0.66,0.79) | -2.32 (-4.47,-0.13) |
| High SDI | Austria | 40 to 44 | -0.1 (-0.75,0.56) | -2.41 (-4.18,-0.61) |
| High SDI | Austria | 45 to 49 | -0.08 (-0.66,0.5) | -2.39 (-3.8,-0.96) |
| High SDI | Austria | 50 to 54 | 0.15 (-0.36,0.66) | -1.96 (-3.08,-0.84) |
| High SDI | Austria | 55 to 59 | 0.76 (0.28,1.24) | -1.28 (-2.22,-0.34) |
| High SDI | Austria | 60 to 64 | 0.98 (0.53,1.43) | -0.97 (-1.77,-0.17) |
| High SDI | Austria | 65 to 69 | 1.19 (0.76,1.61) | -0.59 (-1.28,0.11) |
| High SDI | Austria | 70 to 74 | 1.47 (1.05,1.9) | -0.13 (-0.75,0.49) |
| High SDI | Austria | 75 to 79 | 1.62 (1.12,2.11) | 0.23 (-0.39,0.86) |
| High SDI | Austria | 80 to 84 | 1.78 (1.18,2.38) | 0.59 (-0.1,1.27) |
| High SDI | Austria | 85 to 89 | 2.31 (1.34,3.28) | 1.34 (0.3,2.39) |
| High SDI | Belgium | 5 to 9 | -0.1 (-1.83,1.67) | -2.82 (-9.34,4.16) |
| High SDI | Belgium | 10 to 14 | -0.57 (-1.93,0.81) | -3.02 (-7.81,2.02) |
| High SDI | Belgium | 15 to 19 | -0.6 (-1.75,0.56) | -2.84 (-6.54,1.01) |
| High SDI | Belgium | 20 to 24 | -0.27 (-1.23,0.71) | -2.81 (-5.93,0.42) |
| High SDI | Belgium | 25 to 29 | 0.11 (-0.7,0.92) | -2.16 (-4.72,0.47) |
| High SDI | Belgium | 30 to 34 | 0.3 (-0.4,1) | -1.86 (-4,0.32) |
| High SDI | Belgium | 35 to 39 | 0.29 (-0.33,0.92) | -1.94 (-3.71,-0.14) |
| High SDI | Belgium | 40 to 44 | -0.06 (-0.62,0.5) | -2.21 (-3.67,-0.72) |
| High SDI | Belgium | 45 to 49 | -0.44 (-0.93,0.06) | -2.5 (-3.7,-1.28) |
| High SDI | Belgium | 50 to 54 | -0.57 (-1.01,-0.13) | -2.57 (-3.56,-1.58) |
| High SDI | Belgium | 55 to 59 | -0.34 (-0.74,0.05) | -2.28 (-3.09,-1.46) |
| High SDI | Belgium | 60 to 64 | -0.08 (-0.43,0.28) | -1.91 (-2.58,-1.24) |
| High SDI | Belgium | 65 to 69 | 0.18 (-0.14,0.51) | -1.56 (-2.12,-0.99) |
| High SDI | Belgium | 70 to 74 | 0.49 (0.17,0.81) | -1.08 (-1.58,-0.58) |
| High SDI | Belgium | 75 to 79 | 0.7 (0.33,1.08) | -0.68 (-1.19,-0.17) |
| High SDI | Belgium | 80 to 84 | 0.91 (0.47,1.35) | -0.26 (-0.81,0.29) |
| High SDI | Belgium | 85 to 89 | 1.18 (0.5,1.86) | 0.23 (-0.58,1.04) |
| High SDI | Bermuda | 5 to 9 | 1 (-20.44,28.21) | -0.64 (-21.89,26.39) |
| High SDI | Bermuda | 10 to 14 | 0.58 (-16.79,21.58) | 0.04 (-17.29,21.01) |
| High SDI | Bermuda | 15 to 19 | 1.34 (-14.8,20.54) | 0.83 (-15.26,19.98) |
| High SDI | Bermuda | 20 to 24 | 1.32 (-14.07,19.47) | 1.26 (-14.42,19.82) |
| High SDI | Bermuda | 25 to 29 | 0.24 (-14.64,17.71) | 1.48 (-14.05,19.82) |
| High SDI | Bermuda | 30 to 34 | 0.5 (-13.76,17.12) | 1.43 (-13.97,19.59) |
| High SDI | Bermuda | 35 to 39 | 0.06 (-12.74,14.75) | 1.35 (-14.02,19.47) |
| High SDI | Bermuda | 40 to 44 | -0.83 (-11.19,10.74) | 0.08 (-14.9,17.71) |
| High SDI | Bermuda | 45 to 49 | -0.64 (-7.37,6.58) | -0.37 (-15.22,17.08) |
| High SDI | Bermuda | 50 to 54 | -0.52 (-5.99,5.26) | -1.65 (-15.88,14.99) |
| High SDI | Bermuda | 55 to 59 | -1.01 (-5.99,4.25) | -2.86 (-16.21,12.61) |
| High SDI | Bermuda | 60 to 64 | 0.13 (-4.54,5.02) | 1.16 (-11.93,16.21) |
| High SDI | Bermuda | 65 to 69 | 0.4 (-4.25,5.28) | 0.96 (-8.58,11.5) |
| High SDI | Bermuda | 70 to 74 | 0.15 (-4.69,5.24) | -0.19 (-8.62,9.02) |
| High SDI | Bermuda | 75 to 79 | -0.47 (-5.81,5.18) | 1.88 (-9.85,15.15) |
| High SDI | Bermuda | 80 to 84 | 4.21 (-8.78,19.05) | 1.45 (-13.78,19.37) |
| High SDI | Bermuda | 85 to 89 | 6.5 (-12.77,30.04) | -0.65 (-21.72,26.1) |
| High SDI | Canada | 5 to 9 | -2.06 (-4.05,-0.02) | -4.25 (-8.73,0.44) |
| High SDI | Canada | 10 to 14 | -1.69 (-2.99,-0.37) | -4.37 (-7.59,-1.05) |
| High SDI | Canada | 15 to 19 | -1.73 (-2.64,-0.82) | -3.66 (-6.01,-1.25) |
| High SDI | Canada | 20 to 24 | -1.88 (-2.5,-1.26) | -2.86 (-4.58,-1.1) |
| High SDI | Canada | 25 to 29 | -2.17 (-2.65,-1.69) | -2.69 (-4.08,-1.27) |
| High SDI | Canada | 30 to 34 | -2.42 (-2.84,-2.01) | -2.71 (-3.87,-1.54) |
| High SDI | Canada | 35 to 39 | -2.62 (-3,-2.23) | -2.92 (-3.88,-1.94) |
| High SDI | Canada | 40 to 44 | -2.59 (-2.94,-2.24) | -3.17 (-3.98,-2.36) |
| High SDI | Canada | 45 to 49 | -2.31 (-2.61,-2) | -3.17 (-3.81,-2.52) |
| High SDI | Canada | 50 to 54 | -2 (-2.27,-1.73) | -3.08 (-3.59,-2.57) |
| High SDI | Canada | 55 to 59 | -1.66 (-1.9,-1.42) | -2.91 (-3.32,-2.5) |
| High SDI | Canada | 60 to 64 | -1.19 (-1.42,-0.97) | -2.61 (-2.95,-2.26) |
| High SDI | Canada | 65 to 69 | -0.66 (-0.88,-0.45) | -2.11 (-2.41,-1.8) |
| High SDI | Canada | 70 to 74 | -0.05 (-0.27,0.18) | -1.4 (-1.67,-1.12) |
| High SDI | Canada | 75 to 79 | 0.31 (0.05,0.57) | -0.72 (-1.01,-0.44) |
| High SDI | Canada | 80 to 84 | 0.54 (0.21,0.87) | -0.09 (-0.42,0.25) |
| High SDI | Canada | 85 to 89 | 0.79 (0.24,1.34) | 0.49 (-0.05,1.04) |
| High SDI | Cyprus | 5 to 9 | 1.31 (-2.68,5.47) | 0.66 (-20.53,27.5) |
| High SDI | Cyprus | 10 to 14 | 1.66 (-1.71,5.14) | -0.29 (-17.14,19.98) |
| High SDI | Cyprus | 15 to 19 | 1.45 (-1.45,4.42) | -1.71 (-16.41,15.57) |
| High SDI | Cyprus | 20 to 24 | 1.88 (-0.56,4.37) | -3.47 (-16.04,10.98) |
| High SDI | Cyprus | 25 to 29 | 1.42 (-0.76,3.65) | -4.45 (-14.34,6.58) |
| High SDI | Cyprus | 30 to 34 | 1.85 (-0.13,3.87) | -2.95 (-9.24,3.77) |
| High SDI | Cyprus | 35 to 39 | 2.51 (0.64,4.42) | -1.95 (-7.04,3.41) |
| High SDI | Cyprus | 40 to 44 | 2.88 (1.1,4.7) | -1.17 (-5.74,3.61) |
| High SDI | Cyprus | 45 to 49 | 2.78 (1.1,4.48) | -0.75 (-4.66,3.32) |
| High SDI | Cyprus | 50 to 54 | 2.75 (1.2,4.31) | -0.72 (-4.04,2.72) |
| High SDI | Cyprus | 55 to 59 | 2.43 (1.04,3.85) | -0.89 (-3.66,1.95) |
| High SDI | Cyprus | 60 to 64 | 2.16 (0.88,3.44) | -1.03 (-3.35,1.35) |
| High SDI | Cyprus | 65 to 69 | 1.78 (0.57,3) | -1.59 (-3.63,0.49) |
| High SDI | Cyprus | 70 to 74 | 1.8 (0.59,3.03) | -1.35 (-3.2,0.54) |
| High SDI | Cyprus | 75 to 79 | 1.66 (0.32,3.01) | -1.17 (-3,0.69) |
| High SDI | Cyprus | 80 to 84 | 0.61 (-1.08,2.33) | -1.89 (-3.91,0.18) |
| High SDI | Cyprus | 85 to 89 | -0.92 (-4.35,2.63) | -2.41 (-6.61,1.97) |
| High SDI | Czechia | 5 to 9 | 1.13 (-1.23,3.55) | -2.05 (-8.53,4.89) |
| High SDI | Czechia | 10 to 14 | 1.83 (0.11,3.58) | -0.72 (-5.05,3.82) |
| High SDI | Czechia | 15 to 19 | 2.66 (1.24,4.1) | -0.43 (-4.02,3.29) |
| High SDI | Czechia | 20 to 24 | 3.4 (2.21,4.6) | 0.83 (-2.02,3.76) |
| High SDI | Czechia | 25 to 29 | 3.03 (2.04,4.03) | 0.71 (-1.68,3.17) |
| High SDI | Czechia | 30 to 34 | 2.59 (1.72,3.46) | 0.1 (-2.02,2.28) |
| High SDI | Czechia | 35 to 39 | 1.61 (0.84,2.38) | -0.65 (-2.45,1.17) |
| High SDI | Czechia | 40 to 44 | 0.84 (0.18,1.51) | -1.41 (-2.88,0.09) |
| High SDI | Czechia | 45 to 49 | 0.46 (-0.13,1.05) | -1.76 (-3.01,-0.5) |
| High SDI | Czechia | 50 to 54 | 0.67 (0.14,1.2) | -1.42 (-2.46,-0.37) |
| High SDI | Czechia | 55 to 59 | 0.86 (0.38,1.34) | -1.18 (-2.05,-0.31) |
| High SDI | Czechia | 60 to 64 | 1.19 (0.77,1.61) | -0.82 (-1.52,-0.12) |
| High SDI | Czechia | 65 to 69 | 1.29 (0.9,1.68) | -0.65 (-1.24,-0.05) |
| High SDI | Czechia | 70 to 74 | 1.44 (1.04,1.84) | -0.37 (-0.93,0.19) |
| High SDI | Czechia | 75 to 79 | 1.5 (0.99,2.01) | -0.15 (-0.8,0.5) |
| High SDI | Czechia | 80 to 84 | 1.74 (1.11,2.37) | 0.27 (-0.49,1.03) |
| High SDI | Czechia | 85 to 89 | 2.5 (1.43,3.59) | 1.09 (-0.17,2.37) |
| High SDI | Denmark | 5 to 9 | 0.19 (-3.08,3.57) | -10.03 (-26.18,9.67) |
| High SDI | Denmark | 10 to 14 | 0.23 (-2.28,2.82) | -7.52 (-19.14,5.77) |
| High SDI | Denmark | 15 to 19 | 1.22 (-0.89,3.38) | -2.37 (-8.65,4.33) |
| High SDI | Denmark | 20 to 24 | 1.55 (-0.27,3.4) | -1.59 (-6.41,3.46) |
| High SDI | Denmark | 25 to 29 | 1.72 (0.19,3.27) | -2.11 (-6.43,2.42) |
| High SDI | Denmark | 30 to 34 | 1.35 (0.03,2.68) | -2.5 (-6.21,1.35) |
| High SDI | Denmark | 35 to 39 | 0.49 (-0.63,1.61) | -2.86 (-5.8,0.18) |
| High SDI | Denmark | 40 to 44 | -0.19 (-1.12,0.74) | -3.26 (-5.59,-0.88) |
| High SDI | Denmark | 45 to 49 | -0.05 (-0.8,0.7) | -3.3 (-5.13,-1.45) |
| High SDI | Denmark | 50 to 54 | 0.23 (-0.4,0.87) | -2.84 (-4.25,-1.42) |
| High SDI | Denmark | 55 to 59 | 0.7 (0.14,1.25) | -2.28 (-3.42,-1.12) |
| High SDI | Denmark | 60 to 64 | 1.05 (0.55,1.55) | -1.79 (-2.74,-0.84) |
| High SDI | Denmark | 65 to 69 | 1.44 (0.97,1.91) | -1.26 (-2.07,-0.45) |
| High SDI | Denmark | 70 to 74 | 1.81 (1.35,2.27) | -0.78 (-1.5,-0.06) |
| High SDI | Denmark | 75 to 79 | 2.26 (1.73,2.78) | -0.16 (-0.89,0.57) |
| High SDI | Denmark | 80 to 84 | 2.65 (1.97,3.32) | 0.56 (-0.29,1.42) |
| High SDI | Denmark | 85 to 89 | 2.89 (1.82,3.97) | 1.04 (-0.26,2.36) |
| High SDI | Estonia | 5 to 9 | 3.52 (1.67,5.4) | -0.72 (-21.52,25.6) |
| High SDI | Estonia | 10 to 14 | 3.04 (1.26,4.86) | -1.57 (-17.65,17.65) |
| High SDI | Estonia | 15 to 19 | 3.18 (1.13,5.28) | -3.14 (-15.86,11.5) |
| High SDI | Estonia | 20 to 24 | 3.2 (0.94,5.5) | -2.88 (-12.89,8.29) |
| High SDI | Estonia | 25 to 29 | 3.23 (0.98,5.54) | -1.21 (-7.42,5.41) |
| High SDI | Estonia | 30 to 34 | 2.92 (0.83,5.06) | -0.8 (-5.7,4.35) |
| High SDI | Estonia | 35 to 39 | 2.42 (0.52,4.36) | -0.92 (-5.27,3.63) |
| High SDI | Estonia | 40 to 44 | 1.84 (0.14,3.56) | -0.79 (-4.53,3.1) |
| High SDI | Estonia | 45 to 49 | 1.89 (0.38,3.43) | -1.39 (-4.66,1.99) |
| High SDI | Estonia | 50 to 54 | 2.55 (1.18,3.95) | -0.45 (-3.12,2.3) |
| High SDI | Estonia | 55 to 59 | 3.13 (1.88,4.39) | 0.24 (-1.91,2.44) |
| High SDI | Estonia | 60 to 64 | 3.36 (2.2,4.53) | 0.52 (-1.33,2.41) |
| High SDI | Estonia | 65 to 69 | 3.48 (2.33,4.64) | 0.95 (-0.77,2.69) |
| High SDI | Estonia | 70 to 74 | 3.65 (2.36,4.95) | 1.3 (-0.45,3.09) |
| High SDI | Estonia | 75 to 79 | 3.76 (2.1,5.46) | 1.8 (-0.26,3.89) |
| High SDI | Estonia | 80 to 84 | 4.21 (2.02,6.45) | 2.58 (0.06,5.17) |
| High SDI | Estonia | 85 to 89 | 4 (0.27,7.88) | 3.16 (-1.36,7.89) |
| High SDI | Finland | 5 to 9 | 0.45 (-2.42,3.41) | -6.22 (-21.97,12.71) |
| High SDI | Finland | 10 to 14 | 1.6 (-0.44,3.68) | -0.26 (-5.81,5.61) |
| High SDI | Finland | 15 to 19 | 2.16 (0.46,3.88) | -0.18 (-4.88,4.75) |
| High SDI | Finland | 20 to 24 | 2.05 (0.58,3.54) | -0.39 (-4.31,3.69) |
| High SDI | Finland | 25 to 29 | 1.73 (0.49,2.99) | -0.96 (-4.31,2.51) |
| High SDI | Finland | 30 to 34 | 1.45 (0.39,2.52) | -0.99 (-3.79,1.88) |
| High SDI | Finland | 35 to 39 | 0.93 (0.02,1.84) | -1.64 (-4.01,0.78) |
| High SDI | Finland | 40 to 44 | 0.43 (-0.37,1.23) | -2.48 (-4.48,-0.44) |
| High SDI | Finland | 45 to 49 | 0.26 (-0.43,0.96) | -2.53 (-4.17,-0.86) |
| High SDI | Finland | 50 to 54 | 0.4 (-0.2,1) | -2.28 (-3.57,-0.97) |
| High SDI | Finland | 55 to 59 | 0.44 (-0.09,0.98) | -2.13 (-3.18,-1.06) |
| High SDI | Finland | 60 to 64 | 0.61 (0.12,1.1) | -1.84 (-2.7,-0.96) |
| High SDI | Finland | 65 to 69 | 0.66 (0.21,1.12) | -1.56 (-2.29,-0.83) |
| High SDI | Finland | 70 to 74 | 0.7 (0.24,1.16) | -1.27 (-1.93,-0.61) |
| High SDI | Finland | 75 to 79 | 0.6 (0.08,1.12) | -0.98 (-1.65,-0.31) |
| High SDI | Finland | 80 to 84 | 0.41 (-0.23,1.05) | -0.75 (-1.49,-0.01) |
| High SDI | Finland | 85 to 89 | 0.59 (-0.43,1.63) | -0.15 (-1.3,1.02) |
| High SDI | France | 5 to 9 | -0.25 (-0.98,0.48) | -3.19 (-6.09,-0.19) |
| High SDI | France | 10 to 14 | -0.14 (-0.69,0.42) | -3 (-5.03,-0.94) |
| High SDI | France | 15 to 19 | 0.34 (-0.12,0.82) | -2.67 (-4.25,-1.06) |
| High SDI | France | 20 to 24 | 0.69 (0.29,1.09) | -2.3 (-3.56,-1.02) |
| High SDI | France | 25 to 29 | 0.75 (0.42,1.09) | -2.26 (-3.31,-1.2) |
| High SDI | France | 30 to 34 | 0.71 (0.42,1) | -2.31 (-3.17,-1.44) |
| High SDI | France | 35 to 39 | 0.39 (0.13,0.64) | -2.62 (-3.35,-1.89) |
| High SDI | France | 40 to 44 | 0.07 (-0.16,0.29) | -2.85 (-3.45,-2.24) |
| High SDI | France | 45 to 49 | 0.04 (-0.16,0.24) | -2.79 (-3.28,-2.31) |
| High SDI | France | 50 to 54 | 0.15 (-0.03,0.33) | -2.57 (-2.96,-2.16) |
| High SDI | France | 55 to 59 | 0.36 (0.2,0.52) | -2.24 (-2.57,-1.91) |
| High SDI | France | 60 to 64 | 0.54 (0.39,0.68) | -1.91 (-2.18,-1.64) |
| High SDI | France | 65 to 69 | 0.63 (0.5,0.77) | -1.66 (-1.89,-1.42) |
| High SDI | France | 70 to 74 | 0.75 (0.61,0.88) | -1.36 (-1.57,-1.16) |
| High SDI | France | 75 to 79 | 0.8 (0.65,0.96) | -1.07 (-1.29,-0.86) |
| High SDI | France | 80 to 84 | 1.2 (1.02,1.38) | -0.44 (-0.66,-0.22) |
| High SDI | France | 85 to 89 | 1.29 (1.04,1.55) | -0.16 (-0.46,0.14) |
| High SDI | Germany | 5 to 9 | -1.19 (-2.03,-0.35) | -2.79 (-5.74,0.25) |
| High SDI | Germany | 10 to 14 | -1.01 (-1.65,-0.36) | -2.58 (-4.74,-0.37) |
| High SDI | Germany | 15 to 19 | -1.03 (-1.56,-0.49) | -2.38 (-4,-0.74) |
| High SDI | Germany | 20 to 24 | -1.1 (-1.55,-0.64) | -2.44 (-3.73,-1.14) |
| High SDI | Germany | 25 to 29 | -0.97 (-1.35,-0.6) | -2.65 (-3.71,-1.58) |
| High SDI | Germany | 30 to 34 | -0.84 (-1.15,-0.52) | -2.68 (-3.55,-1.8) |
| High SDI | Germany | 35 to 39 | -0.87 (-1.15,-0.59) | -2.76 (-3.49,-2.03) |
| High SDI | Germany | 40 to 44 | -1.02 (-1.26,-0.78) | -2.88 (-3.48,-2.27) |
| High SDI | Germany | 45 to 49 | -0.99 (-1.18,-0.79) | -2.79 (-3.26,-2.32) |
| High SDI | Germany | 50 to 54 | -1.03 (-1.19,-0.87) | -2.79 (-3.14,-2.43) |
| High SDI | Germany | 55 to 59 | -0.56 (-0.7,-0.42) | -2.29 (-2.58,-2.01) |
| High SDI | Germany | 60 to 64 | -0.08 (-0.21,0.04) | -1.78 (-2.02,-1.54) |
| High SDI | Germany | 65 to 69 | 0.37 (0.25,0.49) | -1.26 (-1.47,-1.05) |
| High SDI | Germany | 70 to 74 | 0.91 (0.79,1.03) | -0.64 (-0.82,-0.45) |
| High SDI | Germany | 75 to 79 | 1.31 (1.17,1.44) | -0.06 (-0.24,0.13) |
| High SDI | Germany | 80 to 84 | 1.34 (1.18,1.5) | 0.21 (0.01,0.41) |
| High SDI | Germany | 85 to 89 | 1.83 (1.58,2.09) | 1.08 (0.76,1.41) |
| High SDI | Greenland | 5 to 9 | 1.8 (-19.95,29.47) | - |
| High SDI | Greenland | 10 to 14 | 0.81 (-16.65,21.93) | - |
| High SDI | Greenland | 15 to 19 | -0.44 (-16.33,18.46) | - |
| High SDI | Greenland | 20 to 24 | -0.67 (-16.05,17.53) | - |
| High SDI | Greenland | 25 to 29 | 0.78 (-14.63,18.98) | - |
| High SDI | Greenland | 30 to 34 | 1.62 (-13.52,19.42) | - |
| High SDI | Greenland | 35 to 39 | 1.92 (-13.15,19.61) | - |
| High SDI | Greenland | 40 to 44 | 1.29 (-13.16,18.15) | - |
| High SDI | Greenland | 45 to 49 | 0.18 (-13.25,15.68) | - |
| High SDI | Greenland | 50 to 54 | -0.65 (-12.74,13.12) | - |
| High SDI | Greenland | 55 to 59 | 5.09 (-6.79,18.49) | - |
| High SDI | Greenland | 60 to 64 | 8.47 (-3.63,22.09) | - |
| High SDI | Greenland | 65 to 69 | 9.01 (-3.91,23.68) | - |
| High SDI | Greenland | 70 to 74 | 6 (-7.84,21.92) | - |
| High SDI | Greenland | 75 to 79 | 1.42 (-13.67,19.15) | - |
| High SDI | Greenland | 80 to 84 | -3.96 (-20.5,16.02) | - |
| High SDI | Greenland | 85 to 89 | -3.39 (-24.09,22.97) | - |
| High SDI | Iceland | 5 to 9 | -0.6 (-7.1,6.36) | -0.49 (-21.6,26.3) |
| High SDI | Iceland | 10 to 14 | 1.6 (-2.32,5.68) | -0.28 (-17.5,20.54) |
| High SDI | Iceland | 15 to 19 | 2.51 (-0.92,6.07) | -0.38 (-16.27,18.52) |
| High SDI | Iceland | 20 to 24 | 3.63 (0.42,6.94) | -0.68 (-16.04,17.49) |
| High SDI | Iceland | 25 to 29 | 4.68 (1.66,7.79) | -0.84 (-15.72,16.67) |
| High SDI | Iceland | 30 to 34 | 4.73 (1.75,7.78) | -0.48 (-15.2,16.78) |
| High SDI | Iceland | 35 to 39 | 4.17 (1.26,7.17) | -0.3 (-14.49,16.25) |
| High SDI | Iceland | 40 to 44 | 2.87 (0.01,5.82) | -0.69 (-13.51,14.03) |
| High SDI | Iceland | 45 to 49 | 1.8 (-1,4.68) | -1.33 (-12.04,10.68) |
| High SDI | Iceland | 50 to 54 | 1.32 (-1.4,4.11) | -1.68 (-9.31,6.58) |
| High SDI | Iceland | 55 to 59 | 1.49 (-1.12,4.17) | -1.83 (-7.87,4.61) |
| High SDI | Iceland | 60 to 64 | 1.47 (-1,4) | -1.64 (-6.71,3.69) |
| High SDI | Iceland | 65 to 69 | 1.82 (-0.6,4.31) | -0.67 (-5.05,3.9) |
| High SDI | Iceland | 70 to 74 | 1.94 (-0.52,4.47) | -0.24 (-4.14,3.81) |
| High SDI | Iceland | 75 to 79 | 1.74 (-1.07,4.63) | -0.05 (-3.92,3.98) |
| High SDI | Iceland | 80 to 84 | 1.62 (-1.95,5.32) | 1.44 (-3.51,6.64) |
| High SDI | Iceland | 85 to 89 | 2.74 (-3.52,9.41) | 1.7 (-5.08,8.97) |
| High SDI | Ireland | 5 to 9 | -0.13 (-2.46,2.26) | -8.39 (-23.99,10.41) |
| High SDI | Ireland | 10 to 14 | -0.31 (-2.11,1.53) | -2.41 (-9.09,4.75) |
| High SDI | Ireland | 15 to 19 | 0.25 (-1.29,1.82) | -1.6 (-6.31,3.34) |
| High SDI | Ireland | 20 to 24 | 1.28 (-0.08,2.65) | -1.45 (-5.6,2.89) |
| High SDI | Ireland | 25 to 29 | 2.03 (0.87,3.19) | -1.15 (-4.65,2.49) |
| High SDI | Ireland | 30 to 34 | 2.11 (1.09,3.15) | -1.53 (-4.62,1.67) |
| High SDI | Ireland | 35 to 39 | 1.99 (1.05,2.94) | -1.73 (-4.38,1) |
| High SDI | Ireland | 40 to 44 | 1.71 (0.84,2.58) | -1.94 (-4.19,0.37) |
| High SDI | Ireland | 45 to 49 | 1.41 (0.62,2.21) | -2.05 (-3.96,-0.11) |
| High SDI | Ireland | 50 to 54 | 1.19 (0.47,1.91) | -2.07 (-3.65,-0.46) |
| High SDI | Ireland | 55 to 59 | 0.96 (0.3,1.62) | -2.13 (-3.45,-0.79) |
| High SDI | Ireland | 60 to 64 | 0.86 (0.26,1.47) | -2.07 (-3.19,-0.94) |
| High SDI | Ireland | 65 to 69 | 0.95 (0.37,1.53) | -1.75 (-2.72,-0.77) |
| High SDI | Ireland | 70 to 74 | 1.27 (0.68,1.85) | -1.25 (-2.13,-0.35) |
| High SDI | Ireland | 75 to 79 | 1.66 (0.99,2.34) | -0.54 (-1.44,0.37) |
| High SDI | Ireland | 80 to 84 | 2.08 (1.19,2.97) | 0.25 (-0.82,1.33) |
| High SDI | Ireland | 85 to 89 | 2.54 (1.01,4.09) | 0.79 (-0.94,2.55) |
| High SDI | Japan | 5 to 9 | -0.16 (-1.45,1.14) | -2.61 (-5.02,-0.13) |
| High SDI | Japan | 10 to 14 | 0.05 (-0.91,1.02) | -2.47 (-4.06,-0.84) |
| High SDI | Japan | 15 to 19 | 0.02 (-0.8,0.86) | -2.71 (-3.95,-1.45) |
| High SDI | Japan | 20 to 24 | 0.2 (-0.52,0.92) | -2.55 (-3.58,-1.5) |
| High SDI | Japan | 25 to 29 | 0.3 (-0.31,0.92) | -2.34 (-3.23,-1.44) |
| High SDI | Japan | 30 to 34 | 0.07 (-0.44,0.58) | -2.42 (-3.17,-1.67) |
| High SDI | Japan | 35 to 39 | -0.42 (-0.84,0) | -2.75 (-3.37,-2.12) |
| High SDI | Japan | 40 to 44 | -0.73 (-1.06,-0.39) | -2.94 (-3.43,-2.45) |
| High SDI | Japan | 45 to 49 | -0.48 (-0.75,-0.2) | -2.66 (-3.05,-2.26) |
| High SDI | Japan | 50 to 54 | 0.18 (-0.06,0.41) | -1.97 (-2.29,-1.65) |
| High SDI | Japan | 55 to 59 | 0.84 (0.64,1.03) | -1.3 (-1.56,-1.05) |
| High SDI | Japan | 60 to 64 | 1.12 (0.95,1.29) | -0.93 (-1.13,-0.72) |
| High SDI | Japan | 65 to 69 | 1.32 (1.17,1.47) | -0.64 (-0.8,-0.47) |
| High SDI | Japan | 70 to 74 | 1.55 (1.4,1.7) | -0.29 (-0.44,-0.14) |
| High SDI | Japan | 75 to 79 | 1.84 (1.67,2) | 0.32 (0.17,0.47) |
| High SDI | Japan | 80 to 84 | 2.14 (1.93,2.34) | 1.06 (0.89,1.24) |
| High SDI | Japan | 85 to 89 | 2.31 (1.96,2.65) | 1.77 (1.48,2.05) |
| High SDI | Kuwait | 5 to 9 | 2.4 (-0.61,5.51) | -2.36 (-9.43,5.26) |
| High SDI | Kuwait | 10 to 14 | 1.98 (-0.56,4.6) | -2.01 (-7.54,3.85) |
| High SDI | Kuwait | 15 to 19 | 1.66 (-0.63,4) | -1.58 (-6.29,3.36) |
| High SDI | Kuwait | 20 to 24 | 1.32 (-0.65,3.33) | -1.22 (-5.21,2.94) |
| High SDI | Kuwait | 25 to 29 | 1.01 (-0.66,2.71) | -1.33 (-4.73,2.18) |
| High SDI | Kuwait | 30 to 34 | 0.9 (-0.6,2.42) | -1.69 (-4.71,1.42) |
| High SDI | Kuwait | 35 to 39 | 0.8 (-0.67,2.3) | -1.86 (-4.7,1.06) |
| High SDI | Kuwait | 40 to 44 | 0.59 (-0.93,2.14) | -1.93 (-4.67,0.88) |
| High SDI | Kuwait | 45 to 49 | 0.14 (-1.44,1.74) | -2.27 (-4.96,0.48) |
| High SDI | Kuwait | 50 to 54 | -0.06 (-1.75,1.66) | -2.5 (-5.18,0.25) |
| High SDI | Kuwait | 55 to 59 | -0.05 (-1.81,1.73) | -2.17 (-4.87,0.6) |
| High SDI | Kuwait | 60 to 64 | 0.18 (-1.61,2.01) | -1.63 (-4.28,1.09) |
| High SDI | Kuwait | 65 to 69 | 0.69 (-1.27,2.7) | -0.63 (-3.39,2.2) |
| High SDI | Kuwait | 70 to 74 | 1.12 (-1.24,3.54) | -0.25 (-3.2,2.79) |
| High SDI | Kuwait | 75 to 79 | 1.1 (-2.03,4.32) | 0.16 (-3.53,3.99) |
| High SDI | Kuwait | 80 to 84 | 1.19 (-3.36,5.95) | -0.06 (-4.8,4.9) |
| High SDI | Kuwait | 85 to 89 | -0.57 (-6.88,6.17) | -1.27 (-7.63,5.52) |
| High SDI | Latvia | 5 to 9 | 2.65 (-4.75,10.62) | -3.55 (-21.58,18.62) |
| High SDI | Latvia | 10 to 14 | 3.11 (-2.67,9.23) | 0.34 (-13.07,15.82) |
| High SDI | Latvia | 15 to 19 | 3.51 (-1.44,8.7) | 2.74 (-4.26,10.26) |
| High SDI | Latvia | 20 to 24 | 3.37 (-0.79,7.71) | 2.54 (-3.21,8.64) |
| High SDI | Latvia | 25 to 29 | 2.52 (-1.02,6.18) | 1.72 (-3.34,7.03) |
| High SDI | Latvia | 30 to 34 | 2.88 (-0.03,5.87) | 1.06 (-3.43,5.75) |
| High SDI | Latvia | 35 to 39 | 3.07 (0.57,5.62) | 1.43 (-2.35,5.35) |
| High SDI | Latvia | 40 to 44 | 2.77 (0.64,4.95) | 1.36 (-1.77,4.58) |
| High SDI | Latvia | 45 to 49 | 2.65 (0.86,4.46) | 1.29 (-1.32,3.97) |
| High SDI | Latvia | 50 to 54 | 3.08 (1.58,4.61) | 1.65 (-0.5,3.85) |
| High SDI | Latvia | 55 to 59 | 3.32 (2.03,4.64) | 1.93 (0.12,3.77) |
| High SDI | Latvia | 60 to 64 | 3.56 (2.36,4.76) | 2.28 (0.68,3.91) |
| High SDI | Latvia | 65 to 69 | 3.79 (2.62,4.97) | 2.64 (1.13,4.17) |
| High SDI | Latvia | 70 to 74 | 3.78 (2.49,5.09) | 2.7 (1.14,4.29) |
| High SDI | Latvia | 75 to 79 | 3.68 (2.04,5.35) | 2.83 (0.95,4.75) |
| High SDI | Latvia | 80 to 84 | 3.86 (1.73,6.04) | 3.04 (0.66,5.48) |
| High SDI | Latvia | 85 to 89 | 3.42 (0.08,6.87) | 2.66 (-1.19,6.65) |
| High SDI | Lithuania | 5 to 9 | 1.44 (-3.49,6.63) | -6.47 (-23.36,14.15) |
| High SDI | Lithuania | 10 to 14 | 1.92 (-1.81,5.79) | -3.2 (-15.27,10.59) |
| High SDI | Lithuania | 15 to 19 | 2.22 (-0.75,5.28) | 2.02 (-3.67,8.05) |
| High SDI | Lithuania | 20 to 24 | 1.79 (-0.72,4.37) | 1.29 (-3.68,6.51) |
| High SDI | Lithuania | 25 to 29 | 1.92 (-0.17,4.05) | 1.08 (-3.22,5.57) |
| High SDI | Lithuania | 30 to 34 | 2.49 (0.68,4.33) | 1.45 (-2.16,5.2) |
| High SDI | Lithuania | 35 to 39 | 2.61 (0.95,4.3) | 1.28 (-1.92,4.59) |
| High SDI | Lithuania | 40 to 44 | 2.52 (1,4.06) | 1.58 (-1.05,4.27) |
| High SDI | Lithuania | 45 to 49 | 2.43 (1.09,3.79) | 1.44 (-0.77,3.69) |
| High SDI | Lithuania | 50 to 54 | 2.49 (1.3,3.69) | 1.51 (-0.33,3.39) |
| High SDI | Lithuania | 55 to 59 | 2.62 (1.57,3.69) | 1.66 (0.1,3.24) |
| High SDI | Lithuania | 60 to 64 | 2.67 (1.68,3.66) | 1.75 (0.35,3.17) |
| High SDI | Lithuania | 65 to 69 | 2.87 (1.9,3.86) | 1.96 (0.64,3.29) |
| High SDI | Lithuania | 70 to 74 | 2.93 (1.86,4.01) | 2.13 (0.78,3.49) |
| High SDI | Lithuania | 75 to 79 | 3.23 (1.82,4.67) | 2.46 (0.83,4.12) |
| High SDI | Lithuania | 80 to 84 | 3.56 (1.69,5.45) | 2.83 (0.79,4.92) |
| High SDI | Lithuania | 85 to 89 | 3.59 (0.64,6.64) | 3.07 (-0.23,6.47) |
| High SDI | Luxembourg | 5 to 9 | -1.47 (-9.64,7.44) | -1.37 (-22.27,25.14) |
| High SDI | Luxembourg | 10 to 14 | -2.45 (-9.56,5.22) | -1.28 (-18.32,19.31) |
| High SDI | Luxembourg | 15 to 19 | -2.02 (-7.45,3.73) | -0.55 (-16.37,18.26) |
| High SDI | Luxembourg | 20 to 24 | -1.87 (-6.43,2.91) | -2.65 (-17.34,14.65) |
| High SDI | Luxembourg | 25 to 29 | -1.57 (-5.26,2.27) | -4.53 (-18.47,11.8) |
| High SDI | Luxembourg | 30 to 34 | -1.13 (-4.25,2.09) | -5.53 (-18.36,9.31) |
| High SDI | Luxembourg | 35 to 39 | -0.77 (-3.52,2.06) | -6.14 (-17.13,6.3) |
| High SDI | Luxembourg | 40 to 44 | -0.5 (-3.01,2.07) | -5.11 (-13.85,4.51) |
| High SDI | Luxembourg | 45 to 49 | 0.12 (-2.1,2.39) | -2.37 (-7.43,2.97) |
| High SDI | Luxembourg | 50 to 54 | 0.47 (-1.54,2.52) | -1.81 (-5.96,2.53) |
| High SDI | Luxembourg | 55 to 59 | 0.68 (-1.17,2.57) | -1.67 (-5.28,2.08) |
| High SDI | Luxembourg | 60 to 64 | 0.88 (-0.81,2.61) | -1.52 (-4.61,1.67) |
| High SDI | Luxembourg | 65 to 69 | 0.84 (-0.79,2.5) | -1.5 (-4.23,1.32) |
| High SDI | Luxembourg | 70 to 74 | 0.98 (-0.71,2.69) | -1.05 (-3.6,1.57) |
| High SDI | Luxembourg | 75 to 79 | 0.92 (-0.98,2.87) | -0.82 (-3.41,1.84) |
| High SDI | Luxembourg | 80 to 84 | 0.94 (-1.37,3.3) | -0.4 (-3.31,2.58) |
| High SDI | Luxembourg | 85 to 89 | 1.43 (-2.31,5.31) | -0.52 (-4.56,3.7) |
| High SDI | Monaco | 5 to 9 | -0.49 (-21.61,26.31) | -0.89 (-22,25.95) |
| High SDI | Monaco | 10 to 14 | -1.15 (-18.22,19.49) | -1.19 (-18.28,19.47) |
| High SDI | Monaco | 15 to 19 | -0.75 (-16.54,18.04) | -1.39 (-17.12,17.32) |
| High SDI | Monaco | 20 to 24 | -1.37 (-16.3,16.22) | -0.75 (-16.11,17.44) |
| High SDI | Monaco | 25 to 29 | -0.68 (-15.42,16.62) | 0.3 (-15.04,18.42) |
| High SDI | Monaco | 30 to 34 | 0.4 (-13.83,16.98) | 0.72 (-14.58,18.76) |
| High SDI | Monaco | 35 to 39 | 0.81 (-12.13,15.66) | 0.46 (-14.79,18.43) |
| High SDI | Monaco | 40 to 44 | 0.63 (-10.21,12.78) | -0.29 (-15.4,17.53) |
| High SDI | Monaco | 45 to 49 | 1.5 (-6.09,9.7) | -0.76 (-15.52,16.59) |
| High SDI | Monaco | 50 to 54 | 0.34 (-5.84,6.93) | -1.31 (-15.89,15.8) |
| High SDI | Monaco | 55 to 59 | -0.35 (-5.78,5.39) | -1.35 (-15.39,15.03) |
| High SDI | Monaco | 60 to 64 | 0.2 (-4.75,5.4) | -1.46 (-14.17,13.14) |
| High SDI | Monaco | 65 to 69 | 0.88 (-3.8,5.79) | -1.23 (-11.83,10.63) |
| High SDI | Monaco | 70 to 74 | 1.84 (-2.95,6.87) | -0.9 (-8.03,6.79) |
| High SDI | Monaco | 75 to 79 | 1.48 (-3.6,6.83) | 0.7 (-7.08,9.13) |
| High SDI | Monaco | 80 to 84 | 0.78 (-5,6.92) | 1.42 (-6.33,9.81) |
| High SDI | Monaco | 85 to 89 | -0.45 (-7.86,7.57) | 6.66 (-11.63,28.74) |
| High SDI | Netherlands | 5 to 9 | -0.17 (-1.98,1.67) | -2.97 (-9.07,3.55) |
| High SDI | Netherlands | 10 to 14 | 0.53 (-0.76,1.83) | -1.75 (-5.53,2.17) |
| High SDI | Netherlands | 15 to 19 | 0.75 (-0.33,1.85) | -2.06 (-5.27,1.25) |
| High SDI | Netherlands | 20 to 24 | 0.97 (0.05,1.91) | -1.83 (-4.52,0.94) |
| High SDI | Netherlands | 25 to 29 | 0.95 (0.18,1.73) | -1.98 (-4.31,0.41) |
| High SDI | Netherlands | 30 to 34 | 0.79 (0.15,1.44) | -1.95 (-3.92,0.06) |
| High SDI | Netherlands | 35 to 39 | 0.43 (-0.12,0.97) | -2.24 (-3.87,-0.58) |
| High SDI | Netherlands | 40 to 44 | 0.12 (-0.34,0.57) | -2.51 (-3.83,-1.17) |
| High SDI | Netherlands | 45 to 49 | -0.07 (-0.44,0.29) | -2.61 (-3.64,-1.57) |
| High SDI | Netherlands | 50 to 54 | -0.09 (-0.4,0.23) | -2.58 (-3.4,-1.75) |
| High SDI | Netherlands | 55 to 59 | 0.22 (-0.06,0.49) | -2.31 (-3,-1.63) |
| High SDI | Netherlands | 60 to 64 | 0.57 (0.32,0.82) | -1.91 (-2.48,-1.33) |
| High SDI | Netherlands | 65 to 69 | 0.92 (0.69,1.16) | -1.43 (-1.93,-0.93) |
| High SDI | Netherlands | 70 to 74 | 1.25 (1.01,1.48) | -0.93 (-1.38,-0.48) |
| High SDI | Netherlands | 75 to 79 | 1.35 (1.09,1.61) | -0.58 (-1.04,-0.12) |
| High SDI | Netherlands | 80 to 84 | 1.12 (0.79,1.45) | -0.52 (-1.04,0.01) |
| High SDI | Netherlands | 85 to 89 | 1 (0.48,1.52) | -0.35 (-1.14,0.45) |
| High SDI | New Zealand | 5 to 9 | -2.44 (-4.93,0.11) | -11.09 (-27.04,8.35) |
| High SDI | New Zealand | 10 to 14 | -1.4 (-3.14,0.36) | -8.85 (-20.23,4.14) |
| High SDI | New Zealand | 15 to 19 | -0.58 (-2.06,0.91) | -4.5 (-10.2,1.57) |
| High SDI | New Zealand | 20 to 24 | 0.51 (-0.77,1.8) | -2.94 (-7,1.29) |
| High SDI | New Zealand | 25 to 29 | 0.93 (-0.2,2.07) | -2.58 (-5.92,0.88) |
| High SDI | New Zealand | 30 to 34 | 1.16 (0.13,2.2) | -2.1 (-4.99,0.87) |
| High SDI | New Zealand | 35 to 39 | 0.9 (-0.04,1.85) | -2.2 (-4.77,0.44) |
| High SDI | New Zealand | 40 to 44 | 0.74 (-0.1,1.59) | -1.98 (-4.11,0.2) |
| High SDI | New Zealand | 45 to 49 | 0.41 (-0.32,1.15) | -2.06 (-3.79,-0.31) |
| High SDI | New Zealand | 50 to 54 | -0.02 (-0.66,0.62) | -2.29 (-3.69,-0.86) |
| High SDI | New Zealand | 55 to 59 | -0.33 (-0.89,0.24) | -2.51 (-3.67,-1.34) |
| High SDI | New Zealand | 60 to 64 | -0.43 (-0.94,0.08) | -2.44 (-3.43,-1.45) |
| High SDI | New Zealand | 65 to 69 | -0.26 (-0.74,0.22) | -2.19 (-3.06,-1.32) |
| High SDI | New Zealand | 70 to 74 | 0.1 (-0.37,0.57) | -1.7 (-2.49,-0.9) |
| High SDI | New Zealand | 75 to 79 | 0.54 (0.01,1.06) | -1.1 (-1.9,-0.29) |
| High SDI | New Zealand | 80 to 84 | 0.97 (0.32,1.63) | -0.52 (-1.45,0.43) |
| High SDI | New Zealand | 85 to 89 | 1.24 (0.2,2.3) | 0 (-1.47,1.48) |
| High SDI | Norway | 5 to 9 | -1.74 (-4.71,1.33) | -10.65 (-26.68,8.89) |
| High SDI | Norway | 10 to 14 | -0.94 (-3,1.17) | -7.84 (-19.34,5.3) |
| High SDI | Norway | 15 to 19 | -0.43 (-2.08,1.25) | -2.32 (-7.75,3.43) |
| High SDI | Norway | 20 to 24 | -0.05 (-1.48,1.41) | -2.46 (-6.9,2.2) |
| High SDI | Norway | 25 to 29 | 0.54 (-0.69,1.79) | -2.47 (-6.4,1.62) |
| High SDI | Norway | 30 to 34 | 0.74 (-0.35,1.84) | -1.91 (-5.14,1.43) |
| High SDI | Norway | 35 to 39 | 0.75 (-0.22,1.73) | -2.47 (-5.27,0.41) |
| High SDI | Norway | 40 to 44 | 0.37 (-0.49,1.23) | -2.88 (-5.14,-0.56) |
| High SDI | Norway | 45 to 49 | 0.05 (-0.69,0.81) | -3.14 (-4.97,-1.28) |
| High SDI | Norway | 50 to 54 | -0.02 (-0.7,0.65) | -2.98 (-4.5,-1.44) |
| High SDI | Norway | 55 to 59 | 0.13 (-0.49,0.75) | -2.73 (-4,-1.44) |
| High SDI | Norway | 60 to 64 | 0.32 (-0.24,0.88) | -2.3 (-3.35,-1.25) |
| High SDI | Norway | 65 to 69 | 0.66 (0.14,1.19) | -1.74 (-2.62,-0.85) |
| High SDI | Norway | 70 to 74 | 1.02 (0.51,1.53) | -1.14 (-1.91,-0.37) |
| High SDI | Norway | 75 to 79 | 1.16 (0.6,1.72) | -0.66 (-1.41,0.09) |
| High SDI | Norway | 80 to 84 | 1.11 (0.42,1.8) | -0.26 (-1.09,0.57) |
| High SDI | Norway | 85 to 89 | 1.23 (0.17,2.3) | 0.23 (-0.97,1.45) |
| High SDI | Poland | 5 to 9 | -0.1 (-1.53,1.35) | -3.16 (-6.34,0.13) |
| High SDI | Poland | 10 to 14 | 0.83 (-0.24,1.9) | -2.65 (-4.97,-0.28) |
| High SDI | Poland | 15 to 19 | 1.55 (0.66,2.45) | -1.49 (-3.22,0.28) |
| High SDI | Poland | 20 to 24 | 2 (1.23,2.78) | -0.83 (-2.22,0.58) |
| High SDI | Poland | 25 to 29 | 1.87 (1.2,2.55) | -0.65 (-1.81,0.53) |
| High SDI | Poland | 30 to 34 | 1.48 (0.9,2.07) | -0.83 (-1.83,0.18) |
| High SDI | Poland | 35 to 39 | 1.07 (0.56,1.59) | -1.23 (-2.08,-0.36) |
| High SDI | Poland | 40 to 44 | 0.86 (0.41,1.32) | -1.48 (-2.21,-0.74) |
| High SDI | Poland | 45 to 49 | 0.84 (0.44,1.25) | -1.43 (-2.07,-0.79) |
| High SDI | Poland | 50 to 54 | 1.02 (0.65,1.38) | -1.16 (-1.69,-0.62) |
| High SDI | Poland | 55 to 59 | 1.2 (0.89,1.51) | -0.81 (-1.25,-0.37) |
| High SDI | Poland | 60 to 64 | 1.38 (1.1,1.66) | -0.48 (-0.85,-0.11) |
| High SDI | Poland | 65 to 69 | 1.72 (1.45,2) | 0 (-0.34,0.35) |
| High SDI | Poland | 70 to 74 | 2.18 (1.87,2.49) | 0.64 (0.28,1.01) |
| High SDI | Poland | 75 to 79 | 2.62 (2.21,3.03) | 1.35 (0.9,1.79) |
| High SDI | Poland | 80 to 84 | 2.87 (2.34,3.41) | 1.83 (1.28,2.39) |
| High SDI | Poland | 85 to 89 | 3.19 (2.24,4.15) | 2.32 (1.37,3.29) |
| High SDI | Puerto Rico | 5 to 9 | -2 (-6.43,2.64) | -5.49 (-21.56,13.89) |
| High SDI | Puerto Rico | 10 to 14 | -0.94 (-3.81,2.01) | -1.14 (-7.68,5.87) |
| High SDI | Puerto Rico | 15 to 19 | -0.41 (-2.48,1.71) | -1.9 (-5.98,2.35) |
| High SDI | Puerto Rico | 20 to 24 | 0.42 (-1.17,2.02) | -1.78 (-4.74,1.28) |
| High SDI | Puerto Rico | 25 to 29 | 0.95 (-0.35,2.28) | -1.86 (-4.42,0.78) |
| High SDI | Puerto Rico | 30 to 34 | 1.11 (-0.05,2.27) | -1.34 (-3.55,0.92) |
| High SDI | Puerto Rico | 35 to 39 | 0.96 (-0.11,2.05) | -1.38 (-3.36,0.65) |
| High SDI | Puerto Rico | 40 to 44 | 0.65 (-0.36,1.67) | -1.54 (-3.32,0.28) |
| High SDI | Puerto Rico | 45 to 49 | 0.57 (-0.36,1.51) | -1.58 (-3.18,0.05) |
| High SDI | Puerto Rico | 50 to 54 | 0.51 (-0.33,1.36) | -1.5 (-2.92,-0.07) |
| High SDI | Puerto Rico | 55 to 59 | 0.49 (-0.29,1.27) | -1.44 (-2.7,-0.17) |
| High SDI | Puerto Rico | 60 to 64 | 0.39 (-0.34,1.12) | -1.39 (-2.52,-0.24) |
| High SDI | Puerto Rico | 65 to 69 | 0.23 (-0.48,0.95) | -1.5 (-2.56,-0.44) |
| High SDI | Puerto Rico | 70 to 74 | 0.16 (-0.56,0.89) | -1.45 (-2.46,-0.42) |
| High SDI | Puerto Rico | 75 to 79 | 0.41 (-0.42,1.25) | -1.11 (-2.2,-0.02) |
| High SDI | Puerto Rico | 80 to 84 | 0.63 (-0.47,1.73) | -0.89 (-2.19,0.43) |
| High SDI | Puerto Rico | 85 to 89 | 1.07 (-0.7,2.87) | -0.42 (-2.46,1.66) |
| High SDI | Qatar | 5 to 9 | 2.1 (-5.25,10.02) | -4.57 (-24.54,20.69) |
| High SDI | Qatar | 10 to 14 | 2.9 (-3.04,9.21) | -3.36 (-18.84,15.07) |
| High SDI | Qatar | 15 to 19 | 3.01 (-1.87,8.14) | -2.84 (-15,11.07) |
| High SDI | Qatar | 20 to 24 | 3.12 (-0.94,7.35) | -1.7 (-9.9,7.24) |
| High SDI | Qatar | 25 to 29 | 2.99 (-0.39,6.49) | -2.25 (-8.62,4.56) |
| High SDI | Qatar | 30 to 34 | 2.85 (-0.21,6.01) | -2.07 (-7.47,3.64) |
| High SDI | Qatar | 35 to 39 | 2.47 (-0.41,5.43) | -2.03 (-6.73,2.92) |
| High SDI | Qatar | 40 to 44 | 2.07 (-0.86,5.09) | -2.19 (-6.52,2.34) |
| High SDI | Qatar | 45 to 49 | 1.83 (-1.31,5.07) | -3.06 (-7.36,1.44) |
| High SDI | Qatar | 50 to 54 | 1.22 (-2.15,4.72) | -2.55 (-7.33,2.48) |
| High SDI | Qatar | 55 to 59 | 0.66 (-2.92,4.38) | -2.74 (-7.98,2.79) |
| High SDI | Qatar | 60 to 64 | 0.74 (-3.21,4.85) | -2.73 (-8.47,3.37) |
| High SDI | Qatar | 65 to 69 | 0.26 (-4.76,5.54) | -1.47 (-8.26,5.84) |
| High SDI | Qatar | 70 to 74 | 3.67 (-6.23,14.61) | 1.96 (-8.35,13.43) |
| High SDI | Qatar | 75 to 79 | 4.45 (-8.16,18.78) | 2.61 (-9.99,16.97) |
| High SDI | Qatar | 80 to 84 | 2.17 (-12.98,19.97) | 1.71 (-13.4,19.47) |
| High SDI | Qatar | 85 to 89 | -2.04 (-22.12,23.23) | -2.29 (-22.4,23.03) |
| High SDI | Republic of Korea | 5 to 9 | -0.01 (-1.51,1.51) | -4.7 (-8.17,-1.1) |
| High SDI | Republic of Korea | 10 to 14 | 0.72 (-0.37,1.81) | -4.36 (-6.73,-1.93) |
| High SDI | Republic of Korea | 15 to 19 | 0.99 (0.09,1.9) | -3.98 (-5.69,-2.24) |
| High SDI | Republic of Korea | 20 to 24 | 1.23 (0.47,2) | -3.67 (-5.04,-2.29) |
| High SDI | Republic of Korea | 25 to 29 | 1.38 (0.72,2.05) | -3.45 (-4.65,-2.24) |
| High SDI | Republic of Korea | 30 to 34 | 1.53 (0.97,2.1) | -3.06 (-4.09,-2.02) |
| High SDI | Republic of Korea | 35 to 39 | 1.69 (1.22,2.16) | -2.73 (-3.59,-1.87) |
| High SDI | Republic of Korea | 40 to 44 | 1.71 (1.29,2.13) | -2.53 (-3.26,-1.78) |
| High SDI | Republic of Korea | 45 to 49 | 1.76 (1.39,2.13) | -2.35 (-2.97,-1.72) |
| High SDI | Republic of Korea | 50 to 54 | 1.55 (1.21,1.88) | -2.38 (-2.92,-1.84) |
| High SDI | Republic of Korea | 55 to 59 | 1.61 (1.3,1.91) | -2.17 (-2.64,-1.71) |
| High SDI | Republic of Korea | 60 to 64 | 1.84 (1.54,2.13) | -1.77 (-2.2,-1.34) |
| High SDI | Republic of Korea | 65 to 69 | 2.32 (2.01,2.63) | -1.05 (-1.47,-0.63) |
| High SDI | Republic of Korea | 70 to 74 | 2.93 (2.58,3.28) | -0.13 (-0.56,0.3) |
| High SDI | Republic of Korea | 75 to 79 | 3.59 (3.14,4.04) | 0.93 (0.43,1.44) |
| High SDI | Republic of Korea | 80 to 84 | 4.13 (3.42,4.85) | 1.89 (1.16,2.63) |
| High SDI | Republic of Korea | 85 to 89 | 4.84 (3.41,6.29) | 2.83 (1.41,4.28) |
| High SDI | San Marino | 5 to 9 | -0.63 (-21.72,26.13) | -0.86 (-21.97,25.96) |
| High SDI | San Marino | 10 to 14 | -0.33 (-17.52,20.46) | -0.99 (-18.11,19.7) |
| High SDI | San Marino | 15 to 19 | -0.32 (-15.95,18.21) | -0.29 (-16.2,18.63) |
| High SDI | San Marino | 20 to 24 | -0.37 (-15.35,17.25) | 0.72 (-14.87,19.17) |
| High SDI | San Marino | 25 to 29 | -0.05 (-14.29,16.57) | 1.35 (-14.16,19.65) |
| High SDI | San Marino | 30 to 34 | 0.23 (-12.63,14.98) | 1 (-14.34,19.08) |
| High SDI | San Marino | 35 to 39 | -0.44 (-11.21,11.64) | -0.26 (-15.39,17.59) |
| High SDI | San Marino | 40 to 44 | -0.23 (-8.13,8.36) | -1.53 (-16.46,16.06) |
| High SDI | San Marino | 45 to 49 | -0.06 (-6.68,7.03) | -2.26 (-16.8,14.82) |
| High SDI | San Marino | 50 to 54 | -0.33 (-6.02,5.71) | -2.55 (-16.94,14.35) |
| High SDI | San Marino | 55 to 59 | -0.18 (-5.27,5.18) | -2.33 (-16.24,13.88) |
| High SDI | San Marino | 60 to 64 | 0.12 (-4.5,4.95) | -1.98 (-14.62,12.53) |
| High SDI | San Marino | 65 to 69 | 0.19 (-4.17,4.76) | -1.83 (-12.31,9.89) |
| High SDI | San Marino | 70 to 74 | 0.4 (-4.23,5.25) | -1.86 (-8.79,5.61) |
| High SDI | San Marino | 75 to 79 | 0.34 (-4.61,5.55) | -1.95 (-7.81,4.29) |
| High SDI | San Marino | 80 to 84 | -0.61 (-6.22,5.34) | -2.28 (-8.23,4.06) |
| High SDI | San Marino | 85 to 89 | -2.38 (-9.58,5.41) | 3.45 (-14.15,24.66) |
| High SDI | Saudi Arabia | 5 to 9 | 0.91 (-0.65,2.49) | -3.17 (-6.29,0.07) |
| High SDI | Saudi Arabia | 10 to 14 | 2.5 (1.35,3.67) | -1.35 (-3.44,0.79) |
| High SDI | Saudi Arabia | 15 to 19 | 3.9 (2.96,4.84) | 0.09 (-1.47,1.68) |
| High SDI | Saudi Arabia | 20 to 24 | 4.84 (4.03,5.65) | 1.35 (0.09,2.62) |
| High SDI | Saudi Arabia | 25 to 29 | 5.22 (4.49,5.96) | 1.8 (0.69,2.93) |
| High SDI | Saudi Arabia | 30 to 34 | 5.06 (4.37,5.75) | 1.81 (0.78,2.84) |
| High SDI | Saudi Arabia | 35 to 39 | 4.62 (3.96,5.29) | 1.61 (0.66,2.57) |
| High SDI | Saudi Arabia | 40 to 44 | 4.29 (3.63,4.95) | 1.49 (0.58,2.41) |
| High SDI | Saudi Arabia | 45 to 49 | 3.99 (3.32,4.65) | 1.4 (0.52,2.28) |
| High SDI | Saudi Arabia | 50 to 54 | 3.7 (3.03,4.38) | 1.36 (0.49,2.23) |
| High SDI | Saudi Arabia | 55 to 59 | 3.49 (2.79,4.19) | 1.37 (0.51,2.23) |
| High SDI | Saudi Arabia | 60 to 64 | 3.38 (2.64,4.12) | 1.43 (0.56,2.3) |
| High SDI | Saudi Arabia | 65 to 69 | 3.34 (2.54,4.15) | 1.65 (0.75,2.56) |
| High SDI | Saudi Arabia | 70 to 74 | 3.39 (2.42,4.37) | 2.02 (0.99,3.06) |
| High SDI | Saudi Arabia | 75 to 79 | 3.36 (2.09,4.64) | 2.15 (0.88,3.43) |
| High SDI | Saudi Arabia | 80 to 84 | 2.98 (1.26,4.72) | 2.01 (0.35,3.69) |
| High SDI | Saudi Arabia | 85 to 89 | 2.75 (-0.15,5.72) | 1.7 (-0.98,4.45) |
| High SDI | Singapore | 5 to 9 | 1.59 (-2.74,6.1) | -6.49 (-22.31,12.55) |
| High SDI | Singapore | 10 to 14 | 2.89 (-0.33,6.22) | -0.3 (-6.97,6.84) |
| High SDI | Singapore | 15 to 19 | 3.51 (0.79,6.3) | -0.3 (-5.28,4.94) |
| High SDI | Singapore | 20 to 24 | 2.39 (0.02,4.81) | -1.58 (-6.02,3.07) |
| High SDI | Singapore | 25 to 29 | 1.27 (-0.73,3.3) | -1.88 (-5.46,1.84) |
| High SDI | Singapore | 30 to 34 | 0.91 (-0.78,2.64) | -2.58 (-5.73,0.69) |
| High SDI | Singapore | 35 to 39 | 0.24 (-1.23,1.73) | -3.4 (-6.13,-0.59) |
| High SDI | Singapore | 40 to 44 | 0.19 (-1.12,1.52) | -3.23 (-5.6,-0.8) |
| High SDI | Singapore | 45 to 49 | 0.53 (-0.63,1.7) | -2.84 (-4.87,-0.77) |
| High SDI | Singapore | 50 to 54 | 0.48 (-0.56,1.54) | -2.66 (-4.41,-0.88) |
| High SDI | Singapore | 55 to 59 | 0.6 (-0.35,1.57) | -2.31 (-3.8,-0.8) |
| High SDI | Singapore | 60 to 64 | 0.88 (-0.02,1.78) | -1.95 (-3.27,-0.62) |
| High SDI | Singapore | 65 to 69 | 1.1 (0.19,2.01) | -1.52 (-2.77,-0.26) |
| High SDI | Singapore | 70 to 74 | 1.4 (0.39,2.41) | -0.99 (-2.27,0.31) |
| High SDI | Singapore | 75 to 79 | 1.61 (0.38,2.86) | -0.47 (-1.9,0.98) |
| High SDI | Singapore | 80 to 84 | 1.71 (0,3.46) | 0.01 (-1.87,1.93) |
| High SDI | Singapore | 85 to 89 | 1.9 (-1.21,5.1) | 0.19 (-2.96,3.45) |
| High SDI | Slovakia | 5 to 9 | 0.24 (-2.99,3.57) | -1.24 (-7.91,5.92) |
| High SDI | Slovakia | 10 to 14 | 0.96 (-1.64,3.63) | -1.18 (-6.31,4.23) |
| High SDI | Slovakia | 15 to 19 | 1.79 (-0.43,4.07) | -1.22 (-5.66,3.43) |
| High SDI | Slovakia | 20 to 24 | 2.11 (0.22,4.02) | -1.19 (-5.11,2.9) |
| High SDI | Slovakia | 25 to 29 | 1.62 (0.02,3.24) | -0.55 (-3.76,2.78) |
| High SDI | Slovakia | 30 to 34 | 1.22 (-0.14,2.61) | -0.79 (-3.57,2.07) |
| High SDI | Slovakia | 35 to 39 | 0.77 (-0.42,1.97) | -1.26 (-3.66,1.2) |
| High SDI | Slovakia | 40 to 44 | 0.68 (-0.35,1.73) | -1.23 (-3.23,0.81) |
| High SDI | Slovakia | 45 to 49 | 0.96 (0.05,1.89) | -1 (-2.69,0.73) |
| High SDI | Slovakia | 50 to 54 | 1.37 (0.57,2.19) | -0.62 (-2.04,0.83) |
| High SDI | Slovakia | 55 to 59 | 1.6 (0.87,2.33) | -0.37 (-1.57,0.84) |
| High SDI | Slovakia | 60 to 64 | 1.75 (1.09,2.41) | -0.16 (-1.19,0.88) |
| High SDI | Slovakia | 65 to 69 | 1.75 (1.12,2.39) | -0.01 (-0.96,0.94) |
| High SDI | Slovakia | 70 to 74 | 1.93 (1.24,2.62) | 0.31 (-0.64,1.28) |
| High SDI | Slovakia | 75 to 79 | 2.27 (1.33,3.21) | 0.91 (-0.27,2.1) |
| High SDI | Slovakia | 80 to 84 | 2.62 (1.41,3.84) | 1.34 (-0.09,2.79) |
| High SDI | Slovakia | 85 to 89 | 3.48 (1.33,5.68) | 2.5 (-0.13,5.19) |
| High SDI | Slovenia | 5 to 9 | 3.13 (-1.01,7.44) | -1.21 (-21.78,24.78) |
| High SDI | Slovenia | 10 to 14 | 3.23 (0.05,6.51) | -1.21 (-16.68,17.14) |
| High SDI | Slovenia | 15 to 19 | 3.72 (1.18,6.32) | -1.33 (-12.74,11.58) |
| High SDI | Slovenia | 20 to 24 | 3.48 (1.36,5.63) | 0.78 (-5.86,7.89) |
| High SDI | Slovenia | 25 to 29 | 3.65 (1.99,5.33) | 0.59 (-4.78,6.26) |
| High SDI | Slovenia | 30 to 34 | 3 (1.59,4.44) | 0 (-4.52,4.72) |
| High SDI | Slovenia | 35 to 39 | 2.36 (1.14,3.6) | -0.93 (-4.74,3.04) |
| High SDI | Slovenia | 40 to 44 | 1.94 (0.87,3.02) | -1.59 (-4.82,1.75) |
| High SDI | Slovenia | 45 to 49 | 1.87 (0.91,2.84) | -1.7 (-4.41,1.09) |
| High SDI | Slovenia | 50 to 54 | 2.4 (1.54,3.26) | -0.81 (-2.93,1.36) |
| High SDI | Slovenia | 55 to 59 | 2.78 (2,3.56) | -0.15 (-1.86,1.6) |
| High SDI | Slovenia | 60 to 64 | 2.88 (2.17,3.6) | 0.11 (-1.32,1.56) |
| High SDI | Slovenia | 65 to 69 | 3.03 (2.32,3.74) | 0.42 (-0.86,1.73) |
| High SDI | Slovenia | 70 to 74 | 3.17 (2.39,3.96) | 0.79 (-0.47,2.08) |
| High SDI | Slovenia | 75 to 79 | 3.48 (2.47,4.5) | 1.43 (0.01,2.87) |
| High SDI | Slovenia | 80 to 84 | 3.88 (2.65,5.13) | 2.26 (0.67,3.87) |
| High SDI | Slovenia | 85 to 89 | 4.83 (2.58,7.13) | 3.53 (0.67,6.47) |
| High SDI | Sweden | 5 to 9 | -1.38 (-3.91,1.23) | -7.26 (-22.83,11.45) |
| High SDI | Sweden | 10 to 14 | 0.5 (-1.25,2.29) | -0.99 (-6.46,4.81) |
| High SDI | Sweden | 15 to 19 | 1.24 (-0.32,2.83) | -0.81 (-5.41,4.02) |
| High SDI | Sweden | 20 to 24 | 1.76 (0.38,3.17) | -0.34 (-4.09,3.56) |
| High SDI | Sweden | 25 to 29 | 1.49 (0.32,2.67) | -1.14 (-4.39,2.23) |
| High SDI | Sweden | 30 to 34 | 1.04 (0.03,2.06) | -1.36 (-4.06,1.42) |
| High SDI | Sweden | 35 to 39 | 0.19 (-0.7,1.08) | -2.14 (-4.39,0.16) |
| High SDI | Sweden | 40 to 44 | -0.81 (-1.58,-0.04) | -3.12 (-4.96,-1.25) |
| High SDI | Sweden | 45 to 49 | -1.5 (-2.14,-0.85) | -3.68 (-5.13,-2.2) |
| High SDI | Sweden | 50 to 54 | -1.5 (-2.03,-0.96) | -3.55 (-4.67,-2.4) |
| High SDI | Sweden | 55 to 59 | -1.41 (-1.88,-0.94) | -3.43 (-4.34,-2.51) |
| High SDI | Sweden | 60 to 64 | -1.12 (-1.53,-0.71) | -3.04 (-3.77,-2.31) |
| High SDI | Sweden | 65 to 69 | -0.6 (-0.97,-0.24) | -2.37 (-2.96,-1.78) |
| High SDI | Sweden | 70 to 74 | -0.15 (-0.49,0.19) | -1.72 (-2.21,-1.22) |
| High SDI | Sweden | 75 to 79 | 0.33 (-0.04,0.7) | -0.98 (-1.45,-0.5) |
| High SDI | Sweden | 80 to 84 | 0.75 (0.29,1.2) | -0.3 (-0.83,0.23) |
| High SDI | Sweden | 85 to 89 | 1.02 (0.33,1.72) | 0.19 (-0.57,0.95) |
| High SDI | Switzerland | 5 to 9 | -1.28 (-2.99,0.46) | -0.43 (-7.6,7.29) |
| High SDI | Switzerland | 10 to 14 | -1.15 (-2.47,0.19) | -1.49 (-7,4.36) |
| High SDI | Switzerland | 15 to 19 | -0.61 (-1.7,0.49) | -2.76 (-7.36,2.07) |
| High SDI | Switzerland | 20 to 24 | -0.6 (-1.54,0.35) | -3.57 (-7.47,0.5) |
| High SDI | Switzerland | 25 to 29 | -0.5 (-1.31,0.31) | -3.37 (-6.46,-0.18) |
| High SDI | Switzerland | 30 to 34 | -0.38 (-1.09,0.34) | -3.55 (-6.23,-0.78) |
| High SDI | Switzerland | 35 to 39 | -0.53 (-1.19,0.14) | -3.27 (-5.49,-0.99) |
| High SDI | Switzerland | 40 to 44 | -0.82 (-1.44,-0.2) | -3.37 (-5.25,-1.44) |
| High SDI | Switzerland | 45 to 49 | -0.87 (-1.42,-0.3) | -3.26 (-4.78,-1.71) |
| High SDI | Switzerland | 50 to 54 | -0.8 (-1.3,-0.3) | -3.01 (-4.24,-1.77) |
| High SDI | Switzerland | 55 to 59 | -0.64 (-1.09,-0.18) | -2.78 (-3.79,-1.76) |
| High SDI | Switzerland | 60 to 64 | -0.37 (-0.79,0.05) | -2.42 (-3.27,-1.57) |
| High SDI | Switzerland | 65 to 69 | -0.07 (-0.47,0.33) | -2.03 (-2.75,-1.3) |
| High SDI | Switzerland | 70 to 74 | 0.1 (-0.29,0.49) | -1.58 (-2.22,-0.94) |
| High SDI | Switzerland | 75 to 79 | 0.34 (-0.09,0.77) | -0.9 (-1.52,-0.28) |
| High SDI | Switzerland | 80 to 84 | 0.71 (0.19,1.24) | -0.05 (-0.72,0.63) |
| High SDI | Switzerland | 85 to 89 | 0.85 (0.04,1.67) | 0.71 (-0.31,1.74) |
| High SDI | Taiwan (Province of China) | 5 to 9 | -0.18 (-2.4,2.09) | -1.99 (-6.39,2.61) |
| High SDI | Taiwan (Province of China) | 10 to 14 | 0.4 (-1.09,1.92) | -1.5 (-4.25,1.34) |
| High SDI | Taiwan (Province of China) | 15 to 19 | 0.31 (-0.84,1.46) | -1.92 (-3.91,0.11) |
| High SDI | Taiwan (Province of China) | 20 to 24 | 0.1 (-0.85,1.07) | -2.05 (-3.66,-0.41) |
| High SDI | Taiwan (Province of China) | 25 to 29 | 0.1 (-0.72,0.93) | -1.95 (-3.34,-0.54) |
| High SDI | Taiwan (Province of China) | 30 to 34 | 0.38 (-0.31,1.08) | -1.57 (-2.75,-0.38) |
| High SDI | Taiwan (Province of China) | 35 to 39 | 0.56 (-0.03,1.15) | -1.22 (-2.2,-0.23) |
| High SDI | Taiwan (Province of China) | 40 to 44 | 0.62 (0.09,1.15) | -0.98 (-1.82,-0.13) |
| High SDI | Taiwan (Province of China) | 45 to 49 | 0.64 (0.15,1.13) | -0.82 (-1.56,-0.06) |
| High SDI | Taiwan (Province of China) | 50 to 54 | 0.38 (-0.06,0.83) | -0.97 (-1.61,-0.32) |
| High SDI | Taiwan (Province of China) | 55 to 59 | 0.14 (-0.27,0.55) | -1.13 (-1.69,-0.56) |
| High SDI | Taiwan (Province of China) | 60 to 64 | -0.12 (-0.51,0.26) | -1.3 (-1.81,-0.79) |
| High SDI | Taiwan (Province of China) | 65 to 69 | -0.16 (-0.54,0.22) | -1.25 (-1.73,-0.77) |
| High SDI | Taiwan (Province of China) | 70 to 74 | 0.23 (-0.19,0.64) | -0.77 (-1.25,-0.28) |
| High SDI | Taiwan (Province of China) | 75 to 79 | 0.92 (0.42,1.42) | 0.06 (-0.48,0.61) |
| High SDI | Taiwan (Province of China) | 80 to 84 | 1.65 (0.92,2.39) | 0.89 (0.14,1.64) |
| High SDI | Taiwan (Province of China) | 85 to 89 | 2.66 (1.12,4.21) | 1.79 (0.34,3.27) |
| High SDI | United Arab Emirates | 5 to 9 | -1.22 (-6.05,3.87) | -2.31 (-9.26,5.17) |
| High SDI | United Arab Emirates | 10 to 14 | 1.32 (-1.97,4.71) | -0.86 (-6.05,4.62) |
| High SDI | United Arab Emirates | 15 to 19 | 2.39 (-0.09,4.93) | 0.33 (-3.61,4.44) |
| High SDI | United Arab Emirates | 20 to 24 | 3.3 (1.29,5.35) | 1.23 (-1.89,4.45) |
| High SDI | United Arab Emirates | 25 to 29 | 2.17 (0.5,3.87) | 0.21 (-2.35,2.83) |
| High SDI | United Arab Emirates | 30 to 34 | 0.37 (-1.07,1.83) | -1.66 (-3.78,0.5) |
| High SDI | United Arab Emirates | 35 to 39 | -0.81 (-2.16,0.56) | -2.68 (-4.6,-0.72) |
| High SDI | United Arab Emirates | 40 to 44 | -0.89 (-2.28,0.52) | -2.64 (-4.53,-0.71) |
| High SDI | United Arab Emirates | 45 to 49 | -1.64 (-3.08,-0.17) | -3.14 (-5.06,-1.18) |
| High SDI | United Arab Emirates | 50 to 54 | -2.05 (-3.64,-0.42) | -3.3 (-5.34,-1.21) |
| High SDI | United Arab Emirates | 55 to 59 | -1.6 (-3.39,0.23) | -2.46 (-4.67,-0.2) |
| High SDI | United Arab Emirates | 60 to 64 | -0.56 (-2.65,1.57) | -1.28 (-3.74,1.25) |
| High SDI | United Arab Emirates | 65 to 69 | 1.16 (-1.42,3.81) | -0.25 (-2.96,2.53) |
| High SDI | United Arab Emirates | 70 to 74 | 3.78 (-0.02,7.73) | 2.95 (-0.8,6.84) |
| High SDI | United Arab Emirates | 75 to 79 | 4.28 (-0.68,9.48) | 3.82 (-1.06,8.95) |
| High SDI | United Arab Emirates | 80 to 84 | 4.19 (-2.54,11.38) | 4.43 (-2.19,11.5) |
| High SDI | United Arab Emirates | 85 to 89 | 7.44 (-11.13,29.88) | 8.74 (-9.55,30.74) |
| High SDI | United Kingdom | 5 to 9 | -0.8 (-1.69,0.09) | -3.12 (-6.35,0.23) |
| High SDI | United Kingdom | 10 to 14 | -0.24 (-0.89,0.42) | -2.67 (-4.86,-0.44) |
| High SDI | United Kingdom | 15 to 19 | 0.13 (-0.42,0.69) | -2.47 (-4.1,-0.82) |
| High SDI | United Kingdom | 20 to 24 | 0.43 (-0.05,0.9) | -2.23 (-3.49,-0.96) |
| High SDI | United Kingdom | 25 to 29 | 0.65 (0.26,1.05) | -1.95 (-2.95,-0.95) |
| High SDI | United Kingdom | 30 to 34 | 0.72 (0.38,1.06) | -1.87 (-2.7,-1.04) |
| High SDI | United Kingdom | 35 to 39 | 0.53 (0.23,0.83) | -1.99 (-2.68,-1.3) |
| High SDI | United Kingdom | 40 to 44 | 0.14 (-0.13,0.4) | -2.31 (-2.88,-1.73) |
| High SDI | United Kingdom | 45 to 49 | -0.21 (-0.43,0.02) | -2.59 (-3.05,-2.12) |
| High SDI | United Kingdom | 50 to 54 | -0.54 (-0.74,-0.34) | -2.82 (-3.19,-2.45) |
| High SDI | United Kingdom | 55 to 59 | -0.68 (-0.86,-0.49) | -2.88 (-3.19,-2.57) |
| High SDI | United Kingdom | 60 to 64 | -0.46 (-0.62,-0.3) | -2.57 (-2.84,-2.31) |
| High SDI | United Kingdom | 65 to 69 | 0.04 (-0.11,0.2) | -1.96 (-2.18,-1.73) |
| High SDI | United Kingdom | 70 to 74 | 0.48 (0.33,0.63) | -1.35 (-1.55,-1.15) |
| High SDI | United Kingdom | 75 to 79 | 1.23 (1.07,1.4) | -0.33 (-0.53,-0.13) |
| High SDI | United Kingdom | 80 to 84 | 1.51 (1.31,1.72) | 0.27 (0.04,0.49) |
| High SDI | United Kingdom | 85 to 89 | 1.72 (1.39,2.05) | 0.78 (0.43,1.12) |
| High SDI | United States of America | 5 to 9 | -1.79 (-2.54,-1.03) | -3.47 (-5.11,-1.8) |
| High SDI | United States of America | 10 to 14 | -1.78 (-2.31,-1.24) | -3.48 (-4.54,-2.4) |
| High SDI | United States of America | 15 to 19 | -1.68 (-2.12,-1.25) | -3.41 (-4.18,-2.64) |
| High SDI | United States of America | 20 to 24 | -1.63 (-1.98,-1.27) | -3.31 (-3.89,-2.72) |
| High SDI | United States of America | 25 to 29 | -1.8 (-2.08,-1.51) | -3.42 (-3.88,-2.96) |
| High SDI | United States of America | 30 to 34 | -2.23 (-2.46,-2) | -3.74 (-4.11,-3.36) |
| High SDI | United States of America | 35 to 39 | -2.67 (-2.87,-2.46) | -4.03 (-4.34,-3.72) |
| High SDI | United States of America | 40 to 44 | -2.88 (-3.06,-2.7) | -4.13 (-4.38,-3.87) |
| High SDI | United States of America | 45 to 49 | -2.72 (-2.87,-2.58) | -3.9 (-4.11,-3.69) |
| High SDI | United States of America | 50 to 54 | -2.39 (-2.51,-2.26) | -3.54 (-3.7,-3.37) |
| High SDI | United States of America | 55 to 59 | -2.09 (-2.2,-1.98) | -3.22 (-3.36,-3.09) |
| High SDI | United States of America | 60 to 64 | -1.93 (-2.03,-1.84) | -3.04 (-3.15,-2.93) |
| High SDI | United States of America | 65 to 69 | -1.71 (-1.8,-1.62) | -2.77 (-2.87,-2.67) |
| High SDI | United States of America | 70 to 74 | -1.35 (-1.44,-1.25) | -2.33 (-2.42,-2.24) |
| High SDI | United States of America | 75 to 79 | -0.86 (-0.95,-0.76) | -1.71 (-1.8,-1.62) |
| High SDI | United States of America | 80 to 84 | -0.26 (-0.39,-0.14) | -0.99 (-1.09,-0.89) |
| High SDI | United States of America | 85 to 89 | 0.39 (0.21,0.58) | -0.25 (-0.4,-0.11) |
| High SDI | United States Virgin Islands | 5 to 9 | 3.07 (-18.65,30.59) | 4.13 (-18.1,32.38) |
| High SDI | United States Virgin Islands | 10 to 14 | 2.81 (-14.39,23.46) | 3.23 (-14.64,24.84) |
| High SDI | United States Virgin Islands | 15 to 19 | 3.64 (-11.41,21.25) | 2.16 (-14.14,21.55) |
| High SDI | United States Virgin Islands | 20 to 24 | 4.3 (-8.43,18.8) | 1.49 (-14.23,20.08) |
| High SDI | United States Virgin Islands | 25 to 29 | 4.54 (-6.09,16.38) | 1.43 (-14.09,19.75) |
| High SDI | United States Virgin Islands | 30 to 34 | 2.91 (-7.31,14.26) | 2.54 (-13,20.85) |
| High SDI | United States Virgin Islands | 35 to 39 | 2.06 (-8.05,13.28) | 1.03 (-13.93,18.6) |
| High SDI | United States Virgin Islands | 40 to 44 | 0.74 (-8.24,10.61) | 0.12 (-14.61,17.39) |
| High SDI | United States Virgin Islands | 45 to 49 | 0.65 (-5.97,7.74) | -0.01 (-14.06,16.35) |
| High SDI | United States Virgin Islands | 50 to 54 | 0.47 (-5.15,6.42) | -0.56 (-13.11,13.79) |
| High SDI | United States Virgin Islands | 55 to 59 | -0.77 (-5.96,4.71) | 0.45 (-10.58,12.83) |
| High SDI | United States Virgin Islands | 60 to 64 | -2.72 (-7.82,2.67) | 2.91 (-6.58,13.36) |
| High SDI | United States Virgin Islands | 65 to 69 | -3.27 (-8.43,2.17) | 1.72 (-7.2,11.48) |
| High SDI | United States Virgin Islands | 70 to 74 | -3.57 (-9.06,2.24) | 2.82 (-8.54,15.6) |
| High SDI | United States Virgin Islands | 75 to 79 | 0.73 (-10.34,13.16) | 2.33 (-11.3,18.05) |
| High SDI | United States Virgin Islands | 80 to 84 | 2.37 (-12.71,20.04) | -0.82 (-17.22,18.82) |
| High SDI | United States Virgin Islands | 85 to 89 | 0.33 (-20.35,26.4) | -3.47 (-24.01,22.61) |
| High-middle SDI | Antigua and Barbuda | 5 to 9 | 0.08 (-21.31,27.28) | - |
| High-middle SDI | Antigua and Barbuda | 10 to 14 | -0.52 (-17.76,20.33) | - |
| High-middle SDI | Antigua and Barbuda | 15 to 19 | -0.57 (-16.43,18.32) | - |
| High-middle SDI | Antigua and Barbuda | 20 to 24 | -0.28 (-15.71,17.98) | - |
| High-middle SDI | Antigua and Barbuda | 25 to 29 | -1.69 (-16.57,15.83) | - |
| High-middle SDI | Antigua and Barbuda | 30 to 34 | -0.67 (-15.49,16.73) | - |
| High-middle SDI | Antigua and Barbuda | 35 to 39 | 0.38 (-14.18,17.41) | - |
| High-middle SDI | Antigua and Barbuda | 40 to 44 | 1.5 (-12.42,17.63) | - |
| High-middle SDI | Antigua and Barbuda | 45 to 49 | 1.89 (-10.88,16.5) | - |
| High-middle SDI | Antigua and Barbuda | 50 to 54 | 3.3 (-8.52,16.63) | - |
| High-middle SDI | Antigua and Barbuda | 55 to 59 | 5.98 (-6.51,20.14) | - |
| High-middle SDI | Antigua and Barbuda | 60 to 64 | 8.81 (-3.41,22.58) | - |
| High-middle SDI | Antigua and Barbuda | 65 to 69 | 9.5 (-3.15,23.81) | - |
| High-middle SDI | Antigua and Barbuda | 70 to 74 | 8.38 (-4.88,23.5) | - |
| High-middle SDI | Antigua and Barbuda | 75 to 79 | 4.56 (-10.03,21.52) | - |
| High-middle SDI | Antigua and Barbuda | 80 to 84 | 0.26 (-16.9,20.98) | - |
| High-middle SDI | Antigua and Barbuda | 85 to 89 | 0.76 (-20.81,28.21) | - |
| High-middle SDI | Argentina | 5 to 9 | -0.19 (-1.4,1.04) | -1.91 (-4.15,0.38) |
| High-middle SDI | Argentina | 10 to 14 | 0.05 (-0.86,0.97) | -1.39 (-2.98,0.23) |
| High-middle SDI | Argentina | 15 to 19 | 0.3 (-0.43,1.03) | -1.22 (-2.44,0.02) |
| High-middle SDI | Argentina | 20 to 24 | 0.37 (-0.25,0.99) | -1.19 (-2.21,-0.17) |
| High-middle SDI | Argentina | 25 to 29 | 0.16 (-0.38,0.69) | -1.43 (-2.32,-0.53) |
| High-middle SDI | Argentina | 30 to 34 | 0.03 (-0.46,0.51) | -1.53 (-2.32,-0.72) |
| High-middle SDI | Argentina | 35 to 39 | 0 (-0.44,0.45) | -1.5 (-2.21,-0.78) |
| High-middle SDI | Argentina | 40 to 44 | -0.03 (-0.44,0.38) | -1.42 (-2.05,-0.79) |
| High-middle SDI | Argentina | 45 to 49 | 0.06 (-0.33,0.44) | -1.23 (-1.78,-0.67) |
| High-middle SDI | Argentina | 50 to 54 | 0.15 (-0.21,0.5) | -1.01 (-1.5,-0.52) |
| High-middle SDI | Argentina | 55 to 59 | 0.13 (-0.19,0.46) | -0.91 (-1.34,-0.48) |
| High-middle SDI | Argentina | 60 to 64 | 0.03 (-0.27,0.34) | -0.91 (-1.29,-0.53) |
| High-middle SDI | Argentina | 65 to 69 | -0.06 (-0.36,0.23) | -0.9 (-1.25,-0.55) |
| High-middle SDI | Argentina | 70 to 74 | -0.13 (-0.44,0.18) | -0.87 (-1.21,-0.52) |
| High-middle SDI | Argentina | 75 to 79 | 0.01 (-0.36,0.38) | -0.6 (-0.98,-0.22) |
| High-middle SDI | Argentina | 80 to 84 | 0.12 (-0.36,0.61) | -0.36 (-0.84,0.12) |
| High-middle SDI | Argentina | 85 to 89 | 0.99 (0.11,1.88) | 0.59 (-0.25,1.43) |
| High-middle SDI | Bahamas | 5 to 9 | -6.96 (-24.11,14.07) | -0.05 (-21.03,26.51) |
| High-middle SDI | Bahamas | 10 to 14 | -3.08 (-16.24,12.15) | 1.12 (-15.31,20.75) |
| High-middle SDI | Bahamas | 15 to 19 | 0.15 (-7.67,8.62) | 2.06 (-11.33,17.48) |
| High-middle SDI | Bahamas | 20 to 24 | 0.66 (-5.36,7.07) | 2.34 (-7.08,12.72) |
| High-middle SDI | Bahamas | 25 to 29 | 0.43 (-4.62,5.75) | 0.35 (-6.49,7.69) |
| High-middle SDI | Bahamas | 30 to 34 | 1.15 (-3.32,5.83) | -1.93 (-8.32,4.91) |
| High-middle SDI | Bahamas | 35 to 39 | 1.55 (-2.74,6.02) | -2.14 (-7.59,3.63) |
| High-middle SDI | Bahamas | 40 to 44 | 1.79 (-2.37,6.13) | -2.19 (-7.3,3.19) |
| High-middle SDI | Bahamas | 45 to 49 | 1.73 (-2.32,5.94) | -2.24 (-7.12,2.89) |
| High-middle SDI | Bahamas | 50 to 54 | 1.25 (-2.74,5.4) | -1.85 (-6.56,3.11) |
| High-middle SDI | Bahamas | 55 to 59 | 0.47 (-3.57,4.67) | -1.3 (-5.85,3.47) |
| High-middle SDI | Bahamas | 60 to 64 | 1.26 (-3.01,5.72) | -1.1 (-5.68,3.7) |
| High-middle SDI | Bahamas | 65 to 69 | 1.06 (-3.44,5.76) | -1.69 (-6.45,3.32) |
| High-middle SDI | Bahamas | 70 to 74 | 0.37 (-4.42,5.4) | -2.09 (-7.1,3.19) |
| High-middle SDI | Bahamas | 75 to 79 | -0.45 (-5.8,5.2) | -1.68 (-8.22,5.32) |
| High-middle SDI | Bahamas | 80 to 84 | 1.88 (-11.41,17.15) | 1.91 (-11.6,17.47) |
| High-middle SDI | Bahamas | 85 to 89 | -0.18 (-20.19,24.85) | 1.3 (-19.09,26.83) |
| High-middle SDI | Bahrain | 5 to 9 | -2.09 (-9.17,5.55) | -2.75 (-23.1,22.98) |
| High-middle SDI | Bahrain | 10 to 14 | -0.41 (-6.03,5.54) | -2.48 (-18.03,16.01) |
| High-middle SDI | Bahrain | 15 to 19 | 1.16 (-3.4,5.94) | -2.73 (-14.78,11.03) |
| High-middle SDI | Bahrain | 20 to 24 | 2 (-1.86,6) | -2.22 (-9.87,6.09) |
| High-middle SDI | Bahrain | 25 to 29 | 2 (-1.39,5.5) | -2.46 (-8.47,3.94) |
| High-middle SDI | Bahrain | 30 to 34 | 1.37 (-1.7,4.53) | -2.88 (-7.95,2.48) |
| High-middle SDI | Bahrain | 35 to 39 | 0.52 (-2.42,3.55) | -2.87 (-7.36,1.84) |
| High-middle SDI | Bahrain | 40 to 44 | 0.54 (-2.53,3.71) | -2.82 (-7.14,1.71) |
| High-middle SDI | Bahrain | 45 to 49 | 0.33 (-2.92,3.7) | -3.35 (-7.66,1.17) |
| High-middle SDI | Bahrain | 50 to 54 | -0.74 (-4.02,2.65) | -3.36 (-7.48,0.95) |
| High-middle SDI | Bahrain | 55 to 59 | -1.11 (-4.39,2.28) | -3.06 (-7.05,1.09) |
| High-middle SDI | Bahrain | 60 to 64 | -1.4 (-4.63,1.94) | -3.16 (-7.05,0.91) |
| High-middle SDI | Bahrain | 65 to 69 | -1.89 (-5.23,1.57) | -2.85 (-6.81,1.28) |
| High-middle SDI | Bahrain | 70 to 74 | -0.77 (-4.97,3.63) | -1.69 (-6.21,3.05) |
| High-middle SDI | Bahrain | 75 to 79 | -0.7 (-5.73,4.6) | -1.57 (-6.79,3.94) |
| High-middle SDI | Bahrain | 80 to 84 | 2.58 (-10.18,17.14) | 1.92 (-10.85,16.5) |
| High-middle SDI | Bahrain | 85 to 89 | 2.72 (-16.18,25.89) | 2.39 (-16.48,25.52) |
| High-middle SDI | Barbados | 5 to 9 | -0.07 (-20.33,25.35) | -0.17 (-21.2,26.47) |
| High-middle SDI | Barbados | 10 to 14 | -0.55 (-14.34,15.47) | -1.7 (-18.28,18.23) |
| High-middle SDI | Barbados | 15 to 19 | 1.61 (-5,8.69) | -2.36 (-16.88,14.71) |
| High-middle SDI | Barbados | 20 to 24 | 0.13 (-5.41,5.99) | -4.04 (-16.02,9.66) |
| High-middle SDI | Barbados | 25 to 29 | 0.18 (-4.61,5.21) | -3.96 (-13.52,6.65) |
| High-middle SDI | Barbados | 30 to 34 | 0.39 (-3.7,4.65) | -1.83 (-8.17,4.95) |
| High-middle SDI | Barbados | 35 to 39 | 0.38 (-3.34,4.25) | -1 (-6.51,4.83) |
| High-middle SDI | Barbados | 40 to 44 | 0.44 (-3.09,4.09) | -0.96 (-6.25,4.62) |
| High-middle SDI | Barbados | 45 to 49 | 0.26 (-3,3.63) | 0.12 (-4.58,5.06) |
| High-middle SDI | Barbados | 50 to 54 | 0.43 (-2.63,3.59) | -0.28 (-4.61,4.25) |
| High-middle SDI | Barbados | 55 to 59 | 0.5 (-2.38,3.47) | -0.6 (-4.62,3.6) |
| High-middle SDI | Barbados | 60 to 64 | 0.63 (-2.07,3.41) | -0.87 (-4.49,2.89) |
| High-middle SDI | Barbados | 65 to 69 | 0.57 (-2.08,3.3) | -0.44 (-3.78,3.01) |
| High-middle SDI | Barbados | 70 to 74 | 0.2 (-2.48,2.96) | -0.47 (-3.77,2.95) |
| High-middle SDI | Barbados | 75 to 79 | -0.26 (-3.23,2.79) | -0.27 (-3.73,3.32) |
| High-middle SDI | Barbados | 80 to 84 | -0.46 (-3.99,3.21) | 0.94 (-3.33,5.39) |
| High-middle SDI | Barbados | 85 to 89 | 1.54 (-4.91,8.42) | 2.11 (-4.62,9.3) |
| High-middle SDI | Belarus | 5 to 9 | 5.54 (3.47,7.66) | 2.57 (-3.32,8.82) |
| High-middle SDI | Belarus | 10 to 14 | 5.36 (3.52,7.24) | 2.07 (-2.93,7.32) |
| High-middle SDI | Belarus | 15 to 19 | 4.52 (2.75,6.32) | 0.66 (-3.72,5.24) |
| High-middle SDI | Belarus | 20 to 24 | 4.04 (2.42,5.67) | 0.88 (-2.51,4.39) |
| High-middle SDI | Belarus | 25 to 29 | 4.08 (2.74,5.44) | 0.78 (-1.97,3.61) |
| High-middle SDI | Belarus | 30 to 34 | 4.26 (3.12,5.41) | 1.33 (-0.88,3.59) |
| High-middle SDI | Belarus | 35 to 39 | 4.53 (3.51,5.56) | 1.71 (-0.18,3.64) |
| High-middle SDI | Belarus | 40 to 44 | 4.58 (3.65,5.51) | 2.05 (0.43,3.7) |
| High-middle SDI | Belarus | 45 to 49 | 4.23 (3.39,5.07) | 1.81 (0.41,3.23) |
| High-middle SDI | Belarus | 50 to 54 | 4.12 (3.37,4.88) | 1.73 (0.54,2.93) |
| High-middle SDI | Belarus | 55 to 59 | 4.2 (3.53,4.86) | 1.94 (0.95,2.94) |
| High-middle SDI | Belarus | 60 to 64 | 4.15 (3.53,4.77) | 1.97 (1.08,2.86) |
| High-middle SDI | Belarus | 65 to 69 | 4.22 (3.59,4.85) | 2.17 (1.33,3.03) |
| High-middle SDI | Belarus | 70 to 74 | 4.09 (3.35,4.83) | 2.24 (1.31,3.18) |
| High-middle SDI | Belarus | 75 to 79 | 3.48 (2.5,4.47) | 1.84 (0.71,2.98) |
| High-middle SDI | Belarus | 80 to 84 | 3.09 (1.81,4.4) | 1.57 (0.17,2.98) |
| High-middle SDI | Belarus | 85 to 89 | 3.36 (0.94,5.85) | 1.62 (-0.85,4.15) |
| High-middle SDI | Brunei Darussalam | 5 to 9 | -2.92 (-22.96,22.33) | -0.56 (-21.53,26.03) |
| High-middle SDI | Brunei Darussalam | 10 to 14 | -3.96 (-18.71,13.47) | -0.89 (-17.7,19.35) |
| High-middle SDI | Brunei Darussalam | 15 to 19 | -2.68 (-14.54,10.83) | -0.47 (-15.57,17.32) |
| High-middle SDI | Brunei Darussalam | 20 to 24 | -0.99 (-8.3,6.9) | -0.45 (-13.96,15.18) |
| High-middle SDI | Brunei Darussalam | 25 to 29 | -0.19 (-5.88,5.86) | -1.01 (-12.17,11.55) |
| High-middle SDI | Brunei Darussalam | 30 to 34 | -0.33 (-5.29,4.9) | -1.13 (-8.46,6.79) |
| High-middle SDI | Brunei Darussalam | 35 to 39 | 0.5 (-4.1,5.31) | -1.73 (-7.55,4.46) |
| High-middle SDI | Brunei Darussalam | 40 to 44 | 1.28 (-3.08,5.82) | -1.97 (-7.81,4.23) |
| High-middle SDI | Brunei Darussalam | 45 to 49 | 1.03 (-3.19,5.44) | -1.72 (-7.32,4.21) |
| High-middle SDI | Brunei Darussalam | 50 to 54 | 0.5 (-3.67,4.85) | -1.32 (-6.69,4.36) |
| High-middle SDI | Brunei Darussalam | 55 to 59 | -0.15 (-4.21,4.07) | -1.76 (-6.69,3.44) |
| High-middle SDI | Brunei Darussalam | 60 to 64 | 0.31 (-3.82,4.63) | -1.34 (-5.97,3.52) |
| High-middle SDI | Brunei Darussalam | 65 to 69 | -0.24 (-4.59,4.3) | -1.43 (-5.98,3.34) |
| High-middle SDI | Brunei Darussalam | 70 to 74 | -0.16 (-4.84,4.75) | -1.11 (-5.76,3.77) |
| High-middle SDI | Brunei Darussalam | 75 to 79 | -0.27 (-5.58,5.35) | -0.39 (-5.57,5.08) |
| High-middle SDI | Brunei Darussalam | 80 to 84 | 5.5 (-7.63,20.49) | 0.15 (-6.53,7.32) |
| High-middle SDI | Brunei Darussalam | 85 to 89 | 8.7 (-10.96,32.7) | 5.73 (-12.19,27.31) |
| High-middle SDI | Bulgaria | 5 to 9 | 0.03 (-3.04,3.19) | -1.83 (-8.3,5.09) |
| High-middle SDI | Bulgaria | 10 to 14 | 1.43 (-0.8,3.7) | -1.32 (-6.11,3.72) |
| High-middle SDI | Bulgaria | 15 to 19 | 2.31 (0.35,4.31) | -0.06 (-3.81,3.84) |
| High-middle SDI | Bulgaria | 20 to 24 | 2.51 (0.72,4.32) | 0.6 (-2.56,3.86) |
| High-middle SDI | Bulgaria | 25 to 29 | 2.47 (0.92,4.04) | 0.69 (-1.98,3.43) |
| High-middle SDI | Bulgaria | 30 to 34 | 2.27 (0.92,3.63) | 0.46 (-1.87,2.84) |
| High-middle SDI | Bulgaria | 35 to 39 | 2.33 (1.17,3.5) | 0.41 (-1.62,2.47) |
| High-middle SDI | Bulgaria | 40 to 44 | 2.35 (1.36,3.34) | 0.39 (-1.35,2.15) |
| High-middle SDI | Bulgaria | 45 to 49 | 2.68 (1.83,3.53) | 0.78 (-0.69,2.27) |
| High-middle SDI | Bulgaria | 50 to 54 | 3.01 (2.26,3.76) | 1.26 (0,2.53) |
| High-middle SDI | Bulgaria | 55 to 59 | 3.12 (2.45,3.78) | 1.62 (0.56,2.7) |
| High-middle SDI | Bulgaria | 60 to 64 | 3.08 (2.47,3.69) | 1.85 (0.92,2.79) |
| High-middle SDI | Bulgaria | 65 to 69 | 2.87 (2.27,3.48) | 1.98 (1.13,2.84) |
| High-middle SDI | Bulgaria | 70 to 74 | 2.54 (1.88,3.2) | 1.94 (1.08,2.81) |
| High-middle SDI | Bulgaria | 75 to 79 | 2.29 (1.38,3.21) | 1.86 (0.81,2.92) |
| High-middle SDI | Bulgaria | 80 to 84 | 2.26 (1.05,3.49) | 1.98 (0.67,3.3) |
| High-middle SDI | Bulgaria | 85 to 89 | 1.76 (-0.39,3.95) | 1.66 (-0.63,4.01) |
| High-middle SDI | Chile | 5 to 9 | 0.01 (-2.39,2.46) | -3.19 (-7.7,1.55) |
| High-middle SDI | Chile | 10 to 14 | 0.64 (-1.18,2.5) | -2.58 (-5.9,0.86) |
| High-middle SDI | Chile | 15 to 19 | 1.07 (-0.39,2.56) | -1.66 (-4.05,0.79) |
| High-middle SDI | Chile | 20 to 24 | 1.22 (0.02,2.45) | -1.54 (-3.45,0.41) |
| High-middle SDI | Chile | 25 to 29 | 1.24 (0.23,2.26) | -1.49 (-3.09,0.13) |
| High-middle SDI | Chile | 30 to 34 | 1.18 (0.29,2.07) | -1.5 (-2.91,-0.06) |
| High-middle SDI | Chile | 35 to 39 | 1.04 (0.23,1.85) | -1.55 (-2.82,-0.27) |
| High-middle SDI | Chile | 40 to 44 | 0.88 (0.15,1.62) | -1.65 (-2.78,-0.51) |
| High-middle SDI | Chile | 45 to 49 | 0.53 (-0.12,1.2) | -1.85 (-2.83,-0.86) |
| High-middle SDI | Chile | 50 to 54 | 0.52 (-0.07,1.12) | -1.76 (-2.61,-0.9) |
| High-middle SDI | Chile | 55 to 59 | 0.61 (0.06,1.16) | -1.58 (-2.33,-0.81) |
| High-middle SDI | Chile | 60 to 64 | 0.7 (0.18,1.22) | -1.35 (-2.03,-0.66) |
| High-middle SDI | Chile | 65 to 69 | 0.77 (0.26,1.29) | -1.1 (-1.74,-0.46) |
| High-middle SDI | Chile | 70 to 74 | 0.86 (0.32,1.41) | -0.81 (-1.44,-0.17) |
| High-middle SDI | Chile | 75 to 79 | 0.7 (0.06,1.33) | -0.74 (-1.43,-0.05) |
| High-middle SDI | Chile | 80 to 84 | 0.63 (-0.22,1.49) | -0.59 (-1.45,0.28) |
| High-middle SDI | Chile | 85 to 89 | 0.96 (-0.48,2.42) | -0.03 (-1.45,1.41) |
| High-middle SDI | Croatia | 5 to 9 | 1.08 (-2.13,4.39) | 1.33 (-5.96,9.19) |
| High-middle SDI | Croatia | 10 to 14 | 1.84 (-0.54,4.26) | 1.04 (-4.57,6.99) |
| High-middle SDI | Croatia | 15 to 19 | 1.91 (-0.12,3.99) | 0.37 (-4.28,5.25) |
| High-middle SDI | Croatia | 20 to 24 | 2.01 (0.15,3.89) | 0.07 (-3.88,4.17) |
| High-middle SDI | Croatia | 25 to 29 | 2.1 (0.43,3.81) | -0.5 (-3.98,3.11) |
| High-middle SDI | Croatia | 30 to 34 | 1.73 (0.22,3.26) | -0.94 (-4.02,2.24) |
| High-middle SDI | Croatia | 35 to 39 | 1.17 (-0.17,2.53) | -1.42 (-4.1,1.33) |
| High-middle SDI | Croatia | 40 to 44 | 1.01 (-0.17,2.21) | -1.32 (-3.59,0.99) |
| High-middle SDI | Croatia | 45 to 49 | 1.11 (0.07,2.16) | -1.17 (-3.06,0.75) |
| High-middle SDI | Croatia | 50 to 54 | 1.53 (0.64,2.43) | -0.57 (-2.08,0.95) |
| High-middle SDI | Croatia | 55 to 59 | 1.98 (1.21,2.76) | -0.12 (-1.35,1.12) |
| High-middle SDI | Croatia | 60 to 64 | 2.24 (1.56,2.92) | 0.28 (-0.75,1.31) |
| High-middle SDI | Croatia | 65 to 69 | 2.37 (1.72,3.02) | 0.51 (-0.41,1.43) |
| High-middle SDI | Croatia | 70 to 74 | 2.75 (2.06,3.44) | 1.03 (0.13,1.94) |
| High-middle SDI | Croatia | 75 to 79 | 2.93 (2.02,3.86) | 1.4 (0.31,2.5) |
| High-middle SDI | Croatia | 80 to 84 | 2.86 (1.64,4.1) | 1.39 (0,2.79) |
| High-middle SDI | Croatia | 85 to 89 | 2.92 (1.14,4.73) | 1.61 (-0.49,3.75) |
| High-middle SDI | Dominica | 5 to 9 | 2.29 (-19.46,29.92) | 1.12 (-20.45,28.55) |
| High-middle SDI | Dominica | 10 to 14 | 1.13 (-16.35,22.26) | 0.66 (-16.76,21.73) |
| High-middle SDI | Dominica | 15 to 19 | 0.31 (-15.68,19.35) | 0.37 (-15.64,19.43) |
| High-middle SDI | Dominica | 20 to 24 | 1.02 (-14.59,19.49) | 0.53 (-15.04,18.95) |
| High-middle SDI | Dominica | 25 to 29 | -0.51 (-15.4,17.01) | 1.02 (-14.43,19.26) |
| High-middle SDI | Dominica | 30 to 34 | 0.01 (-14.78,17.36) | -0.48 (-15.42,17.1) |
| High-middle SDI | Dominica | 35 to 39 | 0.92 (-13.46,17.69) | 0.12 (-14.8,17.66) |
| High-middle SDI | Dominica | 40 to 44 | 2.13 (-11.38,17.69) | 0.63 (-13.97,17.71) |
| High-middle SDI | Dominica | 45 to 49 | 2.91 (-9.17,16.59) | 1.9 (-12.07,18.09) |
| High-middle SDI | Dominica | 50 to 54 | 3.96 (-5.64,14.53) | 3.95 (-9.06,18.83) |
| High-middle SDI | Dominica | 55 to 59 | 1.74 (-6.61,10.84) | 5.33 (-6.48,18.64) |
| High-middle SDI | Dominica | 60 to 64 | 0.25 (-6.28,7.24) | 3.93 (-6.85,15.95) |
| High-middle SDI | Dominica | 65 to 69 | -0.84 (-6.59,5.26) | 1.49 (-6,9.58) |
| High-middle SDI | Dominica | 70 to 74 | -1.1 (-6.98,5.14) | -0.42 (-6.73,6.32) |
| High-middle SDI | Dominica | 75 to 79 | -0.69 (-7.86,7.04) | -0.84 (-8.23,7.14) |
| High-middle SDI | Dominica | 80 to 84 | -0.68 (-14.72,15.66) | -0.42 (-14.8,16.38) |
| High-middle SDI | Dominica | 85 to 89 | -0.88 (-21.21,24.7) | -0.13 (-21.02,26.3) |
| High-middle SDI | Georgia | 5 to 9 | -4.5 (-8.73,-0.07) | -2.17 (-8.58,4.69) |
| High-middle SDI | Georgia | 10 to 14 | -3.34 (-6.3,-0.3) | -2.43 (-7.28,2.66) |
| High-middle SDI | Georgia | 15 to 19 | -2.3 (-4.72,0.19) | -2.89 (-7.01,1.42) |
| High-middle SDI | Georgia | 20 to 24 | -1.85 (-3.92,0.28) | -2.97 (-6.58,0.77) |
| High-middle SDI | Georgia | 25 to 29 | -1.63 (-3.43,0.2) | -1.96 (-4.87,1.03) |
| High-middle SDI | Georgia | 30 to 34 | -0.92 (-2.51,0.7) | -0.96 (-3.51,1.67) |
| High-middle SDI | Georgia | 35 to 39 | -0.12 (-1.61,1.39) | -0.53 (-2.91,1.92) |
| High-middle SDI | Georgia | 40 to 44 | 0.86 (-0.55,2.29) | 0.77 (-1.38,2.97) |
| High-middle SDI | Georgia | 45 to 49 | 1.94 (0.6,3.3) | 1.78 (-0.19,3.79) |
| High-middle SDI | Georgia | 50 to 54 | 2.68 (1.43,3.95) | 2.48 (0.72,4.27) |
| High-middle SDI | Georgia | 55 to 59 | 3.27 (2.11,4.44) | 3.09 (1.52,4.68) |
| High-middle SDI | Georgia | 60 to 64 | 3.56 (2.44,4.7) | 3.43 (1.97,4.91) |
| High-middle SDI | Georgia | 65 to 69 | 3.71 (2.53,4.89) | 3.67 (2.21,5.14) |
| High-middle SDI | Georgia | 70 to 74 | 3.79 (2.33,5.27) | 3.95 (2.25,5.68) |
| High-middle SDI | Georgia | 75 to 79 | 4.05 (2.09,6.05) | 4.2 (2.1,6.35) |
| High-middle SDI | Georgia | 80 to 84 | 3.9 (1.09,6.79) | 3.91 (1.06,6.84) |
| High-middle SDI | Georgia | 85 to 89 | 4.86 (-1.38,11.5) | 4.88 (-1.38,11.54) |
| High-middle SDI | Greece | 5 to 9 | -1.34 (-4.11,1.5) | -6.92 (-22.77,12.18) |
| High-middle SDI | Greece | 10 to 14 | -0.87 (-2.79,1.1) | -1.08 (-7.82,6.16) |
| High-middle SDI | Greece | 15 to 19 | -0.42 (-1.94,1.13) | -0.82 (-5.48,4.07) |
| High-middle SDI | Greece | 20 to 24 | -0.09 (-1.33,1.17) | -1.16 (-5.12,2.96) |
| High-middle SDI | Greece | 25 to 29 | 0.24 (-0.77,1.26) | -0.99 (-4.14,2.25) |
| High-middle SDI | Greece | 30 to 34 | 0.6 (-0.25,1.46) | -0.75 (-3.38,1.94) |
| High-middle SDI | Greece | 35 to 39 | 0.94 (0.19,1.69) | -0.38 (-2.52,1.81) |
| High-middle SDI | Greece | 40 to 44 | 1.03 (0.35,1.71) | -0.13 (-1.92,1.68) |
| High-middle SDI | Greece | 45 to 49 | 1.07 (0.46,1.69) | -0.02 (-1.53,1.51) |
| High-middle SDI | Greece | 50 to 54 | 1.41 (0.84,1.98) | 0.38 (-0.88,1.66) |
| High-middle SDI | Greece | 55 to 59 | 1.51 (0.99,2.03) | 0.51 (-0.55,1.58) |
| High-middle SDI | Greece | 60 to 64 | 1.39 (0.92,1.85) | 0.46 (-0.43,1.35) |
| High-middle SDI | Greece | 65 to 69 | 1.06 (0.62,1.49) | 0.24 (-0.52,1.01) |
| High-middle SDI | Greece | 70 to 74 | 0.83 (0.39,1.27) | 0.09 (-0.6,0.8) |
| High-middle SDI | Greece | 75 to 79 | 0.46 (-0.02,0.96) | -0.13 (-0.82,0.56) |
| High-middle SDI | Greece | 80 to 84 | 0.33 (-0.26,0.92) | -0.15 (-0.89,0.6) |
| High-middle SDI | Greece | 85 to 89 | 0.8 (-0.15,1.76) | 0.46 (-0.71,1.64) |
| High-middle SDI | Guam | 5 to 9 | 1.07 (-17.55,23.9) | -0.68 (-21.98,26.43) |
| High-middle SDI | Guam | 10 to 14 | 0.5 (-6.75,8.32) | -0.01 (-17.34,20.97) |
| High-middle SDI | Guam | 15 to 19 | 0.16 (-5.85,6.55) | -0.05 (-16,18.94) |
| High-middle SDI | Guam | 20 to 24 | 0.45 (-5.09,6.33) | 0.29 (-15.24,18.67) |
| High-middle SDI | Guam | 25 to 29 | 0.99 (-4.35,6.64) | 1.09 (-14.37,19.34) |
| High-middle SDI | Guam | 30 to 34 | 2.01 (-3.87,8.26) | 0.44 (-14.64,18.19) |
| High-middle SDI | Guam | 35 to 39 | 2.32 (-3.89,8.93) | 0.42 (-14.64,18.13) |
| High-middle SDI | Guam | 40 to 44 | 3.68 (-3.77,11.7) | 0.69 (-14.13,18.07) |
| High-middle SDI | Guam | 45 to 49 | 6.28 (-2.72,16.12) | 1.34 (-12.89,17.9) |
| High-middle SDI | Guam | 50 to 54 | 7.08 (-2.98,18.19) | 3.94 (-9.89,19.88) |
| High-middle SDI | Guam | 55 to 59 | 6.22 (-4.22,17.79) | 6.59 (-6.26,21.21) |
| High-middle SDI | Guam | 60 to 64 | 8.39 (-2.93,21.04) | 6.14 (-6.7,20.74) |
| High-middle SDI | Guam | 65 to 69 | 2.69 (-5.27,11.3) | 4.36 (-8.95,19.62) |
| High-middle SDI | Guam | 70 to 74 | 2.2 (-8.67,14.37) | 1.54 (-12.52,17.85) |
| High-middle SDI | Guam | 75 to 79 | 1.32 (-12,16.66) | -2.17 (-17.63,16.2) |
| High-middle SDI | Guam | 80 to 84 | -1.74 (-17.78,17.42) | -3.93 (-20.54,16.16) |
| High-middle SDI | Guam | 85 to 89 | -5.2 (-25.3,20.29) | -5.09 (-25.47,20.87) |
| High-middle SDI | Hungary | 5 to 9 | 1.37 (-1.22,4.03) | -1.19 (-7.79,5.88) |
| High-middle SDI | Hungary | 10 to 14 | 1.7 (-0.25,3.69) | -1.19 (-5.93,3.8) |
| High-middle SDI | Hungary | 15 to 19 | 2.04 (0.41,3.71) | -0.18 (-3.6,3.36) |
| High-middle SDI | Hungary | 20 to 24 | 1.98 (0.59,3.39) | -0.65 (-3.48,2.26) |
| High-middle SDI | Hungary | 25 to 29 | 1.37 (0.2,2.55) | -1.3 (-3.69,1.15) |
| High-middle SDI | Hungary | 30 to 34 | 0.43 (-0.57,1.43) | -2.1 (-4.12,-0.03) |
| High-middle SDI | Hungary | 35 to 39 | -0.32 (-1.15,0.52) | -2.63 (-4.29,-0.95) |
| High-middle SDI | Hungary | 40 to 44 | -0.48 (-1.18,0.22) | -2.59 (-3.92,-1.24) |
| High-middle SDI | Hungary | 45 to 49 | 0.07 (-0.54,0.69) | -1.91 (-3.02,-0.78) |
| High-middle SDI | Hungary | 50 to 54 | 0.74 (0.2,1.29) | -1.11 (-2.05,-0.17) |
| High-middle SDI | Hungary | 55 to 59 | 1.22 (0.73,1.71) | -0.51 (-1.3,0.28) |
| High-middle SDI | Hungary | 60 to 64 | 1.23 (0.8,1.67) | -0.37 (-1.03,0.29) |
| High-middle SDI | Hungary | 65 to 69 | 0.99 (0.57,1.42) | -0.46 (-1.05,0.14) |
| High-middle SDI | Hungary | 70 to 74 | 0.7 (0.26,1.15) | -0.56 (-1.15,0.03) |
| High-middle SDI | Hungary | 75 to 79 | 0.68 (0.11,1.24) | -0.4 (-1.07,0.27) |
| High-middle SDI | Hungary | 80 to 84 | 0.81 (0.09,1.54) | -0.09 (-0.89,0.72) |
| High-middle SDI | Hungary | 85 to 89 | 0.84 (-0.38,2.08) | 0.07 (-1.25,1.4) |
| High-middle SDI | Israel | 5 to 9 | -0.37 (-1.9,1.18) | -2 (-7.01,3.27) |
| High-middle SDI | Israel | 10 to 14 | 0.38 (-0.83,1.61) | -1.92 (-5.73,2.04) |
| High-middle SDI | Israel | 15 to 19 | 0.88 (-0.18,1.95) | -1.86 (-4.93,1.3) |
| High-middle SDI | Israel | 20 to 24 | 0.69 (-0.28,1.66) | -2.34 (-5.06,0.47) |
| High-middle SDI | Israel | 25 to 29 | 0.47 (-0.4,1.35) | -2.41 (-4.82,0.06) |
| High-middle SDI | Israel | 30 to 34 | 0.32 (-0.47,1.12) | -2.33 (-4.43,-0.19) |
| High-middle SDI | Israel | 35 to 39 | 0.15 (-0.59,0.89) | -2.41 (-4.26,-0.53) |
| High-middle SDI | Israel | 40 to 44 | 0.25 (-0.43,0.93) | -2.38 (-3.96,-0.77) |
| High-middle SDI | Israel | 45 to 49 | 0.64 (0.02,1.26) | -1.96 (-3.31,-0.6) |
| High-middle SDI | Israel | 50 to 54 | 0.89 (0.31,1.47) | -1.54 (-2.68,-0.38) |
| High-middle SDI | Israel | 55 to 59 | 0.8 (0.28,1.32) | -1.57 (-2.52,-0.61) |
| High-middle SDI | Israel | 60 to 64 | 0.55 (0.08,1.03) | -1.71 (-2.51,-0.9) |
| High-middle SDI | Israel | 65 to 69 | 0.42 (-0.02,0.87) | -1.65 (-2.34,-0.95) |
| High-middle SDI | Israel | 70 to 74 | 0.49 (0.04,0.94) | -1.41 (-2.05,-0.76) |
| High-middle SDI | Israel | 75 to 79 | 0.67 (0.15,1.19) | -0.97 (-1.63,-0.3) |
| High-middle SDI | Israel | 80 to 84 | 0.98 (0.35,1.61) | -0.37 (-1.1,0.37) |
| High-middle SDI | Israel | 85 to 89 | 1.03 (-0.01,2.08) | -0.19 (-1.33,0.97) |
| High-middle SDI | Italy | 5 to 9 | -1.72 (-2.49,-0.95) | -3.79 (-6.91,-0.57) |
| High-middle SDI | Italy | 10 to 14 | -1.37 (-1.89,-0.84) | -3.43 (-5.46,-1.37) |
| High-middle SDI | Italy | 15 to 19 | -0.93 (-1.35,-0.52) | -3.12 (-4.62,-1.61) |
| High-middle SDI | Italy | 20 to 24 | -0.53 (-0.88,-0.18) | -2.81 (-4,-1.6) |
| High-middle SDI | Italy | 25 to 29 | -0.55 (-0.85,-0.26) | -2.78 (-3.76,-1.79) |
| High-middle SDI | Italy | 30 to 34 | -0.75 (-1.01,-0.49) | -2.92 (-3.75,-2.09) |
| High-middle SDI | Italy | 35 to 39 | -0.9 (-1.13,-0.67) | -3.01 (-3.7,-2.31) |
| High-middle SDI | Italy | 40 to 44 | -0.93 (-1.13,-0.72) | -2.95 (-3.52,-2.38) |
| High-middle SDI | Italy | 45 to 49 | -0.89 (-1.08,-0.71) | -2.84 (-3.31,-2.38) |
| High-middle SDI | Italy | 50 to 54 | -0.61 (-0.77,-0.45) | -2.52 (-2.9,-2.14) |
| High-middle SDI | Italy | 55 to 59 | -0.34 (-0.49,-0.19) | -2.22 (-2.54,-1.9) |
| High-middle SDI | Italy | 60 to 64 | -0.17 (-0.3,-0.03) | -2.01 (-2.28,-1.75) |
| High-middle SDI | Italy | 65 to 69 | 0.09 (-0.04,0.22) | -1.7 (-1.93,-1.48) |
| High-middle SDI | Italy | 70 to 74 | 0.46 (0.33,0.59) | -1.23 (-1.43,-1.03) |
| High-middle SDI | Italy | 75 to 79 | 0.8 (0.64,0.95) | -0.74 (-0.94,-0.54) |
| High-middle SDI | Italy | 80 to 84 | 1.24 (1.04,1.43) | -0.12 (-0.35,0.1) |
| High-middle SDI | Italy | 85 to 89 | 1.76 (1.43,2.09) | 0.62 (0.27,0.98) |
| High-middle SDI | Jordan | 5 to 9 | 1.36 (-0.04,2.78) | -2.05 (-4.81,0.79) |
| High-middle SDI | Jordan | 10 to 14 | 1.59 (0.29,2.89) | -1.93 (-4.28,0.48) |
| High-middle SDI | Jordan | 15 to 19 | 2 (0.74,3.29) | -1.43 (-3.63,0.81) |
| High-middle SDI | Jordan | 20 to 24 | 2.27 (1.04,3.51) | -0.94 (-2.99,1.15) |
| High-middle SDI | Jordan | 25 to 29 | 2.39 (1.2,3.6) | -0.78 (-2.73,1.2) |
| High-middle SDI | Jordan | 30 to 34 | 2.2 (1.02,3.39) | -0.9 (-2.78,1.01) |
| High-middle SDI | Jordan | 35 to 39 | 1.85 (0.69,3.03) | -0.94 (-2.74,0.9) |
| High-middle SDI | Jordan | 40 to 44 | 1.56 (0.39,2.74) | -1.04 (-2.77,0.73) |
| High-middle SDI | Jordan | 45 to 49 | 1.07 (-0.07,2.23) | -1.31 (-2.93,0.35) |
| High-middle SDI | Jordan | 50 to 54 | 0.69 (-0.42,1.81) | -1.47 (-2.98,0.06) |
| High-middle SDI | Jordan | 55 to 59 | 0.63 (-0.46,1.73) | -1.44 (-2.86,-0.01) |
| High-middle SDI | Jordan | 60 to 64 | 0.76 (-0.37,1.9) | -1.17 (-2.56,0.24) |
| High-middle SDI | Jordan | 65 to 69 | 1.04 (-0.23,2.31) | -0.7 (-2.15,0.77) |
| High-middle SDI | Jordan | 70 to 74 | 1.36 (-0.18,2.93) | -0.04 (-1.72,1.66) |
| High-middle SDI | Jordan | 75 to 79 | 1.64 (-0.58,3.91) | 0.24 (-1.95,2.48) |
| High-middle SDI | Jordan | 80 to 84 | 1.6 (-1.66,4.96) | 0.16 (-2.81,3.22) |
| High-middle SDI | Jordan | 85 to 89 | 2.52 (-3.58,9) | -0.19 (-4.7,4.54) |
| High-middle SDI | Kazakhstan | 5 to 9 | -1.47 (-3.1,0.19) | -3.97 (-6.94,-0.9) |
| High-middle SDI | Kazakhstan | 10 to 14 | -1.99 (-3.36,-0.61) | -4.41 (-6.75,-2) |
| High-middle SDI | Kazakhstan | 15 to 19 | -2.64 (-3.92,-1.34) | -4.98 (-7.04,-2.87) |
| High-middle SDI | Kazakhstan | 20 to 24 | -2.86 (-4.02,-1.69) | -5.1 (-6.89,-3.27) |
| High-middle SDI | Kazakhstan | 25 to 29 | -2.51 (-3.52,-1.49) | -4.51 (-6.04,-2.96) |
| High-middle SDI | Kazakhstan | 30 to 34 | -1.89 (-2.79,-0.98) | -3.98 (-5.33,-2.6) |
| High-middle SDI | Kazakhstan | 35 to 39 | -1.56 (-2.41,-0.7) | -3.58 (-4.83,-2.31) |
| High-middle SDI | Kazakhstan | 40 to 44 | -1.55 (-2.38,-0.71) | -3.42 (-4.59,-2.22) |
| High-middle SDI | Kazakhstan | 45 to 49 | -1.86 (-2.69,-1.02) | -3.54 (-4.67,-2.4) |
| High-middle SDI | Kazakhstan | 50 to 54 | -1.8 (-2.61,-1) | -3.33 (-4.38,-2.26) |
| High-middle SDI | Kazakhstan | 55 to 59 | -1.48 (-2.23,-0.72) | -2.89 (-3.84,-1.92) |
| High-middle SDI | Kazakhstan | 60 to 64 | -1.15 (-1.94,-0.36) | -2.49 (-3.44,-1.52) |
| High-middle SDI | Kazakhstan | 65 to 69 | -0.85 (-1.72,0.02) | -2.08 (-3.08,-1.07) |
| High-middle SDI | Kazakhstan | 70 to 74 | -0.58 (-1.7,0.55) | -1.66 (-2.89,-0.42) |
| High-middle SDI | Kazakhstan | 75 to 79 | -0.3 (-1.85,1.27) | -1.38 (-2.95,0.22) |
| High-middle SDI | Kazakhstan | 80 to 84 | -0.18 (-2.33,2.02) | -1.15 (-3.25,1) |
| High-middle SDI | Kazakhstan | 85 to 89 | -0.27 (-4.17,3.79) | -0.98 (-4.85,3.05) |
| High-middle SDI | Lebanon | 5 to 9 | 1.65 (-0.71,4.05) | -0.99 (-6.23,4.54) |
| High-middle SDI | Lebanon | 10 to 14 | 2.25 (0.32,4.23) | -0.84 (-5.08,3.59) |
| High-middle SDI | Lebanon | 15 to 19 | 2.67 (0.95,4.43) | 0.03 (-3.2,3.36) |
| High-middle SDI | Lebanon | 20 to 24 | 2.72 (1.19,4.28) | -0.33 (-3.11,2.53) |
| High-middle SDI | Lebanon | 25 to 29 | 2.65 (1.29,4.02) | -0.53 (-2.98,1.99) |
| High-middle SDI | Lebanon | 30 to 34 | 2.42 (1.15,3.7) | -0.66 (-2.9,1.63) |
| High-middle SDI | Lebanon | 35 to 39 | 2.15 (0.94,3.36) | -0.85 (-2.91,1.24) |
| High-middle SDI | Lebanon | 40 to 44 | 2.02 (0.89,3.16) | -0.72 (-2.56,1.16) |
| High-middle SDI | Lebanon | 45 to 49 | 1.92 (0.88,2.96) | -0.62 (-2.22,1.02) |
| High-middle SDI | Lebanon | 50 to 54 | 1.75 (0.8,2.71) | -0.62 (-2.02,0.8) |
| High-middle SDI | Lebanon | 55 to 59 | 1.61 (0.74,2.49) | -0.6 (-1.82,0.63) |
| High-middle SDI | Lebanon | 60 to 64 | 1.52 (0.71,2.34) | -0.46 (-1.54,0.64) |
| High-middle SDI | Lebanon | 65 to 69 | 1.44 (0.66,2.23) | -0.37 (-1.35,0.61) |
| High-middle SDI | Lebanon | 70 to 74 | 1.52 (0.68,2.36) | -0.08 (-1.04,0.89) |
| High-middle SDI | Lebanon | 75 to 79 | 1.65 (0.58,2.73) | 0.3 (-0.82,1.44) |
| High-middle SDI | Lebanon | 80 to 84 | 1.76 (0.22,3.32) | 0.62 (-0.91,2.16) |
| High-middle SDI | Lebanon | 85 to 89 | 1.92 (-0.64,4.54) | 0.73 (-1.67,3.19) |
| High-middle SDI | Libya | 5 to 9 | 3.33 (1.57,5.11) | 0.86 (-2.51,4.35) |
| High-middle SDI | Libya | 10 to 14 | 3.18 (1.67,4.7) | 1.11 (-1.58,3.88) |
| High-middle SDI | Libya | 15 to 19 | 2.95 (1.57,4.35) | 1.14 (-1.23,3.56) |
| High-middle SDI | Libya | 20 to 24 | 2.91 (1.61,4.22) | 1.27 (-0.87,3.46) |
| High-middle SDI | Libya | 25 to 29 | 2.94 (1.73,4.16) | 1.46 (-0.5,3.46) |
| High-middle SDI | Libya | 30 to 34 | 2.93 (1.79,4.09) | 1.29 (-0.51,3.12) |
| High-middle SDI | Libya | 35 to 39 | 2.76 (1.65,3.88) | 1.16 (-0.52,2.86) |
| High-middle SDI | Libya | 40 to 44 | 2.69 (1.58,3.81) | 1.15 (-0.46,2.79) |
| High-middle SDI | Libya | 45 to 49 | 2.58 (1.48,3.7) | 1.17 (-0.35,2.72) |
| High-middle SDI | Libya | 50 to 54 | 2.46 (1.38,3.56) | 1.12 (-0.32,2.58) |
| High-middle SDI | Libya | 55 to 59 | 2.28 (1.22,3.35) | 1.06 (-0.28,2.43) |
| High-middle SDI | Libya | 60 to 64 | 2.08 (1.06,3.12) | 0.93 (-0.33,2.21) |
| High-middle SDI | Libya | 65 to 69 | 1.92 (0.86,2.99) | 0.89 (-0.35,2.13) |
| High-middle SDI | Libya | 70 to 74 | 1.73 (0.55,2.92) | 0.86 (-0.42,2.15) |
| High-middle SDI | Libya | 75 to 79 | 1.84 (0.37,3.32) | 1.13 (-0.36,2.65) |
| High-middle SDI | Libya | 80 to 84 | 2.35 (0.22,4.53) | 1.64 (-0.36,3.69) |
| High-middle SDI | Libya | 85 to 89 | 2.96 (-0.73,6.78) | 2.06 (-1.19,5.43) |
| High-middle SDI | Malaysia | 5 to 9 | 0.85 (-0.97,2.71) | -1.37 (-4.33,1.69) |
| High-middle SDI | Malaysia | 10 to 14 | 1 (-0.31,2.32) | -1.06 (-3.01,0.94) |
| High-middle SDI | Malaysia | 15 to 19 | 0.99 (-0.1,2.09) | -1.04 (-2.55,0.5) |
| High-middle SDI | Malaysia | 20 to 24 | 0.99 (0.02,1.98) | -0.87 (-2.18,0.45) |
| High-middle SDI | Malaysia | 25 to 29 | 1.15 (0.26,2.05) | -0.6 (-1.8,0.6) |
| High-middle SDI | Malaysia | 30 to 34 | 1.48 (0.65,2.31) | -0.15 (-1.24,0.96) |
| High-middle SDI | Malaysia | 35 to 39 | 1.77 (1,2.55) | 0.31 (-0.69,1.33) |
| High-middle SDI | Malaysia | 40 to 44 | 1.99 (1.27,2.73) | 0.72 (-0.2,1.64) |
| High-middle SDI | Malaysia | 45 to 49 | 1.97 (1.29,2.66) | 0.82 (0,1.66) |
| High-middle SDI | Malaysia | 50 to 54 | 1.53 (0.9,2.18) | 0.55 (-0.2,1.3) |
| High-middle SDI | Malaysia | 55 to 59 | 1 (0.4,1.61) | 0.1 (-0.58,0.79) |
| High-middle SDI | Malaysia | 60 to 64 | 0.54 (-0.06,1.14) | -0.29 (-0.94,0.37) |
| High-middle SDI | Malaysia | 65 to 69 | 0.3 (-0.33,0.94) | -0.42 (-1.09,0.25) |
| High-middle SDI | Malaysia | 70 to 74 | 0.01 (-0.72,0.74) | -0.64 (-1.37,0.1) |
| High-middle SDI | Malaysia | 75 to 79 | 0.35 (-0.61,1.32) | -0.22 (-1.14,0.71) |
| High-middle SDI | Malaysia | 80 to 84 | 1.11 (-0.3,2.55) | 0.65 (-0.65,1.96) |
| High-middle SDI | Malaysia | 85 to 89 | 1.9 (-0.42,4.27) | 1.26 (-0.76,3.33) |
| High-middle SDI | Malta | 5 to 9 | 3.16 (-1.83,8.4) | 1.7 (-19.85,29.05) |
| High-middle SDI | Malta | 10 to 14 | 0.47 (-3.96,5.1) | 2.12 (-15.5,23.42) |
| High-middle SDI | Malta | 15 to 19 | 0.26 (-3.34,3.98) | -0.22 (-15.96,18.47) |
| High-middle SDI | Malta | 20 to 24 | 0.57 (-2.49,3.73) | 0.68 (-14.6,18.68) |
| High-middle SDI | Malta | 25 to 29 | 1.18 (-1.71,4.15) | 1.4 (-13.41,18.74) |
| High-middle SDI | Malta | 30 to 34 | 1.78 (-1.05,4.7) | 2.43 (-11.36,18.36) |
| High-middle SDI | Malta | 35 to 39 | 2.62 (-0.18,5.5) | 3.31 (-8.68,16.87) |
| High-middle SDI | Malta | 40 to 44 | 2.82 (0.09,5.62) | 3.83 (-5.25,13.78) |
| High-middle SDI | Malta | 45 to 49 | 2.89 (0.3,5.55) | 0.81 (-5.82,7.91) |
| High-middle SDI | Malta | 50 to 54 | 2.6 (0.13,5.13) | 0.49 (-4.77,6.03) |
| High-middle SDI | Malta | 55 to 59 | 1.86 (-0.36,4.13) | -0.22 (-4.43,4.16) |
| High-middle SDI | Malta | 60 to 64 | 1.08 (-0.91,3.12) | -0.79 (-4.25,2.8) |
| High-middle SDI | Malta | 65 to 69 | 0.72 (-1.13,2.61) | -1.23 (-4.21,1.84) |
| High-middle SDI | Malta | 70 to 74 | 0.48 (-1.33,2.33) | -1.83 (-4.49,0.9) |
| High-middle SDI | Malta | 75 to 79 | 0.72 (-1.45,2.95) | -1.55 (-4.44,1.44) |
| High-middle SDI | Malta | 80 to 84 | 1.22 (-1.63,4.15) | -1.34 (-4.68,2.12) |
| High-middle SDI | Malta | 85 to 89 | 1.54 (-3.02,6.31) | -1.43 (-6.28,3.67) |
| High-middle SDI | Montenegro | 5 to 9 | -5.45 (-21.41,13.76) | 1.02 (-20.4,28.2) |
| High-middle SDI | Montenegro | 10 to 14 | 0.53 (-6.28,7.83) | 1.21 (-16.26,22.34) |
| High-middle SDI | Montenegro | 15 to 19 | 1.39 (-4.01,7.09) | 1.79 (-14.42,21.06) |
| High-middle SDI | Montenegro | 20 to 24 | 1.11 (-4.03,6.53) | 0.89 (-14.43,18.95) |
| High-middle SDI | Montenegro | 25 to 29 | 0.83 (-4.08,6) | -0.17 (-15.02,17.29) |
| High-middle SDI | Montenegro | 30 to 34 | 0.44 (-4.09,5.2) | -1.61 (-15.47,14.52) |
| High-middle SDI | Montenegro | 35 to 39 | 0.47 (-3.64,4.74) | -2.22 (-14.53,11.86) |
| High-middle SDI | Montenegro | 40 to 44 | 0.47 (-3.27,4.36) | -2.52 (-12.33,8.38) |
| High-middle SDI | Montenegro | 45 to 49 | 1.03 (-2.29,4.47) | -1.32 (-7.73,5.54) |
| High-middle SDI | Montenegro | 50 to 54 | 1.37 (-1.6,4.44) | -0.85 (-6.03,4.61) |
| High-middle SDI | Montenegro | 55 to 59 | 1.61 (-1.04,4.33) | -0.38 (-4.65,4.09) |
| High-middle SDI | Montenegro | 60 to 64 | 1.5 (-0.94,4.01) | -0.16 (-4.01,3.85) |
| High-middle SDI | Montenegro | 65 to 69 | 1.73 (-0.78,4.3) | 0.31 (-3.33,4.09) |
| High-middle SDI | Montenegro | 70 to 74 | 1.88 (-1.07,4.93) | 0.31 (-3.44,4.21) |
| High-middle SDI | Montenegro | 75 to 79 | 1.76 (-2.13,5.8) | 1.15 (-3.64,6.18) |
| High-middle SDI | Montenegro | 80 to 84 | 2.1 (-2.84,7.3) | 1 (-4.38,6.68) |
| High-middle SDI | Montenegro | 85 to 89 | 1.26 (-5.71,8.74) | 6.24 (-11.74,27.88) |
| High-middle SDI | North Macedonia | 5 to 9 | 0.34 (-6.11,7.24) | -8.26 (-24.97,12.17) |
| High-middle SDI | North Macedonia | 10 to 14 | 2.11 (-2.11,6.51) | -9.61 (-21.98,4.73) |
| High-middle SDI | North Macedonia | 15 to 19 | 1.86 (-2.02,5.88) | -8.95 (-19.49,2.97) |
| High-middle SDI | North Macedonia | 20 to 24 | 1.68 (-1.97,5.47) | -6.06 (-14.98,3.8) |
| High-middle SDI | North Macedonia | 25 to 29 | 1.51 (-1.82,4.96) | -0.49 (-5.54,4.84) |
| High-middle SDI | North Macedonia | 30 to 34 | 1.45 (-1.59,4.57) | -1.43 (-6.25,3.63) |
| High-middle SDI | North Macedonia | 35 to 39 | 1.06 (-1.67,3.86) | -1.9 (-6.42,2.85) |
| High-middle SDI | North Macedonia | 40 to 44 | 0.82 (-1.64,3.34) | -2 (-6.07,2.25) |
| High-middle SDI | North Macedonia | 45 to 49 | 0.96 (-1.23,3.2) | -1.57 (-5.04,2.03) |
| High-middle SDI | North Macedonia | 50 to 54 | 1.25 (-0.66,3.21) | -0.78 (-3.66,2.19) |
| High-middle SDI | North Macedonia | 55 to 59 | 1.45 (-0.25,3.18) | -0.24 (-2.63,2.21) |
| High-middle SDI | North Macedonia | 60 to 64 | 1.69 (0.12,3.29) | -0.07 (-2.2,2.09) |
| High-middle SDI | North Macedonia | 65 to 69 | 1.56 (-0.02,3.15) | 0.09 (-1.9,2.13) |
| High-middle SDI | North Macedonia | 70 to 74 | 1.33 (-0.44,3.13) | -0.04 (-2.09,2.06) |
| High-middle SDI | North Macedonia | 75 to 79 | 1.56 (-0.8,3.97) | 0.09 (-2.38,2.61) |
| High-middle SDI | North Macedonia | 80 to 84 | 1.23 (-1.87,4.43) | 0.19 (-2.96,3.44) |
| High-middle SDI | North Macedonia | 85 to 89 | 2.96 (-3.23,9.54) | 2.17 (-4.05,8.8) |
| High-middle SDI | Oman | 5 to 9 | 2.54 (-0.54,5.73) | -1.85 (-8.69,5.51) |
| High-middle SDI | Oman | 10 to 14 | 3.04 (0.37,5.78) | -0.73 (-6.14,5) |
| High-middle SDI | Oman | 15 to 19 | 3.25 (0.74,5.82) | -0.18 (-4.88,4.74) |
| High-middle SDI | Oman | 20 to 24 | 3.16 (0.86,5.52) | 0.19 (-3.82,4.37) |
| High-middle SDI | Oman | 25 to 29 | 2.81 (0.7,4.96) | -0.27 (-3.92,3.52) |
| High-middle SDI | Oman | 30 to 34 | 2.4 (0.39,4.44) | -0.05 (-3.41,3.44) |
| High-middle SDI | Oman | 35 to 39 | 2.02 (0.09,3.99) | -0.17 (-3.28,3.04) |
| High-middle SDI | Oman | 40 to 44 | 1.68 (-0.22,3.63) | -0.53 (-3.47,2.49) |
| High-middle SDI | Oman | 45 to 49 | 1.35 (-0.53,3.27) | -0.9 (-3.67,1.95) |
| High-middle SDI | Oman | 50 to 54 | 1.18 (-0.69,3.08) | -0.86 (-3.44,1.79) |
| High-middle SDI | Oman | 55 to 59 | 1.2 (-0.66,3.1) | -0.7 (-3.15,1.8) |
| High-middle SDI | Oman | 60 to 64 | 1.04 (-0.82,2.92) | -0.6 (-2.92,1.79) |
| High-middle SDI | Oman | 65 to 69 | 1.3 (-0.75,3.39) | -0.22 (-2.61,2.23) |
| High-middle SDI | Oman | 70 to 74 | 1.63 (-0.78,4.1) | -0.05 (-2.63,2.6) |
| High-middle SDI | Oman | 75 to 79 | 1.66 (-1.31,4.73) | 0.23 (-2.83,3.38) |
| High-middle SDI | Oman | 80 to 84 | 2.48 (-2.22,7.4) | 0.51 (-3.54,4.73) |
| High-middle SDI | Oman | 85 to 89 | 1.33 (-5.41,8.54) | 0.43 (-6.08,7.39) |
| High-middle SDI | Portugal | 5 to 9 | -1.62 (-3.64,0.44) | -3.12 (-9.52,3.73) |
| High-middle SDI | Portugal | 10 to 14 | -1.68 (-3.15,-0.19) | -4.32 (-9.01,0.62) |
| High-middle SDI | Portugal | 15 to 19 | -0.91 (-2.04,0.25) | -4.65 (-8.45,-0.68) |
| High-middle SDI | Portugal | 20 to 24 | -0.14 (-1.03,0.77) | -3.82 (-6.74,-0.8) |
| High-middle SDI | Portugal | 25 to 29 | 0.43 (-0.25,1.11) | -3.4 (-5.81,-0.93) |
| High-middle SDI | Portugal | 30 to 34 | 0.58 (0.02,1.15) | -3.03 (-4.99,-1.03) |
| High-middle SDI | Portugal | 35 to 39 | 0.79 (0.27,1.31) | -2.82 (-4.43,-1.19) |
| High-middle SDI | Portugal | 40 to 44 | 1.21 (0.73,1.7) | -2.2 (-3.51,-0.87) |
| High-middle SDI | Portugal | 45 to 49 | 1.52 (1.06,1.99) | -1.51 (-2.61,-0.39) |
| High-middle SDI | Portugal | 50 to 54 | 1.68 (1.24,2.12) | -1.05 (-1.97,-0.11) |
| High-middle SDI | Portugal | 55 to 59 | 1.53 (1.12,1.94) | -0.94 (-1.72,-0.16) |
| High-middle SDI | Portugal | 60 to 64 | 1.38 (1,1.75) | -0.91 (-1.57,-0.24) |
| High-middle SDI | Portugal | 65 to 69 | 1.46 (1.1,1.82) | -0.65 (-1.22,-0.07) |
| High-middle SDI | Portugal | 70 to 74 | 1.83 (1.46,2.21) | -0.14 (-0.67,0.4) |
| High-middle SDI | Portugal | 75 to 79 | 2.26 (1.82,2.71) | 0.48 (-0.08,1.03) |
| High-middle SDI | Portugal | 80 to 84 | 2.47 (1.9,3.04) | 0.92 (0.25,1.59) |
| High-middle SDI | Portugal | 85 to 89 | 2.11 (1.19,3.05) | 0.83 (-0.24,1.92) |
| High-middle SDI | Republic of Moldova | 5 to 9 | -0.25 (-3.05,2.64) | -1.01 (-7.51,5.95) |
| High-middle SDI | Republic of Moldova | 10 to 14 | 0.58 (-1.72,2.94) | -0.69 (-5.76,4.65) |
| High-middle SDI | Republic of Moldova | 15 to 19 | 1.01 (-1.29,3.36) | -0.97 (-5.44,3.72) |
| High-middle SDI | Republic of Moldova | 20 to 24 | 1.26 (-1,3.56) | -1.17 (-5.25,3.1) |
| High-middle SDI | Republic of Moldova | 25 to 29 | 1.85 (-0.23,3.97) | -0.51 (-4.07,3.19) |
| High-middle SDI | Republic of Moldova | 30 to 34 | 1.89 (0.01,3.81) | -0.15 (-3.3,3.1) |
| High-middle SDI | Republic of Moldova | 35 to 39 | 1.8 (0.08,3.54) | -0.09 (-2.87,2.76) |
| High-middle SDI | Republic of Moldova | 40 to 44 | 1.92 (0.38,3.48) | 0.36 (-2.02,2.8) |
| High-middle SDI | Republic of Moldova | 45 to 49 | 1.95 (0.55,3.36) | 0.6 (-1.49,2.73) |
| High-middle SDI | Republic of Moldova | 50 to 54 | 2.2 (0.95,3.46) | 0.87 (-0.92,2.69) |
| High-middle SDI | Republic of Moldova | 55 to 59 | 2.55 (1.43,3.67) | 1.14 (-0.38,2.68) |
| High-middle SDI | Republic of Moldova | 60 to 64 | 2.65 (1.59,3.73) | 1.39 (-0.01,2.8) |
| High-middle SDI | Republic of Moldova | 65 to 69 | 2.67 (1.55,3.82) | 1.53 (0.14,2.95) |
| High-middle SDI | Republic of Moldova | 70 to 74 | 2.53 (1.17,3.91) | 1.44 (-0.14,3.06) |
| High-middle SDI | Republic of Moldova | 75 to 79 | 2.11 (0.24,4.01) | 1.25 (-0.81,3.36) |
| High-middle SDI | Republic of Moldova | 80 to 84 | 1.51 (-1.1,4.18) | 0.6 (-2.09,3.37) |
| High-middle SDI | Republic of Moldova | 85 to 89 | 0.18 (-4.41,4.98) | -0.74 (-5.36,4.11) |
| High-middle SDI | Romania | 5 to 9 | -1.43 (-3.35,0.52) | -4.83 (-8.97,-0.5) |
| High-middle SDI | Romania | 10 to 14 | -0.25 (-1.68,1.2) | -3.21 (-5.88,-0.46) |
| High-middle SDI | Romania | 15 to 19 | 0.75 (-0.53,2.05) | -1.85 (-3.94,0.29) |
| High-middle SDI | Romania | 20 to 24 | 1.24 (0.04,2.45) | -1.22 (-3.03,0.62) |
| High-middle SDI | Romania | 25 to 29 | 1.57 (0.48,2.67) | -0.62 (-2.19,0.98) |
| High-middle SDI | Romania | 30 to 34 | 1.15 (0.17,2.15) | -0.77 (-2.14,0.62) |
| High-middle SDI | Romania | 35 to 39 | 0.73 (-0.16,1.63) | -0.77 (-1.97,0.44) |
| High-middle SDI | Romania | 40 to 44 | 0.82 (0.03,1.62) | -0.52 (-1.54,0.5) |
| High-middle SDI | Romania | 45 to 49 | 1.29 (0.59,1.99) | -0.01 (-0.89,0.88) |
| High-middle SDI | Romania | 50 to 54 | 1.89 (1.28,2.51) | 0.45 (-0.32,1.23) |
| High-middle SDI | Romania | 55 to 59 | 2.65 (2.12,3.19) | 1.1 (0.43,1.78) |
| High-middle SDI | Romania | 60 to 64 | 3.05 (2.58,3.52) | 1.4 (0.81,2) |
| High-middle SDI | Romania | 65 to 69 | 3.31 (2.86,3.77) | 1.66 (1.1,2.23) |
| High-middle SDI | Romania | 70 to 74 | 3.5 (2.99,4.01) | 1.91 (1.29,2.52) |
| High-middle SDI | Romania | 75 to 79 | 3.77 (3.06,4.48) | 2.25 (1.46,3.05) |
| High-middle SDI | Romania | 80 to 84 | 3.85 (2.89,4.82) | 2.46 (1.44,3.49) |
| High-middle SDI | Romania | 85 to 89 | 3.54 (1.87,5.23) | 2.27 (0.53,4.03) |
| High-middle SDI | Russian Federation | 5 to 9 | -2.53 (-3.52,-1.54) | -4.72 (-6.26,-3.15) |
| High-middle SDI | Russian Federation | 10 to 14 | -2.28 (-3.07,-1.49) | -4.44 (-5.57,-3.3) |
| High-middle SDI | Russian Federation | 15 to 19 | -1.95 (-2.66,-1.24) | -4.12 (-5.05,-3.18) |
| High-middle SDI | Russian Federation | 20 to 24 | -0.76 (-1.37,-0.14) | -2.99 (-3.75,-2.22) |
| High-middle SDI | Russian Federation | 25 to 29 | 0.61 (0.13,1.1) | -1.65 (-2.25,-1.04) |
| High-middle SDI | Russian Federation | 30 to 34 | 1.61 (1.21,2.01) | -0.6 (-1.09,-0.1) |
| High-middle SDI | Russian Federation | 35 to 39 | 2.13 (1.78,2.48) | 0.08 (-0.36,0.52) |
| High-middle SDI | Russian Federation | 40 to 44 | 2.09 (1.78,2.41) | 0.1 (-0.29,0.5) |
| High-middle SDI | Russian Federation | 45 to 49 | 1.79 (1.51,2.08) | -0.18 (-0.53,0.17) |
| High-middle SDI | Russian Federation | 50 to 54 | 1.57 (1.31,1.83) | -0.39 (-0.69,-0.08) |
| High-middle SDI | Russian Federation | 55 to 59 | 1.8 (1.57,2.03) | -0.09 (-0.35,0.16) |
| High-middle SDI | Russian Federation | 60 to 64 | 2.18 (1.97,2.4) | 0.35 (0.13,0.59) |
| High-middle SDI | Russian Federation | 65 to 69 | 2.73 (2.5,2.95) | 1 (0.77,1.22) |
| High-middle SDI | Russian Federation | 70 to 74 | 3.39 (3.1,3.68) | 1.75 (1.49,2.01) |
| High-middle SDI | Russian Federation | 75 to 79 | 4.26 (3.86,4.66) | 2.7 (2.37,3.04) |
| High-middle SDI | Russian Federation | 80 to 84 | 4.9 (4.33,5.48) | 3.36 (2.91,3.81) |
| High-middle SDI | Russian Federation | 85 to 89 | 5.46 (4.36,6.58) | 3.89 (3.04,4.74) |
| High-middle SDI | Saint Kitts and Nevis | 5 to 9 | 0.74 (-20.84,28.2) | - |
| High-middle SDI | Saint Kitts and Nevis | 10 to 14 | 0.26 (-17.12,21.28) | - |
| High-middle SDI | Saint Kitts and Nevis | 15 to 19 | -0.28 (-16.2,18.65) | - |
| High-middle SDI | Saint Kitts and Nevis | 20 to 24 | -0.65 (-16.03,17.56) | - |
| High-middle SDI | Saint Kitts and Nevis | 25 to 29 | -0.7 (-15.89,17.22) | - |
| High-middle SDI | Saint Kitts and Nevis | 30 to 34 | -1.55 (-16.26,15.74) | - |
| High-middle SDI | Saint Kitts and Nevis | 35 to 39 | -2.21 (-16.7,14.79) | - |
| High-middle SDI | Saint Kitts and Nevis | 40 to 44 | -3.1 (-17.24,13.45) | - |
| High-middle SDI | Saint Kitts and Nevis | 45 to 49 | -0.61 (-14.81,15.97) | - |
| High-middle SDI | Saint Kitts and Nevis | 50 to 54 | 1.59 (-11.92,17.17) | - |
| High-middle SDI | Saint Kitts and Nevis | 55 to 59 | 4.29 (-8.22,18.51) | - |
| High-middle SDI | Saint Kitts and Nevis | 60 to 64 | 5.01 (-7.55,19.29) | - |
| High-middle SDI | Saint Kitts and Nevis | 65 to 69 | 4.38 (-9.5,20.38) | - |
| High-middle SDI | Saint Kitts and Nevis | 70 to 74 | 1.54 (-14.04,19.95) | - |
| High-middle SDI | Saint Kitts and Nevis | 75 to 79 | 1.48 (-14.67,20.68) | - |
| High-middle SDI | Saint Kitts and Nevis | 80 to 84 | 0.72 (-16.73,21.82) | - |
| High-middle SDI | Saint Kitts and Nevis | 85 to 89 | 0 (-21.48,27.34) | - |
| High-middle SDI | Serbia | 5 to 9 | -3.11 (-6.26,0.14) | -3.63 (-9.85,3.01) |
| High-middle SDI | Serbia | 10 to 14 | -2.39 (-4.67,-0.05) | -4.02 (-8.73,0.93) |
| High-middle SDI | Serbia | 15 to 19 | -1.52 (-3.54,0.55) | -3.47 (-7.45,0.68) |
| High-middle SDI | Serbia | 20 to 24 | -0.64 (-2.46,1.21) | -2.36 (-5.59,0.98) |
| High-middle SDI | Serbia | 25 to 29 | -0.15 (-1.73,1.46) | -1.87 (-4.59,0.93) |
| High-middle SDI | Serbia | 30 to 34 | 0.25 (-1.12,1.63) | -1.91 (-4.3,0.54) |
| High-middle SDI | Serbia | 35 to 39 | 0.3 (-0.89,1.5) | -1.53 (-3.55,0.53) |
| High-middle SDI | Serbia | 40 to 44 | 0.39 (-0.64,1.43) | -1.56 (-3.27,0.18) |
| High-middle SDI | Serbia | 45 to 49 | 0.81 (-0.09,1.72) | -0.93 (-2.35,0.51) |
| High-middle SDI | Serbia | 50 to 54 | 1.53 (0.74,2.32) | -0.07 (-1.23,1.11) |
| High-middle SDI | Serbia | 55 to 59 | 1.8 (1.12,2.48) | 0.34 (-0.6,1.29) |
| High-middle SDI | Serbia | 60 to 64 | 1.8 (1.19,2.41) | 0.51 (-0.28,1.31) |
| High-middle SDI | Serbia | 65 to 69 | 1.73 (1.13,2.33) | 0.62 (-0.11,1.35) |
| High-middle SDI | Serbia | 70 to 74 | 1.73 (1.05,2.41) | 0.78 (0.01,1.55) |
| High-middle SDI | Serbia | 75 to 79 | 1.84 (0.92,2.77) | 1.02 (0.07,1.99) |
| High-middle SDI | Serbia | 80 to 84 | 1.38 (-0.19,2.99) | 0.74 (-0.81,2.31) |
| High-middle SDI | Serbia | 85 to 89 | 0.53 (-1.68,2.79) | -0.06 (-2.19,2.1) |
| High-middle SDI | Seychelles | 5 to 9 | 1.42 (-20.2,28.9) | 0.14 (-21.26,27.36) |
| High-middle SDI | Seychelles | 10 to 14 | 0.85 (-16.61,21.95) | -0.03 (-17.35,20.92) |
| High-middle SDI | Seychelles | 15 to 19 | 0.4 (-15.6,19.44) | 0.3 (-15.7,19.35) |
| High-middle SDI | Seychelles | 20 to 24 | -0.79 (-15.89,17.02) | 0.26 (-15.26,18.61) |
| High-middle SDI | Seychelles | 25 to 29 | -0.96 (-15.74,16.4) | -1.49 (-16.39,16.08) |
| High-middle SDI | Seychelles | 30 to 34 | -0.45 (-14.66,16.12) | -1.16 (-15.9,16.16) |
| High-middle SDI | Seychelles | 35 to 39 | 0.82 (-12.59,16.3) | -0.57 (-15.01,16.32) |
| High-middle SDI | Seychelles | 40 to 44 | 2.39 (-9.93,16.39) | 0.53 (-13.28,16.53) |
| High-middle SDI | Seychelles | 45 to 49 | 4.33 (-6.74,16.7) | 3.11 (-9.88,17.96) |
| High-middle SDI | Seychelles | 50 to 54 | 3.91 (-6.29,15.23) | 6.99 (-5.41,21.02) |
| High-middle SDI | Seychelles | 55 to 59 | 4.37 (-5.46,15.23) | 6.12 (-6.16,20.02) |
| High-middle SDI | Seychelles | 60 to 64 | 5.23 (-4.84,16.36) | 5.7 (-5.28,17.96) |
| High-middle SDI | Seychelles | 65 to 69 | 8.24 (-3.44,21.34) | 6.3 (-5.84,20.01) |
| High-middle SDI | Seychelles | 70 to 74 | 8.87 (-4.24,23.77) | 4.58 (-8.92,20.08) |
| High-middle SDI | Seychelles | 75 to 79 | 6.97 (-7.66,23.92) | 1.83 (-13.4,19.74) |
| High-middle SDI | Seychelles | 80 to 84 | 1.67 (-15.2,21.91) | 0.68 (-16.48,21.36) |
| High-middle SDI | Seychelles | 85 to 89 | -1.82 (-22.7,24.69) | -0.98 (-22.17,25.96) |
| High-middle SDI | Spain | 5 to 9 | -0.38 (-1.6,0.85) | -3.44 (-7.12,0.39) |
| High-middle SDI | Spain | 10 to 14 | -0.17 (-1.12,0.79) | -3.03 (-5.5,-0.49) |
| High-middle SDI | Spain | 15 to 19 | -0.12 (-0.95,0.72) | -2.87 (-4.82,-0.88) |
| High-middle SDI | Spain | 20 to 24 | -0.82 (-1.54,-0.09) | -3 (-4.59,-1.37) |
| High-middle SDI | Spain | 25 to 29 | -1.75 (-2.35,-1.15) | -3.49 (-4.8,-2.17) |
| High-middle SDI | Spain | 30 to 34 | -2.32 (-2.8,-1.83) | -3.85 (-4.9,-2.78) |
| High-middle SDI | Spain | 35 to 39 | -2.33 (-2.73,-1.92) | -3.9 (-4.74,-3.05) |
| High-middle SDI | Spain | 40 to 44 | -1.88 (-2.23,-1.53) | -3.59 (-4.28,-2.89) |
| High-middle SDI | Spain | 45 to 49 | -0.97 (-1.27,-0.66) | -2.85 (-3.43,-2.26) |
| High-middle SDI | Spain | 50 to 54 | -0.27 (-0.54,0) | -2.23 (-2.71,-1.74) |
| High-middle SDI | Spain | 55 to 59 | -0.01 (-0.26,0.24) | -1.89 (-2.31,-1.48) |
| High-middle SDI | Spain | 60 to 64 | -0.02 (-0.25,0.21) | -1.76 (-2.11,-1.4) |
| High-middle SDI | Spain | 65 to 69 | -0.05 (-0.27,0.17) | -1.59 (-1.9,-1.29) |
| High-middle SDI | Spain | 70 to 74 | 0.11 (-0.11,0.32) | -1.25 (-1.53,-0.97) |
| High-middle SDI | Spain | 75 to 79 | 0.33 (0.09,0.58) | -0.67 (-0.95,-0.39) |
| High-middle SDI | Spain | 80 to 84 | 0.63 (0.31,0.94) | 0.02 (-0.3,0.34) |
| High-middle SDI | Spain | 85 to 89 | 1.06 (0.53,1.59) | 0.85 (0.33,1.36) |
| High-middle SDI | Trinidad and Tobago | 5 to 9 | 1.5 (-4.08,7.4) | -2.9 (-12.55,7.81) |
| High-middle SDI | Trinidad and Tobago | 10 to 14 | 1.14 (-3.44,5.92) | -5.72 (-17.55,7.8) |
| High-middle SDI | Trinidad and Tobago | 15 to 19 | 1.09 (-2.83,5.17) | 0.08 (-6.28,6.86) |
| High-middle SDI | Trinidad and Tobago | 20 to 24 | 1.38 (-1.88,4.76) | 1.14 (-3.56,6.08) |
| High-middle SDI | Trinidad and Tobago | 25 to 29 | 1.32 (-1.46,4.18) | 0.44 (-3.62,4.67) |
| High-middle SDI | Trinidad and Tobago | 30 to 34 | 0.93 (-1.56,3.47) | 0.25 (-3.35,3.99) |
| High-middle SDI | Trinidad and Tobago | 35 to 39 | 1.15 (-1.18,3.53) | 0.05 (-3.23,3.45) |
| High-middle SDI | Trinidad and Tobago | 40 to 44 | 0.77 (-1.49,3.07) | -0.12 (-3.21,3.07) |
| High-middle SDI | Trinidad and Tobago | 45 to 49 | 0.56 (-1.53,2.7) | -0.38 (-3.22,2.55) |
| High-middle SDI | Trinidad and Tobago | 50 to 54 | 0.13 (-1.83,2.13) | -0.31 (-2.88,2.33) |
| High-middle SDI | Trinidad and Tobago | 55 to 59 | -0.14 (-2,1.75) | -0.75 (-3.12,1.68) |
| High-middle SDI | Trinidad and Tobago | 60 to 64 | -0.1 (-1.97,1.8) | -0.88 (-3.16,1.44) |
| High-middle SDI | Trinidad and Tobago | 65 to 69 | -0.33 (-2.27,1.66) | -1.09 (-3.37,1.25) |
| High-middle SDI | Trinidad and Tobago | 70 to 74 | -0.52 (-2.62,1.63) | -0.98 (-3.37,1.47) |
| High-middle SDI | Trinidad and Tobago | 75 to 79 | -0.35 (-2.98,2.35) | -0.62 (-3.48,2.33) |
| High-middle SDI | Trinidad and Tobago | 80 to 84 | -0.42 (-3.98,3.26) | -0.62 (-4.26,3.16) |
| High-middle SDI | Trinidad and Tobago | 85 to 89 | 0.33 (-5.98,7.07) | -0.13 (-6.5,6.68) |
| High-middle SDI | Ukraine | 5 to 9 | -0.36 (-1.82,1.11) | -0.89 (-3.47,1.76) |
| High-middle SDI | Ukraine | 10 to 14 | -0.71 (-1.83,0.42) | -1.16 (-3.05,0.77) |
| High-middle SDI | Ukraine | 15 to 19 | -0.64 (-1.59,0.33) | -1.21 (-2.75,0.36) |
| High-middle SDI | Ukraine | 20 to 24 | -0.36 (-1.15,0.43) | -0.93 (-2.15,0.31) |
| High-middle SDI | Ukraine | 25 to 29 | 0.19 (-0.44,0.82) | -0.45 (-1.42,0.53) |
| High-middle SDI | Ukraine | 30 to 34 | 0.8 (0.27,1.34) | 0.24 (-0.56,1.06) |
| High-middle SDI | Ukraine | 35 to 39 | 1.38 (0.89,1.87) | 0.91 (0.19,1.63) |
| High-middle SDI | Ukraine | 40 to 44 | 1.51 (1.06,1.97) | 1.09 (0.43,1.74) |
| High-middle SDI | Ukraine | 45 to 49 | 1.45 (1.02,1.87) | 0.98 (0.39,1.57) |
| High-middle SDI | Ukraine | 50 to 54 | 1.22 (0.84,1.6) | 0.7 (0.19,1.22) |
| High-middle SDI | Ukraine | 55 to 59 | 1.28 (0.95,1.61) | 0.72 (0.28,1.16) |
| High-middle SDI | Ukraine | 60 to 64 | 1.56 (1.24,1.88) | 0.98 (0.57,1.39) |
| High-middle SDI | Ukraine | 65 to 69 | 1.99 (1.66,2.32) | 1.4 (0.99,1.81) |
| High-middle SDI | Ukraine | 70 to 74 | 2.39 (1.98,2.8) | 1.75 (1.27,2.24) |
| High-middle SDI | Ukraine | 75 to 79 | 2.47 (1.89,3.05) | 1.78 (1.14,2.43) |
| High-middle SDI | Ukraine | 80 to 84 | 1.77 (0.96,2.58) | 1.13 (0.27,1.99) |
| High-middle SDI | Ukraine | 85 to 89 | 1.86 (0.28,3.46) | 1.25 (-0.39,2.91) |
| Low SDI | Afghanistan | 5 to 9 | -0.43 (-1.98,1.14) | -1.31 (-3.14,0.56) |
| Low SDI | Afghanistan | 10 to 14 | 0.15 (-1.23,1.55) | -0.72 (-2.3,0.89) |
| Low SDI | Afghanistan | 15 to 19 | 0.44 (-0.83,1.73) | -0.29 (-1.73,1.17) |
| Low SDI | Afghanistan | 20 to 24 | 0.63 (-0.59,1.86) | 0.01 (-1.37,1.42) |
| Low SDI | Afghanistan | 25 to 29 | 0.78 (-0.46,2.03) | 0.22 (-1.2,1.67) |
| Low SDI | Afghanistan | 30 to 34 | 0.99 (-0.36,2.36) | 0.59 (-0.97,2.18) |
| Low SDI | Afghanistan | 35 to 39 | 0.94 (-0.43,2.34) | 0.47 (-1.08,2.05) |
| Low SDI | Afghanistan | 40 to 44 | 0.67 (-0.57,1.91) | 0.15 (-1.23,1.55) |
| Low SDI | Afghanistan | 45 to 49 | 0.14 (-0.97,1.26) | -0.39 (-1.6,0.84) |
| Low SDI | Afghanistan | 50 to 54 | -0.36 (-1.43,0.72) | -0.81 (-1.97,0.36) |
| Low SDI | Afghanistan | 55 to 59 | -0.54 (-1.63,0.57) | -0.95 (-2.12,0.23) |
| Low SDI | Afghanistan | 60 to 64 | -0.4 (-1.45,0.66) | -0.66 (-1.75,0.44) |
| Low SDI | Afghanistan | 65 to 69 | -0.24 (-1.25,0.77) | -0.5 (-1.52,0.52) |
| Low SDI | Afghanistan | 70 to 74 | -0.16 (-1.29,0.99) | -0.34 (-1.44,0.78) |
| Low SDI | Afghanistan | 75 to 79 | -0.02 (-1.52,1.5) | -0.23 (-1.63,1.19) |
| Low SDI | Afghanistan | 80 to 84 | 0.12 (-2.17,2.46) | -0.16 (-2.2,1.91) |
| Low SDI | Afghanistan | 85 to 89 | 0.73 (-3.76,5.43) | 0.23 (-3.52,4.12) |
| Low SDI | Angola | 5 to 9 | -1.79 (-3.66,0.12) | -1.85 (-3.85,0.18) |
| Low SDI | Angola | 10 to 14 | -0.45 (-2.08,1.21) | -0.74 (-2.5,1.06) |
| Low SDI | Angola | 15 to 19 | 0.59 (-0.99,2.19) | 0.02 (-1.69,1.77) |
| Low SDI | Angola | 20 to 24 | 1.14 (-0.4,2.71) | 0.4 (-1.32,2.15) |
| Low SDI | Angola | 25 to 29 | 1.43 (-0.05,2.93) | 0.67 (-1.02,2.39) |
| Low SDI | Angola | 30 to 34 | 1.53 (0.13,2.96) | 0.79 (-0.82,2.42) |
| Low SDI | Angola | 35 to 39 | 1.31 (-0.02,2.65) | 0.6 (-0.91,2.13) |
| Low SDI | Angola | 40 to 44 | 1.03 (-0.22,2.29) | 0.41 (-0.98,1.82) |
| Low SDI | Angola | 45 to 49 | 0.75 (-0.44,1.96) | 0.18 (-1.13,1.5) |
| Low SDI | Angola | 50 to 54 | 0.4 (-0.72,1.55) | -0.12 (-1.32,1.1) |
| Low SDI | Angola | 55 to 59 | 0.13 (-0.97,1.25) | -0.32 (-1.46,0.84) |
| Low SDI | Angola | 60 to 64 | -0.02 (-1.15,1.13) | -0.34 (-1.5,0.83) |
| Low SDI | Angola | 65 to 69 | -0.03 (-1.28,1.23) | -0.3 (-1.54,0.95) |
| Low SDI | Angola | 70 to 74 | -0.08 (-1.59,1.45) | -0.15 (-1.62,1.35) |
| Low SDI | Angola | 75 to 79 | 0.2 (-1.94,2.4) | 0.18 (-1.81,2.21) |
| Low SDI | Angola | 80 to 84 | 0.17 (-3.11,3.56) | 0.82 (-2.39,4.15) |
| Low SDI | Angola | 85 to 89 | -0.08 (-6.22,6.46) | 0.96 (-5.15,7.47) |
| Low SDI | Benin | 5 to 9 | 0.42 (-1.47,2.34) | 0.09 (-1.99,2.21) |
| Low SDI | Benin | 10 to 14 | 0.92 (-1.04,2.92) | 0.49 (-1.7,2.73) |
| Low SDI | Benin | 15 to 19 | 1.05 (-1.15,3.3) | 0.65 (-1.85,3.2) |
| Low SDI | Benin | 20 to 24 | 1.15 (-1.19,3.54) | 0.66 (-1.99,3.39) |
| Low SDI | Benin | 25 to 29 | 1.08 (-1.25,3.46) | 0.61 (-2.09,3.38) |
| Low SDI | Benin | 30 to 34 | 1.05 (-1.24,3.39) | 0.69 (-1.99,3.44) |
| Low SDI | Benin | 35 to 39 | 0.79 (-1.44,3.07) | 0.69 (-1.93,3.37) |
| Low SDI | Benin | 40 to 44 | 0.75 (-1.43,2.99) | 0.8 (-1.73,3.4) |
| Low SDI | Benin | 45 to 49 | 0.65 (-1.49,2.84) | 0.85 (-1.59,3.35) |
| Low SDI | Benin | 50 to 54 | 0.67 (-1.38,2.76) | 0.72 (-1.55,3.04) |
| Low SDI | Benin | 55 to 59 | 0.58 (-1.38,2.59) | 0.46 (-1.64,2.61) |
| Low SDI | Benin | 60 to 64 | 0.41 (-1.49,2.36) | 0.3 (-1.69,2.33) |
| Low SDI | Benin | 65 to 69 | 0.18 (-1.78,2.19) | 0.08 (-1.91,2.11) |
| Low SDI | Benin | 70 to 74 | 0.15 (-2.05,2.4) | -0.13 (-2.27,2.06) |
| Low SDI | Benin | 75 to 79 | -0.19 (-2.74,2.42) | -0.02 (-2.53,2.56) |
| Low SDI | Benin | 80 to 84 | 0.1 (-3.76,4.12) | -0.13 (-3.57,3.44) |
| Low SDI | Benin | 85 to 89 | -0.62 (-7.1,6.31) | -0.48 (-6.77,6.22) |
| Low SDI | Bhutan | 5 to 9 | 1.38 (-6.3,9.7) | -7.49 (-24.56,13.43) |
| Low SDI | Bhutan | 10 to 14 | 2.19 (-5.43,10.42) | -4.42 (-19.59,13.61) |
| Low SDI | Bhutan | 15 to 19 | 2.2 (-5.91,11.02) | -2.76 (-17.63,14.8) |
| Low SDI | Bhutan | 20 to 24 | 2.01 (-6.17,10.89) | -3.66 (-18.15,13.41) |
| Low SDI | Bhutan | 25 to 29 | 1.47 (-6.53,10.16) | -0.34 (-14.98,16.82) |
| Low SDI | Bhutan | 30 to 34 | 0.58 (-6.73,8.47) | 0.16 (-14.06,16.73) |
| Low SDI | Bhutan | 35 to 39 | -0.85 (-7.43,6.2) | 0.26 (-13.27,15.9) |
| Low SDI | Bhutan | 40 to 44 | -1.44 (-7.17,4.65) | 0.53 (-11.23,13.84) |
| Low SDI | Bhutan | 45 to 49 | -1.95 (-7.19,3.58) | 0.79 (-8.19,10.65) |
| Low SDI | Bhutan | 50 to 54 | -1.74 (-6.74,3.53) | -2.5 (-9.16,4.64) |
| Low SDI | Bhutan | 55 to 59 | -0.5 (-5.31,4.56) | -1.73 (-7.28,4.15) |
| Low SDI | Bhutan | 60 to 64 | 0.19 (-4.48,5.08) | -1.09 (-6.14,4.23) |
| Low SDI | Bhutan | 65 to 69 | 0.22 (-4.5,5.17) | -1.02 (-5.84,4.05) |
| Low SDI | Bhutan | 70 to 74 | -0.86 (-5.8,4.34) | -1.61 (-6.6,3.64) |
| Low SDI | Bhutan | 75 to 79 | 3.27 (-7.62,15.45) | 3.41 (-7.48,15.58) |
| Low SDI | Bhutan | 80 to 84 | 4.5 (-9.82,21.08) | 5.2 (-9.08,21.72) |
| Low SDI | Bhutan | 85 to 89 | 4.1 (-15.25,27.89) | 5.46 (-13.75,28.95) |
| Low SDI | Burkina Faso | 5 to 9 | 1.13 (-0.33,2.61) | 0.88 (-0.7,2.48) |
| Low SDI | Burkina Faso | 10 to 14 | 1.61 (0.11,3.13) | 1.31 (-0.35,2.99) |
| Low SDI | Burkina Faso | 15 to 19 | 1.82 (0.13,3.54) | 1.55 (-0.38,3.51) |
| Low SDI | Burkina Faso | 20 to 24 | 1.87 (0.06,3.71) | 1.6 (-0.49,3.73) |
| Low SDI | Burkina Faso | 25 to 29 | 1.86 (0.04,3.7) | 1.46 (-0.66,3.63) |
| Low SDI | Burkina Faso | 30 to 34 | 1.73 (-0.02,3.51) | 1.33 (-0.73,3.44) |
| Low SDI | Burkina Faso | 35 to 39 | 1.48 (-0.2,3.18) | 1.1 (-0.85,3.08) |
| Low SDI | Burkina Faso | 40 to 44 | 1.33 (-0.27,2.96) | 0.96 (-0.88,2.83) |
| Low SDI | Burkina Faso | 45 to 49 | 1.24 (-0.29,2.79) | 0.9 (-0.82,2.64) |
| Low SDI | Burkina Faso | 50 to 54 | 1 (-0.43,2.45) | 0.83 (-0.72,2.41) |
| Low SDI | Burkina Faso | 55 to 59 | 0.85 (-0.49,2.21) | 0.68 (-0.75,2.13) |
| Low SDI | Burkina Faso | 60 to 64 | 0.61 (-0.68,1.91) | 0.47 (-0.87,1.83) |
| Low SDI | Burkina Faso | 65 to 69 | 0.43 (-0.89,1.77) | 0.25 (-1.08,1.61) |
| Low SDI | Burkina Faso | 70 to 74 | 0.23 (-1.26,1.75) | 0.05 (-1.4,1.52) |
| Low SDI | Burkina Faso | 75 to 79 | -0.01 (-1.8,1.81) | 0.06 (-1.65,1.79) |
| Low SDI | Burkina Faso | 80 to 84 | -0.25 (-2.87,2.44) | 0.12 (-2.32,2.63) |
| Low SDI | Burkina Faso | 85 to 89 | 0.35 (-5.69,6.77) | 1.42 (-4.57,7.78) |
| Low SDI | Burundi | 5 to 9 | -2.07 (-3.68,-0.43) | -2.23 (-3.94,-0.49) |
| Low SDI | Burundi | 10 to 14 | -1.41 (-2.85,0.06) | -1.76 (-3.3,-0.19) |
| Low SDI | Burundi | 15 to 19 | -1.16 (-2.64,0.34) | -1.6 (-3.2,0.03) |
| Low SDI | Burundi | 20 to 24 | -0.93 (-2.46,0.62) | -1.52 (-3.19,0.19) |
| Low SDI | Burundi | 25 to 29 | -0.85 (-2.36,0.68) | -1.4 (-3.08,0.32) |
| Low SDI | Burundi | 30 to 34 | -0.84 (-2.3,0.65) | -1.31 (-2.96,0.38) |
| Low SDI | Burundi | 35 to 39 | -0.94 (-2.37,0.5) | -1.27 (-2.88,0.36) |
| Low SDI | Burundi | 40 to 44 | -1.24 (-2.62,0.16) | -1.53 (-3.07,0.04) |
| Low SDI | Burundi | 45 to 49 | -1.61 (-2.95,-0.25) | -1.74 (-3.21,-0.25) |
| Low SDI | Burundi | 50 to 54 | -1.77 (-3.05,-0.48) | -1.95 (-3.32,-0.56) |
| Low SDI | Burundi | 55 to 59 | -1.85 (-3.06,-0.63) | -1.94 (-3.22,-0.65) |
| Low SDI | Burundi | 60 to 64 | -1.81 (-3,-0.61) | -1.88 (-3.1,-0.65) |
| Low SDI | Burundi | 65 to 69 | -1.61 (-2.85,-0.36) | -1.76 (-3,-0.5) |
| Low SDI | Burundi | 70 to 74 | -1.42 (-2.82,0) | -1.56 (-2.93,-0.18) |
| Low SDI | Burundi | 75 to 79 | -1.2 (-2.91,0.54) | -1.3 (-2.9,0.33) |
| Low SDI | Burundi | 80 to 84 | -0.71 (-3.09,1.72) | -0.97 (-3.08,1.19) |
| Low SDI | Burundi | 85 to 89 | -0.31 (-4.8,4.4) | -0.67 (-4.42,3.24) |
| Low SDI | Cambodia | 5 to 9 | -0.44 (-2.75,1.92) | -1.74 (-4.57,1.17) |
| Low SDI | Cambodia | 10 to 14 | 0.02 (-1.79,1.86) | -1.35 (-3.54,0.88) |
| Low SDI | Cambodia | 15 to 19 | 0.54 (-1.02,2.13) | -0.5 (-2.3,1.32) |
| Low SDI | Cambodia | 20 to 24 | 0.74 (-0.72,2.21) | -0.3 (-1.96,1.39) |
| Low SDI | Cambodia | 25 to 29 | 0.93 (-0.45,2.34) | -0.03 (-1.61,1.59) |
| Low SDI | Cambodia | 30 to 34 | 1.02 (-0.34,2.39) | 0.14 (-1.42,1.72) |
| Low SDI | Cambodia | 35 to 39 | 1.05 (-0.28,2.39) | 0.18 (-1.33,1.71) |
| Low SDI | Cambodia | 40 to 44 | 1 (-0.28,2.29) | 0.12 (-1.31,1.56) |
| Low SDI | Cambodia | 45 to 49 | 0.87 (-0.32,2.07) | 0.14 (-1.16,1.46) |
| Low SDI | Cambodia | 50 to 54 | 0.68 (-0.42,1.79) | 0.11 (-1.06,1.29) |
| Low SDI | Cambodia | 55 to 59 | 0.62 (-0.42,1.67) | 0.09 (-0.99,1.18) |
| Low SDI | Cambodia | 60 to 64 | 0.6 (-0.42,1.63) | 0.17 (-0.87,1.23) |
| Low SDI | Cambodia | 65 to 69 | 0.82 (-0.26,1.92) | 0.47 (-0.61,1.56) |
| Low SDI | Cambodia | 70 to 74 | 1.03 (-0.25,2.33) | 0.72 (-0.51,1.97) |
| Low SDI | Cambodia | 75 to 79 | 1.34 (-0.44,3.15) | 0.96 (-0.66,2.6) |
| Low SDI | Cambodia | 80 to 84 | 1.35 (-1.35,4.12) | 1.31 (-1.18,3.86) |
| Low SDI | Cambodia | 85 to 89 | 2.46 (-3.57,8.87) | 1.25 (-3.18,5.88) |
| Low SDI | Cameroon | 5 to 9 | 0.99 (-0.17,2.15) | 0.65 (-0.62,1.94) |
| Low SDI | Cameroon | 10 to 14 | 1.33 (0.2,2.47) | 0.9 (-0.36,2.18) |
| Low SDI | Cameroon | 15 to 19 | 1.55 (0.33,2.78) | 1.05 (-0.34,2.46) |
| Low SDI | Cameroon | 20 to 24 | 1.72 (0.46,3.01) | 1.08 (-0.39,2.57) |
| Low SDI | Cameroon | 25 to 29 | 1.78 (0.52,3.04) | 1.1 (-0.38,2.59) |
| Low SDI | Cameroon | 30 to 34 | 1.75 (0.53,2.99) | 1.17 (-0.28,2.63) |
| Low SDI | Cameroon | 35 to 39 | 1.57 (0.39,2.76) | 1.07 (-0.31,2.48) |
| Low SDI | Cameroon | 40 to 44 | 1.41 (0.26,2.57) | 0.92 (-0.4,2.26) |
| Low SDI | Cameroon | 45 to 49 | 1.17 (0.06,2.31) | 0.76 (-0.49,2.03) |
| Low SDI | Cameroon | 50 to 54 | 1 (-0.07,2.08) | 0.59 (-0.58,1.77) |
| Low SDI | Cameroon | 55 to 59 | 0.84 (-0.18,1.88) | 0.52 (-0.58,1.63) |
| Low SDI | Cameroon | 60 to 64 | 0.67 (-0.34,1.69) | 0.42 (-0.63,1.49) |
| Low SDI | Cameroon | 65 to 69 | 0.47 (-0.61,1.55) | 0.28 (-0.81,1.38) |
| Low SDI | Cameroon | 70 to 74 | 0.29 (-1.01,1.6) | 0.13 (-1.14,1.42) |
| Low SDI | Cameroon | 75 to 79 | 0.05 (-1.6,1.72) | -0.05 (-1.6,1.53) |
| Low SDI | Cameroon | 80 to 84 | -0.14 (-2.64,2.43) | -0.03 (-2.37,2.37) |
| Low SDI | Cameroon | 85 to 89 | -0.69 (-5.21,4.03) | -0.04 (-4.47,4.59) |
| Low SDI | Central African Republic | 5 to 9 | -0.57 (-4.53,3.57) | -0.43 (-4.43,3.73) |
| Low SDI | Central African Republic | 10 to 14 | -0.44 (-3.76,2.99) | -0.16 (-3.59,3.38) |
| Low SDI | Central African Republic | 15 to 19 | 0.13 (-2.93,3.28) | 0.06 (-3.16,3.38) |
| Low SDI | Central African Republic | 20 to 24 | 0.16 (-2.77,3.17) | -0.11 (-3.29,3.18) |
| Low SDI | Central African Republic | 25 to 29 | 0.18 (-2.63,3.06) | 0.04 (-2.99,3.17) |
| Low SDI | Central African Republic | 30 to 34 | 0.25 (-2.32,2.89) | 0.2 (-2.61,3.1) |
| Low SDI | Central African Republic | 35 to 39 | 0.21 (-2.15,2.61) | -0.08 (-2.66,2.56) |
| Low SDI | Central African Republic | 40 to 44 | -0.06 (-2.22,2.16) | -0.08 (-2.45,2.35) |
| Low SDI | Central African Republic | 45 to 49 | 0.14 (-1.87,2.19) | -0.14 (-2.32,2.09) |
| Low SDI | Central African Republic | 50 to 54 | -0.16 (-2.08,1.8) | -0.19 (-2.23,1.89) |
| Low SDI | Central African Republic | 55 to 59 | -0.3 (-2.2,1.64) | -0.34 (-2.32,1.67) |
| Low SDI | Central African Republic | 60 to 64 | -0.31 (-2.32,1.73) | -0.37 (-2.42,1.72) |
| Low SDI | Central African Republic | 65 to 69 | -0.42 (-2.7,1.91) | -0.41 (-2.68,1.91) |
| Low SDI | Central African Republic | 70 to 74 | -0.53 (-3.39,2.42) | 0.05 (-2.78,2.96) |
| Low SDI | Central African Republic | 75 to 79 | -0.5 (-4.54,3.7) | 0.51 (-3.47,4.65) |
| Low SDI | Central African Republic | 80 to 84 | -1.27 (-7.68,5.59) | 0.46 (-5.83,7.17) |
| Low SDI | Central African Republic | 85 to 89 | -2.46 (-20.23,19.26) | 2.94 (-14.71,24.24) |
| Low SDI | Chad | 5 to 9 | 1.39 (-0.41,3.22) | 1.4 (-0.54,3.38) |
| Low SDI | Chad | 10 to 14 | 1.54 (-0.4,3.52) | 1.42 (-0.7,3.59) |
| Low SDI | Chad | 15 to 19 | 1.55 (-0.71,3.86) | 1.29 (-1.18,3.83) |
| Low SDI | Chad | 20 to 24 | 1.49 (-0.96,4) | 1.45 (-1.34,4.32) |
| Low SDI | Chad | 25 to 29 | 1.62 (-0.89,4.2) | 1.61 (-1.3,4.6) |
| Low SDI | Chad | 30 to 34 | 1.65 (-0.82,4.18) | 1.49 (-1.34,4.39) |
| Low SDI | Chad | 35 to 39 | 1.4 (-0.97,3.83) | 1.36 (-1.3,4.09) |
| Low SDI | Chad | 40 to 44 | 1.45 (-0.8,3.75) | 1.2 (-1.28,3.74) |
| Low SDI | Chad | 45 to 49 | 1.37 (-0.76,3.55) | 0.83 (-1.47,3.19) |
| Low SDI | Chad | 50 to 54 | 1.16 (-0.83,3.2) | 0.93 (-1.17,3.07) |
| Low SDI | Chad | 55 to 59 | 0.97 (-0.9,2.88) | 0.9 (-1.06,2.9) |
| Low SDI | Chad | 60 to 64 | 0.86 (-0.94,2.69) | 0.79 (-1.07,2.69) |
| Low SDI | Chad | 65 to 69 | 0.64 (-1.24,2.55) | 0.63 (-1.25,2.56) |
| Low SDI | Chad | 70 to 74 | 0.44 (-1.65,2.58) | 0.47 (-1.59,2.58) |
| Low SDI | Chad | 75 to 79 | 0.3 (-2.26,2.92) | 0.25 (-2.2,2.76) |
| Low SDI | Chad | 80 to 84 | 0.38 (-3.58,4.5) | -0.38 (-3.91,3.28) |
| Low SDI | Chad | 85 to 89 | -0.72 (-7.36,6.4) | -1.29 (-7.77,5.64) |
| Low SDI | Comoros | 5 to 9 | -0.58 (-7.91,7.34) | -0.43 (-7.79,7.52) |
| Low SDI | Comoros | 10 to 14 | -1 (-6.79,5.15) | -0.91 (-6.75,5.3) |
| Low SDI | Comoros | 15 to 19 | -1.13 (-6.42,4.46) | -1.26 (-6.67,4.46) |
| Low SDI | Comoros | 20 to 24 | -1.22 (-6.33,4.16) | -1.97 (-7.44,3.83) |
| Low SDI | Comoros | 25 to 29 | -1.57 (-6.62,3.75) | -2.54 (-7.77,2.99) |
| Low SDI | Comoros | 30 to 34 | -1.3 (-6.06,3.69) | -2.51 (-7.96,3.26) |
| Low SDI | Comoros | 35 to 39 | -0.54 (-5.08,4.21) | -2.2 (-7.36,3.25) |
| Low SDI | Comoros | 40 to 44 | -0.62 (-5.08,4.05) | -2.19 (-7.13,3.02) |
| Low SDI | Comoros | 45 to 49 | -0.76 (-5.08,3.76) | -2.1 (-6.77,2.81) |
| Low SDI | Comoros | 50 to 54 | -0.09 (-4.26,4.26) | -1.3 (-5.72,3.32) |
| Low SDI | Comoros | 55 to 59 | -0.06 (-4.04,4.09) | -1.17 (-5.42,3.26) |
| Low SDI | Comoros | 60 to 64 | -0.61 (-4.37,3.29) | -0.42 (-4.46,3.79) |
| Low SDI | Comoros | 65 to 69 | -0.71 (-4.43,3.16) | -0.65 (-4.48,3.34) |
| Low SDI | Comoros | 70 to 74 | -0.33 (-4.7,4.24) | 0.09 (-4.3,4.67) |
| Low SDI | Comoros | 75 to 79 | -1.02 (-6.22,4.47) | -0.29 (-5.42,5.12) |
| Low SDI | Comoros | 80 to 84 | 3.4 (-9.53,18.19) | -0.81 (-7.39,6.25) |
| Low SDI | Comoros | 85 to 89 | 4.3 (-14.91,27.85) | 2.11 (-15.22,22.98) |
| Low SDI | Coted'Ivoire | 5 to 9 | 1.17 (0.06,2.29) | 0.6 (-0.65,1.87) |
| Low SDI | Coted'Ivoire | 10 to 14 | 1.3 (0.24,2.37) | 0.71 (-0.5,1.94) |
| Low SDI | Coted'Ivoire | 15 to 19 | 1.43 (0.31,2.57) | 0.75 (-0.55,2.06) |
| Low SDI | Coted'Ivoire | 20 to 24 | 1.55 (0.4,2.72) | 0.78 (-0.56,2.15) |
| Low SDI | Coted'Ivoire | 25 to 29 | 1.57 (0.42,2.73) | 0.75 (-0.6,2.12) |
| Low SDI | Coted'Ivoire | 30 to 34 | 1.3 (0.19,2.43) | 0.55 (-0.77,1.9) |
| Low SDI | Coted'Ivoire | 35 to 39 | 0.91 (-0.18,2) | 0.3 (-0.98,1.6) |
| Low SDI | Coted'Ivoire | 40 to 44 | 0.55 (-0.51,1.62) | 0.06 (-1.17,1.3) |
| Low SDI | Coted'Ivoire | 45 to 49 | 0.3 (-0.73,1.34) | -0.1 (-1.27,1.08) |
| Low SDI | Coted'Ivoire | 50 to 54 | 0.21 (-0.77,1.2) | -0.17 (-1.25,0.93) |
| Low SDI | Coted'Ivoire | 55 to 59 | 0.18 (-0.76,1.12) | -0.16 (-1.17,0.86) |
| Low SDI | Coted'Ivoire | 60 to 64 | 0.16 (-0.75,1.08) | -0.13 (-1.09,0.83) |
| Low SDI | Coted'Ivoire | 65 to 69 | 0.18 (-0.78,1.15) | -0.06 (-1.04,0.92) |
| Low SDI | Coted'Ivoire | 70 to 74 | 0.2 (-0.91,1.33) | -0.01 (-1.09,1.09) |
| Low SDI | Coted'Ivoire | 75 to 79 | 0.36 (-1.07,1.82) | 0.18 (-1.16,1.53) |
| Low SDI | Coted'Ivoire | 80 to 84 | 0.74 (-1.54,3.08) | 0.34 (-1.65,2.37) |
| Low SDI | Coted'Ivoire | 85 to 89 | 0.46 (-3.9,5.02) | -0.06 (-3.66,3.68) |
| Low SDI | Democratic Republic of the Congo | 5 to 9 | -0.96 (-2.18,0.27) | -1.18 (-2.47,0.13) |
| Low SDI | Democratic Republic of the Congo | 10 to 14 | 0.05 (-0.97,1.08) | -0.3 (-1.4,0.81) |
| Low SDI | Democratic Republic of the Congo | 15 to 19 | 0.6 (-0.36,1.58) | 0.13 (-0.92,1.2) |
| Low SDI | Democratic Republic of the Congo | 20 to 24 | 0.9 (-0.04,1.84) | 0.38 (-0.66,1.43) |
| Low SDI | Democratic Republic of the Congo | 25 to 29 | 1.11 (0.23,2) | 0.59 (-0.42,1.61) |
| Low SDI | Democratic Republic of the Congo | 30 to 34 | 1.2 (0.37,2.04) | 0.72 (-0.24,1.69) |
| Low SDI | Democratic Republic of the Congo | 35 to 39 | 1.1 (0.32,1.89) | 0.7 (-0.2,1.61) |
| Low SDI | Democratic Republic of the Congo | 40 to 44 | 1.01 (0.27,1.76) | 0.68 (-0.15,1.52) |
| Low SDI | Democratic Republic of the Congo | 45 to 49 | 0.85 (0.16,1.56) | 0.56 (-0.21,1.33) |
| Low SDI | Democratic Republic of the Congo | 50 to 54 | 0.67 (0.01,1.33) | 0.41 (-0.3,1.12) |
| Low SDI | Democratic Republic of the Congo | 55 to 59 | 0.5 (-0.12,1.13) | 0.28 (-0.38,0.94) |
| Low SDI | Democratic Republic of the Congo | 60 to 64 | 0.39 (-0.24,1.01) | 0.2 (-0.44,0.85) |
| Low SDI | Democratic Republic of the Congo | 65 to 69 | 0.28 (-0.39,0.95) | 0.16 (-0.51,0.83) |
| Low SDI | Democratic Republic of the Congo | 70 to 74 | 0.24 (-0.55,1.03) | 0.13 (-0.63,0.9) |
| Low SDI | Democratic Republic of the Congo | 75 to 79 | 0.21 (-0.83,1.25) | 0.15 (-0.83,1.13) |
| Low SDI | Democratic Republic of the Congo | 80 to 84 | 0.1 (-1.55,1.78) | 0.19 (-1.34,1.74) |
| Low SDI | Democratic Republic of the Congo | 85 to 89 | -0.06 (-3.56,3.56) | -0.08 (-3.14,3.08) |
| Low SDI | Djibouti | 5 to 9 | -0.57 (-6.72,5.99) | -2.66 (-9.82,5.07) |
| Low SDI | Djibouti | 10 to 14 | -1.23 (-6.57,4.41) | -2.41 (-8.16,3.7) |
| Low SDI | Djibouti | 15 to 19 | -1.53 (-6.5,3.69) | -2.24 (-7.87,3.73) |
| Low SDI | Djibouti | 20 to 24 | -1.26 (-6.21,3.94) | -2.25 (-7.94,3.78) |
| Low SDI | Djibouti | 25 to 29 | -0.75 (-5.31,4.03) | -1.88 (-7.56,4.15) |
| Low SDI | Djibouti | 30 to 34 | -0.33 (-4.64,4.17) | -1.4 (-6.86,4.39) |
| Low SDI | Djibouti | 35 to 39 | 0.1 (-3.98,4.34) | -0.98 (-5.8,4.09) |
| Low SDI | Djibouti | 40 to 44 | -0.01 (-3.97,4.12) | -0.78 (-5.16,3.8) |
| Low SDI | Djibouti | 45 to 49 | -0.05 (-3.94,4) | -0.61 (-4.76,3.73) |
| Low SDI | Djibouti | 50 to 54 | 0.58 (-3.28,4.6) | -0.67 (-4.75,3.58) |
| Low SDI | Djibouti | 55 to 59 | 0.58 (-3.33,4.66) | -0.31 (-4.33,3.88) |
| Low SDI | Djibouti | 60 to 64 | 0.82 (-3.29,5.12) | 0.16 (-3.98,4.48) |
| Low SDI | Djibouti | 65 to 69 | 0.14 (-4.19,4.67) | -0.02 (-4.35,4.51) |
| Low SDI | Djibouti | 70 to 74 | -0.73 (-5.67,4.46) | -1.06 (-5.7,3.81) |
| Low SDI | Djibouti | 75 to 79 | 2.18 (-8.82,14.51) | 3.58 (-7.24,15.66) |
| Low SDI | Djibouti | 80 to 84 | 2.56 (-12.3,19.93) | 5.57 (-8.87,22.29) |
| Low SDI | Djibouti | 85 to 89 | -0.44 (-20.79,25.14) | 4.76 (-14.71,28.68) |
| Low SDI | Eritrea | 5 to 9 | -0.63 (-3.16,1.97) | -0.69 (-3.38,2.07) |
| Low SDI | Eritrea | 10 to 14 | -0.31 (-2.49,1.92) | -0.55 (-2.88,1.84) |
| Low SDI | Eritrea | 15 to 19 | 0.21 (-1.85,2.31) | -0.21 (-2.44,2.07) |
| Low SDI | Eritrea | 20 to 24 | 0.43 (-1.58,2.48) | 0.08 (-2.14,2.35) |
| Low SDI | Eritrea | 25 to 29 | 0.56 (-1.37,2.53) | 0.19 (-2.01,2.45) |
| Low SDI | Eritrea | 30 to 34 | 0.52 (-1.33,2.41) | 0.21 (-1.93,2.39) |
| Low SDI | Eritrea | 35 to 39 | 0.41 (-1.34,2.2) | 0.14 (-1.87,2.2) |
| Low SDI | Eritrea | 40 to 44 | 0.22 (-1.45,1.92) | -0.02 (-1.89,1.88) |
| Low SDI | Eritrea | 45 to 49 | -0.04 (-1.65,1.59) | -0.32 (-2.08,1.46) |
| Low SDI | Eritrea | 50 to 54 | -0.25 (-1.79,1.31) | -0.54 (-2.18,1.14) |
| Low SDI | Eritrea | 55 to 59 | -0.46 (-1.97,1.07) | -0.6 (-2.18,1.01) |
| Low SDI | Eritrea | 60 to 64 | -0.52 (-2.08,1.07) | -0.59 (-2.21,1.05) |
| Low SDI | Eritrea | 65 to 69 | -0.39 (-2.15,1.4) | -0.33 (-2.11,1.48) |
| Low SDI | Eritrea | 70 to 74 | -0.1 (-2.31,2.16) | -0.14 (-2.29,2.06) |
| Low SDI | Eritrea | 75 to 79 | -0.31 (-3.18,2.64) | -0.1 (-2.95,2.82) |
| Low SDI | Eritrea | 80 to 84 | 1.29 (-4.48,7.4) | 0 (-4.58,4.8) |
| Low SDI | Eritrea | 85 to 89 | 5.38 (-12.21,26.49) | 4.87 (-12.57,25.8) |
| Low SDI | Ethiopia | 5 to 9 | -1.83 (-2.38,-1.27) | -2.5 (-3.12,-1.89) |
| Low SDI | Ethiopia | 10 to 14 | -1.63 (-2.13,-1.14) | -2.38 (-2.93,-1.83) |
| Low SDI | Ethiopia | 15 to 19 | -1.33 (-1.84,-0.82) | -2.15 (-2.71,-1.58) |
| Low SDI | Ethiopia | 20 to 24 | -0.97 (-1.49,-0.44) | -1.85 (-2.44,-1.26) |
| Low SDI | Ethiopia | 25 to 29 | -0.72 (-1.24,-0.19) | -1.65 (-2.25,-1.05) |
| Low SDI | Ethiopia | 30 to 34 | -0.72 (-1.23,-0.2) | -1.62 (-2.21,-1.03) |
| Low SDI | Ethiopia | 35 to 39 | -1.02 (-1.51,-0.53) | -1.82 (-2.38,-1.26) |
| Low SDI | Ethiopia | 40 to 44 | -1.5 (-1.96,-1.03) | -2.21 (-2.74,-1.68) |
| Low SDI | Ethiopia | 45 to 49 | -1.93 (-2.37,-1.48) | -2.55 (-3.05,-2.06) |
| Low SDI | Ethiopia | 50 to 54 | -2.12 (-2.54,-1.7) | -2.66 (-3.12,-2.2) |
| Low SDI | Ethiopia | 55 to 59 | -2.09 (-2.5,-1.68) | -2.56 (-3,-2.13) |
| Low SDI | Ethiopia | 60 to 64 | -1.91 (-2.31,-1.52) | -2.31 (-2.72,-1.9) |
| Low SDI | Ethiopia | 65 to 69 | -1.61 (-2.02,-1.2) | -1.95 (-2.36,-1.53) |
| Low SDI | Ethiopia | 70 to 74 | -1.22 (-1.68,-0.75) | -1.49 (-1.94,-1.04) |
| Low SDI | Ethiopia | 75 to 79 | -0.75 (-1.33,-0.16) | -0.98 (-1.52,-0.43) |
| Low SDI | Ethiopia | 80 to 84 | -0.02 (-0.96,0.93) | -0.23 (-1.07,0.61) |
| Low SDI | Ethiopia | 85 to 89 | 0.64 (-1.48,2.81) | 0.62 (-1.29,2.55) |
| Low SDI | Gambia | 5 to 9 | 0.07 (-4.4,4.76) | -0.58 (-5.46,4.55) |
| Low SDI | Gambia | 10 to 14 | 0.98 (-3.23,5.39) | 0 (-4.53,4.76) |
| Low SDI | Gambia | 15 to 19 | 1.05 (-3.29,5.58) | -0.21 (-4.79,4.58) |
| Low SDI | Gambia | 20 to 24 | 1.02 (-3.31,5.54) | -0.88 (-5.89,4.4) |
| Low SDI | Gambia | 25 to 29 | 1.08 (-3.18,5.52) | -0.21 (-5,4.82) |
| Low SDI | Gambia | 30 to 34 | 1.54 (-2.68,5.95) | -0.59 (-5.23,4.27) |
| Low SDI | Gambia | 35 to 39 | 1.3 (-2.88,5.65) | -0.95 (-5.46,3.77) |
| Low SDI | Gambia | 40 to 44 | 1.1 (-3.02,5.38) | -1.02 (-5.45,3.62) |
| Low SDI | Gambia | 45 to 49 | 0.95 (-3.05,5.12) | -0.61 (-5.02,4) |
| Low SDI | Gambia | 50 to 54 | 0.63 (-3.24,4.66) | 0.02 (-4.19,4.42) |
| Low SDI | Gambia | 55 to 59 | 0.7 (-3.03,4.57) | 0.58 (-3.28,4.6) |
| Low SDI | Gambia | 60 to 64 | 1.06 (-2.36,4.59) | 0.88 (-2.83,4.73) |
| Low SDI | Gambia | 65 to 69 | 0.63 (-2.78,4.16) | 0.14 (-3.43,3.84) |
| Low SDI | Gambia | 70 to 74 | 1.53 (-2.67,5.91) | 0.43 (-3.53,4.56) |
| Low SDI | Gambia | 75 to 79 | 1.35 (-3.61,6.55) | 1.5 (-3.33,6.57) |
| Low SDI | Gambia | 80 to 84 | 0.48 (-6.01,7.41) | 1.25 (-5.11,8.05) |
| Low SDI | Gambia | 85 to 89 | 3.35 (-14.15,24.43) | 3.55 (-13.99,24.65) |
| Low SDI | Guinea | 5 to 9 | -0.33 (-2.27,1.64) | -0.41 (-2.5,1.73) |
| Low SDI | Guinea | 10 to 14 | 0.33 (-1.69,2.4) | -0.04 (-2.23,2.2) |
| Low SDI | Guinea | 15 to 19 | 0.92 (-1.39,3.29) | 0.32 (-2.2,2.91) |
| Low SDI | Guinea | 20 to 24 | 1.29 (-1.18,3.83) | 0.66 (-2.1,3.5) |
| Low SDI | Guinea | 25 to 29 | 1.43 (-1.06,3.99) | 0.89 (-1.97,3.84) |
| Low SDI | Guinea | 30 to 34 | 1.35 (-1.1,3.85) | 0.77 (-2.04,3.67) |
| Low SDI | Guinea | 35 to 39 | 1.07 (-1.31,3.51) | 0.69 (-2.04,3.49) |
| Low SDI | Guinea | 40 to 44 | 0.82 (-1.45,3.15) | 0.49 (-2.06,3.11) |
| Low SDI | Guinea | 45 to 49 | 0.61 (-1.51,2.78) | 0.33 (-2.03,2.75) |
| Low SDI | Guinea | 50 to 54 | 0.54 (-1.4,2.52) | 0.3 (-1.82,2.47) |
| Low SDI | Guinea | 55 to 59 | 0.55 (-1.22,2.35) | 0.4 (-1.46,2.3) |
| Low SDI | Guinea | 60 to 64 | 0.55 (-1.08,2.2) | 0.32 (-1.35,2.01) |
| Low SDI | Guinea | 65 to 69 | 0.66 (-0.92,2.26) | 0.43 (-1.15,2.05) |
| Low SDI | Guinea | 70 to 74 | 0.69 (-0.99,2.39) | 0.53 (-1.11,2.2) |
| Low SDI | Guinea | 75 to 79 | 0.85 (-1.2,2.96) | 0.73 (-1.19,2.69) |
| Low SDI | Guinea | 80 to 84 | 0.96 (-2.08,4.09) | 0.68 (-1.97,3.4) |
| Low SDI | Guinea | 85 to 89 | 1.46 (-4.67,7.99) | -0.07 (-4.63,4.72) |
| Low SDI | Guinea-Bissau | 5 to 9 | -1.25 (-5.64,3.34) | -1.39 (-6.05,3.5) |
| Low SDI | Guinea-Bissau | 10 to 14 | 0.11 (-3.65,4.02) | -0.92 (-5.34,3.71) |
| Low SDI | Guinea-Bissau | 15 to 19 | 0.42 (-3.58,4.59) | -0.65 (-5.24,4.16) |
| Low SDI | Guinea-Bissau | 20 to 24 | 0.6 (-3.54,4.93) | -0.47 (-5.09,4.37) |
| Low SDI | Guinea-Bissau | 25 to 29 | 0.96 (-3.25,5.35) | -0.68 (-5.12,3.98) |
| Low SDI | Guinea-Bissau | 30 to 34 | 1.06 (-3.05,5.34) | -0.65 (-5.04,3.94) |
| Low SDI | Guinea-Bissau | 35 to 39 | 1.24 (-2.81,5.45) | 0.25 (-4.1,4.8) |
| Low SDI | Guinea-Bissau | 40 to 44 | 1.18 (-2.84,5.36) | 0.57 (-3.71,5.03) |
| Low SDI | Guinea-Bissau | 45 to 49 | 0.65 (-3.26,4.71) | 0.68 (-3.5,5.04) |
| Low SDI | Guinea-Bissau | 50 to 54 | 0.62 (-3.22,4.62) | 0.83 (-3.14,4.97) |
| Low SDI | Guinea-Bissau | 55 to 59 | 0.88 (-2.95,4.85) | 0.73 (-3.18,4.8) |
| Low SDI | Guinea-Bissau | 60 to 64 | 0.66 (-3.12,4.58) | 0.38 (-3.41,4.33) |
| Low SDI | Guinea-Bissau | 65 to 69 | 0.58 (-3.45,4.78) | 0.33 (-3.61,4.43) |
| Low SDI | Guinea-Bissau | 70 to 74 | 0.82 (-4.22,6.12) | -0.11 (-4.75,4.75) |
| Low SDI | Guinea-Bissau | 75 to 79 | 0.27 (-6.39,7.4) | -0.08 (-6.57,6.85) |
| Low SDI | Guinea-Bissau | 80 to 84 | 2.16 (-11.47,17.88) | 2.95 (-10.65,18.63) |
| Low SDI | Guinea-Bissau | 85 to 89 | 0.89 (-19.43,26.35) | 1.72 (-18.7,27.27) |
| Low SDI | Haiti | 5 to 9 | -0.15 (-1.64,1.37) | -0.64 (-2.4,1.15) |
| Low SDI | Haiti | 10 to 14 | 0.09 (-1.3,1.51) | -0.34 (-1.93,1.27) |
| Low SDI | Haiti | 15 to 19 | 0.31 (-1.08,1.71) | -0.16 (-1.72,1.42) |
| Low SDI | Haiti | 20 to 24 | 0.31 (-1.05,1.69) | -0.09 (-1.61,1.45) |
| Low SDI | Haiti | 25 to 29 | 0.32 (-0.99,1.65) | -0.05 (-1.52,1.45) |
| Low SDI | Haiti | 30 to 34 | 0.25 (-1,1.51) | -0.08 (-1.5,1.35) |
| Low SDI | Haiti | 35 to 39 | 0.12 (-1.08,1.34) | -0.19 (-1.55,1.18) |
| Low SDI | Haiti | 40 to 44 | 0 (-1.16,1.18) | -0.24 (-1.54,1.07) |
| Low SDI | Haiti | 45 to 49 | -0.06 (-1.17,1.07) | -0.28 (-1.51,0.96) |
| Low SDI | Haiti | 50 to 54 | -0.12 (-1.19,0.95) | -0.33 (-1.49,0.84) |
| Low SDI | Haiti | 55 to 59 | -0.11 (-1.13,0.92) | -0.29 (-1.37,0.81) |
| Low SDI | Haiti | 60 to 64 | -0.06 (-1.07,0.96) | -0.23 (-1.29,0.83) |
| Low SDI | Haiti | 65 to 69 | 0.01 (-1.06,1.09) | -0.19 (-1.27,0.91) |
| Low SDI | Haiti | 70 to 74 | 0.1 (-1.16,1.37) | -0.09 (-1.33,1.16) |
| Low SDI | Haiti | 75 to 79 | 0.22 (-1.47,1.94) | -0.04 (-1.64,1.59) |
| Low SDI | Haiti | 80 to 84 | 0.4 (-2.3,3.17) | 0.3 (-2.21,2.89) |
| Low SDI | Haiti | 85 to 89 | 1.7 (-4.35,8.12) | 0.09 (-4.4,4.78) |
| Low SDI | Liberia | 5 to 9 | 0.29 (-2.49,3.14) | -0.05 (-3.11,3.09) |
| Low SDI | Liberia | 10 to 14 | 0.94 (-1.78,3.73) | 0.58 (-2.53,3.8) |
| Low SDI | Liberia | 15 to 19 | 1.55 (-1.53,4.72) | 0.9 (-2.69,4.62) |
| Low SDI | Liberia | 20 to 24 | 1.84 (-1.5,5.29) | 1.67 (-2.38,5.89) |
| Low SDI | Liberia | 25 to 29 | 2.68 (-0.8,6.29) | 1.91 (-2.22,6.21) |
| Low SDI | Liberia | 30 to 34 | 3.06 (-0.29,6.54) | 1.86 (-2.1,5.98) |
| Low SDI | Liberia | 35 to 39 | 2.82 (-0.42,6.16) | 1.6 (-2.15,5.49) |
| Low SDI | Liberia | 40 to 44 | 2.45 (-0.7,5.7) | 1.86 (-1.76,5.6) |
| Low SDI | Liberia | 45 to 49 | 2.25 (-0.89,5.49) | 1.42 (-2.08,5.04) |
| Low SDI | Liberia | 50 to 54 | 1.34 (-1.64,4.4) | 1.19 (-2.08,4.57) |
| Low SDI | Liberia | 55 to 59 | 1.42 (-1.38,4.3) | 1.05 (-1.96,4.16) |
| Low SDI | Liberia | 60 to 64 | 1.22 (-1.47,4) | 0.81 (-2.01,3.71) |
| Low SDI | Liberia | 65 to 69 | 0.93 (-1.78,3.71) | 0.52 (-2.25,3.37) |
| Low SDI | Liberia | 70 to 74 | 0.45 (-2.53,3.52) | 0.48 (-2.5,3.56) |
| Low SDI | Liberia | 75 to 79 | 0.28 (-3.09,3.76) | 0.31 (-3.05,3.79) |
| Low SDI | Liberia | 80 to 84 | 1.63 (-4.48,8.13) | -0.46 (-4.81,4.09) |
| Low SDI | Liberia | 85 to 89 | 6.46 (-11.41,27.95) | 5.39 (-12.18,26.48) |
| Low SDI | Madagascar | 5 to 9 | -0.63 (-1.93,0.68) | -0.87 (-2.26,0.55) |
| Low SDI | Madagascar | 10 to 14 | 0 (-1.13,1.14) | -0.32 (-1.56,0.94) |
| Low SDI | Madagascar | 15 to 19 | 0.38 (-0.73,1.5) | -0.01 (-1.24,1.24) |
| Low SDI | Madagascar | 20 to 24 | 0.5 (-0.6,1.61) | 0.1 (-1.15,1.36) |
| Low SDI | Madagascar | 25 to 29 | 0.52 (-0.57,1.62) | 0.12 (-1.14,1.39) |
| Low SDI | Madagascar | 30 to 34 | 0.51 (-0.57,1.6) | 0.15 (-1.1,1.41) |
| Low SDI | Madagascar | 35 to 39 | 0.41 (-0.65,1.48) | 0.07 (-1.15,1.31) |
| Low SDI | Madagascar | 40 to 44 | 0.26 (-0.78,1.31) | 0.02 (-1.16,1.21) |
| Low SDI | Madagascar | 45 to 49 | 0.17 (-0.85,1.2) | -0.09 (-1.22,1.05) |
| Low SDI | Madagascar | 50 to 54 | 0.05 (-0.93,1.05) | -0.2 (-1.27,0.88) |
| Low SDI | Madagascar | 55 to 59 | -0.03 (-0.99,0.94) | -0.26 (-1.27,0.77) |
| Low SDI | Madagascar | 60 to 64 | -0.07 (-1.03,0.89) | -0.28 (-1.26,0.72) |
| Low SDI | Madagascar | 65 to 69 | -0.15 (-1.15,0.86) | -0.34 (-1.35,0.69) |
| Low SDI | Madagascar | 70 to 74 | -0.19 (-1.35,0.98) | -0.3 (-1.43,0.85) |
| Low SDI | Madagascar | 75 to 79 | -0.03 (-1.55,1.51) | -0.12 (-1.55,1.34) |
| Low SDI | Madagascar | 80 to 84 | 0.21 (-1.92,2.38) | 0.05 (-1.88,2.01) |
| Low SDI | Madagascar | 85 to 89 | -0.01 (-3.39,3.5) | 0 (-3.04,3.14) |
| Low SDI | Malawi | 5 to 9 | -0.16 (-0.98,0.67) | -0.64 (-1.56,0.28) |
| Low SDI | Malawi | 10 to 14 | 0.6 (-0.13,1.34) | 0.08 (-0.74,0.91) |
| Low SDI | Malawi | 15 to 19 | 1.05 (0.31,1.79) | 0.49 (-0.34,1.32) |
| Low SDI | Malawi | 20 to 24 | 1.26 (0.51,2.02) | 0.63 (-0.22,1.5) |
| Low SDI | Malawi | 25 to 29 | 1.33 (0.57,2.09) | 0.7 (-0.18,1.58) |
| Low SDI | Malawi | 30 to 34 | 1.35 (0.59,2.12) | 0.76 (-0.12,1.66) |
| Low SDI | Malawi | 35 to 39 | 1.2 (0.45,1.97) | 0.7 (-0.18,1.58) |
| Low SDI | Malawi | 40 to 44 | 0.99 (0.24,1.75) | 0.57 (-0.28,1.43) |
| Low SDI | Malawi | 45 to 49 | 0.82 (0.09,1.56) | 0.44 (-0.37,1.26) |
| Low SDI | Malawi | 50 to 54 | 0.7 (0,1.42) | 0.36 (-0.42,1.13) |
| Low SDI | Malawi | 55 to 59 | 0.69 (0,1.38) | 0.37 (-0.35,1.1) |
| Low SDI | Malawi | 60 to 64 | 0.77 (0.11,1.44) | 0.48 (-0.21,1.17) |
| Low SDI | Malawi | 65 to 69 | 0.81 (0.14,1.49) | 0.59 (-0.08,1.27) |
| Low SDI | Malawi | 70 to 74 | 0.8 (0.05,1.56) | 0.64 (-0.09,1.37) |
| Low SDI | Malawi | 75 to 79 | 0.7 (-0.26,1.67) | 0.63 (-0.27,1.55) |
| Low SDI | Malawi | 80 to 84 | 0.74 (-0.66,2.17) | 0.68 (-0.6,1.96) |
| Low SDI | Malawi | 85 to 89 | 0.62 (-1.97,3.27) | 0.82 (-1.58,3.26) |
| Low SDI | Mali | 5 to 9 | -0.44 (-2.35,1.52) | -0.76 (-2.82,1.34) |
| Low SDI | Mali | 10 to 14 | -0.08 (-2.1,1.99) | -0.11 (-2.36,2.2) |
| Low SDI | Mali | 15 to 19 | 0.29 (-2.02,2.66) | 0.26 (-2.32,2.9) |
| Low SDI | Mali | 20 to 24 | 0.44 (-2.03,2.97) | 0.54 (-2.27,3.43) |
| Low SDI | Mali | 25 to 29 | 0.46 (-2.02,3.02) | 0.38 (-2.51,3.34) |
| Low SDI | Mali | 30 to 34 | 0.48 (-1.96,2.99) | 0.3 (-2.55,3.24) |
| Low SDI | Mali | 35 to 39 | 0.48 (-1.88,2.88) | 0.21 (-2.54,3.05) |
| Low SDI | Mali | 40 to 44 | 0.42 (-1.83,2.72) | 0.18 (-2.38,2.81) |
| Low SDI | Mali | 45 to 49 | 0.37 (-1.73,2.52) | 0.01 (-2.34,2.42) |
| Low SDI | Mali | 50 to 54 | 0.11 (-1.82,2.09) | -0.09 (-2.18,2.04) |
| Low SDI | Mali | 55 to 59 | 0.19 (-1.58,1.99) | -0.2 (-2.08,1.71) |
| Low SDI | Mali | 60 to 64 | 0.25 (-1.42,1.94) | -0.06 (-1.79,1.71) |
| Low SDI | Mali | 65 to 69 | 0.36 (-1.39,2.14) | 0.08 (-1.67,1.86) |
| Low SDI | Mali | 70 to 74 | 0.24 (-1.76,2.29) | 0.3 (-1.7,2.35) |
| Low SDI | Mali | 75 to 79 | 0.02 (-2.42,2.52) | 0.58 (-1.85,3.06) |
| Low SDI | Mali | 80 to 84 | 0.15 (-3.67,4.11) | 0.35 (-3.05,3.86) |
| Low SDI | Mali | 85 to 89 | -0.69 (-7.15,6.22) | -0.19 (-6.47,6.52) |
| Low SDI | Mozambique | 5 to 9 | 0.02 (-2.77,2.89) | -0.44 (-3.5,2.71) |
| Low SDI | Mozambique | 10 to 14 | 0.61 (-1.79,3.07) | 0.43 (-2.17,3.09) |
| Low SDI | Mozambique | 15 to 19 | 1.64 (-0.55,3.87) | 1.41 (-1,3.88) |
| Low SDI | Mozambique | 20 to 24 | 2.14 (0.08,4.24) | 1.98 (-0.35,4.36) |
| Low SDI | Mozambique | 25 to 29 | 2.34 (0.43,4.3) | 2.21 (0.01,4.47) |
| Low SDI | Mozambique | 30 to 34 | 2.5 (0.72,4.3) | 2.21 (0.16,4.31) |
| Low SDI | Mozambique | 35 to 39 | 2.37 (0.76,4) | 2.14 (0.3,4.01) |
| Low SDI | Mozambique | 40 to 44 | 2.23 (0.79,3.69) | 1.99 (0.38,3.64) |
| Low SDI | Mozambique | 45 to 49 | 2.09 (0.81,3.4) | 1.86 (0.45,3.29) |
| Low SDI | Mozambique | 50 to 54 | 1.89 (0.73,3.06) | 1.72 (0.47,2.98) |
| Low SDI | Mozambique | 55 to 59 | 1.66 (0.61,2.72) | 1.52 (0.41,2.64) |
| Low SDI | Mozambique | 60 to 64 | 1.55 (0.56,2.54) | 1.43 (0.41,2.46) |
| Low SDI | Mozambique | 65 to 69 | 1.45 (0.48,2.44) | 1.37 (0.39,2.36) |
| Low SDI | Mozambique | 70 to 74 | 1.43 (0.37,2.5) | 1.34 (0.32,2.38) |
| Low SDI | Mozambique | 75 to 79 | 1.47 (0.16,2.79) | 1.34 (0.12,2.57) |
| Low SDI | Mozambique | 80 to 84 | 1.38 (-0.45,3.24) | 1.41 (-0.26,3.11) |
| Low SDI | Mozambique | 85 to 89 | 1.37 (-1.88,4.72) | 1.47 (-1.45,4.48) |
| Low SDI | Nepal | 5 to 9 | -0.18 (-1.69,1.36) | -1.65 (-3.68,0.42) |
| Low SDI | Nepal | 10 to 14 | 0.73 (-0.55,2.03) | -0.72 (-2.36,0.95) |
| Low SDI | Nepal | 15 to 19 | 1.19 (-0.08,2.47) | -0.16 (-1.72,1.43) |
| Low SDI | Nepal | 20 to 24 | 1.22 (-0.06,2.51) | -0.05 (-1.6,1.53) |
| Low SDI | Nepal | 25 to 29 | 1.13 (-0.12,2.4) | -0.08 (-1.62,1.49) |
| Low SDI | Nepal | 30 to 34 | 1.14 (-0.08,2.38) | -0.08 (-1.57,1.44) |
| Low SDI | Nepal | 35 to 39 | 1.09 (-0.08,2.28) | -0.03 (-1.45,1.41) |
| Low SDI | Nepal | 40 to 44 | 1.1 (-0.02,2.23) | 0.09 (-1.24,1.43) |
| Low SDI | Nepal | 45 to 49 | 1.1 (0.05,2.16) | 0.18 (-1.03,1.41) |
| Low SDI | Nepal | 50 to 54 | 1.02 (0.05,2) | 0.26 (-0.83,1.36) |
| Low SDI | Nepal | 55 to 59 | 0.99 (0.1,1.9) | 0.36 (-0.62,1.35) |
| Low SDI | Nepal | 60 to 64 | 1.04 (0.18,1.9) | 0.47 (-0.44,1.39) |
| Low SDI | Nepal | 65 to 69 | 1.11 (0.24,1.98) | 0.65 (-0.24,1.55) |
| Low SDI | Nepal | 70 to 74 | 1.33 (0.34,2.33) | 0.88 (-0.08,1.86) |
| Low SDI | Nepal | 75 to 79 | 1.62 (0.31,2.95) | 1.29 (0.06,2.54) |
| Low SDI | Nepal | 80 to 84 | 1.96 (0.04,3.92) | 1.7 (-0.04,3.47) |
| Low SDI | Nepal | 85 to 89 | 2.5 (-1.1,6.23) | 2.21 (-0.94,5.45) |
| Low SDI | Niger | 5 to 9 | -1.63 (-3.12,-0.11) | -1.87 (-3.46,-0.26) |
| Low SDI | Niger | 10 to 14 | -0.79 (-2.41,0.85) | -1.07 (-2.84,0.72) |
| Low SDI | Niger | 15 to 19 | -0.25 (-2.2,1.75) | -0.59 (-2.77,1.64) |
| Low SDI | Niger | 20 to 24 | 0.31 (-1.88,2.54) | -0.03 (-2.5,2.5) |
| Low SDI | Niger | 25 to 29 | 0.69 (-1.55,2.99) | 0.36 (-2.23,3.01) |
| Low SDI | Niger | 30 to 34 | 0.81 (-1.39,3.07) | 0.51 (-2.04,3.13) |
| Low SDI | Niger | 35 to 39 | 0.61 (-1.49,2.76) | 0.32 (-2.1,2.81) |
| Low SDI | Niger | 40 to 44 | 0.39 (-1.65,2.47) | 0.2 (-2.11,2.57) |
| Low SDI | Niger | 45 to 49 | 0.25 (-1.69,2.24) | 0.07 (-2.09,2.27) |
| Low SDI | Niger | 50 to 54 | 0.11 (-1.69,1.94) | -0.06 (-2.01,1.94) |
| Low SDI | Niger | 55 to 59 | -0.07 (-1.79,1.69) | -0.12 (-1.95,1.75) |
| Low SDI | Niger | 60 to 64 | -0.18 (-1.9,1.58) | -0.25 (-2.05,1.57) |
| Low SDI | Niger | 65 to 69 | -0.2 (-2.02,1.66) | -0.16 (-2.01,1.71) |
| Low SDI | Niger | 70 to 74 | -0.21 (-2.36,1.98) | -0.02 (-2.13,2.13) |
| Low SDI | Niger | 75 to 79 | -0.48 (-3.21,2.32) | -0.01 (-2.71,2.77) |
| Low SDI | Niger | 80 to 84 | -1.2 (-5.16,2.92) | -0.36 (-4.29,3.74) |
| Low SDI | Niger | 85 to 89 | 3.98 (-13.3,24.71) | -2.04 (-8.58,4.98) |
| Low SDI | Papua New Guinea | 5 to 9 | 0.9 (-2.53,4.45) | -1.06 (-7.61,5.95) |
| Low SDI | Papua New Guinea | 10 to 14 | 1.33 (-0.95,3.65) | 0.35 (-3.49,4.34) |
| Low SDI | Papua New Guinea | 15 to 19 | 1.4 (-0.75,3.59) | 0.62 (-2.66,4) |
| Low SDI | Papua New Guinea | 20 to 24 | 1.16 (-0.94,3.31) | 0.62 (-2.48,3.82) |
| Low SDI | Papua New Guinea | 25 to 29 | 0.9 (-1.15,3) | 0.33 (-2.59,3.33) |
| Low SDI | Papua New Guinea | 30 to 34 | 0.8 (-1.37,3.02) | 0.01 (-2.92,3.03) |
| Low SDI | Papua New Guinea | 35 to 39 | 0.7 (-1.6,3.07) | 0.19 (-2.66,3.12) |
| Low SDI | Papua New Guinea | 40 to 44 | 0.41 (-2.01,2.89) | 0.16 (-2.65,3.05) |
| Low SDI | Papua New Guinea | 45 to 49 | 0.21 (-2.28,2.77) | 0.13 (-2.68,3.01) |
| Low SDI | Papua New Guinea | 50 to 54 | -0.02 (-2.59,2.61) | 0.29 (-2.51,3.17) |
| Low SDI | Papua New Guinea | 55 to 59 | -0.02 (-2.6,2.61) | 0.35 (-2.41,3.19) |
| Low SDI | Papua New Guinea | 60 to 64 | 0.01 (-2.59,2.67) | 0.17 (-2.56,2.98) |
| Low SDI | Papua New Guinea | 65 to 69 | 0.15 (-2.49,2.86) | -0.19 (-2.9,2.59) |
| Low SDI | Papua New Guinea | 70 to 74 | 0.49 (-2.43,3.5) | -0.16 (-3.04,2.8) |
| Low SDI | Papua New Guinea | 75 to 79 | 0.83 (-3.13,4.94) | -0.38 (-3.84,3.21) |
| Low SDI | Papua New Guinea | 80 to 84 | 0.91 (-5.2,7.41) | 0.94 (-5.04,7.3) |
| Low SDI | Papua New Guinea | 85 to 89 | 3.66 (-13.76,24.61) | 4.37 (-13.15,25.41) |
| Low SDI | Rwanda | 5 to 9 | -3.17 (-4.7,-1.62) | -3.63 (-5.3,-1.94) |
| Low SDI | Rwanda | 10 to 14 | -2.46 (-3.74,-1.16) | -3.01 (-4.41,-1.59) |
| Low SDI | Rwanda | 15 to 19 | -1.82 (-3.07,-0.56) | -2.44 (-3.83,-1.04) |
| Low SDI | Rwanda | 20 to 24 | -1.48 (-2.73,-0.21) | -2.18 (-3.58,-0.75) |
| Low SDI | Rwanda | 25 to 29 | -1.4 (-2.61,-0.18) | -2.15 (-3.54,-0.75) |
| Low SDI | Rwanda | 30 to 34 | -1.5 (-2.66,-0.33) | -2.24 (-3.57,-0.88) |
| Low SDI | Rwanda | 35 to 39 | -1.71 (-2.83,-0.58) | -2.42 (-3.7,-1.12) |
| Low SDI | Rwanda | 40 to 44 | -2.05 (-3.15,-0.94) | -2.75 (-3.99,-1.49) |
| Low SDI | Rwanda | 45 to 49 | -2.22 (-3.3,-1.14) | -2.86 (-4.06,-1.65) |
| Low SDI | Rwanda | 50 to 54 | -2.26 (-3.29,-1.21) | -2.83 (-3.94,-1.7) |
| Low SDI | Rwanda | 55 to 59 | -2.15 (-3.13,-1.16) | -2.66 (-3.7,-1.62) |
| Low SDI | Rwanda | 60 to 64 | -1.91 (-2.87,-0.94) | -2.35 (-3.35,-1.35) |
| Low SDI | Rwanda | 65 to 69 | -1.58 (-2.6,-0.55) | -1.98 (-3,-0.94) |
| Low SDI | Rwanda | 70 to 74 | -1.17 (-2.34,0.01) | -1.55 (-2.68,-0.4) |
| Low SDI | Rwanda | 75 to 79 | -0.71 (-2.24,0.84) | -1.08 (-2.5,0.36) |
| Low SDI | Rwanda | 80 to 84 | -0.28 (-2.52,2.01) | -0.71 (-2.69,1.31) |
| Low SDI | Rwanda | 85 to 89 | 0.33 (-4.04,4.89) | -0.32 (-3.94,3.44) |
| Low SDI | Senegal | 5 to 9 | -0.03 (-1.83,1.81) | -0.39 (-2.37,1.64) |
| Low SDI | Senegal | 10 to 14 | 0.75 (-0.94,2.47) | 0.28 (-1.62,2.22) |
| Low SDI | Senegal | 15 to 19 | 1.2 (-0.61,3.04) | 0.75 (-1.34,2.89) |
| Low SDI | Senegal | 20 to 24 | 1.42 (-0.45,3.32) | 1.06 (-1.16,3.32) |
| Low SDI | Senegal | 25 to 29 | 1.53 (-0.33,3.42) | 1.25 (-1.01,3.56) |
| Low SDI | Senegal | 30 to 34 | 1.56 (-0.28,3.43) | 1.34 (-0.89,3.61) |
| Low SDI | Senegal | 35 to 39 | 1.49 (-0.3,3.32) | 1.22 (-0.92,3.41) |
| Low SDI | Senegal | 40 to 44 | 1.5 (-0.25,3.28) | 1.06 (-0.96,3.13) |
| Low SDI | Senegal | 45 to 49 | 1.48 (-0.21,3.2) | 0.94 (-0.97,2.88) |
| Low SDI | Senegal | 50 to 54 | 1.29 (-0.3,2.9) | 0.75 (-1.01,2.53) |
| Low SDI | Senegal | 55 to 59 | 1.12 (-0.39,2.65) | 0.68 (-0.95,2.33) |
| Low SDI | Senegal | 60 to 64 | 0.83 (-0.63,2.31) | 0.56 (-0.97,2.12) |
| Low SDI | Senegal | 65 to 69 | 0.62 (-0.89,2.15) | 0.48 (-1.06,2.06) |
| Low SDI | Senegal | 70 to 74 | 0.34 (-1.37,2.08) | 0.31 (-1.38,2.03) |
| Low SDI | Senegal | 75 to 79 | 0.18 (-1.93,2.34) | 0.2 (-1.81,2.25) |
| Low SDI | Senegal | 80 to 84 | 0.43 (-2.85,3.81) | 0.4 (-2.58,3.47) |
| Low SDI | Senegal | 85 to 89 | 0.34 (-5.83,6.91) | 1.1 (-4.97,7.56) |
| Low SDI | Sierra Leone | 5 to 9 | 0.59 (-1.69,2.93) | 0.3 (-2.17,2.84) |
| Low SDI | Sierra Leone | 10 to 14 | 1.17 (-1.21,3.61) | 0.75 (-1.87,3.43) |
| Low SDI | Sierra Leone | 15 to 19 | 1.77 (-0.97,4.58) | 1.19 (-1.88,4.35) |
| Low SDI | Sierra Leone | 20 to 24 | 2.33 (-0.61,5.36) | 1.64 (-1.76,5.16) |
| Low SDI | Sierra Leone | 25 to 29 | 2.48 (-0.45,5.49) | 2.14 (-1.42,5.82) |
| Low SDI | Sierra Leone | 30 to 34 | 2.27 (-0.54,5.17) | 2.36 (-1.08,5.92) |
| Low SDI | Sierra Leone | 35 to 39 | 1.86 (-0.82,4.62) | 2.04 (-1.11,5.3) |
| Low SDI | Sierra Leone | 40 to 44 | 1.55 (-1.04,4.2) | 1.61 (-1.32,4.63) |
| Low SDI | Sierra Leone | 45 to 49 | 1.27 (-1.25,3.85) | 1.21 (-1.55,4.06) |
| Low SDI | Sierra Leone | 50 to 54 | 1.4 (-0.99,3.85) | 0.78 (-1.77,3.4) |
| Low SDI | Sierra Leone | 55 to 59 | 1.3 (-0.98,3.64) | 0.69 (-1.71,3.15) |
| Low SDI | Sierra Leone | 60 to 64 | 1 (-1.18,3.23) | 0.55 (-1.72,2.88) |
| Low SDI | Sierra Leone | 65 to 69 | 0.68 (-1.53,2.94) | 0.31 (-1.93,2.6) |
| Low SDI | Sierra Leone | 70 to 74 | 0.18 (-2.18,2.59) | -0.06 (-2.39,2.33) |
| Low SDI | Sierra Leone | 75 to 79 | -0.29 (-2.98,2.47) | -0.05 (-2.71,2.68) |
| Low SDI | Sierra Leone | 80 to 84 | 0.24 (-3.77,4.41) | -0.47 (-4.11,3.3) |
| Low SDI | Sierra Leone | 85 to 89 | -0.55 (-7.23,6.62) | -1.13 (-7.67,5.87) |
| Low SDI | Solomon Islands | 5 to 9 | 2.99 (-16.51,27.03) | -2.31 (-23.04,24.01) |
| Low SDI | Solomon Islands | 10 to 14 | 6.03 (-4.57,17.82) | -1.78 (-18.3,18.07) |
| Low SDI | Solomon Islands | 15 to 19 | 6.53 (-2.89,16.87) | -0.29 (-14.89,16.81) |
| Low SDI | Solomon Islands | 20 to 24 | 5.26 (-2.74,13.93) | 1.87 (-11.36,17.08) |
| Low SDI | Solomon Islands | 25 to 29 | 2.4 (-4.14,9.39) | 3.27 (-8.64,16.73) |
| Low SDI | Solomon Islands | 30 to 34 | -0.49 (-7.82,7.42) | 4.36 (-7.31,17.5) |
| Low SDI | Solomon Islands | 35 to 39 | 0.58 (-7.34,9.17) | 4.72 (-6.34,17.09) |
| Low SDI | Solomon Islands | 40 to 44 | 2.85 (-6.32,12.92) | 6.75 (-4.65,19.5) |
| Low SDI | Solomon Islands | 45 to 49 | 8.11 (-2.3,19.64) | 9.84 (-1.48,22.45) |
| Low SDI | Solomon Islands | 50 to 54 | 10.97 (-0.44,23.69) | 9.85 (-1.71,22.76) |
| Low SDI | Solomon Islands | 55 to 59 | 10.93 (-0.5,23.67) | 9.31 (-2.49,22.53) |
| Low SDI | Solomon Islands | 60 to 64 | 10.72 (-1.32,24.23) | 10.29 (-1.9,23.99) |
| Low SDI | Solomon Islands | 65 to 69 | 7.98 (-4.86,22.54) | 8.57 (-4.43,23.34) |
| Low SDI | Solomon Islands | 70 to 74 | 3.83 (-10.17,20.01) | 6.59 (-6.94,22.08) |
| Low SDI | Solomon Islands | 75 to 79 | 0.74 (-14.41,18.57) | 3.81 (-10.53,20.45) |
| Low SDI | Solomon Islands | 80 to 84 | -2.73 (-19.21,17.11) | -1.1 (-17.67,18.8) |
| Low SDI | Solomon Islands | 85 to 89 | -5.44 (-25.59,20.16) | -3.85 (-24.41,22.3) |
| Low SDI | Somalia | 5 to 9 | -0.99 (-2.47,0.51) | -0.78 (-2.33,0.8) |
| Low SDI | Somalia | 10 to 14 | -0.34 (-1.61,0.94) | -0.3 (-1.65,1.08) |
| Low SDI | Somalia | 15 to 19 | 0.02 (-1.25,1.29) | -0.08 (-1.43,1.29) |
| Low SDI | Somalia | 20 to 24 | 0.31 (-1.01,1.66) | 0.14 (-1.31,1.61) |
| Low SDI | Somalia | 25 to 29 | 0.46 (-0.89,1.83) | 0.21 (-1.29,1.74) |
| Low SDI | Somalia | 30 to 34 | 0.43 (-0.86,1.74) | 0.22 (-1.23,1.7) |
| Low SDI | Somalia | 35 to 39 | 0.18 (-1.01,1.38) | 0.03 (-1.3,1.38) |
| Low SDI | Somalia | 40 to 44 | -0.14 (-1.24,0.98) | -0.23 (-1.45,1) |
| Low SDI | Somalia | 45 to 49 | -0.37 (-1.44,0.72) | -0.45 (-1.62,0.73) |
| Low SDI | Somalia | 50 to 54 | -0.31 (-1.35,0.74) | -0.35 (-1.47,0.77) |
| Low SDI | Somalia | 55 to 59 | -0.29 (-1.3,0.73) | -0.3 (-1.36,0.76) |
| Low SDI | Somalia | 60 to 64 | -0.32 (-1.36,0.74) | -0.22 (-1.28,0.86) |
| Low SDI | Somalia | 65 to 69 | -0.2 (-1.41,1.02) | -0.19 (-1.39,1.01) |
| Low SDI | Somalia | 70 to 74 | -0.18 (-1.75,1.42) | -0.23 (-1.74,1.3) |
| Low SDI | Somalia | 75 to 79 | -0.15 (-2.43,2.19) | -0.25 (-2.35,1.9) |
| Low SDI | Somalia | 80 to 84 | -0.48 (-3.63,2.77) | -0.31 (-3.2,2.66) |
| Low SDI | Somalia | 85 to 89 | -0.02 (-6.32,6.69) | -0.74 (-5.52,4.29) |
| Low SDI | South Sudan | 5 to 9 | 1.32 (-0.37,3.05) | 1.23 (-0.6,3.1) |
| Low SDI | South Sudan | 10 to 14 | 1.21 (-0.39,2.83) | 1.07 (-0.69,2.86) |
| Low SDI | South Sudan | 15 to 19 | 1.09 (-0.6,2.8) | 0.94 (-0.94,2.85) |
| Low SDI | South Sudan | 20 to 24 | 1.15 (-0.6,2.93) | 0.83 (-1.15,2.85) |
| Low SDI | South Sudan | 25 to 29 | 1.28 (-0.44,3.03) | 0.99 (-0.99,3.01) |
| Low SDI | South Sudan | 30 to 34 | 1.23 (-0.42,2.9) | 0.97 (-0.93,2.91) |
| Low SDI | South Sudan | 35 to 39 | 1.09 (-0.47,2.68) | 0.68 (-1.12,2.52) |
| Low SDI | South Sudan | 40 to 44 | 0.87 (-0.62,2.39) | 0.58 (-1.13,2.31) |
| Low SDI | South Sudan | 45 to 49 | 0.72 (-0.71,2.17) | 0.41 (-1.18,2.02) |
| Low SDI | South Sudan | 50 to 54 | 0.52 (-0.85,1.9) | 0.3 (-1.19,1.8) |
| Low SDI | South Sudan | 55 to 59 | 0.3 (-1.01,1.64) | 0.12 (-1.28,1.54) |
| Low SDI | South Sudan | 60 to 64 | 0.13 (-1.17,1.44) | 0.02 (-1.33,1.38) |
| Low SDI | South Sudan | 65 to 69 | 0.04 (-1.28,1.38) | -0.18 (-1.51,1.16) |
| Low SDI | South Sudan | 70 to 74 | 0.04 (-1.36,1.46) | -0.2 (-1.57,1.18) |
| Low SDI | South Sudan | 75 to 79 | 0.22 (-1.46,1.93) | -0.02 (-1.62,1.6) |
| Low SDI | South Sudan | 80 to 84 | 0.57 (-1.75,2.95) | 0.47 (-1.68,2.67) |
| Low SDI | South Sudan | 85 to 89 | 1.58 (-2.92,6.29) | 1.06 (-2.72,5) |
| Low SDI | Timor-Leste | 5 to 9 | 0.38 (-7.64,9.1) | -7.82 (-25.08,13.41) |
| Low SDI | Timor-Leste | 10 to 14 | 3.64 (-5.07,13.15) | -0.27 (-15.01,17.01) |
| Low SDI | Timor-Leste | 15 to 19 | 6.92 (-2.97,17.82) | 5.53 (-6.38,18.95) |
| Low SDI | Timor-Leste | 20 to 24 | 9.95 (-0.81,21.88) | 6.41 (-5.71,20.09) |
| Low SDI | Timor-Leste | 25 to 29 | 11.55 (0.23,24.14) | 6.96 (-6.13,21.87) |
| Low SDI | Timor-Leste | 30 to 34 | 10.13 (-0.86,22.34) | 6.47 (-7.63,22.73) |
| Low SDI | Timor-Leste | 35 to 39 | 8.3 (-2.23,19.97) | 3.96 (-8.67,18.33) |
| Low SDI | Timor-Leste | 40 to 44 | 5.24 (-4.18,15.58) | 3.97 (-6.47,15.57) |
| Low SDI | Timor-Leste | 45 to 49 | 2.24 (-5.65,10.78) | 2 (-6.35,11.1) |
| Low SDI | Timor-Leste | 50 to 54 | 0.11 (-6.1,6.73) | -0.77 (-7.17,6.07) |
| Low SDI | Timor-Leste | 55 to 59 | 0.06 (-4.97,5.36) | -1.86 (-7.28,3.88) |
| Low SDI | Timor-Leste | 60 to 64 | -1.01 (-5.82,4.06) | -1.45 (-6.27,3.61) |
| Low SDI | Timor-Leste | 65 to 69 | -0.18 (-5.18,5.07) | -1.65 (-6.43,3.37) |
| Low SDI | Timor-Leste | 70 to 74 | 3.27 (-6.6,14.19) | -0.97 (-7.01,5.47) |
| Low SDI | Timor-Leste | 75 to 79 | 3.92 (-8.67,18.25) | 1.2 (-9.95,13.74) |
| Low SDI | Timor-Leste | 80 to 84 | 2.35 (-12.86,20.2) | 0.88 (-13.84,18.11) |
| Low SDI | Timor-Leste | 85 to 89 | -0.09 (-20.61,25.73) | -0.23 (-20.71,25.55) |
| Low SDI | Togo | 5 to 9 | 0.22 (-2.36,2.87) | -0.22 (-3.06,2.7) |
| Low SDI | Togo | 10 to 14 | 0.86 (-1.53,3.31) | 0.52 (-2.19,3.3) |
| Low SDI | Togo | 15 to 19 | 1.16 (-1.37,3.75) | 0.77 (-2.06,3.69) |
| Low SDI | Togo | 20 to 24 | 1.65 (-0.95,4.32) | 1.2 (-1.79,4.28) |
| Low SDI | Togo | 25 to 29 | 2.03 (-0.53,4.66) | 1.27 (-1.7,4.33) |
| Low SDI | Togo | 30 to 34 | 2.18 (-0.33,4.76) | 1.28 (-1.6,4.25) |
| Low SDI | Togo | 35 to 39 | 1.94 (-0.46,4.41) | 1.18 (-1.56,3.99) |
| Low SDI | Togo | 40 to 44 | 1.76 (-0.51,4.09) | 1.24 (-1.33,3.88) |
| Low SDI | Togo | 45 to 49 | 1.53 (-0.68,3.78) | 0.95 (-1.53,3.48) |
| Low SDI | Togo | 50 to 54 | 1.44 (-0.7,3.62) | 1.01 (-1.33,3.4) |
| Low SDI | Togo | 55 to 59 | 1.32 (-0.82,3.52) | 0.82 (-1.43,3.13) |
| Low SDI | Togo | 60 to 64 | 1.23 (-0.95,3.46) | 0.68 (-1.54,2.96) |
| Low SDI | Togo | 65 to 69 | 0.81 (-1.56,3.24) | 0.61 (-1.77,3.05) |
| Low SDI | Togo | 70 to 74 | 0.4 (-2.42,3.3) | 0.06 (-2.64,2.83) |
| Low SDI | Togo | 75 to 79 | 0.91 (-2.95,4.92) | -0.1 (-3.45,3.35) |
| Low SDI | Togo | 80 to 84 | 1.45 (-4.67,7.97) | 1.62 (-4.39,8) |
| Low SDI | Togo | 85 to 89 | 5.05 (-12.7,26.4) | 6.24 (-11.58,27.65) |
| Low SDI | Uganda | 5 to 9 | 0.45 (-0.25,1.16) | 0.01 (-0.74,0.77) |
| Low SDI | Uganda | 10 to 14 | 1.06 (0.42,1.7) | 0.57 (-0.13,1.28) |
| Low SDI | Uganda | 15 to 19 | 1.46 (0.8,2.11) | 0.89 (0.16,1.62) |
| Low SDI | Uganda | 20 to 24 | 1.67 (1,2.34) | 1.07 (0.31,1.84) |
| Low SDI | Uganda | 25 to 29 | 1.63 (0.95,2.31) | 0.99 (0.21,1.79) |
| Low SDI | Uganda | 30 to 34 | 1.53 (0.85,2.22) | 0.9 (0.1,1.71) |
| Low SDI | Uganda | 35 to 39 | 1.26 (0.58,1.94) | 0.71 (-0.08,1.5) |
| Low SDI | Uganda | 40 to 44 | 0.96 (0.3,1.63) | 0.5 (-0.26,1.26) |
| Low SDI | Uganda | 45 to 49 | 0.64 (0,1.29) | 0.22 (-0.49,0.94) |
| Low SDI | Uganda | 50 to 54 | 0.35 (-0.25,0.97) | -0.01 (-0.67,0.66) |
| Low SDI | Uganda | 55 to 59 | 0.12 (-0.46,0.7) | -0.19 (-0.81,0.44) |
| Low SDI | Uganda | 60 to 64 | 0.04 (-0.52,0.61) | -0.22 (-0.82,0.37) |
| Low SDI | Uganda | 65 to 69 | 0.04 (-0.55,0.63) | -0.17 (-0.76,0.43) |
| Low SDI | Uganda | 70 to 74 | 0.09 (-0.57,0.75) | -0.09 (-0.73,0.56) |
| Low SDI | Uganda | 75 to 79 | 0.14 (-0.67,0.95) | 0 (-0.76,0.76) |
| Low SDI | Uganda | 80 to 84 | 0.26 (-0.84,1.38) | 0.18 (-0.84,1.2) |
| Low SDI | Uganda | 85 to 89 | 0.53 (-1.5,2.59) | 0.39 (-1.4,2.22) |
| Low SDI | United Republic of Tanzania | 5 to 9 | -0.05 (-0.79,0.69) | -0.43 (-1.24,0.39) |
| Low SDI | United Republic of Tanzania | 10 to 14 | 0.21 (-0.47,0.88) | -0.22 (-0.97,0.53) |
| Low SDI | United Republic of Tanzania | 15 to 19 | 0.27 (-0.41,0.95) | -0.21 (-0.98,0.57) |
| Low SDI | United Republic of Tanzania | 20 to 24 | 0.32 (-0.37,1.01) | -0.19 (-0.99,0.62) |
| Low SDI | United Republic of Tanzania | 25 to 29 | 0.39 (-0.29,1.07) | -0.13 (-0.94,0.68) |
| Low SDI | United Republic of Tanzania | 30 to 34 | 0.46 (-0.21,1.14) | -0.03 (-0.83,0.78) |
| Low SDI | United Republic of Tanzania | 35 to 39 | 0.3 (-0.36,0.97) | -0.15 (-0.92,0.64) |
| Low SDI | United Republic of Tanzania | 40 to 44 | 0.11 (-0.53,0.76) | -0.31 (-1.05,0.44) |
| Low SDI | United Republic of Tanzania | 45 to 49 | -0.03 (-0.65,0.6) | -0.41 (-1.11,0.3) |
| Low SDI | United Republic of Tanzania | 50 to 54 | -0.12 (-0.72,0.47) | -0.46 (-1.11,0.2) |
| Low SDI | United Republic of Tanzania | 55 to 59 | -0.21 (-0.77,0.36) | -0.49 (-1.1,0.12) |
| Low SDI | United Republic of Tanzania | 60 to 64 | -0.26 (-0.8,0.29) | -0.49 (-1.06,0.09) |
| Low SDI | United Republic of Tanzania | 65 to 69 | -0.29 (-0.84,0.27) | -0.48 (-1.04,0.09) |
| Low SDI | United Republic of Tanzania | 70 to 74 | -0.28 (-0.9,0.34) | -0.43 (-1.04,0.17) |
| Low SDI | United Republic of Tanzania | 75 to 79 | -0.23 (-0.98,0.53) | -0.36 (-1.08,0.35) |
| Low SDI | United Republic of Tanzania | 80 to 84 | -0.13 (-1.17,0.93) | -0.24 (-1.18,0.72) |
| Low SDI | United Republic of Tanzania | 85 to 89 | 0.05 (-1.94,2.08) | -0.04 (-1.79,1.75) |
| Low SDI | Vanuatu | 5 to 9 | -2.92 (-23.75,23.6) | - |
| Low SDI | Vanuatu | 10 to 14 | -1 (-18.05,19.61) | - |
| Low SDI | Vanuatu | 15 to 19 | 0.9 (-14.65,19.28) | - |
| Low SDI | Vanuatu | 20 to 24 | 2.28 (-12.3,19.28) | - |
| Low SDI | Vanuatu | 25 to 29 | 3.29 (-10.54,19.25) | - |
| Low SDI | Vanuatu | 30 to 34 | 1.07 (-13.37,17.91) | - |
| Low SDI | Vanuatu | 35 to 39 | -1.87 (-16.73,15.63) | - |
| Low SDI | Vanuatu | 40 to 44 | -2.72 (-17.48,14.69) | - |
| Low SDI | Vanuatu | 45 to 49 | -3.43 (-18.09,13.84) | - |
| Low SDI | Vanuatu | 50 to 54 | -3.62 (-18.24,13.62) | - |
| Low SDI | Vanuatu | 55 to 59 | -3.5 (-18.14,13.77) | - |
| Low SDI | Vanuatu | 60 to 64 | -3.68 (-18.31,13.57) | - |
| Low SDI | Vanuatu | 65 to 69 | -3.39 (-18.17,14.07) | - |
| Low SDI | Vanuatu | 70 to 74 | -3.1 (-18.11,14.66) | - |
| Low SDI | Vanuatu | 75 to 79 | -3.19 (-18.64,15.2) | - |
| Low SDI | Vanuatu | 80 to 84 | -3.26 (-20.03,17.03) | - |
| Low SDI | Vanuatu | 85 to 89 | -3.78 (-24.45,22.54) | - |
| Low SDI | Yemen | 5 to 9 | -0.08 (-1.67,1.52) | -1.56 (-3.68,0.6) |
| Low SDI | Yemen | 10 to 14 | 0.4 (-1.08,1.91) | -1.05 (-2.96,0.89) |
| Low SDI | Yemen | 15 to 19 | 0.61 (-0.98,2.23) | -0.62 (-2.62,1.42) |
| Low SDI | Yemen | 20 to 24 | 0.71 (-0.94,2.38) | -0.45 (-2.49,1.64) |
| Low SDI | Yemen | 25 to 29 | 0.91 (-0.68,2.51) | -0.06 (-2.03,1.96) |
| Low SDI | Yemen | 30 to 34 | 0.83 (-0.63,2.32) | -0.06 (-1.89,1.8) |
| Low SDI | Yemen | 35 to 39 | 0.7 (-0.65,2.07) | -0.04 (-1.7,1.64) |
| Low SDI | Yemen | 40 to 44 | 0.56 (-0.72,1.86) | -0.2 (-1.74,1.37) |
| Low SDI | Yemen | 45 to 49 | 0.41 (-0.82,1.67) | -0.29 (-1.74,1.18) |
| Low SDI | Yemen | 50 to 54 | 0.28 (-0.92,1.49) | -0.38 (-1.75,1.01) |
| Low SDI | Yemen | 55 to 59 | 0.31 (-0.86,1.49) | -0.3 (-1.59,1.01) |
| Low SDI | Yemen | 60 to 64 | 0.33 (-0.83,1.5) | -0.12 (-1.36,1.14) |
| Low SDI | Yemen | 65 to 69 | 0.44 (-0.78,1.68) | 0.06 (-1.21,1.34) |
| Low SDI | Yemen | 70 to 74 | 0.73 (-0.73,2.21) | 0.32 (-1.12,1.78) |
| Low SDI | Yemen | 75 to 79 | 0.99 (-1.02,3.04) | 0.61 (-1.28,2.54) |
| Low SDI | Yemen | 80 to 84 | 1.37 (-1.87,4.71) | 0.55 (-2.19,3.36) |
| Low SDI | Yemen | 85 to 89 | 1.2 (-4.91,7.71) | 1.52 (-4.49,7.9) |
| Low SDI | Zimbabwe | 5 to 9 | 5.7 (3.93,7.51) | 5.77 (3.63,7.96) |
| Low SDI | Zimbabwe | 10 to 14 | 4.78 (3.36,6.22) | 4.97 (3.25,6.71) |
| Low SDI | Zimbabwe | 15 to 19 | 3.77 (2.57,4.99) | 4 (2.56,5.46) |
| Low SDI | Zimbabwe | 20 to 24 | 3.32 (2.24,4.42) | 3.61 (2.31,4.93) |
| Low SDI | Zimbabwe | 25 to 29 | 3.11 (2.13,4.1) | 3.39 (2.2,4.6) |
| Low SDI | Zimbabwe | 30 to 34 | 3.07 (2.17,3.98) | 3.3 (2.2,4.41) |
| Low SDI | Zimbabwe | 35 to 39 | 3.04 (2.19,3.9) | 3.24 (2.21,4.27) |
| Low SDI | Zimbabwe | 40 to 44 | 2.99 (2.16,3.83) | 3.16 (2.18,4.16) |
| Low SDI | Zimbabwe | 45 to 49 | 2.93 (2.1,3.77) | 3.07 (2.11,4.03) |
| Low SDI | Zimbabwe | 50 to 54 | 2.82 (1.99,3.66) | 2.97 (2.03,3.91) |
| Low SDI | Zimbabwe | 55 to 59 | 2.72 (1.89,3.56) | 2.86 (1.96,3.78) |
| Low SDI | Zimbabwe | 60 to 64 | 2.65 (1.81,3.5) | 2.77 (1.88,3.67) |
| Low SDI | Zimbabwe | 65 to 69 | 2.5 (1.64,3.37) | 2.68 (1.79,3.57) |
| Low SDI | Zimbabwe | 70 to 74 | 2.3 (1.36,3.25) | 2.43 (1.49,3.37) |
| Low SDI | Zimbabwe | 75 to 79 | 1.94 (0.79,3.09) | 2.06 (0.97,3.16) |
| Low SDI | Zimbabwe | 80 to 84 | 1.54 (-0.13,3.25) | 1.66 (0.12,3.22) |
| Low SDI | Zimbabwe | 85 to 89 | 1.46 (-1.73,4.75) | 1.62 (-1.25,4.58) |
| Low-middle SDI | Bangladesh | 5 to 9 | 0.41 (-0.09,0.92) | -1.89 (-2.61,-1.16) |
| Low-middle SDI | Bangladesh | 10 to 14 | 1.06 (0.61,1.52) | -1.06 (-1.67,-0.46) |
| Low-middle SDI | Bangladesh | 15 to 19 | 1.28 (0.79,1.78) | -0.68 (-1.31,-0.04) |
| Low-middle SDI | Bangladesh | 20 to 24 | 1.11 (0.58,1.64) | -0.75 (-1.42,-0.08) |
| Low-middle SDI | Bangladesh | 25 to 29 | 0.69 (0.16,1.23) | -1.08 (-1.75,-0.4) |
| Low-middle SDI | Bangladesh | 30 to 34 | 0.26 (-0.26,0.79) | -1.4 (-2.05,-0.73) |
| Low-middle SDI | Bangladesh | 35 to 39 | -0.22 (-0.72,0.29) | -1.71 (-2.34,-1.08) |
| Low-middle SDI | Bangladesh | 40 to 44 | -0.5 (-0.98,-0.02) | -1.84 (-2.42,-1.25) |
| Low-middle SDI | Bangladesh | 45 to 49 | -0.55 (-0.99,-0.1) | -1.72 (-2.24,-1.19) |
| Low-middle SDI | Bangladesh | 50 to 54 | -0.64 (-1.05,-0.23) | -1.67 (-2.13,-1.2) |
| Low-middle SDI | Bangladesh | 55 to 59 | -0.67 (-1.04,-0.29) | -1.58 (-2,-1.16) |
| Low-middle SDI | Bangladesh | 60 to 64 | -0.71 (-1.07,-0.35) | -1.5 (-1.89,-1.11) |
| Low-middle SDI | Bangladesh | 65 to 69 | -0.63 (-1.01,-0.25) | -1.32 (-1.71,-0.92) |
| Low-middle SDI | Bangladesh | 70 to 74 | -0.64 (-1.08,-0.2) | -1.23 (-1.66,-0.79) |
| Low-middle SDI | Bangladesh | 75 to 79 | -0.61 (-1.13,-0.09) | -1.08 (-1.57,-0.58) |
| Low-middle SDI | Bangladesh | 80 to 84 | -0.26 (-0.93,0.42) | -0.66 (-1.27,-0.04) |
| Low-middle SDI | Bangladesh | 85 to 89 | 0.38 (-0.77,1.55) | 0 (-1.02,1.04) |
| Low-middle SDI | Belize | 5 to 9 | -1.85 (-22.63,24.51) | -2.54 (-23.46,24.1) |
| Low-middle SDI | Belize | 10 to 14 | -1.96 (-18.48,17.9) | -2.34 (-19.28,18.14) |
| Low-middle SDI | Belize | 15 to 19 | -0.84 (-15.31,16.11) | -2.62 (-18.16,15.88) |
| Low-middle SDI | Belize | 20 to 24 | 3.03 (-10.14,18.12) | -2.71 (-17.78,15.12) |
| Low-middle SDI | Belize | 25 to 29 | 7.26 (-4.41,20.35) | -2.74 (-17.62,14.82) |
| Low-middle SDI | Belize | 30 to 34 | 7.86 (-3.18,20.15) | -4.21 (-18.6,12.73) |
| Low-middle SDI | Belize | 35 to 39 | 7.37 (-3.56,19.55) | -4.52 (-18.86,12.35) |
| Low-middle SDI | Belize | 40 to 44 | 6.05 (-4.45,17.71) | -2.36 (-16.94,14.77) |
| Low-middle SDI | Belize | 45 to 49 | 4.04 (-6.8,16.15) | -0.65 (-14.87,15.94) |
| Low-middle SDI | Belize | 50 to 54 | 6.75 (-5.02,19.98) | 0.79 (-12.73,16.4) |
| Low-middle SDI | Belize | 55 to 59 | 8.98 (-2.93,22.36) | 2.22 (-10.61,16.89) |
| Low-middle SDI | Belize | 60 to 64 | 9.11 (-3.04,22.78) | 0.67 (-12.93,16.4) |
| Low-middle SDI | Belize | 65 to 69 | 7.38 (-5.46,21.97) | -1.89 (-16.77,15.65) |
| Low-middle SDI | Belize | 70 to 74 | 3.19 (-10.72,19.27) | -2.43 (-17.51,15.4) |
| Low-middle SDI | Belize | 75 to 79 | -2.25 (-17.66,16.05) | -3 (-18.48,15.42) |
| Low-middle SDI | Belize | 80 to 84 | -2.96 (-19.71,17.29) | -3.19 (-19.98,17.12) |
| Low-middle SDI | Belize | 85 to 89 | -2.39 (-23.3,24.23) | -2.5 (-23.44,24.17) |
| Low-middle SDI | Bolivia (Plurinational State of) | 5 to 9 | -1.09 (-3.09,0.96) | -2.5 (-5.11,0.18) |
| Low-middle SDI | Bolivia (Plurinational State of) | 10 to 14 | -0.27 (-1.88,1.36) | -1.78 (-3.86,0.35) |
| Low-middle SDI | Bolivia (Plurinational State of) | 15 to 19 | 0.55 (-0.84,1.95) | -1.08 (-2.95,0.83) |
| Low-middle SDI | Bolivia (Plurinational State of) | 20 to 24 | 1.08 (-0.16,2.34) | -0.8 (-2.58,1.01) |
| Low-middle SDI | Bolivia (Plurinational State of) | 25 to 29 | 1.32 (0.2,2.45) | -0.78 (-2.47,0.95) |
| Low-middle SDI | Bolivia (Plurinational State of) | 30 to 34 | 1.35 (0.31,2.39) | -0.81 (-2.41,0.82) |
| Low-middle SDI | Bolivia (Plurinational State of) | 35 to 39 | 1.11 (0.15,2.09) | -0.93 (-2.41,0.58) |
| Low-middle SDI | Bolivia (Plurinational State of) | 40 to 44 | 1 (0.1,1.91) | -0.98 (-2.34,0.39) |
| Low-middle SDI | Bolivia (Plurinational State of) | 45 to 49 | 1.09 (0.26,1.93) | -0.74 (-1.96,0.5) |
| Low-middle SDI | Bolivia (Plurinational State of) | 50 to 54 | 1.28 (0.52,2.04) | -0.5 (-1.58,0.59) |
| Low-middle SDI | Bolivia (Plurinational State of) | 55 to 59 | 1.46 (0.76,2.16) | -0.31 (-1.28,0.66) |
| Low-middle SDI | Bolivia (Plurinational State of) | 60 to 64 | 1.63 (0.98,2.28) | -0.12 (-1,0.76) |
| Low-middle SDI | Bolivia (Plurinational State of) | 65 to 69 | 1.83 (1.19,2.48) | 0.1 (-0.73,0.94) |
| Low-middle SDI | Bolivia (Plurinational State of) | 70 to 74 | 2.04 (1.33,2.76) | 0.4 (-0.48,1.28) |
| Low-middle SDI | Bolivia (Plurinational State of) | 75 to 79 | 2.2 (1.25,3.17) | 0.68 (-0.39,1.76) |
| Low-middle SDI | Bolivia (Plurinational State of) | 80 to 84 | 2.44 (0.97,3.94) | 1.02 (-0.49,2.56) |
| Low-middle SDI | Bolivia (Plurinational State of) | 85 to 89 | 2.68 (-0.11,5.56) | 1.44 (-1.36,4.33) |
| Low-middle SDI | Cabo Verde | 5 to 9 | 6.27 (-3.17,16.63) | -3.46 (-22.39,20.08) |
| Low-middle SDI | Cabo Verde | 10 to 14 | 6.22 (-2.24,15.41) | -3.43 (-19.34,15.63) |
| Low-middle SDI | Cabo Verde | 15 to 19 | 5.47 (-2.89,14.54) | -1.98 (-17.02,15.79) |
| Low-middle SDI | Cabo Verde | 20 to 24 | 3.94 (-4.02,12.55) | 1.14 (-13.26,17.93) |
| Low-middle SDI | Cabo Verde | 25 to 29 | 2.84 (-4.72,11) | 1.64 (-11.69,16.97) |
| Low-middle SDI | Cabo Verde | 30 to 34 | 3.03 (-4.36,11) | 1.79 (-10.1,15.24) |
| Low-middle SDI | Cabo Verde | 35 to 39 | 3.77 (-3.56,11.65) | 5.03 (-4.86,15.95) |
| Low-middle SDI | Cabo Verde | 40 to 44 | 4.68 (-2.8,12.72) | 5.11 (-4.66,15.87) |
| Low-middle SDI | Cabo Verde | 45 to 49 | 5.97 (-2.07,14.67) | 4.72 (-5.32,15.82) |
| Low-middle SDI | Cabo Verde | 50 to 54 | 6 (-2.16,14.83) | 5.12 (-4.96,16.27) |
| Low-middle SDI | Cabo Verde | 55 to 59 | 6.16 (-1.72,14.68) | 4.88 (-4.19,14.82) |
| Low-middle SDI | Cabo Verde | 60 to 64 | 6.48 (-1.08,14.62) | 4.1 (-4.08,12.98) |
| Low-middle SDI | Cabo Verde | 65 to 69 | 11.12 (0.43,22.94) | 2.67 (-5.95,12.07) |
| Low-middle SDI | Cabo Verde | 70 to 74 | 12.12 (-0.43,26.26) | 6.93 (-4.73,20.01) |
| Low-middle SDI | Cabo Verde | 75 to 79 | 11.24 (-2.68,27.16) | 8.52 (-5.16,24.18) |
| Low-middle SDI | Cabo Verde | 80 to 84 | 7.47 (-8.5,26.24) | 7.8 (-8.21,26.6) |
| Low-middle SDI | Cabo Verde | 85 to 89 | -0.03 (-20.72,26.05) | 2.77 (-18.49,29.58) |
| Low-middle SDI | Congo | 5 to 9 | -0.46 (-4.98,4.28) | -1.9 (-7.05,3.53) |
| Low-middle SDI | Congo | 10 to 14 | 0.29 (-3.37,4.08) | -0.64 (-4.83,3.74) |
| Low-middle SDI | Congo | 15 to 19 | 0.53 (-2.68,3.83) | -0.1 (-3.66,3.6) |
| Low-middle SDI | Congo | 20 to 24 | 0.63 (-2.25,3.6) | -0.13 (-3.38,3.24) |
| Low-middle SDI | Congo | 25 to 29 | 0.8 (-1.72,3.38) | -0.25 (-3.2,2.8) |
| Low-middle SDI | Congo | 30 to 34 | 0.61 (-1.73,3) | -0.18 (-2.91,2.63) |
| Low-middle SDI | Congo | 35 to 39 | 0.53 (-1.66,2.77) | -0.61 (-3.16,2) |
| Low-middle SDI | Congo | 40 to 44 | 0.33 (-1.74,2.45) | -0.53 (-2.89,1.89) |
| Low-middle SDI | Congo | 45 to 49 | 0.1 (-1.91,2.15) | -0.66 (-2.9,1.63) |
| Low-middle SDI | Congo | 50 to 54 | 0.09 (-1.85,2.07) | -0.7 (-2.81,1.46) |
| Low-middle SDI | Congo | 55 to 59 | -0.16 (-2.05,1.78) | -0.72 (-2.74,1.35) |
| Low-middle SDI | Congo | 60 to 64 | -0.28 (-2.18,1.65) | -0.65 (-2.64,1.38) |
| Low-middle SDI | Congo | 65 to 69 | 0.02 (-2.05,2.14) | -0.55 (-2.63,1.57) |
| Low-middle SDI | Congo | 70 to 74 | -0.07 (-2.47,2.39) | -0.43 (-2.77,1.96) |
| Low-middle SDI | Congo | 75 to 79 | -0.42 (-3.45,2.7) | -0.3 (-3.3,2.8) |
| Low-middle SDI | Congo | 80 to 84 | 1.26 (-4.65,7.53) | -0.98 (-5.06,3.26) |
| Low-middle SDI | Congo | 85 to 89 | 5.32 (-12.34,26.52) | 4.16 (-13.17,24.95) |
| Low-middle SDI | Democratic People's Republic of Korea | 5 to 9 | -1.12 (-4.04,1.89) | -2.23 (-5.91,1.59) |
| Low-middle SDI | Democratic People's Republic of Korea | 10 to 14 | 0.02 (-1.9,1.98) | -1.39 (-3.85,1.13) |
| Low-middle SDI | Democratic People's Republic of Korea | 15 to 19 | 0.65 (-0.84,2.16) | -0.51 (-2.33,1.34) |
| Low-middle SDI | Democratic People's Republic of Korea | 20 to 24 | 1 (-0.27,2.28) | 0.14 (-1.35,1.66) |
| Low-middle SDI | Democratic People's Republic of Korea | 25 to 29 | 1.23 (0.12,2.35) | 0.4 (-0.93,1.76) |
| Low-middle SDI | Democratic People's Republic of Korea | 30 to 34 | 1.26 (0.25,2.27) | 0.48 (-0.73,1.72) |
| Low-middle SDI | Democratic People's Republic of Korea | 35 to 39 | 1.2 (0.28,2.12) | 0.48 (-0.61,1.59) |
| Low-middle SDI | Democratic People's Republic of Korea | 40 to 44 | 1.1 (0.29,1.93) | 0.46 (-0.5,1.44) |
| Low-middle SDI | Democratic People's Republic of Korea | 45 to 49 | 1.02 (0.32,1.73) | 0.41 (-0.4,1.23) |
| Low-middle SDI | Democratic People's Republic of Korea | 50 to 54 | 0.94 (0.31,1.57) | 0.34 (-0.36,1.05) |
| Low-middle SDI | Democratic People's Republic of Korea | 55 to 59 | 0.92 (0.32,1.51) | 0.36 (-0.29,1.01) |
| Low-middle SDI | Democratic People's Republic of Korea | 60 to 64 | 0.95 (0.33,1.56) | 0.43 (-0.22,1.08) |
| Low-middle SDI | Democratic People's Republic of Korea | 65 to 69 | 0.91 (0.23,1.59) | 0.44 (-0.26,1.14) |
| Low-middle SDI | Democratic People's Republic of Korea | 70 to 74 | 0.82 (0.01,1.63) | 0.41 (-0.38,1.2) |
| Low-middle SDI | Democratic People's Republic of Korea | 75 to 79 | 0.59 (-0.5,1.69) | 0.28 (-0.75,1.32) |
| Low-middle SDI | Democratic People's Republic of Korea | 80 to 84 | 0.4 (-1.25,2.08) | 0.08 (-1.42,1.6) |
| Low-middle SDI | Democratic People's Republic of Korea | 85 to 89 | -0.14 (-3,2.81) | -0.2 (-2.81,2.48) |
| Low-middle SDI | Dominican Republic | 5 to 9 | -0.08 (-2.52,2.41) | -0.7 (-4.37,3.12) |
| Low-middle SDI | Dominican Republic | 10 to 14 | 0.94 (-1.2,3.13) | 0.04 (-3.06,3.24) |
| Low-middle SDI | Dominican Republic | 15 to 19 | 2.16 (0.28,4.08) | 1.51 (-1.03,4.12) |
| Low-middle SDI | Dominican Republic | 20 to 24 | 2.43 (0.73,4.15) | 1.83 (-0.41,4.13) |
| Low-middle SDI | Dominican Republic | 25 to 29 | 2.45 (0.91,4.02) | 1.78 (-0.29,3.89) |
| Low-middle SDI | Dominican Republic | 30 to 34 | 2.2 (0.73,3.69) | 1.53 (-0.42,3.51) |
| Low-middle SDI | Dominican Republic | 35 to 39 | 2.07 (0.61,3.55) | 1.3 (-0.58,3.22) |
| Low-middle SDI | Dominican Republic | 40 to 44 | 2.01 (0.54,3.5) | 1.12 (-0.72,2.99) |
| Low-middle SDI | Dominican Republic | 45 to 49 | 1.99 (0.53,3.46) | 1.28 (-0.52,3.11) |
| Low-middle SDI | Dominican Republic | 50 to 54 | 1.9 (0.47,3.35) | 1.36 (-0.41,3.15) |
| Low-middle SDI | Dominican Republic | 55 to 59 | 1.8 (0.37,3.24) | 1.38 (-0.33,3.13) |
| Low-middle SDI | Dominican Republic | 60 to 64 | 1.66 (0.24,3.1) | 1.45 (-0.23,3.15) |
| Low-middle SDI | Dominican Republic | 65 to 69 | 1.51 (0.01,3.03) | 1.37 (-0.35,3.12) |
| Low-middle SDI | Dominican Republic | 70 to 74 | 1.41 (-0.3,3.14) | 1.03 (-0.82,2.92) |
| Low-middle SDI | Dominican Republic | 75 to 79 | 1.49 (-0.63,3.66) | 1.13 (-1.05,3.37) |
| Low-middle SDI | Dominican Republic | 80 to 84 | 1.71 (-1.15,4.66) | 1.6 (-1.28,4.57) |
| Low-middle SDI | Dominican Republic | 85 to 89 | 1.69 (-2.87,6.47) | 1.87 (-2.7,6.65) |
| Low-middle SDI | Egypt | 5 to 9 | -2.1 (-3,-1.18) | -4.11 (-5.6,-2.59) |
| Low-middle SDI | Egypt | 10 to 14 | -0.83 (-1.58,-0.07) | -2.78 (-3.92,-1.62) |
| Low-middle SDI | Egypt | 15 to 19 | 0.18 (-0.52,0.88) | -1.76 (-2.75,-0.75) |
| Low-middle SDI | Egypt | 20 to 24 | 0.48 (-0.2,1.15) | -1.42 (-2.36,-0.47) |
| Low-middle SDI | Egypt | 25 to 29 | 0.44 (-0.2,1.08) | -1.39 (-2.28,-0.49) |
| Low-middle SDI | Egypt | 30 to 34 | 0.21 (-0.38,0.8) | -1.48 (-2.3,-0.65) |
| Low-middle SDI | Egypt | 35 to 39 | -0.25 (-0.8,0.31) | -1.74 (-2.5,-0.98) |
| Low-middle SDI | Egypt | 40 to 44 | -0.51 (-1.04,0.01) | -1.84 (-2.53,-1.14) |
| Low-middle SDI | Egypt | 45 to 49 | -0.33 (-0.83,0.17) | -1.52 (-2.16,-0.88) |
| Low-middle SDI | Egypt | 50 to 54 | 0.47 (-0.02,0.96) | -0.6 (-1.2,-0.01) |
| Low-middle SDI | Egypt | 55 to 59 | 1.12 (0.64,1.61) | 0.14 (-0.43,0.71) |
| Low-middle SDI | Egypt | 60 to 64 | 1.41 (0.9,1.92) | 0.52 (-0.04,1.09) |
| Low-middle SDI | Egypt | 65 to 69 | 1.52 (0.96,2.09) | 0.74 (0.13,1.35) |
| Low-middle SDI | Egypt | 70 to 74 | 1.37 (0.68,2.06) | 0.69 (-0.01,1.4) |
| Low-middle SDI | Egypt | 75 to 79 | 1.31 (0.39,2.25) | 0.72 (-0.18,1.63) |
| Low-middle SDI | Egypt | 80 to 84 | 1.47 (0.1,2.85) | 0.95 (-0.32,2.23) |
| Low-middle SDI | Egypt | 85 to 89 | 1.31 (-1,3.67) | 1.09 (-1.08,3.32) |
| Low-middle SDI | El Salvador | 5 to 9 | 1.44 (-1.89,4.88) | -2.11 (-8.24,4.44) |
| Low-middle SDI | El Salvador | 10 to 14 | 2.3 (-0.35,5.01) | 0.09 (-3.83,4.16) |
| Low-middle SDI | El Salvador | 15 to 19 | 2.28 (-0.03,4.63) | 0.01 (-3.38,3.52) |
| Low-middle SDI | El Salvador | 20 to 24 | 2.46 (0.38,4.59) | -0.04 (-3.14,3.16) |
| Low-middle SDI | El Salvador | 25 to 29 | 2.61 (0.67,4.58) | 0.56 (-2.38,3.59) |
| Low-middle SDI | El Salvador | 30 to 34 | 2.76 (0.92,4.64) | 0.96 (-1.78,3.79) |
| Low-middle SDI | El Salvador | 35 to 39 | 2.96 (1.21,4.74) | 1.11 (-1.44,3.72) |
| Low-middle SDI | El Salvador | 40 to 44 | 3.03 (1.35,4.73) | 1.33 (-1.04,3.75) |
| Low-middle SDI | El Salvador | 45 to 49 | 2.9 (1.3,4.53) | 1.28 (-0.93,3.53) |
| Low-middle SDI | El Salvador | 50 to 54 | 2.76 (1.24,4.31) | 1.25 (-0.81,3.36) |
| Low-middle SDI | El Salvador | 55 to 59 | 2.58 (1.1,4.07) | 1.11 (-0.82,3.08) |
| Low-middle SDI | El Salvador | 60 to 64 | 2.3 (0.86,3.75) | 1 (-0.83,2.86) |
| Low-middle SDI | El Salvador | 65 to 69 | 1.99 (0.52,3.48) | 0.65 (-1.13,2.46) |
| Low-middle SDI | El Salvador | 70 to 74 | 1.93 (0.31,3.57) | 0.63 (-1.2,2.49) |
| Low-middle SDI | El Salvador | 75 to 79 | 1.92 (-0.04,3.9) | 0.79 (-1.3,2.94) |
| Low-middle SDI | El Salvador | 80 to 84 | 1.69 (-0.93,4.38) | 0.89 (-1.79,3.65) |
| Low-middle SDI | El Salvador | 85 to 89 | 1.98 (-2.49,6.67) | 1.55 (-2.96,6.28) |
| Low-middle SDI | Eswatini | 5 to 9 | 1.06 (-6.19,8.87) | 0.67 (-6.64,8.56) |
| Low-middle SDI | Eswatini | 10 to 14 | 0.91 (-4.51,6.65) | -0.19 (-5.87,5.85) |
| Low-middle SDI | Eswatini | 15 to 19 | 1.21 (-3.18,5.79) | -0.63 (-5.62,4.63) |
| Low-middle SDI | Eswatini | 20 to 24 | 1.3 (-2.6,5.37) | 0.1 (-4.47,4.9) |
| Low-middle SDI | Eswatini | 25 to 29 | 1.95 (-1.59,5.61) | 1.56 (-2.54,5.83) |
| Low-middle SDI | Eswatini | 30 to 34 | 2.44 (-0.79,5.77) | 2.23 (-1.51,6.11) |
| Low-middle SDI | Eswatini | 35 to 39 | 2.43 (-0.55,5.5) | 2.4 (-1.14,6.07) |
| Low-middle SDI | Eswatini | 40 to 44 | 2.23 (-0.62,5.17) | 2.21 (-1.18,5.72) |
| Low-middle SDI | Eswatini | 45 to 49 | 2.08 (-0.74,4.98) | 1.83 (-1.49,5.26) |
| Low-middle SDI | Eswatini | 50 to 54 | 1.63 (-1.23,4.59) | 1.58 (-1.63,4.9) |
| Low-middle SDI | Eswatini | 55 to 59 | 1.91 (-1.11,5.03) | 1.19 (-2.03,4.51) |
| Low-middle SDI | Eswatini | 60 to 64 | 1.8 (-1.44,5.16) | 1 (-2.51,4.63) |
| Low-middle SDI | Eswatini | 65 to 69 | 1.14 (-2.34,4.74) | 1.23 (-2.66,5.27) |
| Low-middle SDI | Eswatini | 70 to 74 | 1.16 (-3.17,5.68) | 1.53 (-3,6.26) |
| Low-middle SDI | Eswatini | 75 to 79 | 0.34 (-4.9,5.87) | 0.93 (-4.37,6.53) |
| Low-middle SDI | Eswatini | 80 to 84 | 3.03 (-10.38,18.43) | -0.54 (-7.44,6.87) |
| Low-middle SDI | Eswatini | 85 to 89 | 1.85 (-18.53,27.33) | -1.65 (-19.67,20.4) |
| Low-middle SDI | Ghana | 5 to 9 | -5.49 (-6.66,-4.3) | -5.36 (-6.66,-4.06) |
| Low-middle SDI | Ghana | 10 to 14 | -5.59 (-6.63,-4.54) | -5.44 (-6.6,-4.27) |
| Low-middle SDI | Ghana | 15 to 19 | -5.27 (-6.34,-4.19) | -5.4 (-6.63,-4.16) |
| Low-middle SDI | Ghana | 20 to 24 | -4.38 (-5.42,-3.32) | -4.81 (-6.05,-3.55) |
| Low-middle SDI | Ghana | 25 to 29 | -3.45 (-4.42,-2.47) | -4.01 (-5.19,-2.82) |
| Low-middle SDI | Ghana | 30 to 34 | -2.65 (-3.54,-1.75) | -3.29 (-4.37,-2.19) |
| Low-middle SDI | Ghana | 35 to 39 | -1.88 (-2.7,-1.04) | -2.43 (-3.43,-1.42) |
| Low-middle SDI | Ghana | 40 to 44 | -1.29 (-2.09,-0.49) | -1.71 (-2.66,-0.76) |
| Low-middle SDI | Ghana | 45 to 49 | -0.88 (-1.67,-0.08) | -1.22 (-2.13,-0.3) |
| Low-middle SDI | Ghana | 50 to 54 | -0.56 (-1.34,0.22) | -0.83 (-1.7,0.05) |
| Low-middle SDI | Ghana | 55 to 59 | -0.35 (-1.12,0.42) | -0.59 (-1.43,0.26) |
| Low-middle SDI | Ghana | 60 to 64 | -0.04 (-0.81,0.74) | -0.3 (-1.11,0.53) |
| Low-middle SDI | Ghana | 65 to 69 | 0.33 (-0.54,1.21) | 0.04 (-0.85,0.93) |
| Low-middle SDI | Ghana | 70 to 74 | 0.46 (-0.67,1.6) | 0.25 (-0.86,1.37) |
| Low-middle SDI | Ghana | 75 to 79 | 0.12 (-1.4,1.67) | 0 (-1.44,1.47) |
| Low-middle SDI | Ghana | 80 to 84 | -0.61 (-3.12,1.97) | -0.54 (-2.88,1.86) |
| Low-middle SDI | Ghana | 85 to 89 | -0.14 (-6.11,6.22) | -1.56 (-5.98,3.08) |
| Low-middle SDI | Guatemala | 5 to 9 | 0.08 (-2.78,3.03) | -1.61 (-5.58,2.52) |
| Low-middle SDI | Guatemala | 10 to 14 | 0.57 (-1.79,2.99) | -0.59 (-3.61,2.54) |
| Low-middle SDI | Guatemala | 15 to 19 | 1 (-1.08,3.12) | -0.07 (-2.72,2.66) |
| Low-middle SDI | Guatemala | 20 to 24 | 1.14 (-0.81,3.13) | 0.26 (-2.26,2.84) |
| Low-middle SDI | Guatemala | 25 to 29 | 1.09 (-0.78,2.99) | 0.1 (-2.37,2.64) |
| Low-middle SDI | Guatemala | 30 to 34 | 1.11 (-0.69,2.95) | 0.26 (-2.11,2.68) |
| Low-middle SDI | Guatemala | 35 to 39 | 1.1 (-0.64,2.88) | 0.07 (-2.16,2.34) |
| Low-middle SDI | Guatemala | 40 to 44 | 1.28 (-0.43,3.02) | 0.17 (-1.95,2.34) |
| Low-middle SDI | Guatemala | 45 to 49 | 1.56 (-0.11,3.27) | 0.47 (-1.57,2.56) |
| Low-middle SDI | Guatemala | 50 to 54 | 1.86 (0.22,3.52) | 0.9 (-1.07,2.9) |
| Low-middle SDI | Guatemala | 55 to 59 | 2.01 (0.41,3.64) | 1.15 (-0.74,3.07) |
| Low-middle SDI | Guatemala | 60 to 64 | 2.06 (0.48,3.66) | 1.36 (-0.44,3.2) |
| Low-middle SDI | Guatemala | 65 to 69 | 1.78 (0.15,3.44) | 1.02 (-0.77,2.84) |
| Low-middle SDI | Guatemala | 70 to 74 | 1.3 (-0.53,3.16) | 0.6 (-1.31,2.54) |
| Low-middle SDI | Guatemala | 75 to 79 | 1.06 (-1.29,3.46) | 0 (-2.24,2.3) |
| Low-middle SDI | Guatemala | 80 to 84 | -0.04 (-3.06,3.07) | -0.46 (-3.45,2.61) |
| Low-middle SDI | Guatemala | 85 to 89 | 0.43 (-5.51,6.75) | -1.46 (-5.93,3.23) |
| Low-middle SDI | Honduras | 5 to 9 | -4.11 (-8.62,0.62) | -5.05 (-11.12,1.44) |
| Low-middle SDI | Honduras | 10 to 14 | -2.36 (-5.83,1.25) | -3.07 (-7.43,1.48) |
| Low-middle SDI | Honduras | 15 to 19 | -1.6 (-4.75,1.64) | -2.36 (-6.34,1.79) |
| Low-middle SDI | Honduras | 20 to 24 | -1.51 (-4.48,1.55) | -1.85 (-5.61,2.06) |
| Low-middle SDI | Honduras | 25 to 29 | -1.38 (-4.18,1.5) | -2 (-5.64,1.78) |
| Low-middle SDI | Honduras | 30 to 34 | -1.25 (-3.84,1.42) | -1.87 (-5.25,1.64) |
| Low-middle SDI | Honduras | 35 to 39 | -0.61 (-3.01,1.85) | -1.41 (-4.48,1.77) |
| Low-middle SDI | Honduras | 40 to 44 | 0.12 (-2.12,2.41) | -0.48 (-3.26,2.37) |
| Low-middle SDI | Honduras | 45 to 49 | 0.89 (-1.23,3.05) | 0.26 (-2.3,2.88) |
| Low-middle SDI | Honduras | 50 to 54 | 1.43 (-0.57,3.47) | 0.98 (-1.37,3.39) |
| Low-middle SDI | Honduras | 55 to 59 | 1.91 (-0.01,3.87) | 1.53 (-0.71,3.82) |
| Low-middle SDI | Honduras | 60 to 64 | 2.22 (0.29,4.19) | 1.82 (-0.36,4.06) |
| Low-middle SDI | Honduras | 65 to 69 | 2.47 (0.43,4.56) | 2.04 (-0.18,4.31) |
| Low-middle SDI | Honduras | 70 to 74 | 2.38 (0.13,4.68) | 2.3 (-0.11,4.76) |
| Low-middle SDI | Honduras | 75 to 79 | 2.45 (-0.42,5.41) | 1.95 (-0.88,4.85) |
| Low-middle SDI | Honduras | 80 to 84 | 2.01 (-1.9,6.08) | 1.88 (-2.01,5.93) |
| Low-middle SDI | Honduras | 85 to 89 | 1.37 (-5.01,8.19) | 1.88 (-4.5,8.68) |
| Low-middle SDI | India | 5 to 9 | -2 (-2.41,-1.58) | -3.85 (-4.4,-3.29) |
| Low-middle SDI | India | 10 to 14 | -0.55 (-0.86,-0.23) | -2.25 (-2.64,-1.85) |
| Low-middle SDI | India | 15 to 19 | -0.24 (-0.54,0.06) | -1.76 (-2.12,-1.39) |
| Low-middle SDI | India | 20 to 24 | 0.09 (-0.2,0.38) | -1.35 (-1.69,-1) |
| Low-middle SDI | India | 25 to 29 | 0.54 (0.27,0.81) | -0.88 (-1.21,-0.56) |
| Low-middle SDI | India | 30 to 34 | 0.65 (0.4,0.91) | -0.74 (-1.04,-0.44) |
| Low-middle SDI | India | 35 to 39 | 0.73 (0.49,0.97) | -0.57 (-0.84,-0.29) |
| Low-middle SDI | India | 40 to 44 | 0.9 (0.67,1.12) | -0.3 (-0.56,-0.05) |
| Low-middle SDI | India | 45 to 49 | 0.84 (0.63,1.05) | -0.25 (-0.48,-0.02) |
| Low-middle SDI | India | 50 to 54 | 0.71 (0.51,0.9) | -0.26 (-0.47,-0.05) |
| Low-middle SDI | India | 55 to 59 | 0.67 (0.49,0.85) | -0.18 (-0.37,0.01) |
| Low-middle SDI | India | 60 to 64 | 0.44 (0.27,0.61) | -0.29 (-0.47,-0.12) |
| Low-middle SDI | India | 65 to 69 | 0.44 (0.26,0.62) | -0.17 (-0.34,0.01) |
| Low-middle SDI | India | 70 to 74 | 0.62 (0.41,0.84) | 0.14 (-0.06,0.33) |
| Low-middle SDI | India | 75 to 79 | 0.99 (0.7,1.27) | 0.6 (0.35,0.86) |
| Low-middle SDI | India | 80 to 84 | 1.31 (0.89,1.72) | 0.99 (0.64,1.35) |
| Low-middle SDI | India | 85 to 89 | 1.56 (0.81,2.32) | 1.25 (0.62,1.88) |
| Low-middle SDI | Kenya | 5 to 9 | 1.37 (-0.04,2.79) | 0.95 (-0.64,2.56) |
| Low-middle SDI | Kenya | 10 to 14 | 1.64 (0.46,2.83) | 1.25 (-0.1,2.62) |
| Low-middle SDI | Kenya | 15 to 19 | 1.7 (0.59,2.82) | 1.4 (0.09,2.72) |
| Low-middle SDI | Kenya | 20 to 24 | 1.66 (0.59,2.74) | 1.36 (0.07,2.67) |
| Low-middle SDI | Kenya | 25 to 29 | 1.68 (0.65,2.72) | 1.37 (0.1,2.65) |
| Low-middle SDI | Kenya | 30 to 34 | 1.77 (0.78,2.77) | 1.39 (0.16,2.63) |
| Low-middle SDI | Kenya | 35 to 39 | 1.76 (0.8,2.74) | 1.42 (0.25,2.61) |
| Low-middle SDI | Kenya | 40 to 44 | 1.83 (0.89,2.78) | 1.49 (0.37,2.62) |
| Low-middle SDI | Kenya | 45 to 49 | 1.95 (1.03,2.88) | 1.63 (0.57,2.7) |
| Low-middle SDI | Kenya | 50 to 54 | 2 (1.11,2.9) | 1.75 (0.75,2.76) |
| Low-middle SDI | Kenya | 55 to 59 | 2.09 (1.24,2.95) | 1.87 (0.94,2.81) |
| Low-middle SDI | Kenya | 60 to 64 | 2.17 (1.34,3.01) | 2.02 (1.14,2.91) |
| Low-middle SDI | Kenya | 65 to 69 | 2.29 (1.43,3.16) | 2.16 (1.28,3.05) |
| Low-middle SDI | Kenya | 70 to 74 | 2.43 (1.44,3.43) | 2.31 (1.33,3.29) |
| Low-middle SDI | Kenya | 75 to 79 | 2.55 (1.26,3.85) | 2.39 (1.18,3.61) |
| Low-middle SDI | Kenya | 80 to 84 | 2.31 (0.56,4.08) | 2.29 (0.68,3.92) |
| Low-middle SDI | Kenya | 85 to 89 | 2.05 (-0.87,5.05) | 2.22 (-0.45,4.96) |
| Low-middle SDI | Kyrgyzstan | 5 to 9 | 0.39 (-2.93,3.83) | -1.8 (-7.32,4.05) |
| Low-middle SDI | Kyrgyzstan | 10 to 14 | 0.1 (-2.85,3.13) | -3.3 (-8.29,1.95) |
| Low-middle SDI | Kyrgyzstan | 15 to 19 | -0.13 (-2.95,2.78) | -2.58 (-6.95,2) |
| Low-middle SDI | Kyrgyzstan | 20 to 24 | 0.02 (-2.57,2.68) | -1.78 (-5.48,2.08) |
| Low-middle SDI | Kyrgyzstan | 25 to 29 | 0.28 (-2.08,2.71) | -1.38 (-4.81,2.18) |
| Low-middle SDI | Kyrgyzstan | 30 to 34 | 0.46 (-1.75,2.71) | -1.22 (-4.37,2.03) |
| Low-middle SDI | Kyrgyzstan | 35 to 39 | 0.44 (-1.7,2.63) | -0.86 (-3.84,2.22) |
| Low-middle SDI | Kyrgyzstan | 40 to 44 | 0.04 (-2.06,2.2) | -1.14 (-3.99,1.79) |
| Low-middle SDI | Kyrgyzstan | 45 to 49 | -0.43 (-2.56,1.74) | -1.18 (-4.01,1.72) |
| Low-middle SDI | Kyrgyzstan | 50 to 54 | -0.48 (-2.59,1.68) | -1.12 (-3.83,1.67) |
| Low-middle SDI | Kyrgyzstan | 55 to 59 | -0.21 (-2.26,1.88) | -0.93 (-3.47,1.68) |
| Low-middle SDI | Kyrgyzstan | 60 to 64 | -0.15 (-2.21,1.94) | -1.03 (-3.47,1.46) |
| Low-middle SDI | Kyrgyzstan | 65 to 69 | 0.41 (-1.89,2.77) | -0.45 (-3.01,2.19) |
| Low-middle SDI | Kyrgyzstan | 70 to 74 | 0.41 (-2.51,3.42) | -0.09 (-3.31,3.24) |
| Low-middle SDI | Kyrgyzstan | 75 to 79 | 0.52 (-3.47,4.67) | 0.11 (-4.02,4.43) |
| Low-middle SDI | Kyrgyzstan | 80 to 84 | -0.27 (-5.35,5.07) | -0.41 (-5.53,4.99) |
| Low-middle SDI | Kyrgyzstan | 85 to 89 | -1.32 (-8.37,6.27) | -1.46 (-8.52,6.15) |
| Low-middle SDI | Lao People's Democratic Republic | 5 to 9 | -1.08 (-4.56,2.53) | -1.87 (-5.86,2.28) |
| Low-middle SDI | Lao People's Democratic Republic | 10 to 14 | -0.17 (-2.79,2.52) | -1.44 (-4.5,1.71) |
| Low-middle SDI | Lao People's Democratic Republic | 15 to 19 | 0.1 (-2.26,2.51) | -0.68 (-3.26,1.97) |
| Low-middle SDI | Lao People's Democratic Republic | 20 to 24 | 0.36 (-1.89,2.67) | -0.36 (-2.8,2.14) |
| Low-middle SDI | Lao People's Democratic Republic | 25 to 29 | 0.36 (-1.82,2.59) | -0.13 (-2.56,2.35) |
| Low-middle SDI | Lao People's Democratic Republic | 30 to 34 | 0.1 (-2.03,2.27) | -0.23 (-2.63,2.22) |
| Low-middle SDI | Lao People's Democratic Republic | 35 to 39 | -0.19 (-2.25,1.91) | -0.46 (-2.78,1.92) |
| Low-middle SDI | Lao People's Democratic Republic | 40 to 44 | -0.53 (-2.49,1.47) | -0.81 (-2.99,1.43) |
| Low-middle SDI | Lao People's Democratic Republic | 45 to 49 | -0.5 (-2.36,1.4) | -1.01 (-3.01,1.03) |
| Low-middle SDI | Lao People's Democratic Republic | 50 to 54 | -0.45 (-2.18,1.31) | -1.01 (-2.84,0.86) |
| Low-middle SDI | Lao People's Democratic Republic | 55 to 59 | -0.35 (-1.99,1.31) | -0.79 (-2.49,0.94) |
| Low-middle SDI | Lao People's Democratic Republic | 60 to 64 | -0.28 (-1.9,1.37) | -0.64 (-2.28,1.03) |
| Low-middle SDI | Lao People's Democratic Republic | 65 to 69 | -0.05 (-1.75,1.68) | -0.36 (-2.04,1.35) |
| Low-middle SDI | Lao People's Democratic Republic | 70 to 74 | 0.12 (-1.83,2.11) | -0.13 (-1.99,1.76) |
| Low-middle SDI | Lao People's Democratic Republic | 75 to 79 | 0.61 (-2.06,3.35) | 0.16 (-2.26,2.63) |
| Low-middle SDI | Lao People's Democratic Republic | 80 to 84 | 0.33 (-3.55,4.37) | 0.78 (-3,4.7) |
| Low-middle SDI | Lao People's Democratic Republic | 85 to 89 | 5.55 (-11.89,26.45) | 0.03 (-6.25,6.73) |
| Low-middle SDI | Lesotho | 5 to 9 | 4.33 (-1.65,10.68) | 1.01 (-6.24,8.82) |
| Low-middle SDI | Lesotho | 10 to 14 | 4.08 (-0.65,9.04) | 1.55 (-4,7.42) |
| Low-middle SDI | Lesotho | 15 to 19 | 3.86 (-0.29,8.19) | 2.25 (-2.39,7.11) |
| Low-middle SDI | Lesotho | 20 to 24 | 3.55 (-0.34,7.59) | 3.15 (-1.25,7.75) |
| Low-middle SDI | Lesotho | 25 to 29 | 3.61 (0.05,7.31) | 3.84 (-0.19,8.04) |
| Low-middle SDI | Lesotho | 30 to 34 | 3.96 (0.73,7.29) | 4.03 (0.34,7.85) |
| Low-middle SDI | Lesotho | 35 to 39 | 4.12 (1.22,7.11) | 4.07 (0.7,7.56) |
| Low-middle SDI | Lesotho | 40 to 44 | 3.82 (1.13,6.58) | 4.15 (1.12,7.28) |
| Low-middle SDI | Lesotho | 45 to 49 | 3.48 (0.95,6.07) | 3.78 (0.95,6.7) |
| Low-middle SDI | Lesotho | 50 to 54 | 3.32 (0.88,5.81) | 3.58 (0.87,6.37) |
| Low-middle SDI | Lesotho | 55 to 59 | 3.09 (0.67,5.57) | 3.39 (0.71,6.13) |
| Low-middle SDI | Lesotho | 60 to 64 | 3.06 (0.57,5.62) | 2.96 (0.27,5.73) |
| Low-middle SDI | Lesotho | 65 to 69 | 2.99 (0.33,5.73) | 2.61 (-0.09,5.38) |
| Low-middle SDI | Lesotho | 70 to 74 | 2.35 (-0.58,5.36) | 2.28 (-0.65,5.29) |
| Low-middle SDI | Lesotho | 75 to 79 | 2.06 (-1.52,5.78) | 1.7 (-1.67,5.17) |
| Low-middle SDI | Lesotho | 80 to 84 | 2.45 (-2.68,7.84) | 0.9 (-3.49,5.48) |
| Low-middle SDI | Lesotho | 85 to 89 | 1.41 (-5.76,9.12) | 0.3 (-6.57,7.68) |
| Low-middle SDI | Mauritania | 5 to 9 | 0.92 (-2.61,4.58) | -0.86 (-4.69,3.13) |
| Low-middle SDI | Mauritania | 10 to 14 | 1.37 (-2.09,4.96) | -0.46 (-4.22,3.44) |
| Low-middle SDI | Mauritania | 15 to 19 | 1.04 (-2.71,4.94) | -0.21 (-4.38,4.15) |
| Low-middle SDI | Mauritania | 20 to 24 | 1.2 (-2.69,5.25) | -0.78 (-5.23,3.87) |
| Low-middle SDI | Mauritania | 25 to 29 | 1.52 (-2.42,5.61) | -0.45 (-4.87,4.18) |
| Low-middle SDI | Mauritania | 30 to 34 | 1.34 (-2.6,5.43) | -0.71 (-5.06,3.85) |
| Low-middle SDI | Mauritania | 35 to 39 | 1.78 (-1.99,5.7) | -0.42 (-4.68,4.03) |
| Low-middle SDI | Mauritania | 40 to 44 | 1.85 (-1.7,5.54) | -0.33 (-4.44,3.96) |
| Low-middle SDI | Mauritania | 45 to 49 | 1.51 (-1.88,5.02) | 0.18 (-3.62,4.14) |
| Low-middle SDI | Mauritania | 50 to 54 | 0.82 (-2.33,4.06) | -0.09 (-3.54,3.48) |
| Low-middle SDI | Mauritania | 55 to 59 | 0.77 (-2.18,3.81) | -0.21 (-3.31,2.99) |
| Low-middle SDI | Mauritania | 60 to 64 | 0.54 (-2.23,3.38) | -0.44 (-3.3,2.51) |
| Low-middle SDI | Mauritania | 65 to 69 | 0.16 (-2.67,3.06) | -0.75 (-3.59,2.18) |
| Low-middle SDI | Mauritania | 70 to 74 | -0.58 (-3.58,2.51) | -0.92 (-3.92,2.18) |
| Low-middle SDI | Mauritania | 75 to 79 | -0.64 (-4.18,3.04) | -0.79 (-4.11,2.64) |
| Low-middle SDI | Mauritania | 80 to 84 | 0.36 (-5.74,6.85) | -0.58 (-5.44,4.54) |
| Low-middle SDI | Mauritania | 85 to 89 | 4.85 (-12.77,26.02) | 4.68 (-12.85,25.74) |
| Low-middle SDI | Mongolia | 5 to 9 | -0.92 (-5.2,3.54) | -3.55 (-10,3.35) |
| Low-middle SDI | Mongolia | 10 to 14 | -0.2 (-3.83,3.57) | -2.67 (-7.76,2.69) |
| Low-middle SDI | Mongolia | 15 to 19 | 0.1 (-3.29,3.61) | -2.93 (-7.47,1.83) |
| Low-middle SDI | Mongolia | 20 to 24 | -0.88 (-4.18,2.53) | -3.34 (-7.56,1.06) |
| Low-middle SDI | Mongolia | 25 to 29 | -0.83 (-3.85,2.28) | -3.03 (-6.9,1) |
| Low-middle SDI | Mongolia | 30 to 34 | -0.41 (-3.22,2.48) | -2.09 (-5.55,1.49) |
| Low-middle SDI | Mongolia | 35 to 39 | -0.14 (-2.89,2.68) | -2.13 (-5.5,1.37) |
| Low-middle SDI | Mongolia | 40 to 44 | -0.43 (-3.18,2.39) | -2.06 (-5.28,1.27) |
| Low-middle SDI | Mongolia | 45 to 49 | -0.52 (-3.26,2.29) | -1.9 (-5.07,1.37) |
| Low-middle SDI | Mongolia | 50 to 54 | -0.94 (-3.58,1.76) | -1.84 (-4.87,1.29) |
| Low-middle SDI | Mongolia | 55 to 59 | -0.86 (-3.55,1.9) | -1.96 (-4.87,1.05) |
| Low-middle SDI | Mongolia | 60 to 64 | -0.94 (-3.84,2.04) | -2.04 (-5.08,1.11) |
| Low-middle SDI | Mongolia | 65 to 69 | -1.02 (-4.31,2.39) | -2.34 (-5.7,1.14) |
| Low-middle SDI | Mongolia | 70 to 74 | -0.74 (-4.92,3.62) | -2.47 (-6.48,1.71) |
| Low-middle SDI | Mongolia | 75 to 79 | -1.13 (-6.24,4.25) | -2.09 (-7.26,3.36) |
| Low-middle SDI | Mongolia | 80 to 84 | -1.84 (-8.9,5.78) | -1.36 (-8.22,6.01) |
| Low-middle SDI | Mongolia | 85 to 89 | -1.27 (-19.39,20.93) | -0.79 (-18.94,21.42) |
| Low-middle SDI | Morocco | 5 to 9 | -0.37 (-1.73,1.02) | -2.39 (-4.54,-0.2) |
| Low-middle SDI | Morocco | 10 to 14 | 0.39 (-0.74,1.54) | -1.44 (-3.1,0.25) |
| Low-middle SDI | Morocco | 15 to 19 | 0.66 (-0.39,1.73) | -1 (-2.47,0.49) |
| Low-middle SDI | Morocco | 20 to 24 | 0.51 (-0.49,1.52) | -0.99 (-2.35,0.38) |
| Low-middle SDI | Morocco | 25 to 29 | 0.44 (-0.48,1.38) | -1.05 (-2.32,0.23) |
| Low-middle SDI | Morocco | 30 to 34 | 0.53 (-0.33,1.39) | -0.95 (-2.11,0.22) |
| Low-middle SDI | Morocco | 35 to 39 | 0.76 (-0.04,1.56) | -0.62 (-1.68,0.45) |
| Low-middle SDI | Morocco | 40 to 44 | 1.08 (0.34,1.82) | -0.17 (-1.11,0.79) |
| Low-middle SDI | Morocco | 45 to 49 | 1.39 (0.71,2.08) | 0.24 (-0.6,1.09) |
| Low-middle SDI | Morocco | 50 to 54 | 1.53 (0.91,2.16) | 0.52 (-0.23,1.27) |
| Low-middle SDI | Morocco | 55 to 59 | 1.64 (1.06,2.22) | 0.73 (0.07,1.4) |
| Low-middle SDI | Morocco | 60 to 64 | 1.63 (1.09,2.18) | 0.82 (0.22,1.43) |
| Low-middle SDI | Morocco | 65 to 69 | 1.55 (1,2.1) | 0.88 (0.29,1.47) |
| Low-middle SDI | Morocco | 70 to 74 | 1.49 (0.88,2.09) | 0.92 (0.31,1.54) |
| Low-middle SDI | Morocco | 75 to 79 | 1.44 (0.7,2.18) | 0.95 (0.24,1.67) |
| Low-middle SDI | Morocco | 80 to 84 | 1.4 (0.39,2.42) | 1.05 (0.11,2) |
| Low-middle SDI | Morocco | 85 to 89 | 1.63 (-0.16,3.46) | 1.19 (-0.4,2.82) |
| Low-middle SDI | Myanmar | 5 to 9 | -0.8 (-2.04,0.46) | -2.31 (-3.88,-0.72) |
| Low-middle SDI | Myanmar | 10 to 14 | -0.72 (-1.67,0.25) | -1.94 (-3.09,-0.77) |
| Low-middle SDI | Myanmar | 15 to 19 | -0.53 (-1.35,0.29) | -1.57 (-2.51,-0.62) |
| Low-middle SDI | Myanmar | 20 to 24 | -0.44 (-1.2,0.32) | -1.39 (-2.25,-0.53) |
| Low-middle SDI | Myanmar | 25 to 29 | -0.38 (-1.1,0.34) | -1.27 (-2.09,-0.45) |
| Low-middle SDI | Myanmar | 30 to 34 | -0.36 (-1.06,0.33) | -1.23 (-2.02,-0.43) |
| Low-middle SDI | Myanmar | 35 to 39 | -0.48 (-1.15,0.18) | -1.23 (-1.99,-0.47) |
| Low-middle SDI | Myanmar | 40 to 44 | -0.64 (-1.27,-0.01) | -1.29 (-1.99,-0.58) |
| Low-middle SDI | Myanmar | 45 to 49 | -0.78 (-1.37,-0.18) | -1.33 (-1.98,-0.68) |
| Low-middle SDI | Myanmar | 50 to 54 | -0.81 (-1.36,-0.26) | -1.29 (-1.88,-0.7) |
| Low-middle SDI | Myanmar | 55 to 59 | -0.8 (-1.31,-0.28) | -1.22 (-1.76,-0.67) |
| Low-middle SDI | Myanmar | 60 to 64 | -0.69 (-1.18,-0.19) | -1.09 (-1.6,-0.58) |
| Low-middle SDI | Myanmar | 65 to 69 | -0.49 (-1,0.02) | -0.84 (-1.35,-0.33) |
| Low-middle SDI | Myanmar | 70 to 74 | -0.23 (-0.82,0.37) | -0.54 (-1.11,0.04) |
| Low-middle SDI | Myanmar | 75 to 79 | 0 (-0.8,0.81) | -0.22 (-0.97,0.54) |
| Low-middle SDI | Myanmar | 80 to 84 | 0.29 (-0.96,1.56) | 0.06 (-1.06,1.19) |
| Low-middle SDI | Myanmar | 85 to 89 | 0.43 (-1.92,2.84) | 0.29 (-1.79,2.42) |
| Low-middle SDI | Namibia | 5 to 9 | 3.15 (-1.86,8.41) | 2.62 (-3.35,8.96) |
| Low-middle SDI | Namibia | 10 to 14 | 3.25 (-0.91,7.57) | 2.39 (-2.43,7.46) |
| Low-middle SDI | Namibia | 15 to 19 | 2.62 (-1.16,6.54) | 1.84 (-2.53,6.42) |
| Low-middle SDI | Namibia | 20 to 24 | 2.25 (-1.27,5.9) | 0.85 (-3.35,5.24) |
| Low-middle SDI | Namibia | 25 to 29 | 2.02 (-1.21,5.36) | 0.92 (-2.99,4.99) |
| Low-middle SDI | Namibia | 30 to 34 | 1.64 (-1.35,4.72) | 1.04 (-2.57,4.78) |
| Low-middle SDI | Namibia | 35 to 39 | 1.79 (-1.01,4.67) | 0.98 (-2.43,4.51) |
| Low-middle SDI | Namibia | 40 to 44 | 1.8 (-0.86,4.53) | 0.6 (-2.53,3.84) |
| Low-middle SDI | Namibia | 45 to 49 | 1.66 (-0.91,4.29) | 0.81 (-2.09,3.79) |
| Low-middle SDI | Namibia | 50 to 54 | 1.56 (-0.91,4.09) | 0.8 (-1.93,3.61) |
| Low-middle SDI | Namibia | 55 to 59 | 1.4 (-1.03,3.9) | 0.43 (-2.15,3.08) |
| Low-middle SDI | Namibia | 60 to 64 | 1.63 (-0.86,4.18) | 0.4 (-2.17,3.04) |
| Low-middle SDI | Namibia | 65 to 69 | 1.68 (-0.92,4.34) | 1.25 (-1.39,3.96) |
| Low-middle SDI | Namibia | 70 to 74 | 1.29 (-1.54,4.2) | 1.35 (-1.49,4.27) |
| Low-middle SDI | Namibia | 75 to 79 | 0.98 (-2.68,4.78) | 1.21 (-2.17,4.7) |
| Low-middle SDI | Namibia | 80 to 84 | 1 (-5.05,7.43) | 0.95 (-3.87,6) |
| Low-middle SDI | Namibia | 85 to 89 | 4.4 (-13.13,25.47) | 5.24 (-12.3,26.28) |
| Low-middle SDI | Nicaragua | 5 to 9 | -0.19 (-3.51,3.24) | -1.8 (-6.84,3.51) |
| Low-middle SDI | Nicaragua | 10 to 14 | 0.53 (-2.24,3.39) | -1.69 (-5.98,2.8) |
| Low-middle SDI | Nicaragua | 15 to 19 | 1.01 (-1.6,3.69) | -0.87 (-4.53,2.92) |
| Low-middle SDI | Nicaragua | 20 to 24 | 1.53 (-0.93,4.06) | -0.65 (-4.11,2.94) |
| Low-middle SDI | Nicaragua | 25 to 29 | 1.91 (-0.44,4.31) | -0.57 (-3.92,2.89) |
| Low-middle SDI | Nicaragua | 30 to 34 | 2.14 (-0.1,4.43) | -0.37 (-3.56,2.93) |
| Low-middle SDI | Nicaragua | 35 to 39 | 2.1 (-0.08,4.32) | -0.32 (-3.34,2.79) |
| Low-middle SDI | Nicaragua | 40 to 44 | 2.07 (-0.05,4.25) | 0.04 (-2.8,2.96) |
| Low-middle SDI | Nicaragua | 45 to 49 | 2.18 (0.13,4.27) | 0.25 (-2.43,3.01) |
| Low-middle SDI | Nicaragua | 50 to 54 | 2.2 (0.25,4.19) | 0.81 (-1.73,3.42) |
| Low-middle SDI | Nicaragua | 55 to 59 | 2.27 (0.38,4.19) | 0.97 (-1.42,3.43) |
| Low-middle SDI | Nicaragua | 60 to 64 | 2.26 (0.36,4.2) | 1.31 (-1,3.68) |
| Low-middle SDI | Nicaragua | 65 to 69 | 2.21 (0.2,4.27) | 1.13 (-1.21,3.53) |
| Low-middle SDI | Nicaragua | 70 to 74 | 1.89 (-0.32,4.15) | 0.95 (-1.55,3.51) |
| Low-middle SDI | Nicaragua | 75 to 79 | 1.93 (-0.83,4.77) | 0.82 (-2.07,3.8) |
| Low-middle SDI | Nicaragua | 80 to 84 | 0.96 (-2.55,4.61) | 0.23 (-3.35,3.94) |
| Low-middle SDI | Nicaragua | 85 to 89 | 1.1 (-5.1,7.71) | 0.45 (-5.78,7.1) |
| Low-middle SDI | Nigeria | 5 to 9 | 1.02 (0.59,1.46) | 0.06 (-0.43,0.55) |
| Low-middle SDI | Nigeria | 10 to 14 | 1.22 (0.74,1.7) | 0.28 (-0.26,0.83) |
| Low-middle SDI | Nigeria | 15 to 19 | 1.27 (0.72,1.83) | 0.36 (-0.27,1.01) |
| Low-middle SDI | Nigeria | 20 to 24 | 1.26 (0.66,1.86) | 0.38 (-0.32,1.08) |
| Low-middle SDI | Nigeria | 25 to 29 | 1.26 (0.67,1.86) | 0.4 (-0.3,1.12) |
| Low-middle SDI | Nigeria | 30 to 34 | 1.14 (0.57,1.72) | 0.33 (-0.36,1.02) |
| Low-middle SDI | Nigeria | 35 to 39 | 0.86 (0.31,1.42) | 0.12 (-0.53,0.78) |
| Low-middle SDI | Nigeria | 40 to 44 | 0.61 (0.09,1.14) | -0.06 (-0.67,0.55) |
| Low-middle SDI | Nigeria | 45 to 49 | 0.48 (-0.02,0.97) | -0.12 (-0.68,0.45) |
| Low-middle SDI | Nigeria | 50 to 54 | 0.44 (-0.01,0.9) | -0.06 (-0.57,0.44) |
| Low-middle SDI | Nigeria | 55 to 59 | 0.52 (0.09,0.95) | 0.1 (-0.36,0.56) |
| Low-middle SDI | Nigeria | 60 to 64 | 0.59 (0.18,1) | 0.23 (-0.2,0.66) |
| Low-middle SDI | Nigeria | 65 to 69 | 0.61 (0.2,1.02) | 0.31 (-0.11,0.73) |
| Low-middle SDI | Nigeria | 70 to 74 | 0.67 (0.23,1.11) | 0.41 (-0.03,0.84) |
| Low-middle SDI | Nigeria | 75 to 79 | 0.8 (0.29,1.31) | 0.58 (0.1,1.06) |
| Low-middle SDI | Nigeria | 80 to 84 | 0.95 (0.23,1.67) | 0.74 (0.09,1.4) |
| Low-middle SDI | Nigeria | 85 to 89 | 1.11 (-0.24,2.47) | 1.02 (-0.19,2.24) |
| Low-middle SDI | Pakistan | 5 to 9 | 1.65 (1.31,2) | 0.68 (0.26,1.11) |
| Low-middle SDI | Pakistan | 10 to 14 | 1.62 (1.31,1.93) | 0.72 (0.35,1.1) |
| Low-middle SDI | Pakistan | 15 to 19 | 1.41 (1.1,1.72) | 0.59 (0.23,0.96) |
| Low-middle SDI | Pakistan | 20 to 24 | 1.3 (0.99,1.61) | 0.54 (0.17,0.9) |
| Low-middle SDI | Pakistan | 25 to 29 | 1.21 (0.91,1.52) | 0.48 (0.12,0.84) |
| Low-middle SDI | Pakistan | 30 to 34 | 1.07 (0.77,1.37) | 0.37 (0.02,0.73) |
| Low-middle SDI | Pakistan | 35 to 39 | 0.84 (0.55,1.13) | 0.22 (-0.12,0.57) |
| Low-middle SDI | Pakistan | 40 to 44 | 0.66 (0.37,0.94) | 0.11 (-0.22,0.44) |
| Low-middle SDI | Pakistan | 45 to 49 | 0.53 (0.26,0.81) | 0.06 (-0.25,0.37) |
| Low-middle SDI | Pakistan | 50 to 54 | 0.48 (0.22,0.75) | 0.08 (-0.21,0.36) |
| Low-middle SDI | Pakistan | 55 to 59 | 0.46 (0.21,0.71) | 0.11 (-0.16,0.38) |
| Low-middle SDI | Pakistan | 60 to 64 | 0.47 (0.23,0.72) | 0.17 (-0.09,0.42) |
| Low-middle SDI | Pakistan | 65 to 69 | 0.51 (0.26,0.76) | 0.27 (0.02,0.52) |
| Low-middle SDI | Pakistan | 70 to 74 | 0.58 (0.31,0.85) | 0.38 (0.11,0.65) |
| Low-middle SDI | Pakistan | 75 to 79 | 0.7 (0.37,1.03) | 0.54 (0.23,0.85) |
| Low-middle SDI | Pakistan | 80 to 84 | 0.77 (0.33,1.21) | 0.64 (0.24,1.04) |
| Low-middle SDI | Pakistan | 85 to 89 | 0.75 (0,1.52) | 0.61 (-0.06,1.29) |
| Low-middle SDI | Palestine | 5 to 9 | 1.64 (-1.65,5.04) | -0.02 (-5.51,5.8) |
| Low-middle SDI | Palestine | 10 to 14 | 2.59 (-0.17,5.43) | 0.59 (-3.78,5.17) |
| Low-middle SDI | Palestine | 15 to 19 | 1.77 (-0.84,4.45) | -0.14 (-4.15,4.03) |
| Low-middle SDI | Palestine | 20 to 24 | 2.1 (-0.41,4.68) | 0.06 (-3.8,4.07) |
| Low-middle SDI | Palestine | 25 to 29 | 1.89 (-0.55,4.39) | 0.35 (-3.45,4.31) |
| Low-middle SDI | Palestine | 30 to 34 | 1.46 (-0.97,3.95) | 0.34 (-3.4,4.22) |
| Low-middle SDI | Palestine | 35 to 39 | 0.96 (-1.49,3.47) | -0.15 (-3.74,3.57) |
| Low-middle SDI | Palestine | 40 to 44 | 0.76 (-1.65,3.23) | -0.59 (-3.94,2.89) |
| Low-middle SDI | Palestine | 45 to 49 | 0.24 (-2.07,2.6) | -1.13 (-4.21,2.04) |
| Low-middle SDI | Palestine | 50 to 54 | 0.35 (-1.86,2.61) | -0.96 (-3.77,1.92) |
| Low-middle SDI | Palestine | 55 to 59 | 0.34 (-1.78,2.51) | -0.89 (-3.46,1.74) |
| Low-middle SDI | Palestine | 60 to 64 | 0.42 (-1.62,2.5) | -0.55 (-2.93,1.88) |
| Low-middle SDI | Palestine | 65 to 69 | 0.37 (-1.71,2.5) | -0.62 (-2.96,1.78) |
| Low-middle SDI | Palestine | 70 to 74 | 0.46 (-1.89,2.87) | -0.57 (-3.03,1.95) |
| Low-middle SDI | Palestine | 75 to 79 | 0.45 (-2.53,3.51) | -0.17 (-3.18,2.95) |
| Low-middle SDI | Palestine | 80 to 84 | 0.13 (-3.87,4.29) | 0.02 (-3.98,4.19) |
| Low-middle SDI | Palestine | 85 to 89 | -0.27 (-6.7,6.61) | 0.11 (-6.31,6.98) |
| Low-middle SDI | Samoa | 5 to 9 | 0.64 (-18.09,23.65) | 0.1 (-21.19,27.16) |
| Low-middle SDI | Samoa | 10 to 14 | 2.2 (-7.4,12.8) | -0.29 (-17.53,20.55) |
| Low-middle SDI | Samoa | 15 to 19 | 3.87 (-5.87,14.61) | -0.42 (-16.31,18.48) |
| Low-middle SDI | Samoa | 20 to 24 | 4.66 (-4.59,14.8) | -0.32 (-15.75,17.94) |
| Low-middle SDI | Samoa | 25 to 29 | 5.01 (-4.5,15.48) | -0.07 (-15.35,17.97) |
| Low-middle SDI | Samoa | 30 to 34 | 6.28 (-4.01,17.67) | -1 (-15.77,16.36) |
| Low-middle SDI | Samoa | 35 to 39 | 6.96 (-4.46,19.75) | -1.26 (-15.86,15.89) |
| Low-middle SDI | Samoa | 40 to 44 | 6.79 (-5.03,20.08) | -0.27 (-14.64,16.51) |
| Low-middle SDI | Samoa | 45 to 49 | 6.33 (-4.51,18.41) | 0.39 (-13.16,16.05) |
| Low-middle SDI | Samoa | 50 to 54 | 4.47 (-5.21,15.14) | 1.2 (-10.81,14.83) |
| Low-middle SDI | Samoa | 55 to 59 | 1.73 (-6.5,10.68) | 2.24 (-7.63,13.17) |
| Low-middle SDI | Samoa | 60 to 64 | -0.12 (-6.69,6.92) | 1.36 (-6.63,10.02) |
| Low-middle SDI | Samoa | 65 to 69 | -0.41 (-6.11,5.63) | 0.16 (-6.44,7.23) |
| Low-middle SDI | Samoa | 70 to 74 | -0.69 (-6.47,5.45) | -0.28 (-6.11,5.92) |
| Low-middle SDI | Samoa | 75 to 79 | 3.46 (-7.9,16.23) | 3.53 (-7.83,16.3) |
| Low-middle SDI | Samoa | 80 to 84 | 4.47 (-10.73,22.26) | 4.2 (-11.01,22) |
| Low-middle SDI | Samoa | 85 to 89 | 1.61 (-19.24,27.84) | 1.6 (-19.32,27.95) |
| Low-middle SDI | Sao Tome and Principe | 5 to 9 | -1.22 (-22.45,25.82) | - |
| Low-middle SDI | Sao Tome and Principe | 10 to 14 | -1.38 (-18.48,19.31) | - |
| Low-middle SDI | Sao Tome and Principe | 15 to 19 | -1.55 (-17.27,17.15) | - |
| Low-middle SDI | Sao Tome and Principe | 20 to 24 | -1.96 (-17.14,16.01) | - |
| Low-middle SDI | Sao Tome and Principe | 25 to 29 | -2.63 (-17.53,14.96) | - |
| Low-middle SDI | Sao Tome and Principe | 30 to 34 | -3.2 (-17.9,14.13) | - |
| Low-middle SDI | Sao Tome and Principe | 35 to 39 | -3.56 (-18.2,13.7) | - |
| Low-middle SDI | Sao Tome and Principe | 40 to 44 | -3.79 (-18.39,13.43) | - |
| Low-middle SDI | Sao Tome and Principe | 45 to 49 | -4 (-18.57,13.18) | - |
| Low-middle SDI | Sao Tome and Principe | 50 to 54 | -3.62 (-18.24,13.63) | - |
| Low-middle SDI | Sao Tome and Principe | 55 to 59 | -2.58 (-17.36,14.86) | - |
| Low-middle SDI | Sao Tome and Principe | 60 to 64 | -1.53 (-16.49,16.1) | - |
| Low-middle SDI | Sao Tome and Principe | 65 to 69 | -0.67 (-15.87,17.27) | - |
| Low-middle SDI | Sao Tome and Principe | 70 to 74 | -0.57 (-15.97,17.66) | - |
| Low-middle SDI | Sao Tome and Principe | 75 to 79 | -1.21 (-16.98,17.55) | - |
| Low-middle SDI | Sao Tome and Principe | 80 to 84 | -1.69 (-18.74,18.93) | - |
| Low-middle SDI | Sao Tome and Principe | 85 to 89 | -1.4 (-22.59,25.59) | - |
| Low-middle SDI | Sudan | 5 to 9 | 0.71 (-0.34,1.78) | -0.98 (-2.45,0.51) |
| Low-middle SDI | Sudan | 10 to 14 | 1.34 (0.35,2.34) | -0.19 (-1.5,1.14) |
| Low-middle SDI | Sudan | 15 to 19 | 1.6 (0.59,2.63) | 0.2 (-1.1,1.52) |
| Low-middle SDI | Sudan | 20 to 24 | 1.71 (0.68,2.75) | 0.38 (-0.92,1.7) |
| Low-middle SDI | Sudan | 25 to 29 | 1.65 (0.64,2.67) | 0.33 (-0.96,1.64) |
| Low-middle SDI | Sudan | 30 to 34 | 1.46 (0.47,2.46) | 0.16 (-1.1,1.43) |
| Low-middle SDI | Sudan | 35 to 39 | 1.18 (0.21,2.16) | -0.08 (-1.29,1.15) |
| Low-middle SDI | Sudan | 40 to 44 | 0.99 (0.03,1.97) | -0.16 (-1.33,1.02) |
| Low-middle SDI | Sudan | 45 to 49 | 0.78 (-0.17,1.73) | -0.26 (-1.37,0.86) |
| Low-middle SDI | Sudan | 50 to 54 | 0.64 (-0.29,1.59) | -0.27 (-1.34,0.81) |
| Low-middle SDI | Sudan | 55 to 59 | 0.59 (-0.33,1.52) | -0.2 (-1.23,0.83) |
| Low-middle SDI | Sudan | 60 to 64 | 0.59 (-0.33,1.52) | -0.08 (-1.07,0.93) |
| Low-middle SDI | Sudan | 65 to 69 | 0.59 (-0.36,1.55) | 0.04 (-0.96,1.04) |
| Low-middle SDI | Sudan | 70 to 74 | 0.64 (-0.39,1.68) | 0.19 (-0.84,1.23) |
| Low-middle SDI | Sudan | 75 to 79 | 0.73 (-0.52,2) | 0.36 (-0.84,1.58) |
| Low-middle SDI | Sudan | 80 to 84 | 0.96 (-0.95,2.89) | 0.65 (-1.09,2.43) |
| Low-middle SDI | Sudan | 85 to 89 | 1.12 (-2.5,4.88) | 0.62 (-2.56,3.9) |
| Low-middle SDI | Syrian Arab Republic | 5 to 9 | 0.25 (-1.48,2) | -2.8 (-6.34,0.87) |
| Low-middle SDI | Syrian Arab Republic | 10 to 14 | 1.05 (-0.17,2.28) | -1.65 (-3.83,0.59) |
| Low-middle SDI | Syrian Arab Republic | 15 to 19 | 1.27 (0.09,2.46) | -1.27 (-3.21,0.71) |
| Low-middle SDI | Syrian Arab Republic | 20 to 24 | 1.28 (0.06,2.52) | -1.02 (-2.96,0.96) |
| Low-middle SDI | Syrian Arab Republic | 25 to 29 | 1.13 (-0.07,2.35) | -1.18 (-3.08,0.76) |
| Low-middle SDI | Syrian Arab Republic | 30 to 34 | 0.83 (-0.29,1.97) | -1.49 (-3.25,0.3) |
| Low-middle SDI | Syrian Arab Republic | 35 to 39 | 0.4 (-0.67,1.48) | -1.91 (-3.53,-0.26) |
| Low-middle SDI | Syrian Arab Republic | 40 to 44 | 0.12 (-0.91,1.16) | -2.03 (-3.53,-0.51) |
| Low-middle SDI | Syrian Arab Republic | 45 to 49 | -0.14 (-1.13,0.86) | -2.09 (-3.46,-0.69) |
| Low-middle SDI | Syrian Arab Republic | 50 to 54 | -0.22 (-1.19,0.77) | -1.97 (-3.26,-0.66) |
| Low-middle SDI | Syrian Arab Republic | 55 to 59 | -0.27 (-1.23,0.7) | -1.89 (-3.1,-0.66) |
| Low-middle SDI | Syrian Arab Republic | 60 to 64 | -0.17 (-1.14,0.8) | -1.66 (-2.82,-0.49) |
| Low-middle SDI | Syrian Arab Republic | 65 to 69 | -0.15 (-1.2,0.91) | -1.47 (-2.66,-0.26) |
| Low-middle SDI | Syrian Arab Republic | 70 to 74 | -0.07 (-1.31,1.19) | -1.18 (-2.52,0.18) |
| Low-middle SDI | Syrian Arab Republic | 75 to 79 | 0.22 (-1.44,1.9) | -1 (-2.62,0.65) |
| Low-middle SDI | Syrian Arab Republic | 80 to 84 | 0.31 (-1.98,2.65) | -0.48 (-2.67,1.76) |
| Low-middle SDI | Syrian Arab Republic | 85 to 89 | 0.6 (-3.16,4.5) | -0.37 (-3.72,3.1) |
| Low-middle SDI | Tajikistan | 5 to 9 | -1.36 (-3.04,0.34) | -2.16 (-4.64,0.39) |
| Low-middle SDI | Tajikistan | 10 to 14 | -1.56 (-3.1,0) | -2.15 (-4.3,0.04) |
| Low-middle SDI | Tajikistan | 15 to 19 | -1.54 (-3.06,0) | -2.2 (-4.21,-0.16) |
| Low-middle SDI | Tajikistan | 20 to 24 | -1.33 (-2.78,0.14) | -2.06 (-3.95,-0.14) |
| Low-middle SDI | Tajikistan | 25 to 29 | -1.27 (-2.66,0.13) | -2 (-3.82,-0.14) |
| Low-middle SDI | Tajikistan | 30 to 34 | -1.48 (-2.87,-0.07) | -2.09 (-3.9,-0.25) |
| Low-middle SDI | Tajikistan | 35 to 39 | -1.56 (-3.04,-0.06) | -2 (-3.9,-0.06) |
| Low-middle SDI | Tajikistan | 40 to 44 | -1.69 (-3.27,-0.08) | -2.01 (-3.98,0.01) |
| Low-middle SDI | Tajikistan | 45 to 49 | -1.67 (-3.35,0.04) | -2.21 (-4.24,-0.14) |
| Low-middle SDI | Tajikistan | 50 to 54 | -1.88 (-3.55,-0.19) | -2.27 (-4.24,-0.26) |
| Low-middle SDI | Tajikistan | 55 to 59 | -1.68 (-3.31,-0.03) | -2.05 (-3.93,-0.14) |
| Low-middle SDI | Tajikistan | 60 to 64 | -1.32 (-2.98,0.37) | -1.73 (-3.61,0.18) |
| Low-middle SDI | Tajikistan | 65 to 69 | -0.67 (-2.52,1.21) | -0.85 (-2.88,1.23) |
| Low-middle SDI | Tajikistan | 70 to 74 | -0.02 (-2.33,2.35) | -0.16 (-2.56,2.3) |
| Low-middle SDI | Tajikistan | 75 to 79 | 0.5 (-2.5,3.6) | 0.26 (-2.78,3.39) |
| Low-middle SDI | Tajikistan | 80 to 84 | 0.51 (-3.62,4.8) | -0.5 (-4.27,3.41) |
| Low-middle SDI | Tajikistan | 85 to 89 | 0.51 (-6.23,7.73) | -0.38 (-6.96,6.66) |
| Low-middle SDI | Tonga | 5 to 9 | 0.21 (-18.51,23.23) | 0.4 (-21.14,27.81) |
| Low-middle SDI | Tonga | 10 to 14 | 0.9 (-7.43,9.99) | 0.38 (-17.01,21.43) |
| Low-middle SDI | Tonga | 15 to 19 | 2.06 (-7.5,12.59) | 0.27 (-15.74,19.31) |
| Low-middle SDI | Tonga | 20 to 24 | 2.84 (-6.95,13.65) | 0.24 (-15.29,18.61) |
| Low-middle SDI | Tonga | 25 to 29 | 5.66 (-5.87,18.6) | 0.14 (-15.18,18.24) |
| Low-middle SDI | Tonga | 30 to 34 | 4.97 (-8.29,20.15) | -0.35 (-15.48,17.5) |
| Low-middle SDI | Tonga | 35 to 39 | 3.35 (-11.05,20.08) | -0.99 (-16.01,16.73) |
| Low-middle SDI | Tonga | 40 to 44 | 1.23 (-13.6,18.61) | -1.44 (-16.39,16.19) |
| Low-middle SDI | Tonga | 45 to 49 | -0.67 (-15.14,16.27) | -1.55 (-16.48,16.04) |
| Low-middle SDI | Tonga | 50 to 54 | -2.37 (-16.76,14.51) | -1.45 (-16.16,15.84) |
| Low-middle SDI | Tonga | 55 to 59 | -1.17 (-15.33,15.35) | -0.94 (-15.71,16.41) |
| Low-middle SDI | Tonga | 60 to 64 | 0.16 (-13.03,15.35) | -0.4 (-14.83,16.47) |
| Low-middle SDI | Tonga | 65 to 69 | 0.86 (-11.11,14.46) | -0.26 (-13.64,15.2) |
| Low-middle SDI | Tonga | 70 to 74 | 4.37 (-7.99,18.38) | 1.61 (-12.17,17.55) |
| Low-middle SDI | Tonga | 75 to 79 | 5.16 (-8.99,21.51) | 3.16 (-11.04,19.62) |
| Low-middle SDI | Tonga | 80 to 84 | 2.49 (-14.34,22.61) | 1.17 (-15.6,21.28) |
| Low-middle SDI | Tonga | 85 to 89 | -0.42 (-21.54,26.38) | -0.96 (-22.12,25.94) |
| Low-middle SDI | Venezuela (Bolivarian Republic of) | 5 to 9 | 1.42 (0.12,2.74) | -0.28 (-2.44,1.93) |
| Low-middle SDI | Venezuela (Bolivarian Republic of) | 10 to 14 | 1.82 (0.8,2.85) | 0.21 (-1.42,1.87) |
| Low-middle SDI | Venezuela (Bolivarian Republic of) | 15 to 19 | 2.12 (1.23,3.01) | 0.69 (-0.66,2.05) |
| Low-middle SDI | Venezuela (Bolivarian Republic of) | 20 to 24 | 2.34 (1.54,3.15) | 0.93 (-0.28,2.16) |
| Low-middle SDI | Venezuela (Bolivarian Republic of) | 25 to 29 | 2.32 (1.6,3.06) | 0.89 (-0.24,2.02) |
| Low-middle SDI | Venezuela (Bolivarian Republic of) | 30 to 34 | 1.84 (1.16,2.53) | 0.42 (-0.64,1.48) |
| Low-middle SDI | Venezuela (Bolivarian Republic of) | 35 to 39 | 1.14 (0.49,1.79) | -0.16 (-1.14,0.84) |
| Low-middle SDI | Venezuela (Bolivarian Republic of) | 40 to 44 | 0.57 (-0.05,1.19) | -0.62 (-1.53,0.3) |
| Low-middle SDI | Venezuela (Bolivarian Republic of) | 45 to 49 | 0.23 (-0.35,0.82) | -0.83 (-1.66,0.02) |
| Low-middle SDI | Venezuela (Bolivarian Republic of) | 50 to 54 | 0.14 (-0.41,0.69) | -0.86 (-1.62,-0.09) |
| Low-middle SDI | Venezuela (Bolivarian Republic of) | 55 to 59 | 0.25 (-0.27,0.78) | -0.71 (-1.41,0) |
| Low-middle SDI | Venezuela (Bolivarian Republic of) | 60 to 64 | 0.29 (-0.22,0.81) | -0.65 (-1.32,0.03) |
| Low-middle SDI | Venezuela (Bolivarian Republic of) | 65 to 69 | 0.28 (-0.26,0.82) | -0.59 (-1.26,0.08) |
| Low-middle SDI | Venezuela (Bolivarian Republic of) | 70 to 74 | 0.39 (-0.22,1) | -0.36 (-1.07,0.36) |
| Low-middle SDI | Venezuela (Bolivarian Republic of) | 75 to 79 | 0.67 (-0.1,1.44) | 0.07 (-0.78,0.92) |
| Low-middle SDI | Venezuela (Bolivarian Republic of) | 80 to 84 | 1.02 (-0.06,2.12) | 0.53 (-0.6,1.67) |
| Low-middle SDI | Venezuela (Bolivarian Republic of) | 85 to 89 | 2.04 (0.11,4) | 1.49 (-0.45,3.47) |
| Low-middle SDI | Viet Nam | 5 to 9 | 2.32 (1.43,3.21) | -0.32 (-1.74,1.11) |
| Low-middle SDI | Viet Nam | 10 to 14 | 2.75 (2.02,3.49) | 0.37 (-0.72,1.47) |
| Low-middle SDI | Viet Nam | 15 to 19 | 2.9 (2.23,3.58) | 0.71 (-0.22,1.64) |
| Low-middle SDI | Viet Nam | 20 to 24 | 2.7 (2.09,3.32) | 0.61 (-0.22,1.44) |
| Low-middle SDI | Viet Nam | 25 to 29 | 2.44 (1.89,3) | 0.43 (-0.32,1.17) |
| Low-middle SDI | Viet Nam | 30 to 34 | 2.21 (1.71,2.72) | 0.34 (-0.33,1.02) |
| Low-middle SDI | Viet Nam | 35 to 39 | 2.07 (1.6,2.53) | 0.38 (-0.23,0.99) |
| Low-middle SDI | Viet Nam | 40 to 44 | 2.04 (1.61,2.48) | 0.55 (0.01,1.1) |
| Low-middle SDI | Viet Nam | 45 to 49 | 2.01 (1.61,2.42) | 0.68 (0.19,1.17) |
| Low-middle SDI | Viet Nam | 50 to 54 | 1.89 (1.51,2.27) | 0.7 (0.26,1.13) |
| Low-middle SDI | Viet Nam | 55 to 59 | 1.67 (1.33,2.02) | 0.58 (0.2,0.97) |
| Low-middle SDI | Viet Nam | 60 to 64 | 1.34 (1.02,1.66) | 0.34 (-0.01,0.69) |
| Low-middle SDI | Viet Nam | 65 to 69 | 0.91 (0.58,1.24) | 0.04 (-0.31,0.38) |
| Low-middle SDI | Viet Nam | 70 to 74 | 0.46 (0.1,0.82) | -0.29 (-0.66,0.07) |
| Low-middle SDI | Viet Nam | 75 to 79 | 0.01 (-0.43,0.45) | -0.61 (-1.04,-0.19) |
| Low-middle SDI | Viet Nam | 80 to 84 | -0.2 (-0.8,0.4) | -0.71 (-1.27,-0.15) |
| Low-middle SDI | Viet Nam | 85 to 89 | -0.01 (-1.04,1.03) | -0.48 (-1.41,0.45) |
| Low-middle SDI | Zambia | 5 to 9 | -0.57 (-1.88,0.75) | -1.14 (-2.56,0.3) |
| Low-middle SDI | Zambia | 10 to 14 | 0.29 (-0.84,1.43) | -0.38 (-1.63,0.88) |
| Low-middle SDI | Zambia | 15 to 19 | 1.03 (-0.06,2.13) | 0.32 (-0.9,1.54) |
| Low-middle SDI | Zambia | 20 to 24 | 1.27 (0.2,2.35) | 0.56 (-0.66,1.79) |
| Low-middle SDI | Zambia | 25 to 29 | 1.36 (0.3,2.44) | 0.7 (-0.52,1.95) |
| Low-middle SDI | Zambia | 30 to 34 | 1.4 (0.33,2.47) | 0.76 (-0.47,2.01) |
| Low-middle SDI | Zambia | 35 to 39 | 1.31 (0.26,2.38) | 0.7 (-0.51,1.93) |
| Low-middle SDI | Zambia | 40 to 44 | 1.12 (0.08,2.17) | 0.6 (-0.58,1.78) |
| Low-middle SDI | Zambia | 45 to 49 | 0.95 (-0.06,1.98) | 0.48 (-0.65,1.61) |
| Low-middle SDI | Zambia | 50 to 54 | 0.72 (-0.27,1.72) | 0.34 (-0.74,1.42) |
| Low-middle SDI | Zambia | 55 to 59 | 0.45 (-0.52,1.42) | 0.17 (-0.85,1.2) |
| Low-middle SDI | Zambia | 60 to 64 | 0.26 (-0.69,1.22) | -0.01 (-1,0.98) |
| Low-middle SDI | Zambia | 65 to 69 | 0.13 (-0.86,1.14) | -0.13 (-1.13,0.87) |
| Low-middle SDI | Zambia | 70 to 74 | 0.11 (-1.02,1.26) | -0.13 (-1.23,0.98) |
| Low-middle SDI | Zambia | 75 to 79 | 0.16 (-1.31,1.65) | 0.03 (-1.35,1.43) |
| Low-middle SDI | Zambia | 80 to 84 | 0.19 (-1.88,2.29) | 0.08 (-1.78,1.97) |
| Low-middle SDI | Zambia | 85 to 89 | 0.17 (-3.52,4.01) | 0.9 (-2.72,4.66) |
| Middle SDI | Albania | 5 to 9 | -0.53 (-5.19,4.35) | -6.18 (-21.98,12.82) |
| Middle SDI | Albania | 10 to 14 | 1.09 (-2.61,4.93) | -0.18 (-7.25,7.44) |
| Middle SDI | Albania | 15 to 19 | 1.79 (-1.7,5.41) | 0.28 (-5.82,6.78) |
| Middle SDI | Albania | 20 to 24 | 1.9 (-1.45,5.35) | 0.77 (-4.75,6.62) |
| Middle SDI | Albania | 25 to 29 | 1.73 (-1.53,5.1) | 1.01 (-4.13,6.43) |
| Middle SDI | Albania | 30 to 34 | 2.29 (-0.88,5.57) | 1.46 (-3.61,6.8) |
| Middle SDI | Albania | 35 to 39 | 3.36 (0.17,6.65) | 1.55 (-3.46,6.83) |
| Middle SDI | Albania | 40 to 44 | 3.9 (0.72,7.18) | 1.22 (-3.6,6.28) |
| Middle SDI | Albania | 45 to 49 | 3.64 (0.61,6.75) | 0.63 (-3.86,5.33) |
| Middle SDI | Albania | 50 to 54 | 2.9 (0.16,5.71) | -0.07 (-4.03,4.06) |
| Middle SDI | Albania | 55 to 59 | 2.26 (-0.19,4.76) | -0.44 (-3.86,3.11) |
| Middle SDI | Albania | 60 to 64 | 1.6 (-0.66,3.9) | -0.67 (-3.71,2.46) |
| Middle SDI | Albania | 65 to 69 | 1.35 (-0.89,3.63) | -0.63 (-3.47,2.3) |
| Middle SDI | Albania | 70 to 74 | 1.28 (-1.08,3.69) | -0.51 (-3.31,2.37) |
| Middle SDI | Albania | 75 to 79 | 1.41 (-1.39,4.28) | 0.84 (-2.42,4.22) |
| Middle SDI | Albania | 80 to 84 | 2.28 (-2.26,7.02) | 0.6 (-3.42,4.78) |
| Middle SDI | Albania | 85 to 89 | 1.47 (-5.01,8.39) | 0.09 (-6.29,6.89) |
| Middle SDI | Algeria | 5 to 9 | -0.06 (-0.98,0.86) | -2.67 (-4.33,-0.99) |
| Middle SDI | Algeria | 10 to 14 | 0.77 (-0.05,1.59) | -1.82 (-3.2,-0.41) |
| Middle SDI | Algeria | 15 to 19 | 1.22 (0.4,2.04) | -1.29 (-2.6,0.04) |
| Middle SDI | Algeria | 20 to 24 | 1.44 (0.65,2.23) | -0.91 (-2.13,0.33) |
| Middle SDI | Algeria | 25 to 29 | 1.49 (0.74,2.24) | -0.75 (-1.9,0.41) |
| Middle SDI | Algeria | 30 to 34 | 1.49 (0.76,2.22) | -0.68 (-1.76,0.42) |
| Middle SDI | Algeria | 35 to 39 | 1.48 (0.77,2.21) | -0.57 (-1.61,0.48) |
| Middle SDI | Algeria | 40 to 44 | 1.53 (0.81,2.25) | -0.38 (-1.38,0.63) |
| Middle SDI | Algeria | 45 to 49 | 1.57 (0.86,2.29) | -0.19 (-1.14,0.77) |
| Middle SDI | Algeria | 50 to 54 | 1.46 (0.77,2.15) | -0.14 (-1.02,0.75) |
| Middle SDI | Algeria | 55 to 59 | 1.39 (0.74,2.05) | -0.04 (-0.85,0.77) |
| Middle SDI | Algeria | 60 to 64 | 1.25 (0.63,1.87) | -0.06 (-0.8,0.68) |
| Middle SDI | Algeria | 65 to 69 | 1.07 (0.44,1.71) | -0.08 (-0.8,0.64) |
| Middle SDI | Algeria | 70 to 74 | 0.84 (0.16,1.53) | -0.15 (-0.88,0.58) |
| Middle SDI | Algeria | 75 to 79 | 0.68 (-0.08,1.44) | -0.14 (-0.9,0.62) |
| Middle SDI | Algeria | 80 to 84 | 0.5 (-0.47,1.47) | -0.13 (-1.06,0.8) |
| Middle SDI | Algeria | 85 to 89 | 1.66 (-0.36,3.72) | 1.04 (-0.79,2.91) |
| Middle SDI | Armenia | 5 to 9 | 3.42 (-0.69,7.7) | 2.45 (-5,10.5) |
| Middle SDI | Armenia | 10 to 14 | 3.4 (-0.14,7.07) | 2.18 (-3.72,8.43) |
| Middle SDI | Armenia | 15 to 19 | 2.98 (-0.37,6.43) | 1.32 (-3.86,6.78) |
| Middle SDI | Armenia | 20 to 24 | 2.17 (-0.88,5.3) | 0.26 (-4.65,5.42) |
| Middle SDI | Armenia | 25 to 29 | 1.5 (-1.29,4.37) | -0.47 (-5.14,4.43) |
| Middle SDI | Armenia | 30 to 34 | 1.44 (-1.1,4.04) | -0.26 (-4.51,4.17) |
| Middle SDI | Armenia | 35 to 39 | 1.86 (-0.54,4.32) | 1.05 (-2.72,4.97) |
| Middle SDI | Armenia | 40 to 44 | 2.66 (0.36,5.02) | 1.71 (-1.79,5.34) |
| Middle SDI | Armenia | 45 to 49 | 3.6 (1.39,5.85) | 1.81 (-1.38,5.1) |
| Middle SDI | Armenia | 50 to 54 | 3.5 (1.45,5.59) | 1.72 (-1.13,4.65) |
| Middle SDI | Armenia | 55 to 59 | 3.41 (1.59,5.27) | 1.92 (-0.52,4.41) |
| Middle SDI | Armenia | 60 to 64 | 3.18 (1.44,4.94) | 1.92 (-0.32,4.2) |
| Middle SDI | Armenia | 65 to 69 | 3.12 (1.29,4.98) | 2.1 (-0.13,4.38) |
| Middle SDI | Armenia | 70 to 74 | 3.52 (0.84,6.26) | 2.32 (-0.51,5.24) |
| Middle SDI | Armenia | 75 to 79 | 3.82 (0.04,7.74) | 2.62 (-1.2,6.6) |
| Middle SDI | Armenia | 80 to 84 | 4.6 (-1.45,11.01) | 3.65 (-2.4,10.07) |
| Middle SDI | Armenia | 85 to 89 | 8.75 (-9.38,30.5) | 8.36 (-9.72,30.05) |
| Middle SDI | Azerbaijan | 5 to 9 | 0.22 (-1.97,2.46) | -1.79 (-5.36,1.92) |
| Middle SDI | Azerbaijan | 10 to 14 | 0.51 (-1.48,2.54) | -1.48 (-4.62,1.76) |
| Middle SDI | Azerbaijan | 15 to 19 | 0.07 (-1.85,2.02) | -1.76 (-4.58,1.15) |
| Middle SDI | Azerbaijan | 20 to 24 | -0.48 (-2.31,1.38) | -2.18 (-4.76,0.46) |
| Middle SDI | Azerbaijan | 25 to 29 | -0.81 (-2.55,0.95) | -2.44 (-4.86,0.04) |
| Middle SDI | Azerbaijan | 30 to 34 | -1.1 (-2.78,0.61) | -2.59 (-4.95,-0.17) |
| Middle SDI | Azerbaijan | 35 to 39 | -0.47 (-2.17,1.25) | -1.94 (-4.27,0.44) |
| Middle SDI | Azerbaijan | 40 to 44 | 0.05 (-1.65,1.77) | -1.25 (-3.5,1.06) |
| Middle SDI | Azerbaijan | 45 to 49 | 0.02 (-1.69,1.75) | -1.22 (-3.4,1.01) |
| Middle SDI | Azerbaijan | 50 to 54 | -0.09 (-1.7,1.55) | -1.34 (-3.34,0.7) |
| Middle SDI | Azerbaijan | 55 to 59 | 0.07 (-1.41,1.57) | -1.18 (-2.96,0.64) |
| Middle SDI | Azerbaijan | 60 to 64 | 0.18 (-1.32,1.7) | -0.82 (-2.57,0.97) |
| Middle SDI | Azerbaijan | 65 to 69 | 0.59 (-1.14,2.35) | -0.38 (-2.31,1.59) |
| Middle SDI | Azerbaijan | 70 to 74 | 1.11 (-1.23,3.49) | 0.45 (-2.07,3.04) |
| Middle SDI | Azerbaijan | 75 to 79 | 2.97 (-0.74,6.8) | 1.86 (-1.63,5.47) |
| Middle SDI | Azerbaijan | 80 to 84 | 2.26 (-2.01,6.7) | 1.78 (-2.46,6.2) |
| Middle SDI | Azerbaijan | 85 to 89 | 1.21 (-5.66,8.58) | 1.47 (-5.37,8.8) |
| Middle SDI | Bosnia and Herzegovina | 5 to 9 | 1.86 (-3.02,7) | -7.36 (-23.97,12.88) |
| Middle SDI | Bosnia and Herzegovina | 10 to 14 | 2.84 (-0.73,6.54) | -4.31 (-16.2,9.28) |
| Middle SDI | Bosnia and Herzegovina | 15 to 19 | 3.33 (0.3,6.46) | 1.13 (-4.12,6.67) |
| Middle SDI | Bosnia and Herzegovina | 20 to 24 | 2.75 (0.02,5.56) | -0.22 (-5.01,4.8) |
| Middle SDI | Bosnia and Herzegovina | 25 to 29 | 2.39 (-0.11,4.95) | -0.66 (-5.04,3.92) |
| Middle SDI | Bosnia and Herzegovina | 30 to 34 | 2.29 (0.05,4.58) | -0.57 (-4.47,3.48) |
| Middle SDI | Bosnia and Herzegovina | 35 to 39 | 2.21 (0.19,4.27) | -0.22 (-3.63,3.31) |
| Middle SDI | Bosnia and Herzegovina | 40 to 44 | 2.59 (0.77,4.45) | 0.2 (-2.82,3.31) |
| Middle SDI | Bosnia and Herzegovina | 45 to 49 | 3.11 (1.49,4.76) | 0.89 (-1.7,3.55) |
| Middle SDI | Bosnia and Herzegovina | 50 to 54 | 3.14 (1.74,4.57) | 1.02 (-1.1,3.18) |
| Middle SDI | Bosnia and Herzegovina | 55 to 59 | 3.1 (1.88,4.33) | 1.07 (-0.67,2.85) |
| Middle SDI | Bosnia and Herzegovina | 60 to 64 | 3.07 (1.96,4.2) | 1.14 (-0.37,2.68) |
| Middle SDI | Bosnia and Herzegovina | 65 to 69 | 3.05 (1.91,4.19) | 1.32 (-0.13,2.78) |
| Middle SDI | Bosnia and Herzegovina | 70 to 74 | 3.35 (1.98,4.74) | 1.87 (0.27,3.51) |
| Middle SDI | Bosnia and Herzegovina | 75 to 79 | 3.55 (1.6,5.54) | 2.61 (0.44,4.82) |
| Middle SDI | Bosnia and Herzegovina | 80 to 84 | 3.25 (0.67,5.9) | 2.34 (-0.32,5.08) |
| Middle SDI | Bosnia and Herzegovina | 85 to 89 | 3.21 (-1.29,7.91) | 2.34 (-2.21,7.1) |
| Middle SDI | Botswana | 5 to 9 | 1.09 (-5.94,8.64) | -0.13 (-7.38,7.7) |
| Middle SDI | Botswana | 10 to 14 | 1.88 (-3.09,7.1) | -0.25 (-5.97,5.81) |
| Middle SDI | Botswana | 15 to 19 | 1.72 (-2.5,6.13) | -0.77 (-5.79,4.53) |
| Middle SDI | Botswana | 20 to 24 | 1.47 (-2.36,5.46) | -0.72 (-5.28,4.07) |
| Middle SDI | Botswana | 25 to 29 | 1.39 (-1.88,4.77) | -0.75 (-4.75,3.42) |
| Middle SDI | Botswana | 30 to 34 | 1.1 (-1.78,4.07) | -0.87 (-4.33,2.72) |
| Middle SDI | Botswana | 35 to 39 | 0.26 (-2.36,2.96) | -0.71 (-3.83,2.52) |
| Middle SDI | Botswana | 40 to 44 | -0.3 (-2.81,2.28) | -0.67 (-3.62,2.37) |
| Middle SDI | Botswana | 45 to 49 | -0.54 (-3.06,2.06) | -1.04 (-3.99,1.99) |
| Middle SDI | Botswana | 50 to 54 | -0.57 (-3.13,2.07) | -0.8 (-3.71,2.21) |
| Middle SDI | Botswana | 55 to 59 | -0.62 (-3.23,2.05) | -0.65 (-3.54,2.32) |
| Middle SDI | Botswana | 60 to 64 | -0.07 (-2.84,2.79) | -0.29 (-3.24,2.74) |
| Middle SDI | Botswana | 65 to 69 | -0.07 (-2.97,2.92) | -0.08 (-3.05,2.97) |
| Middle SDI | Botswana | 70 to 74 | 0.19 (-3.17,3.67) | -0.03 (-3.3,3.35) |
| Middle SDI | Botswana | 75 to 79 | 0.02 (-4.09,4.3) | -0.38 (-4.22,3.61) |
| Middle SDI | Botswana | 80 to 84 | -0.02 (-6.18,6.55) | 0.23 (-5.82,6.67) |
| Middle SDI | Botswana | 85 to 89 | 3.15 (-14.2,24.01) | 3.68 (-13.75,24.62) |
| Middle SDI | Brazil | 5 to 9 | -1.15 (-1.78,-0.51) | -2.74 (-3.76,-1.71) |
| Middle SDI | Brazil | 10 to 14 | 0.19 (-0.28,0.65) | -1.39 (-2.1,-0.68) |
| Middle SDI | Brazil | 15 to 19 | 0.85 (0.47,1.24) | -0.64 (-1.21,-0.07) |
| Middle SDI | Brazil | 20 to 24 | 0.99 (0.65,1.33) | -0.46 (-0.95,0.04) |
| Middle SDI | Brazil | 25 to 29 | 0.78 (0.47,1.08) | -0.6 (-1.06,-0.15) |
| Middle SDI | Brazil | 30 to 34 | 0.48 (0.21,0.75) | -0.82 (-1.23,-0.4) |
| Middle SDI | Brazil | 35 to 39 | 0.15 (-0.1,0.4) | -1.08 (-1.45,-0.71) |
| Middle SDI | Brazil | 40 to 44 | -0.09 (-0.33,0.14) | -1.26 (-1.6,-0.92) |
| Middle SDI | Brazil | 45 to 49 | -0.11 (-0.33,0.11) | -1.23 (-1.53,-0.92) |
| Middle SDI | Brazil | 50 to 54 | -0.03 (-0.23,0.18) | -1.1 (-1.37,-0.82) |
| Middle SDI | Brazil | 55 to 59 | 0.08 (-0.11,0.27) | -0.93 (-1.19,-0.68) |
| Middle SDI | Brazil | 60 to 64 | 0.15 (-0.03,0.34) | -0.8 (-1.03,-0.56) |
| Middle SDI | Brazil | 65 to 69 | 0.19 (0,0.38) | -0.66 (-0.89,-0.43) |
| Middle SDI | Brazil | 70 to 74 | 0.36 (0.15,0.57) | -0.41 (-0.65,-0.16) |
| Middle SDI | Brazil | 75 to 79 | 0.52 (0.26,0.78) | -0.16 (-0.44,0.12) |
| Middle SDI | Brazil | 80 to 84 | 0.72 (0.35,1.08) | 0.13 (-0.25,0.5) |
| Middle SDI | Brazil | 85 to 89 | 0.9 (0.26,1.54) | 0.35 (-0.29,0.99) |
| Middle SDI | China | 5 to 9 | 0.15 (-0.63,0.93) | -3.48 (-4.33,-2.63) |
| Middle SDI | China | 10 to 14 | 0.73 (0.09,1.38) | -2.58 (-3.22,-1.94) |
| Middle SDI | China | 15 to 19 | 1.37 (0.79,1.95) | -1.76 (-2.29,-1.22) |
| Middle SDI | China | 20 to 24 | 2.08 (1.58,2.59) | -1 (-1.45,-0.56) |
| Middle SDI | China | 25 to 29 | 2.45 (2.03,2.87) | -0.68 (-1.05,-0.3) |
| Middle SDI | China | 30 to 34 | 2.66 (2.3,3.02) | -0.47 (-0.8,-0.14) |
| Middle SDI | China | 35 to 39 | 2.49 (2.16,2.81) | -0.62 (-0.92,-0.32) |
| Middle SDI | China | 40 to 44 | 2.31 (2.03,2.6) | -0.75 (-1.01,-0.49) |
| Middle SDI | China | 45 to 49 | 2.24 (2,2.49) | -0.81 (-1.02,-0.59) |
| Middle SDI | China | 50 to 54 | 2.38 (2.16,2.61) | -0.66 (-0.85,-0.47) |
| Middle SDI | China | 55 to 59 | 2.34 (2.12,2.55) | -0.65 (-0.83,-0.48) |
| Middle SDI | China | 60 to 64 | 2.36 (2.15,2.57) | -0.51 (-0.68,-0.34) |
| Middle SDI | China | 65 to 69 | 2.25 (2.04,2.47) | -0.48 (-0.64,-0.31) |
| Middle SDI | China | 70 to 74 | 2.2 (1.95,2.44) | -0.4 (-0.57,-0.22) |
| Middle SDI | China | 75 to 79 | 2.07 (1.75,2.38) | -0.36 (-0.57,-0.14) |
| Middle SDI | China | 80 to 84 | 2.1 (1.62,2.58) | -0.2 (-0.5,0.1) |
| Middle SDI | China | 85 to 89 | 2.34 (1.45,3.23) | 0.02 (-0.51,0.57) |
| Middle SDI | Colombia | 5 to 9 | 0.16 (-0.76,1.09) | -2.44 (-4.16,-0.69) |
| Middle SDI | Colombia | 10 to 14 | 0.93 (0.19,1.68) | -1.68 (-2.95,-0.39) |
| Middle SDI | Colombia | 15 to 19 | 1.02 (0.37,1.68) | -1.46 (-2.52,-0.38) |
| Middle SDI | Colombia | 20 to 24 | 1.29 (0.7,1.89) | -1.09 (-2.05,-0.13) |
| Middle SDI | Colombia | 25 to 29 | 1.64 (1.09,2.18) | -0.7 (-1.58,0.2) |
| Middle SDI | Colombia | 30 to 34 | 1.72 (1.21,2.23) | -0.48 (-1.31,0.35) |
| Middle SDI | Colombia | 35 to 39 | 1.65 (1.16,2.14) | -0.46 (-1.24,0.32) |
| Middle SDI | Colombia | 40 to 44 | 1.44 (0.97,1.91) | -0.58 (-1.29,0.15) |
| Middle SDI | Colombia | 45 to 49 | 1.25 (0.81,1.69) | -0.66 (-1.32,0) |
| Middle SDI | Colombia | 50 to 54 | 1.16 (0.75,1.58) | -0.66 (-1.26,-0.06) |
| Middle SDI | Colombia | 55 to 59 | 1.24 (0.84,1.63) | -0.51 (-1.06,0.03) |
| Middle SDI | Colombia | 60 to 64 | 1.18 (0.8,1.56) | -0.45 (-0.96,0.06) |
| Middle SDI | Colombia | 65 to 69 | 1.21 (0.82,1.6) | -0.28 (-0.78,0.22) |
| Middle SDI | Colombia | 70 to 74 | 1.21 (0.78,1.65) | -0.15 (-0.67,0.37) |
| Middle SDI | Colombia | 75 to 79 | 1.25 (0.72,1.8) | 0.03 (-0.57,0.63) |
| Middle SDI | Colombia | 80 to 84 | 1.3 (0.52,2.08) | 0.22 (-0.59,1.03) |
| Middle SDI | Colombia | 85 to 89 | 1.89 (0.48,3.31) | 0.99 (-0.46,2.45) |
| Middle SDI | Costa Rica | 5 to 9 | 1.01 (-1.85,3.95) | -2.51 (-8.98,4.42) |
| Middle SDI | Costa Rica | 10 to 14 | 1.4 (-0.87,3.72) | -2.05 (-6.84,2.99) |
| Middle SDI | Costa Rica | 15 to 19 | 1.73 (-0.19,3.68) | -1.03 (-4.75,2.83) |
| Middle SDI | Costa Rica | 20 to 24 | 2 (0.33,3.69) | 0.04 (-2.99,3.17) |
| Middle SDI | Costa Rica | 25 to 29 | 2.08 (0.62,3.56) | -0.01 (-2.76,2.82) |
| Middle SDI | Costa Rica | 30 to 34 | 2 (0.67,3.35) | 0.03 (-2.45,2.56) |
| Middle SDI | Costa Rica | 35 to 39 | 1.76 (0.5,3.04) | 0.09 (-2.17,2.4) |
| Middle SDI | Costa Rica | 40 to 44 | 1.52 (0.33,2.73) | -0.06 (-2.11,2.03) |
| Middle SDI | Costa Rica | 45 to 49 | 1.33 (0.22,2.46) | -0.05 (-1.91,1.84) |
| Middle SDI | Costa Rica | 50 to 54 | 1.14 (0.1,2.19) | -0.17 (-1.84,1.53) |
| Middle SDI | Costa Rica | 55 to 59 | 1.02 (0.05,2) | -0.07 (-1.58,1.46) |
| Middle SDI | Costa Rica | 60 to 64 | 0.98 (0.05,1.92) | -0.08 (-1.47,1.34) |
| Middle SDI | Costa Rica | 65 to 69 | 0.88 (-0.06,1.83) | -0.09 (-1.43,1.26) |
| Middle SDI | Costa Rica | 70 to 74 | 0.81 (-0.21,1.84) | -0.19 (-1.55,1.18) |
| Middle SDI | Costa Rica | 75 to 79 | 0.93 (-0.29,2.17) | 0.14 (-1.37,1.68) |
| Middle SDI | Costa Rica | 80 to 84 | 0.84 (-0.79,2.51) | 0.22 (-1.67,2.15) |
| Middle SDI | Costa Rica | 85 to 89 | 1.59 (-1.25,4.51) | 0.95 (-2.25,4.25) |
| Middle SDI | Cuba | 5 to 9 | 0.04 (-1.69,1.81) | -2.28 (-6.24,1.84) |
| Middle SDI | Cuba | 10 to 14 | -0.69 (-2.25,0.89) | -2.94 (-6.22,0.45) |
| Middle SDI | Cuba | 15 to 19 | -0.68 (-1.97,0.63) | -2.36 (-4.73,0.07) |
| Middle SDI | Cuba | 20 to 24 | -0.61 (-1.71,0.49) | -2.2 (-4.16,-0.21) |
| Middle SDI | Cuba | 25 to 29 | -0.44 (-1.36,0.49) | -1.89 (-3.55,-0.2) |
| Middle SDI | Cuba | 30 to 34 | -0.12 (-0.92,0.68) | -1.47 (-2.93,0.01) |
| Middle SDI | Cuba | 35 to 39 | 0.11 (-0.66,0.89) | -1.14 (-2.48,0.22) |
| Middle SDI | Cuba | 40 to 44 | 0.24 (-0.45,0.94) | -0.92 (-2.07,0.25) |
| Middle SDI | Cuba | 45 to 49 | 0.29 (-0.31,0.9) | -0.84 (-1.81,0.14) |
| Middle SDI | Cuba | 50 to 54 | 0.45 (-0.09,1) | -0.65 (-1.5,0.22) |
| Middle SDI | Cuba | 55 to 59 | 0.62 (0.1,1.14) | -0.45 (-1.23,0.34) |
| Middle SDI | Cuba | 60 to 64 | 0.82 (0.32,1.33) | -0.22 (-0.96,0.53) |
| Middle SDI | Cuba | 65 to 69 | 0.91 (0.41,1.42) | -0.06 (-0.77,0.65) |
| Middle SDI | Cuba | 70 to 74 | 0.79 (0.26,1.33) | -0.14 (-0.86,0.58) |
| Middle SDI | Cuba | 75 to 79 | 0.55 (-0.07,1.18) | -0.34 (-1.12,0.44) |
| Middle SDI | Cuba | 80 to 84 | 0.36 (-0.48,1.21) | -0.49 (-1.46,0.5) |
| Middle SDI | Cuba | 85 to 89 | 0.42 (-0.92,1.79) | -0.36 (-1.89,1.2) |
| Middle SDI | Ecuador | 5 to 9 | 0.27 (-1.44,2) | -1.84 (-4.6,0.99) |
| Middle SDI | Ecuador | 10 to 14 | 1.17 (-0.07,2.42) | -0.89 (-2.87,1.13) |
| Middle SDI | Ecuador | 15 to 19 | 1.98 (0.99,2.97) | -0.06 (-1.7,1.62) |
| Middle SDI | Ecuador | 20 to 24 | 2.48 (1.64,3.32) | 0.25 (-1.26,1.78) |
| Middle SDI | Ecuador | 25 to 29 | 2.79 (2.05,3.54) | 0.48 (-0.95,1.93) |
| Middle SDI | Ecuador | 30 to 34 | 2.96 (2.27,3.65) | 0.66 (-0.69,2.03) |
| Middle SDI | Ecuador | 35 to 39 | 2.88 (2.22,3.54) | 0.62 (-0.66,1.91) |
| Middle SDI | Ecuador | 40 to 44 | 2.82 (2.18,3.47) | 0.57 (-0.62,1.79) |
| Middle SDI | Ecuador | 45 to 49 | 2.99 (2.37,3.61) | 0.82 (-0.3,1.95) |
| Middle SDI | Ecuador | 50 to 54 | 3.43 (2.84,4.02) | 1.26 (0.23,2.31) |
| Middle SDI | Ecuador | 55 to 59 | 3.7 (3.14,4.27) | 1.53 (0.57,2.49) |
| Middle SDI | Ecuador | 60 to 64 | 3.7 (3.16,4.24) | 1.55 (0.66,2.45) |
| Middle SDI | Ecuador | 65 to 69 | 3.64 (3.08,4.19) | 1.55 (0.68,2.43) |
| Middle SDI | Ecuador | 70 to 74 | 3.68 (3.07,4.3) | 1.68 (0.79,2.59) |
| Middle SDI | Ecuador | 75 to 79 | 3.88 (3.1,4.66) | 1.91 (0.89,2.94) |
| Middle SDI | Ecuador | 80 to 84 | 3.78 (2.71,4.86) | 2.02 (0.72,3.33) |
| Middle SDI | Ecuador | 85 to 89 | 3.88 (1.97,5.82) | 2.44 (0.2,4.74) |
| Middle SDI | Equatorial Guinea | 5 to 9 | -0.14 (-9.01,9.6) | 6.53 (-5.96,20.68) |
| Middle SDI | Equatorial Guinea | 10 to 14 | 1.17 (-7.05,10.11) | 6.71 (-4.34,19.04) |
| Middle SDI | Equatorial Guinea | 15 to 19 | 2.35 (-5.48,10.84) | 5.39 (-4.64,16.48) |
| Middle SDI | Equatorial Guinea | 20 to 24 | 3.05 (-4.47,11.17) | 3.37 (-6.47,14.25) |
| Middle SDI | Equatorial Guinea | 25 to 29 | 2.57 (-4.98,10.72) | 2.82 (-6.96,13.62) |
| Middle SDI | Equatorial Guinea | 30 to 34 | 1.9 (-5.48,9.85) | 3.44 (-6.14,14.01) |
| Middle SDI | Equatorial Guinea | 35 to 39 | 1.92 (-5.11,9.48) | 2.82 (-6.21,12.73) |
| Middle SDI | Equatorial Guinea | 40 to 44 | 0.47 (-5.94,7.32) | 2.31 (-5.93,11.26) |
| Middle SDI | Equatorial Guinea | 45 to 49 | -0.44 (-5.85,5.29) | 0.96 (-5.96,8.4) |
| Middle SDI | Equatorial Guinea | 50 to 54 | -0.23 (-5.17,4.96) | 0.53 (-5.02,6.42) |
| Middle SDI | Equatorial Guinea | 55 to 59 | -0.23 (-4.93,4.71) | -0.08 (-5.12,5.22) |
| Middle SDI | Equatorial Guinea | 60 to 64 | -0.3 (-4.97,4.6) | -0.51 (-5.36,4.59) |
| Middle SDI | Equatorial Guinea | 65 to 69 | -0.13 (-5.28,5.3) | -1.18 (-6.14,4.04) |
| Middle SDI | Equatorial Guinea | 70 to 74 | 0.03 (-6.33,6.82) | -0.25 (-6.5,6.42) |
| Middle SDI | Equatorial Guinea | 75 to 79 | 2.41 (-8.95,15.18) | 2.34 (-9.01,15.09) |
| Middle SDI | Equatorial Guinea | 80 to 84 | 3.02 (-12.01,20.62) | 3.06 (-11.98,20.66) |
| Middle SDI | Equatorial Guinea | 85 to 89 | 0.39 (-20.21,26.3) | 0.59 (-20.06,26.57) |
| Middle SDI | Fiji | 5 to 9 | 2.87 (-5.61,12.11) | -0.15 (-21.23,26.58) |
| Middle SDI | Fiji | 10 to 14 | 1.2 (-5,7.81) | 0.58 (-16.25,20.8) |
| Middle SDI | Fiji | 15 to 19 | 0.43 (-5.3,6.5) | 0.85 (-13.54,17.63) |
| Middle SDI | Fiji | 20 to 24 | 0.99 (-4.17,6.43) | 1.06 (-11.22,15.03) |
| Middle SDI | Fiji | 25 to 29 | 1.66 (-3.19,6.76) | 1.33 (-8.3,11.97) |
| Middle SDI | Fiji | 30 to 34 | 0.92 (-3.86,5.93) | 0.89 (-7.9,10.51) |
| Middle SDI | Fiji | 35 to 39 | 0.82 (-3.9,5.78) | 0.46 (-6.83,8.31) |
| Middle SDI | Fiji | 40 to 44 | 0.16 (-4.65,5.21) | 0.64 (-5.97,7.71) |
| Middle SDI | Fiji | 45 to 49 | 0.39 (-4.29,5.3) | -0.07 (-5.88,6.1) |
| Middle SDI | Fiji | 50 to 54 | 0.23 (-4.42,5.11) | -0.39 (-5.93,5.48) |
| Middle SDI | Fiji | 55 to 59 | 0.42 (-4.13,5.17) | -0.51 (-5.54,4.8) |
| Middle SDI | Fiji | 60 to 64 | 0.04 (-4.53,4.83) | -1.19 (-6.15,4.04) |
| Middle SDI | Fiji | 65 to 69 | -0.28 (-5.16,4.85) | -2.04 (-7.05,3.24) |
| Middle SDI | Fiji | 70 to 74 | 0.44 (-5.71,6.99) | -0.45 (-6.54,6.03) |
| Middle SDI | Fiji | 75 to 79 | 3.72 (-7.72,16.57) | 3.39 (-8.04,16.23) |
| Middle SDI | Fiji | 80 to 84 | 5.14 (-10.15,23.04) | 4.98 (-10.3,22.86) |
| Middle SDI | Fiji | 85 to 89 | 3.28 (-17.85,29.84) | 2.92 (-18.18,29.47) |
| Middle SDI | Gabon | 5 to 9 | -1.15 (-8.28,6.53) | -1.65 (-8.82,6.09) |
| Middle SDI | Gabon | 10 to 14 | -0.51 (-6,5.3) | -1.7 (-7.41,4.37) |
| Middle SDI | Gabon | 15 to 19 | 0.27 (-4.35,5.11) | -1.85 (-7.01,3.6) |
| Middle SDI | Gabon | 20 to 24 | 0.49 (-3.84,5.02) | -1.46 (-6.36,3.7) |
| Middle SDI | Gabon | 25 to 29 | 0.67 (-3.33,4.82) | -0.74 (-5.42,4.16) |
| Middle SDI | Gabon | 30 to 34 | 1.06 (-2.67,4.93) | -0.18 (-4.52,4.36) |
| Middle SDI | Gabon | 35 to 39 | 1.18 (-2.32,4.79) | -0.07 (-4.17,4.22) |
| Middle SDI | Gabon | 40 to 44 | 1.11 (-2.13,4.45) | 0.21 (-3.53,4.1) |
| Middle SDI | Gabon | 45 to 49 | 0.7 (-2.37,3.85) | -0.12 (-3.63,3.52) |
| Middle SDI | Gabon | 50 to 54 | 0.56 (-2.31,3.51) | -0.31 (-3.49,2.97) |
| Middle SDI | Gabon | 55 to 59 | 0.15 (-2.55,2.92) | -0.24 (-3.14,2.74) |
| Middle SDI | Gabon | 60 to 64 | 0.32 (-2.34,3.06) | -0.2 (-3,2.69) |
| Middle SDI | Gabon | 65 to 69 | 0.72 (-2.15,3.68) | -0.01 (-2.87,2.93) |
| Middle SDI | Gabon | 70 to 74 | 0.89 (-2.32,4.2) | 0.13 (-3,3.37) |
| Middle SDI | Gabon | 75 to 79 | 0.76 (-3.1,4.78) | 0.55 (-3.29,4.55) |
| Middle SDI | Gabon | 80 to 84 | 1.37 (-4.86,8) | 0.59 (-4.52,5.98) |
| Middle SDI | Gabon | 85 to 89 | 6.82 (-11.14,28.41) | -0.1 (-7.22,7.55) |
| Middle SDI | Grenada | 5 to 9 | 1.18 (-20.12,28.18) | 1.95 (-19.71,29.45) |
| Middle SDI | Grenada | 10 to 14 | 0.75 (-16.06,20.92) | 1.22 (-16.27,22.36) |
| Middle SDI | Grenada | 15 to 19 | 0.5 (-13.67,17) | 1.15 (-14.96,20.31) |
| Middle SDI | Grenada | 20 to 24 | 0.16 (-11.13,12.88) | -0.04 (-15.24,17.87) |
| Middle SDI | Grenada | 25 to 29 | -0.19 (-7.49,7.68) | -1.43 (-16.06,15.75) |
| Middle SDI | Grenada | 30 to 34 | -0.64 (-6.62,5.73) | -2.06 (-15.9,14.07) |
| Middle SDI | Grenada | 35 to 39 | -0.63 (-6.03,5.08) | -1.77 (-14.4,12.72) |
| Middle SDI | Grenada | 40 to 44 | -0.71 (-5.77,4.63) | -1.12 (-12.01,11.11) |
| Middle SDI | Grenada | 45 to 49 | -0.08 (-5.13,5.24) | 0.87 (-7.79,10.34) |
| Middle SDI | Grenada | 50 to 54 | 0.41 (-4.37,5.43) | 1.15 (-6.25,9.13) |
| Middle SDI | Grenada | 55 to 59 | 0.8 (-3.85,5.68) | -0.8 (-6.92,5.73) |
| Middle SDI | Grenada | 60 to 64 | 0.4 (-4.26,5.29) | -2.35 (-7.81,3.43) |
| Middle SDI | Grenada | 65 to 69 | 0.11 (-4.54,4.98) | -2.94 (-8.1,2.51) |
| Middle SDI | Grenada | 70 to 74 | 0.04 (-4.87,5.2) | -2.59 (-7.89,3.02) |
| Middle SDI | Grenada | 75 to 79 | 0.9 (-4.77,6.9) | -1.25 (-7.16,5.03) |
| Middle SDI | Grenada | 80 to 84 | 1.78 (-5.85,10.04) | 0.37 (-7.3,8.67) |
| Middle SDI | Grenada | 85 to 89 | 2.91 (-16.15,26.3) | 1.31 (-17.56,24.49) |
| Middle SDI | Guyana | 5 to 9 | 1.62 (-6.61,10.57) | -5.18 (-23.29,17.19) |
| Middle SDI | Guyana | 10 to 14 | 1.74 (-6.31,10.49) | -3.31 (-17.85,13.8) |
| Middle SDI | Guyana | 15 to 19 | 2.24 (-4.68,9.66) | -1.9 (-13.48,11.24) |
| Middle SDI | Guyana | 20 to 24 | 1.44 (-4.62,7.9) | -0.12 (-6.98,7.24) |
| Middle SDI | Guyana | 25 to 29 | 1.69 (-3.78,7.47) | 0.68 (-5.09,6.79) |
| Middle SDI | Guyana | 30 to 34 | 1.4 (-3.7,6.78) | 0.22 (-5.31,6.06) |
| Middle SDI | Guyana | 35 to 39 | 1.16 (-3.64,6.19) | 0.68 (-4.45,6.09) |
| Middle SDI | Guyana | 40 to 44 | 1.59 (-2.91,6.3) | -0.03 (-4.96,5.15) |
| Middle SDI | Guyana | 45 to 49 | 1.08 (-3.22,5.57) | -0.39 (-5.12,4.58) |
| Middle SDI | Guyana | 50 to 54 | 0.92 (-3.34,5.37) | 0.4 (-4.19,5.21) |
| Middle SDI | Guyana | 55 to 59 | 1.69 (-2.61,6.17) | 0.68 (-3.83,5.4) |
| Middle SDI | Guyana | 60 to 64 | 1.82 (-2.64,6.48) | 0.68 (-3.9,5.48) |
| Middle SDI | Guyana | 65 to 69 | 1.78 (-3.03,6.84) | 0.81 (-3.93,5.79) |
| Middle SDI | Guyana | 70 to 74 | 1.62 (-3.75,7.29) | 0.68 (-4.7,6.35) |
| Middle SDI | Guyana | 75 to 79 | 0.33 (-6.41,7.55) | -0.19 (-6.96,7.08) |
| Middle SDI | Guyana | 80 to 84 | 1.54 (-11.91,17.06) | 2.34 (-11.22,17.98) |
| Middle SDI | Guyana | 85 to 89 | 0.78 (-19.5,26.16) | 1.57 (-18.87,27.14) |
| Middle SDI | Indonesia | 5 to 9 | -0.2 (-0.88,0.49) | -1.46 (-2.36,-0.56) |
| Middle SDI | Indonesia | 10 to 14 | 0.12 (-0.39,0.63) | -0.96 (-1.6,-0.32) |
| Middle SDI | Indonesia | 15 to 19 | 0.42 (0,0.85) | -0.52 (-1.03,-0.01) |
| Middle SDI | Indonesia | 20 to 24 | 0.51 (0.13,0.9) | -0.34 (-0.79,0.11) |
| Middle SDI | Indonesia | 25 to 29 | 0.59 (0.23,0.95) | -0.24 (-0.66,0.19) |
| Middle SDI | Indonesia | 30 to 34 | 0.58 (0.24,0.92) | -0.19 (-0.59,0.21) |
| Middle SDI | Indonesia | 35 to 39 | 0.5 (0.18,0.82) | -0.19 (-0.57,0.18) |
| Middle SDI | Indonesia | 40 to 44 | 0.48 (0.17,0.78) | -0.13 (-0.48,0.21) |
| Middle SDI | Indonesia | 45 to 49 | 0.5 (0.21,0.78) | -0.04 (-0.36,0.29) |
| Middle SDI | Indonesia | 50 to 54 | 0.5 (0.23,0.77) | 0.04 (-0.26,0.33) |
| Middle SDI | Indonesia | 55 to 59 | 0.55 (0.29,0.8) | 0.12 (-0.15,0.4) |
| Middle SDI | Indonesia | 60 to 64 | 0.62 (0.37,0.87) | 0.23 (-0.03,0.5) |
| Middle SDI | Indonesia | 65 to 69 | 0.72 (0.46,0.99) | 0.39 (0.12,0.66) |
| Middle SDI | Indonesia | 70 to 74 | 0.89 (0.57,1.21) | 0.59 (0.28,0.9) |
| Middle SDI | Indonesia | 75 to 79 | 1.1 (0.67,1.54) | 0.85 (0.45,1.25) |
| Middle SDI | Indonesia | 80 to 84 | 1.34 (0.69,1.99) | 1.13 (0.55,1.72) |
| Middle SDI | Indonesia | 85 to 89 | 1.67 (0.53,2.83) | 1.48 (0.47,2.5) |
| Middle SDI | Iran (Islamic Republic of) | 5 to 9 | 1.68 (0.98,2.39) | -1.43 (-2.96,0.11) |
| Middle SDI | Iran (Islamic Republic of) | 10 to 14 | 3.32 (2.75,3.9) | 0.32 (-0.81,1.46) |
| Middle SDI | Iran (Islamic Republic of) | 15 to 19 | 3.68 (3.13,4.23) | 0.95 (-0.03,1.94) |
| Middle SDI | Iran (Islamic Republic of) | 20 to 24 | 3.59 (3.05,4.13) | 1 (0.07,1.93) |
| Middle SDI | Iran (Islamic Republic of) | 25 to 29 | 3.46 (2.95,3.98) | 0.96 (0.09,1.85) |
| Middle SDI | Iran (Islamic Republic of) | 30 to 34 | 3.32 (2.81,3.84) | 0.85 (0.01,1.69) |
| Middle SDI | Iran (Islamic Republic of) | 35 to 39 | 3.12 (2.61,3.64) | 0.73 (-0.08,1.55) |
| Middle SDI | Iran (Islamic Republic of) | 40 to 44 | 2.89 (2.36,3.41) | 0.62 (-0.18,1.41) |
| Middle SDI | Iran (Islamic Republic of) | 45 to 49 | 2.69 (2.17,3.21) | 0.55 (-0.2,1.31) |
| Middle SDI | Iran (Islamic Republic of) | 50 to 54 | 2.47 (1.96,2.99) | 0.52 (-0.18,1.23) |
| Middle SDI | Iran (Islamic Republic of) | 55 to 59 | 2.38 (1.88,2.88) | 0.59 (-0.06,1.25) |
| Middle SDI | Iran (Islamic Republic of) | 60 to 64 | 2.39 (1.9,2.88) | 0.76 (0.15,1.37) |
| Middle SDI | Iran (Islamic Republic of) | 65 to 69 | 2.35 (1.83,2.86) | 0.92 (0.3,1.53) |
| Middle SDI | Iran (Islamic Republic of) | 70 to 74 | 2.34 (1.76,2.93) | 1.07 (0.42,1.72) |
| Middle SDI | Iran (Islamic Republic of) | 75 to 79 | 2.3 (1.52,3.09) | 1.22 (0.42,2.03) |
| Middle SDI | Iran (Islamic Republic of) | 80 to 84 | 2.13 (0.74,3.54) | 1.21 (-0.13,2.57) |
| Middle SDI | Iran (Islamic Republic of) | 85 to 89 | 2.08 (-0.63,4.86) | 1.11 (-1.37,3.65) |
| Middle SDI | Iraq | 5 to 9 | -0.53 (-1.38,0.33) | -3.57 (-5.06,-2.06) |
| Middle SDI | Iraq | 10 to 14 | 0.34 (-0.42,1.12) | -2.48 (-3.72,-1.23) |
| Middle SDI | Iraq | 15 to 19 | 0.79 (0.03,1.55) | -1.79 (-2.94,-0.63) |
| Middle SDI | Iraq | 20 to 24 | 0.92 (0.17,1.67) | -1.5 (-2.6,-0.38) |
| Middle SDI | Iraq | 25 to 29 | 1.01 (0.28,1.74) | -1.37 (-2.45,-0.29) |
| Middle SDI | Iraq | 30 to 34 | 1.01 (0.3,1.73) | -1.35 (-2.38,-0.31) |
| Middle SDI | Iraq | 35 to 39 | 0.96 (0.28,1.65) | -1.29 (-2.25,-0.32) |
| Middle SDI | Iraq | 40 to 44 | 0.87 (0.22,1.53) | -1.21 (-2.1,-0.32) |
| Middle SDI | Iraq | 45 to 49 | 0.78 (0.16,1.41) | -1.12 (-1.93,-0.29) |
| Middle SDI | Iraq | 50 to 54 | 0.79 (0.17,1.42) | -0.92 (-1.7,-0.14) |
| Middle SDI | Iraq | 55 to 59 | 0.91 (0.28,1.55) | -0.64 (-1.4,0.12) |
| Middle SDI | Iraq | 60 to 64 | 0.99 (0.33,1.65) | -0.41 (-1.17,0.35) |
| Middle SDI | Iraq | 65 to 69 | 1.11 (0.39,1.84) | -0.1 (-0.89,0.7) |
| Middle SDI | Iraq | 70 to 74 | 1.2 (0.35,2.05) | 0.21 (-0.68,1.1) |
| Middle SDI | Iraq | 75 to 79 | 1.32 (0.24,2.41) | 0.45 (-0.61,1.53) |
| Middle SDI | Iraq | 80 to 84 | 1.49 (-0.05,3.05) | 0.82 (-0.63,2.3) |
| Middle SDI | Iraq | 85 to 89 | 1.97 (-0.68,4.69) | 1.06 (-1.28,3.46) |
| Middle SDI | Jamaica | 5 to 9 | -1.02 (-4.15,2.21) | -2.94 (-9.26,3.81) |
| Middle SDI | Jamaica | 10 to 14 | -0.51 (-3.15,2.19) | -2.16 (-6.97,2.89) |
| Middle SDI | Jamaica | 15 to 19 | -0.11 (-2.49,2.33) | -0.6 (-4.33,3.27) |
| Middle SDI | Jamaica | 20 to 24 | 0.23 (-1.87,2.37) | -0.48 (-3.69,2.84) |
| Middle SDI | Jamaica | 25 to 29 | 0.03 (-1.82,1.92) | -0.4 (-3.23,2.51) |
| Middle SDI | Jamaica | 30 to 34 | 0.1 (-1.6,1.82) | -0.48 (-3.08,2.19) |
| Middle SDI | Jamaica | 35 to 39 | -0.05 (-1.68,1.61) | -0.46 (-2.89,2.03) |
| Middle SDI | Jamaica | 40 to 44 | 0.05 (-1.5,1.61) | -0.45 (-2.71,1.85) |
| Middle SDI | Jamaica | 45 to 49 | 0.26 (-1.16,1.7) | -0.27 (-2.27,1.76) |
| Middle SDI | Jamaica | 50 to 54 | 0.5 (-0.81,1.83) | -0.01 (-1.82,1.84) |
| Middle SDI | Jamaica | 55 to 59 | 0.57 (-0.67,1.83) | 0.04 (-1.63,1.74) |
| Middle SDI | Jamaica | 60 to 64 | 0.6 (-0.6,1.81) | 0.17 (-1.4,1.77) |
| Middle SDI | Jamaica | 65 to 69 | 0.64 (-0.57,1.87) | 0.21 (-1.31,1.75) |
| Middle SDI | Jamaica | 70 to 74 | 0.8 (-0.5,2.11) | 0.34 (-1.21,1.91) |
| Middle SDI | Jamaica | 75 to 79 | 0.94 (-0.59,2.5) | 0.43 (-1.31,2.19) |
| Middle SDI | Jamaica | 80 to 84 | 1.08 (-1,3.2) | 0.67 (-1.55,2.95) |
| Middle SDI | Jamaica | 85 to 89 | 0.68 (-2.66,4.13) | 0.07 (-3.34,3.6) |
| Middle SDI | Maldives | 5 to 9 | -0.87 (-21.91,25.84) | - |
| Middle SDI | Maldives | 10 to 14 | -0.55 (-17.38,19.72) | - |
| Middle SDI | Maldives | 15 to 19 | 1.15 (-14.01,18.98) | - |
| Middle SDI | Maldives | 20 to 24 | 2.15 (-11.49,17.9) | - |
| Middle SDI | Maldives | 25 to 29 | 3.78 (-8.21,17.34) | - |
| Middle SDI | Maldives | 30 to 34 | 5.14 (-6.32,18.01) | - |
| Middle SDI | Maldives | 35 to 39 | 4.95 (-6.51,17.82) | - |
| Middle SDI | Maldives | 40 to 44 | 4.75 (-7.22,18.27) | - |
| Middle SDI | Maldives | 45 to 49 | 5.29 (-6.44,18.49) | - |
| Middle SDI | Maldives | 50 to 54 | 4.49 (-7.51,18.05) | - |
| Middle SDI | Maldives | 55 to 59 | 2.28 (-8.96,14.91) | - |
| Middle SDI | Maldives | 60 to 64 | 0.87 (-9.26,12.13) | - |
| Middle SDI | Maldives | 65 to 69 | 0.75 (-10.59,13.52) | - |
| Middle SDI | Maldives | 70 to 74 | 0.35 (-12.97,15.71) | - |
| Middle SDI | Maldives | 75 to 79 | -1.42 (-16.15,15.89) | - |
| Middle SDI | Maldives | 80 to 84 | -3.32 (-19.74,16.47) | - |
| Middle SDI | Maldives | 85 to 89 | -6.11 (-26.15,19.37) | - |
| Middle SDI | Mauritius | 5 to 9 | 1.58 (-19.09,27.55) | 2.2 (-19.23,29.32) |
| Middle SDI | Mauritius | 10 to 14 | 1.5 (-12.76,18.08) | 3.44 (-13.35,23.48) |
| Middle SDI | Mauritius | 15 to 19 | 2.19 (-5.5,10.51) | 4.1 (-9.53,19.79) |
| Middle SDI | Mauritius | 20 to 24 | 2.46 (-3.4,8.68) | 5.44 (-4.62,16.57) |
| Middle SDI | Mauritius | 25 to 29 | 3.17 (-1.69,8.26) | 1.63 (-5.92,9.78) |
| Middle SDI | Mauritius | 30 to 34 | 3.25 (-1.3,8) | 1.82 (-4.12,8.14) |
| Middle SDI | Mauritius | 35 to 39 | 3.27 (-0.74,7.43) | 1.38 (-3.65,6.68) |
| Middle SDI | Mauritius | 40 to 44 | 2.51 (-1.27,6.42) | 1.15 (-3.44,5.96) |
| Middle SDI | Mauritius | 45 to 49 | 2.52 (-1.02,6.19) | 1 (-3.42,5.62) |
| Middle SDI | Mauritius | 50 to 54 | 2.01 (-1.33,5.47) | 1.32 (-2.63,5.43) |
| Middle SDI | Mauritius | 55 to 59 | 1.53 (-1.59,4.75) | 0.88 (-2.79,4.68) |
| Middle SDI | Mauritius | 60 to 64 | 1.57 (-1.48,4.73) | 0.93 (-2.46,4.43) |
| Middle SDI | Mauritius | 65 to 69 | 1.45 (-1.74,4.74) | 0.78 (-2.56,4.24) |
| Middle SDI | Mauritius | 70 to 74 | 1.21 (-2.44,5) | 0.62 (-3.09,4.46) |
| Middle SDI | Mauritius | 75 to 79 | 0.81 (-4.04,5.91) | 0.68 (-4.19,5.79) |
| Middle SDI | Mauritius | 80 to 84 | -0.15 (-6.69,6.86) | 0.07 (-6.52,7.13) |
| Middle SDI | Mauritius | 85 to 89 | 3.24 (-14.18,24.21) | 3.77 (-13.75,24.87) |
| Middle SDI | Mexico | 5 to 9 | -0.66 (-1.43,0.12) | -2.27 (-3.56,-0.95) |
| Middle SDI | Mexico | 10 to 14 | 0.46 (-0.13,1.05) | -1.11 (-2.03,-0.19) |
| Middle SDI | Mexico | 15 to 19 | 0.95 (0.44,1.45) | -0.55 (-1.3,0.21) |
| Middle SDI | Mexico | 20 to 24 | 1.25 (0.8,1.7) | -0.15 (-0.82,0.53) |
| Middle SDI | Mexico | 25 to 29 | 1.42 (1.02,1.83) | 0.09 (-0.53,0.72) |
| Middle SDI | Mexico | 30 to 34 | 1.33 (0.95,1.71) | 0.09 (-0.49,0.67) |
| Middle SDI | Mexico | 35 to 39 | 1.34 (0.98,1.71) | 0.15 (-0.39,0.69) |
| Middle SDI | Mexico | 40 to 44 | 1.36 (1.02,1.7) | 0.23 (-0.27,0.72) |
| Middle SDI | Mexico | 45 to 49 | 1.42 (1.1,1.74) | 0.34 (-0.11,0.8) |
| Middle SDI | Mexico | 50 to 54 | 1.42 (1.12,1.73) | 0.43 (0.01,0.85) |
| Middle SDI | Mexico | 55 to 59 | 1.38 (1.09,1.67) | 0.45 (0.07,0.84) |
| Middle SDI | Mexico | 60 to 64 | 1.29 (1.01,1.58) | 0.44 (0.08,0.81) |
| Middle SDI | Mexico | 65 to 69 | 1.25 (0.95,1.54) | 0.47 (0.12,0.83) |
| Middle SDI | Mexico | 70 to 74 | 1.16 (0.84,1.48) | 0.47 (0.1,0.84) |
| Middle SDI | Mexico | 75 to 79 | 1.49 (1.09,1.89) | 0.9 (0.47,1.34) |
| Middle SDI | Mexico | 80 to 84 | 1.43 (0.89,1.97) | 0.95 (0.39,1.5) |
| Middle SDI | Mexico | 85 to 89 | 0.43 (-0.35,1.22) | 0.05 (-0.74,0.85) |
| Middle SDI | Panama | 5 to 9 | 0.87 (-2.24,4.07) | -0.4 (-5.67,5.16) |
| Middle SDI | Panama | 10 to 14 | 1.25 (-1.42,3.98) | -0.07 (-4.44,4.49) |
| Middle SDI | Panama | 15 to 19 | 1.71 (-0.73,4.2) | 0.04 (-3.92,4.16) |
| Middle SDI | Panama | 20 to 24 | 2.28 (0.05,4.56) | 0.33 (-3.33,4.12) |
| Middle SDI | Panama | 25 to 29 | 2.33 (0.26,4.46) | 0.52 (-2.86,4.03) |
| Middle SDI | Panama | 30 to 34 | 2.24 (0.27,4.25) | 0.67 (-2.48,3.92) |
| Middle SDI | Panama | 35 to 39 | 2.13 (0.24,4.06) | 0.65 (-2.28,3.66) |
| Middle SDI | Panama | 40 to 44 | 1.85 (0.07,3.67) | 0.41 (-2.33,3.22) |
| Middle SDI | Panama | 45 to 49 | 1.77 (0.13,3.45) | 0.28 (-2.21,2.83) |
| Middle SDI | Panama | 50 to 54 | 1.77 (0.23,3.32) | 0.49 (-1.76,2.78) |
| Middle SDI | Panama | 55 to 59 | 1.6 (0.15,3.07) | 0.45 (-1.6,2.54) |
| Middle SDI | Panama | 60 to 64 | 1.23 (-0.16,2.63) | 0.34 (-1.57,2.29) |
| Middle SDI | Panama | 65 to 69 | 1.08 (-0.31,2.49) | 0.26 (-1.58,2.12) |
| Middle SDI | Panama | 70 to 74 | 1 (-0.47,2.49) | 0.43 (-1.41,2.31) |
| Middle SDI | Panama | 75 to 79 | 0.99 (-0.74,2.75) | 0.36 (-1.63,2.4) |
| Middle SDI | Panama | 80 to 84 | 0.82 (-1.49,3.18) | 0.66 (-1.96,3.34) |
| Middle SDI | Panama | 85 to 89 | 1.84 (-2.53,6.41) | 1.16 (-3.31,5.83) |
| Middle SDI | Paraguay | 5 to 9 | 0.45 (-2.23,3.21) | -0.83 (-5.19,3.73) |
| Middle SDI | Paraguay | 10 to 14 | 0.6 (-1.81,3.07) | -1.1 (-4.93,2.89) |
| Middle SDI | Paraguay | 15 to 19 | 1.42 (-0.84,3.73) | -0.08 (-3.41,3.37) |
| Middle SDI | Paraguay | 20 to 24 | 1.66 (-0.51,3.86) | 0.25 (-2.88,3.48) |
| Middle SDI | Paraguay | 25 to 29 | 1.36 (-0.7,3.47) | 0.14 (-2.87,3.24) |
| Middle SDI | Paraguay | 30 to 34 | 1.36 (-0.61,3.37) | 0.2 (-2.67,3.16) |
| Middle SDI | Paraguay | 35 to 39 | 1.42 (-0.46,3.33) | 0.58 (-2.12,3.35) |
| Middle SDI | Paraguay | 40 to 44 | 1.44 (-0.33,3.24) | 0.78 (-1.72,3.33) |
| Middle SDI | Paraguay | 45 to 49 | 1.54 (-0.09,3.19) | 0.87 (-1.39,3.18) |
| Middle SDI | Paraguay | 50 to 54 | 1.68 (0.18,3.2) | 0.87 (-1.14,2.93) |
| Middle SDI | Paraguay | 55 to 59 | 1.75 (0.34,3.17) | 0.91 (-0.92,2.78) |
| Middle SDI | Paraguay | 60 to 64 | 1.79 (0.42,3.17) | 0.9 (-0.81,2.63) |
| Middle SDI | Paraguay | 65 to 69 | 1.72 (0.34,3.13) | 1 (-0.67,2.69) |
| Middle SDI | Paraguay | 70 to 74 | 1.9 (0.37,3.45) | 1.31 (-0.43,3.09) |
| Middle SDI | Paraguay | 75 to 79 | 1.91 (0.08,3.78) | 1.61 (-0.39,3.65) |
| Middle SDI | Paraguay | 80 to 84 | 2.28 (-0.3,4.93) | 1.88 (-0.76,4.58) |
| Middle SDI | Paraguay | 85 to 89 | 2.86 (-1.62,7.54) | 2.49 (-2.02,7.2) |
| Middle SDI | Peru | 5 to 9 | 2.02 (0.98,3.06) | -1.38 (-3.15,0.43) |
| Middle SDI | Peru | 10 to 14 | 2.63 (1.84,3.44) | -0.9 (-2.27,0.5) |
| Middle SDI | Peru | 15 to 19 | 2.68 (2.01,3.35) | -0.91 (-2.1,0.3) |
| Middle SDI | Peru | 20 to 24 | 2.97 (2.42,3.53) | -0.73 (-1.8,0.36) |
| Middle SDI | Peru | 25 to 29 | 3.18 (2.71,3.65) | -0.59 (-1.58,0.41) |
| Middle SDI | Peru | 30 to 34 | 3.29 (2.85,3.72) | -0.56 (-1.47,0.37) |
| Middle SDI | Peru | 35 to 39 | 3.33 (2.92,3.74) | -0.5 (-1.35,0.36) |
| Middle SDI | Peru | 40 to 44 | 3.43 (3.03,3.83) | -0.38 (-1.17,0.41) |
| Middle SDI | Peru | 45 to 49 | 3.42 (3.05,3.8) | -0.3 (-1.02,0.43) |
| Middle SDI | Peru | 50 to 54 | 3.53 (3.18,3.89) | -0.19 (-0.85,0.47) |
| Middle SDI | Peru | 55 to 59 | 3.68 (3.34,4.02) | -0.03 (-0.63,0.58) |
| Middle SDI | Peru | 60 to 64 | 3.85 (3.53,4.17) | 0.18 (-0.38,0.75) |
| Middle SDI | Peru | 65 to 69 | 3.84 (3.52,4.16) | 0.32 (-0.22,0.86) |
| Middle SDI | Peru | 70 to 74 | 3.87 (3.52,4.23) | 0.46 (-0.09,1.01) |
| Middle SDI | Peru | 75 to 79 | 3.83 (3.38,4.29) | 0.62 (0,1.24) |
| Middle SDI | Peru | 80 to 84 | 3.9 (3.24,4.57) | 0.91 (0.1,1.73) |
| Middle SDI | Peru | 85 to 89 | 4.25 (3.07,5.45) | 1.39 (0.02,2.78) |
| Middle SDI | Philippines | 5 to 9 | -0.24 (-1.1,0.62) | -1 (-2.14,0.15) |
| Middle SDI | Philippines | 10 to 14 | 0.18 (-0.5,0.87) | -0.51 (-1.39,0.38) |
| Middle SDI | Philippines | 15 to 19 | 0.43 (-0.19,1.05) | -0.17 (-0.94,0.6) |
| Middle SDI | Philippines | 20 to 24 | 0.47 (-0.09,1.04) | -0.08 (-0.77,0.62) |
| Middle SDI | Philippines | 25 to 29 | 0.4 (-0.12,0.93) | -0.1 (-0.74,0.54) |
| Middle SDI | Philippines | 30 to 34 | 0.29 (-0.21,0.8) | -0.16 (-0.78,0.46) |
| Middle SDI | Philippines | 35 to 39 | 0.16 (-0.33,0.65) | -0.22 (-0.81,0.37) |
| Middle SDI | Philippines | 40 to 44 | 0.17 (-0.3,0.65) | -0.17 (-0.72,0.39) |
| Middle SDI | Philippines | 45 to 49 | 0.3 (-0.16,0.76) | 0 (-0.52,0.53) |
| Middle SDI | Philippines | 50 to 54 | 0.47 (0.02,0.92) | 0.21 (-0.29,0.71) |
| Middle SDI | Philippines | 55 to 59 | 0.7 (0.26,1.14) | 0.47 (-0.01,0.95) |
| Middle SDI | Philippines | 60 to 64 | 0.88 (0.43,1.33) | 0.65 (0.18,1.13) |
| Middle SDI | Philippines | 65 to 69 | 1.09 (0.59,1.58) | 0.88 (0.38,1.38) |
| Middle SDI | Philippines | 70 to 74 | 1.12 (0.54,1.7) | 0.93 (0.37,1.5) |
| Middle SDI | Philippines | 75 to 79 | 1.05 (0.31,1.79) | 0.84 (0.14,1.54) |
| Middle SDI | Philippines | 80 to 84 | 0.66 (-0.35,1.68) | 0.49 (-0.43,1.42) |
| Middle SDI | Philippines | 85 to 89 | 0.41 (-1.27,2.13) | 0.18 (-1.3,1.68) |
| Middle SDI | Saint Lucia | 5 to 9 | -6 (-23.82,15.97) | 2.82 (-19.03,30.57) |
| Middle SDI | Saint Lucia | 10 to 14 | -2.72 (-17.89,15.24) | 1.92 (-15.69,23.2) |
| Middle SDI | Saint Lucia | 15 to 19 | -0.98 (-13.97,13.97) | 0.01 (-15.7,18.65) |
| Middle SDI | Saint Lucia | 20 to 24 | 0.76 (-8.77,11.28) | -0.63 (-15.65,17.06) |
| Middle SDI | Saint Lucia | 25 to 29 | -0.69 (-7.32,6.42) | 0.47 (-14.14,17.57) |
| Middle SDI | Saint Lucia | 30 to 34 | -1.16 (-6.77,4.77) | 1.82 (-11.91,17.69) |
| Middle SDI | Saint Lucia | 35 to 39 | -1.66 (-6.81,3.77) | 3.39 (-9.13,17.62) |
| Middle SDI | Saint Lucia | 40 to 44 | -2.15 (-7.07,3.03) | 4.7 (-6.23,16.9) |
| Middle SDI | Saint Lucia | 45 to 49 | -2.4 (-7.12,2.55) | 3.12 (-6.51,13.73) |
| Middle SDI | Saint Lucia | 50 to 54 | -1.94 (-6.48,2.83) | 1.15 (-7.68,10.81) |
| Middle SDI | Saint Lucia | 55 to 59 | -1.6 (-6.13,3.14) | -0.52 (-7.96,7.51) |
| Middle SDI | Saint Lucia | 60 to 64 | -1.43 (-5.97,3.33) | -2.16 (-8.34,4.43) |
| Middle SDI | Saint Lucia | 65 to 69 | -1.45 (-6.23,3.57) | -2.54 (-8.18,3.43) |
| Middle SDI | Saint Lucia | 70 to 74 | -1.52 (-6.54,3.78) | -2.76 (-8.1,2.89) |
| Middle SDI | Saint Lucia | 75 to 79 | -0.95 (-7.46,6.01) | -2.33 (-8.98,4.8) |
| Middle SDI | Saint Lucia | 80 to 84 | 3.41 (-9.71,18.43) | 0.26 (-13.11,15.68) |
| Middle SDI | Saint Lucia | 85 to 89 | 4.11 (-15.11,27.68) | -1.8 (-21.67,23.11) |
| Middle SDI | Saint Vincent and the Grenadines | 5 to 9 | 1.59 (-19.81,28.7) | 1.95 (-19.71,29.47) |
| Middle SDI | Saint Vincent and the Grenadines | 10 to 14 | 1.47 (-15.46,21.78) | 1.52 (-16.02,22.73) |
| Middle SDI | Saint Vincent and the Grenadines | 15 to 19 | 1.08 (-13.15,17.65) | 1.18 (-14.94,20.36) |
| Middle SDI | Saint Vincent and the Grenadines | 20 to 24 | 0.78 (-10.51,13.49) | 0.46 (-14.82,18.48) |
| Middle SDI | Saint Vincent and the Grenadines | 25 to 29 | 0.55 (-6.32,7.93) | 0.27 (-14.69,17.84) |
| Middle SDI | Saint Vincent and the Grenadines | 30 to 34 | 0.28 (-5.45,6.36) | 0.44 (-13.89,17.15) |
| Middle SDI | Saint Vincent and the Grenadines | 35 to 39 | -0.37 (-5.69,5.26) | 1.43 (-12.01,16.93) |
| Middle SDI | Saint Vincent and the Grenadines | 40 to 44 | -1.19 (-6.67,4.62) | 2.74 (-9.47,16.59) |
| Middle SDI | Saint Vincent and the Grenadines | 45 to 49 | -2.37 (-7.68,3.23) | 3.99 (-6.66,15.86) |
| Middle SDI | Saint Vincent and the Grenadines | 50 to 54 | -3.02 (-8.5,2.79) | 3 (-6.49,13.46) |
| Middle SDI | Saint Vincent and the Grenadines | 55 to 59 | -3.16 (-8.43,2.41) | 1.75 (-6.62,10.86) |
| Middle SDI | Saint Vincent and the Grenadines | 60 to 64 | -2.78 (-7.92,2.66) | -0.25 (-6.99,6.98) |
| Middle SDI | Saint Vincent and the Grenadines | 65 to 69 | -2.3 (-7.45,3.14) | -1.46 (-7.25,4.7) |
| Middle SDI | Saint Vincent and the Grenadines | 70 to 74 | -1.45 (-6.99,4.43) | -1.73 (-7.47,4.36) |
| Middle SDI | Saint Vincent and the Grenadines | 75 to 79 | -0.98 (-7.79,6.32) | -0.96 (-7.89,6.5) |
| Middle SDI | Saint Vincent and the Grenadines | 80 to 84 | 1.86 (-11.65,17.44) | 1.54 (-12.03,17.2) |
| Middle SDI | Saint Vincent and the Grenadines | 85 to 89 | 0.42 (-19.81,25.74) | 0.65 (-19.72,26.19) |
| Middle SDI | South Africa | 5 to 9 | 1.65 (0.19,3.13) | 0.25 (-1.54,2.07) |
| Middle SDI | South Africa | 10 to 14 | 1.13 (-0.04,2.33) | -0.2 (-1.59,1.22) |
| Middle SDI | South Africa | 15 to 19 | 0.34 (-0.67,1.37) | -0.8 (-1.94,0.36) |
| Middle SDI | South Africa | 20 to 24 | 0.02 (-0.78,0.83) | -1.12 (-2.02,-0.21) |
| Middle SDI | South Africa | 25 to 29 | 0.06 (-0.58,0.7) | -1.02 (-1.73,-0.3) |
| Middle SDI | South Africa | 30 to 34 | 0.76 (0.23,1.3) | -0.2 (-0.8,0.4) |
| Middle SDI | South Africa | 35 to 39 | 1.78 (1.27,2.29) | 0.95 (0.4,1.5) |
| Middle SDI | South Africa | 40 to 44 | 2.66 (2.15,3.17) | 1.89 (1.36,2.43) |
| Middle SDI | South Africa | 45 to 49 | 2.77 (2.24,3.31) | 2.08 (1.54,2.62) |
| Middle SDI | South Africa | 50 to 54 | 2.2 (1.65,2.76) | 1.57 (1.03,2.11) |
| Middle SDI | South Africa | 55 to 59 | 1.79 (1.22,2.36) | 1.22 (0.68,1.76) |
| Middle SDI | South Africa | 60 to 64 | 1.6 (0.99,2.2) | 1.1 (0.55,1.65) |
| Middle SDI | South Africa | 65 to 69 | 1.52 (0.87,2.17) | 1.12 (0.55,1.69) |
| Middle SDI | South Africa | 70 to 74 | 1.43 (0.71,2.15) | 1.08 (0.48,1.69) |
| Middle SDI | South Africa | 75 to 79 | 1.39 (0.54,2.25) | 1.11 (0.43,1.81) |
| Middle SDI | South Africa | 80 to 84 | 1.22 (0.12,2.33) | 0.99 (0.13,1.86) |
| Middle SDI | South Africa | 85 to 89 | 1.31 (-0.39,3.04) | 1.05 (-0.25,2.36) |
| Middle SDI | Sri Lanka | 5 to 9 | -0.24 (-2.72,2.31) | -3.35 (-7.73,1.25) |
| Middle SDI | Sri Lanka | 10 to 14 | 0.43 (-1.33,2.22) | -2.47 (-5.28,0.42) |
| Middle SDI | Sri Lanka | 15 to 19 | 0.91 (-0.49,2.33) | -2 (-4.08,0.12) |
| Middle SDI | Sri Lanka | 20 to 24 | 0.9 (-0.3,2.12) | -1.79 (-3.5,-0.05) |
| Middle SDI | Sri Lanka | 25 to 29 | 0.8 (-0.29,1.9) | -1.72 (-3.26,-0.14) |
| Middle SDI | Sri Lanka | 30 to 34 | 0.72 (-0.3,1.75) | -1.57 (-3.01,-0.12) |
| Middle SDI | Sri Lanka | 35 to 39 | 0.76 (-0.19,1.73) | -1.33 (-2.64,0) |
| Middle SDI | Sri Lanka | 40 to 44 | 0.86 (-0.04,1.78) | -0.98 (-2.17,0.22) |
| Middle SDI | Sri Lanka | 45 to 49 | 1.06 (0.23,1.9) | -0.61 (-1.66,0.45) |
| Middle SDI | Sri Lanka | 50 to 54 | 1.13 (0.36,1.91) | -0.4 (-1.33,0.54) |
| Middle SDI | Sri Lanka | 55 to 59 | 1.15 (0.42,1.88) | -0.24 (-1.08,0.62) |
| Middle SDI | Sri Lanka | 60 to 64 | 1.08 (0.37,1.79) | -0.19 (-0.98,0.61) |
| Middle SDI | Sri Lanka | 65 to 69 | 0.99 (0.26,1.73) | -0.17 (-0.95,0.62) |
| Middle SDI | Sri Lanka | 70 to 74 | 0.99 (0.16,1.83) | -0.01 (-0.86,0.85) |
| Middle SDI | Sri Lanka | 75 to 79 | 1.18 (0.08,2.29) | 0.38 (-0.69,1.47) |
| Middle SDI | Sri Lanka | 80 to 84 | 1.3 (-0.25,2.87) | 0.57 (-0.87,2.03) |
| Middle SDI | Sri Lanka | 85 to 89 | 1.11 (-1.51,3.8) | 0.22 (-2.09,2.6) |
| Middle SDI | Suriname | 5 to 9 | -1.06 (-9.06,7.64) | -0.55 (-21.37,25.77) |
| Middle SDI | Suriname | 10 to 14 | -0.6 (-8.34,7.8) | -0.21 (-16.19,18.82) |
| Middle SDI | Suriname | 15 to 19 | -0.3 (-6.65,6.48) | -0.02 (-12.47,14.21) |
| Middle SDI | Suriname | 20 to 24 | 0.58 (-4.97,6.45) | 0.11 (-7.54,8.38) |
| Middle SDI | Suriname | 25 to 29 | 1.41 (-3.58,6.66) | -0.75 (-7.42,6.4) |
| Middle SDI | Suriname | 30 to 34 | 0.55 (-4.09,5.41) | -1.14 (-6.46,4.47) |
| Middle SDI | Suriname | 35 to 39 | 0.09 (-4.43,4.83) | -1.66 (-6.81,3.76) |
| Middle SDI | Suriname | 40 to 44 | -0.05 (-4.42,4.51) | -1.62 (-6.53,3.54) |
| Middle SDI | Suriname | 45 to 49 | -0.25 (-4.44,4.11) | -1.22 (-5.92,3.72) |
| Middle SDI | Suriname | 50 to 54 | 0.5 (-3.48,4.64) | -0.8 (-5.21,3.81) |
| Middle SDI | Suriname | 55 to 59 | 0.88 (-2.91,4.82) | -0.48 (-4.76,3.98) |
| Middle SDI | Suriname | 60 to 64 | 0.19 (-3.43,3.94) | -0.47 (-4.86,4.13) |
| Middle SDI | Suriname | 65 to 69 | 0.75 (-3.42,5.09) | 0.2 (-4.41,5.02) |
| Middle SDI | Suriname | 70 to 74 | 0.28 (-4.34,5.12) | -0.11 (-4.93,4.96) |
| Middle SDI | Suriname | 75 to 79 | 0.59 (-5.56,7.13) | 0.43 (-5.76,7.03) |
| Middle SDI | Suriname | 80 to 84 | 3.86 (-9.23,18.83) | 3.81 (-9.28,18.8) |
| Middle SDI | Suriname | 85 to 89 | 4.87 (-14.47,28.59) | 5.22 (-14.2,29.04) |
| Middle SDI | Thailand | 5 to 9 | -1.19 (-2.33,-0.02) | -3.92 (-6.07,-1.73) |
| Middle SDI | Thailand | 10 to 14 | -0.03 (-0.84,0.8) | -2.66 (-4.08,-1.22) |
| Middle SDI | Thailand | 15 to 19 | 0.65 (0,1.3) | -1.87 (-2.89,-0.84) |
| Middle SDI | Thailand | 20 to 24 | 1.02 (0.48,1.56) | -1.36 (-2.17,-0.54) |
| Middle SDI | Thailand | 25 to 29 | 1.27 (0.81,1.74) | -1.01 (-1.71,-0.3) |
| Middle SDI | Thailand | 30 to 34 | 1.38 (0.96,1.8) | -0.75 (-1.37,-0.13) |
| Middle SDI | Thailand | 35 to 39 | 1.41 (1.03,1.8) | -0.56 (-1.11,-0.01) |
| Middle SDI | Thailand | 40 to 44 | 1.25 (0.89,1.62) | -0.52 (-1.01,-0.03) |
| Middle SDI | Thailand | 45 to 49 | 0.96 (0.62,1.31) | -0.66 (-1.1,-0.2) |
| Middle SDI | Thailand | 50 to 54 | 0.51 (0.18,0.85) | -0.95 (-1.36,-0.54) |
| Middle SDI | Thailand | 55 to 59 | 0.19 (-0.13,0.51) | -1.16 (-1.54,-0.78) |
| Middle SDI | Thailand | 60 to 64 | 0.12 (-0.2,0.44) | -1.14 (-1.5,-0.77) |
| Middle SDI | Thailand | 65 to 69 | 0.18 (-0.16,0.52) | -0.97 (-1.35,-0.6) |
| Middle SDI | Thailand | 70 to 74 | 0.07 (-0.33,0.46) | -0.96 (-1.37,-0.54) |
| Middle SDI | Thailand | 75 to 79 | -0.22 (-0.73,0.3) | -1.09 (-1.59,-0.58) |
| Middle SDI | Thailand | 80 to 84 | -0.53 (-1.26,0.2) | -1.23 (-1.91,-0.54) |
| Middle SDI | Thailand | 85 to 89 | -0.73 (-1.97,0.53) | -1.44 (-2.56,-0.31) |
| Middle SDI | Tunisia | 5 to 9 | 0.36 (-1.17,1.92) | -2.59 (-5.89,0.82) |
| Middle SDI | Tunisia | 10 to 14 | 1.05 (-0.26,2.37) | -1.57 (-4.08,1.02) |
| Middle SDI | Tunisia | 15 to 19 | 1.53 (0.27,2.8) | -0.95 (-3.24,1.38) |
| Middle SDI | Tunisia | 20 to 24 | 1.95 (0.77,3.16) | -0.45 (-2.51,1.66) |
| Middle SDI | Tunisia | 25 to 29 | 2.24 (1.16,3.34) | -0.17 (-2.01,1.7) |
| Middle SDI | Tunisia | 30 to 34 | 2.39 (1.39,3.4) | 0.11 (-1.55,1.8) |
| Middle SDI | Tunisia | 35 to 39 | 2.25 (1.29,3.21) | -0.03 (-1.56,1.52) |
| Middle SDI | Tunisia | 40 to 44 | 2.05 (1.12,2.99) | -0.13 (-1.56,1.32) |
| Middle SDI | Tunisia | 45 to 49 | 1.87 (0.97,2.77) | -0.07 (-1.39,1.26) |
| Middle SDI | Tunisia | 50 to 54 | 1.69 (0.85,2.55) | -0.07 (-1.25,1.12) |
| Middle SDI | Tunisia | 55 to 59 | 1.44 (0.67,2.22) | -0.19 (-1.22,0.84) |
| Middle SDI | Tunisia | 60 to 64 | 1.22 (0.5,1.94) | -0.24 (-1.14,0.68) |
| Middle SDI | Tunisia | 65 to 69 | 1.01 (0.3,1.73) | -0.27 (-1.13,0.59) |
| Middle SDI | Tunisia | 70 to 74 | 0.85 (0.07,1.62) | -0.28 (-1.15,0.59) |
| Middle SDI | Tunisia | 75 to 79 | 0.7 (-0.22,1.63) | -0.24 (-1.19,0.73) |
| Middle SDI | Tunisia | 80 to 84 | 0.56 (-0.7,1.83) | -0.15 (-1.39,1.11) |
| Middle SDI | Tunisia | 85 to 89 | 0.19 (-1.93,2.37) | -0.44 (-2.48,1.65) |
| Middle SDI | Türkiye | 5 to 9 | 0.13 (-0.58,0.85) | -3.89 (-5.33,-2.42) |
| Middle SDI | Türkiye | 10 to 14 | 1.14 (0.61,1.67) | -2.75 (-3.72,-1.76) |
| Middle SDI | Türkiye | 15 to 19 | 1.45 (1,1.9) | -2.28 (-3.04,-1.51) |
| Middle SDI | Türkiye | 20 to 24 | 1.39 (0.98,1.8) | -2.21 (-2.88,-1.53) |
| Middle SDI | Türkiye | 25 to 29 | 1.27 (0.88,1.65) | -2.23 (-2.85,-1.6) |
| Middle SDI | Türkiye | 30 to 34 | 1.24 (0.88,1.61) | -2.16 (-2.74,-1.58) |
| Middle SDI | Türkiye | 35 to 39 | 1.13 (0.79,1.48) | -2.12 (-2.65,-1.59) |
| Middle SDI | Türkiye | 40 to 44 | 1.02 (0.69,1.35) | -2.04 (-2.53,-1.55) |
| Middle SDI | Türkiye | 45 to 49 | 0.97 (0.65,1.28) | -1.85 (-2.29,-1.4) |
| Middle SDI | Türkiye | 50 to 54 | 0.89 (0.58,1.19) | -1.72 (-2.13,-1.32) |
| Middle SDI | Türkiye | 55 to 59 | 0.83 (0.55,1.12) | -1.58 (-1.94,-1.21) |
| Middle SDI | Türkiye | 60 to 64 | 0.76 (0.5,1.03) | -1.44 (-1.77,-1.11) |
| Middle SDI | Türkiye | 65 to 69 | 0.77 (0.49,1.04) | -1.18 (-1.5,-0.86) |
| Middle SDI | Türkiye | 70 to 74 | 0.98 (0.66,1.3) | -0.69 (-1.04,-0.35) |
| Middle SDI | Türkiye | 75 to 79 | 1.34 (0.91,1.77) | -0.05 (-0.48,0.38) |
| Middle SDI | Türkiye | 80 to 84 | 1.57 (0.97,2.17) | 0.46 (-0.12,1.03) |
| Middle SDI | Türkiye | 85 to 89 | 1.55 (0.55,2.55) | 0.52 (-0.4,1.45) |
| Middle SDI | Turkmenistan | 5 to 9 | 0.03 (-4.3,4.57) | -0.87 (-7.91,6.72) |
| Middle SDI | Turkmenistan | 10 to 14 | 0.18 (-3.52,4.01) | 0.09 (-5.56,6.08) |
| Middle SDI | Turkmenistan | 15 to 19 | 0.68 (-2.78,4.26) | 1.17 (-3.88,6.5) |
| Middle SDI | Turkmenistan | 20 to 24 | 1.59 (-1.7,5) | 2.57 (-2.02,7.37) |
| Middle SDI | Turkmenistan | 25 to 29 | 2.19 (-1.01,5.49) | 2.29 (-2.2,6.98) |
| Middle SDI | Turkmenistan | 30 to 34 | 2.28 (-0.9,5.57) | 1.71 (-2.71,6.34) |
| Middle SDI | Turkmenistan | 35 to 39 | 2.09 (-1.07,5.35) | 1.51 (-2.85,6.07) |
| Middle SDI | Turkmenistan | 40 to 44 | 2.18 (-1.11,5.57) | 0.95 (-3.3,5.4) |
| Middle SDI | Turkmenistan | 45 to 49 | 2.24 (-1.23,5.82) | 0.39 (-3.76,4.72) |
| Middle SDI | Turkmenistan | 50 to 54 | 2.12 (-1.34,5.7) | 0.4 (-3.55,4.52) |
| Middle SDI | Turkmenistan | 55 to 59 | 1.91 (-1.41,5.34) | 0.03 (-3.6,3.78) |
| Middle SDI | Turkmenistan | 60 to 64 | 1.56 (-1.82,5.06) | 0.24 (-3.39,4.01) |
| Middle SDI | Turkmenistan | 65 to 69 | 1.2 (-2.74,5.29) | 0.65 (-3.39,4.86) |
| Middle SDI | Turkmenistan | 70 to 74 | 0.64 (-4.42,5.96) | 0.51 (-4.57,5.85) |
| Middle SDI | Turkmenistan | 75 to 79 | -0.23 (-6.82,6.83) | -0.15 (-6.78,6.96) |
| Middle SDI | Turkmenistan | 80 to 84 | 1.58 (-11.84,17.05) | 1.77 (-11.69,17.28) |
| Middle SDI | Turkmenistan | 85 to 89 | 0.33 (-19.81,25.53) | 0.44 (-19.74,25.7) |
| Middle SDI | Uruguay | 5 to 9 | -1.9 (-6.37,2.77) | 0.97 (-6.21,8.71) |
| Middle SDI | Uruguay | 10 to 14 | 0.01 (-2.91,3.02) | 0.88 (-4.45,6.51) |
| Middle SDI | Uruguay | 15 to 19 | 1.18 (-0.98,3.39) | 0.45 (-3.59,4.66) |
| Middle SDI | Uruguay | 20 to 24 | 1.6 (-0.22,3.45) | -0.03 (-3.33,3.38) |
| Middle SDI | Uruguay | 25 to 29 | 1.72 (0.13,3.33) | -0.13 (-3.04,2.85) |
| Middle SDI | Uruguay | 30 to 34 | 1.44 (0.02,2.88) | -0.32 (-2.88,2.3) |
| Middle SDI | Uruguay | 35 to 39 | 1.14 (-0.16,2.45) | -0.7 (-2.97,1.62) |
| Middle SDI | Uruguay | 40 to 44 | 0.75 (-0.44,1.94) | -0.8 (-2.76,1.21) |
| Middle SDI | Uruguay | 45 to 49 | 0.46 (-0.62,1.56) | -0.83 (-2.52,0.9) |
| Middle SDI | Uruguay | 50 to 54 | 0.26 (-0.73,1.26) | -0.97 (-2.43,0.52) |
| Middle SDI | Uruguay | 55 to 59 | 0.43 (-0.46,1.32) | -0.74 (-1.98,0.52) |
| Middle SDI | Uruguay | 60 to 64 | 0.46 (-0.35,1.28) | -0.58 (-1.65,0.51) |
| Middle SDI | Uruguay | 65 to 69 | 0.52 (-0.26,1.31) | -0.45 (-1.43,0.54) |
| Middle SDI | Uruguay | 70 to 74 | 0.65 (-0.15,1.46) | -0.23 (-1.17,0.71) |
| Middle SDI | Uruguay | 75 to 79 | 0.93 (0,1.86) | 0.15 (-0.84,1.15) |
| Middle SDI | Uruguay | 80 to 84 | 1.12 (-0.08,2.33) | 0.45 (-0.75,1.67) |
| Middle SDI | Uruguay | 85 to 89 | 1.56 (-0.42,3.59) | 1.01 (-0.92,2.98) |
| Middle SDI | Uzbekistan | 5 to 9 | 1.24 (0.12,2.37) | -0.29 (-2.08,1.53) |
| Middle SDI | Uzbekistan | 10 to 14 | 1 (0.01,1.99) | -0.4 (-1.9,1.11) |
| Middle SDI | Uzbekistan | 15 to 19 | 0.31 (-0.65,1.27) | -0.97 (-2.33,0.42) |
| Middle SDI | Uzbekistan | 20 to 24 | -0.1 (-1.01,0.81) | -1.25 (-2.51,0.02) |
| Middle SDI | Uzbekistan | 25 to 29 | -0.23 (-1.09,0.63) | -1.39 (-2.57,-0.19) |
| Middle SDI | Uzbekistan | 30 to 34 | -0.33 (-1.16,0.51) | -1.41 (-2.54,-0.26) |
| Middle SDI | Uzbekistan | 35 to 39 | -0.32 (-1.15,0.52) | -1.31 (-2.42,-0.18) |
| Middle SDI | Uzbekistan | 40 to 44 | -0.48 (-1.32,0.37) | -1.41 (-2.5,-0.3) |
| Middle SDI | Uzbekistan | 45 to 49 | -0.8 (-1.66,0.06) | -1.64 (-2.72,-0.54) |
| Middle SDI | Uzbekistan | 50 to 54 | -0.72 (-1.57,0.13) | -1.48 (-2.51,-0.43) |
| Middle SDI | Uzbekistan | 55 to 59 | -0.57 (-1.4,0.26) | -1.22 (-2.2,-0.23) |
| Middle SDI | Uzbekistan | 60 to 64 | -0.31 (-1.17,0.56) | -0.88 (-1.88,0.12) |
| Middle SDI | Uzbekistan | 65 to 69 | 0.04 (-0.95,1.04) | -0.43 (-1.53,0.67) |
| Middle SDI | Uzbekistan | 70 to 74 | 0.4 (-0.9,1.71) | 0.07 (-1.31,1.46) |
| Middle SDI | Uzbekistan | 75 to 79 | 0.83 (-1.01,2.72) | 0.47 (-1.36,2.33) |
| Middle SDI | Uzbekistan | 80 to 84 | 1.22 (-1.4,3.9) | 0.73 (-1.76,3.28) |
| Middle SDI | Uzbekistan | 85 to 89 | 1.46 (-3.19,6.34) | 1.1 (-3.51,5.92) |

**Table S4 Age effects on non-Hodgkin lymphoma incidence and mortality across 204 countries and territories**

| **Quintile** | **Country** | **Age** | **Incidence rate (per 100,000 population)** | **Mortality rate (per 100,000 population)** |
| --- | --- | --- | --- | --- |
| High SDI | Andorra | 5 to 9 | 2.24 (0.06,80.38) | 3.32 (0.02,551.04) |
| High SDI | Andorra | 10 to 14 | 2.33 (0.09,58.13) | 3.31 (0.03,403.19) |
| High SDI | Andorra | 15 to 19 | 2.32 (0.12,45.74) | 3.28 (0.03,308.8) |
| High SDI | Andorra | 20 to 24 | 2 (0.12,33.63) | 2.86 (0.04,209.62) |
| High SDI | Andorra | 25 to 29 | 15.73 (4.42,55.99) | 2.26 (0.04,129.02) |
| High SDI | Andorra | 30 to 34 | 16.65 (5.33,51.99) | 1.94 (0.05,79.29) |
| High SDI | Andorra | 35 to 39 | 19.16 (6.9,53.23) | 1.88 (0.06,56.49) |
| High SDI | Andorra | 40 to 44 | 26.54 (11.1,63.47) | 1.89 (0.09,41.62) |
| High SDI | Andorra | 45 to 49 | 37.57 (17.38,81.22) | 1.95 (0.11,35.57) |
| High SDI | Andorra | 50 to 54 | 47.2 (23.51,94.75) | 2.08 (0.13,33.81) |
| High SDI | Andorra | 55 to 59 | 58.5 (29.53,115.9) | 18.98 (4.62,77.9) |
| High SDI | Andorra | 60 to 64 | 79.22 (39.78,157.78) | 25.33 (6.83,93.98) |
| High SDI | Andorra | 65 to 69 | 95.01 (45.67,197.66) | 28.74 (7.37,112.09) |
| High SDI | Andorra | 70 to 74 | 136.55 (59.77,312) | 32.56 (6.82,155.33) |
| High SDI | Andorra | 75 to 79 | 132.39 (52.44,334.2) | 38.14 (7.48,194.57) |
| High SDI | Andorra | 80 to 84 | 158.03 (57.37,435.28) | 46.7 (8.69,250.9) |
| High SDI | Andorra | 85 to 89 | 224.08 (75,669.45) | 65.72 (11.38,379.69) |
| High SDI | Australia | 5 to 9 | 2.66 (2.11,3.36) | 0.95 (0.48,1.85) |
| High SDI | Australia | 10 to 14 | 2.41 (1.95,2.97) | 0.82 (0.45,1.49) |
| High SDI | Australia | 15 to 19 | 3.31 (2.77,3.95) | 1.15 (0.72,1.86) |
| High SDI | Australia | 20 to 24 | 3.54 (3.01,4.15) | 1.21 (0.8,1.81) |
| High SDI | Australia | 25 to 29 | 5.86 (5.16,6.66) | 1.38 (0.97,1.94) |
| High SDI | Australia | 30 to 34 | 7.48 (6.7,8.34) | 1.59 (1.19,2.12) |
| High SDI | Australia | 35 to 39 | 9.9 (9.02,10.87) | 2 (1.58,2.53) |
| High SDI | Australia | 40 to 44 | 12.92 (11.95,13.97) | 2.58 (2.14,3.11) |
| High SDI | Australia | 45 to 49 | 19.03 (17.83,20.32) | 3.5 (3,4.09) |
| High SDI | Australia | 50 to 54 | 26.26 (24.78,27.82) | 5.09 (4.46,5.81) |
| High SDI | Australia | 55 to 59 | 36.14 (34.3,38.09) | 7.26 (6.46,8.15) |
| High SDI | Australia | 60 to 64 | 51.08 (48.64,53.65) | 10.02 (9.01,11.16) |
| High SDI | Australia | 65 to 69 | 65.2 (62.05,68.51) | 14.01 (12.65,15.52) |
| High SDI | Australia | 70 to 74 | 85.94 (81.16,91) | 19.62 (17.5,22) |
| High SDI | Australia | 75 to 79 | 106.82 (100.53,113.5) | 27.42 (24.42,30.8) |
| High SDI | Australia | 80 to 84 | 126.59 (118.57,135.15) | 38.36 (34.07,43.2) |
| High SDI | Australia | 85 to 89 | 152 (141.3,163.51) | 47.26 (41.68,53.58) |
| High SDI | Austria | 5 to 9 | 2.29 (1.59,3.3) | 0.49 (0.15,1.56) |
| High SDI | Austria | 10 to 14 | 2.15 (1.53,3.01) | 0.47 (0.16,1.34) |
| High SDI | Austria | 15 to 19 | 4.16 (3.2,5.41) | 0.81 (0.36,1.8) |
| High SDI | Austria | 20 to 24 | 4.49 (3.56,5.66) | 0.99 (0.51,1.92) |
| High SDI | Austria | 25 to 29 | 6.56 (5.41,7.96) | 1.08 (0.62,1.89) |
| High SDI | Austria | 30 to 34 | 7.45 (6.27,8.85) | 1.09 (0.67,1.78) |
| High SDI | Austria | 35 to 39 | 8.43 (7.22,9.85) | 1.43 (0.95,2.14) |
| High SDI | Austria | 40 to 44 | 11.07 (9.7,12.64) | 2.05 (1.49,2.82) |
| High SDI | Austria | 45 to 49 | 14.29 (12.72,16.05) | 2.92 (2.25,3.8) |
| High SDI | Austria | 50 to 54 | 19.11 (17.23,21.21) | 4.25 (3.41,5.29) |
| High SDI | Austria | 55 to 59 | 24.67 (22.38,27.2) | 6.26 (5.16,7.61) |
| High SDI | Austria | 60 to 64 | 38.68 (35.34,42.34) | 9.61 (8.04,11.48) |
| High SDI | Austria | 65 to 69 | 50.37 (45.92,55.25) | 14.26 (12.01,16.95) |
| High SDI | Austria | 70 to 74 | 67.16 (60.46,74.62) | 21.58 (17.84,26.1) |
| High SDI | Austria | 75 to 79 | 84.53 (75.63,94.46) | 32.72 (26.99,39.67) |
| High SDI | Austria | 80 to 84 | 99.28 (87.9,112.13) | 45.44 (37.23,55.46) |
| High SDI | Austria | 85 to 89 | 123.74 (107.97,141.81) | 59.26 (48,73.16) |
| High SDI | Belgium | 5 to 9 | 3.17 (2.35,4.27) | 0.7 (0.27,1.81) |
| High SDI | Belgium | 10 to 14 | 3.05 (2.32,4) | 0.6 (0.25,1.44) |
| High SDI | Belgium | 15 to 19 | 3.82 (3.02,4.84) | 0.89 (0.44,1.79) |
| High SDI | Belgium | 20 to 24 | 4.54 (3.7,5.59) | 1.01 (0.56,1.81) |
| High SDI | Belgium | 25 to 29 | 7.05 (5.95,8.35) | 1.14 (0.7,1.87) |
| High SDI | Belgium | 30 to 34 | 8.67 (7.48,10.06) | 1.31 (0.87,1.98) |
| High SDI | Belgium | 35 to 39 | 9.69 (8.48,11.07) | 1.77 (1.26,2.48) |
| High SDI | Belgium | 40 to 44 | 12.48 (11.16,13.96) | 2.39 (1.83,3.12) |
| High SDI | Belgium | 45 to 49 | 16.79 (15.25,18.49) | 3.23 (2.59,4.03) |
| High SDI | Belgium | 50 to 54 | 21.22 (19.45,23.15) | 4.57 (3.79,5.5) |
| High SDI | Belgium | 55 to 59 | 28.07 (25.92,30.4) | 6.64 (5.64,7.81) |
| High SDI | Belgium | 60 to 64 | 41.75 (38.77,44.96) | 9.53 (8.2,11.08) |
| High SDI | Belgium | 65 to 69 | 52.41 (48.6,56.51) | 13.92 (12.02,16.11) |
| High SDI | Belgium | 70 to 74 | 73.29 (67.36,79.74) | 20.52 (17.5,24.07) |
| High SDI | Belgium | 75 to 79 | 83.31 (76.19,91.1) | 28.52 (24.26,33.53) |
| High SDI | Belgium | 80 to 84 | 96.24 (87.47,105.89) | 39.14 (33.17,46.19) |
| High SDI | Belgium | 85 to 89 | 122.9 (110.59,136.58) | 47.78 (40.09,56.94) |
| High SDI | Bermuda | 5 to 9 | 2.55 (0.02,266.56) | 2.64 (0.01,643.5) |
| High SDI | Bermuda | 10 to 14 | 2.73 (0.04,204.88) | 2.65 (0.01,488.19) |
| High SDI | Bermuda | 15 to 19 | 3.04 (0.05,173.12) | 2.75 (0.02,400.77) |
| High SDI | Bermuda | 20 to 24 | 3.05 (0.08,122.47) | 2.75 (0.02,318.02) |
| High SDI | Bermuda | 25 to 29 | 2.58 (0.09,75.93) | 2.52 (0.03,228.97) |
| High SDI | Bermuda | 30 to 34 | 2.32 (0.1,52.53) | 2.31 (0.03,165.15) |
| High SDI | Bermuda | 35 to 39 | 2.22 (0.12,41.12) | 2.29 (0.04,129.1) |
| High SDI | Bermuda | 40 to 44 | 9.24 (1.16,73.69) | 2.31 (0.06,83.46) |
| High SDI | Bermuda | 45 to 49 | 22.1 (6.97,70.02) | 2.49 (0.09,72.07) |
| High SDI | Bermuda | 50 to 54 | 23.09 (7.77,68.64) | 2.55 (0.1,63.79) |
| High SDI | Bermuda | 55 to 59 | 26.98 (9.24,78.79) | 2.59 (0.11,60.49) |
| High SDI | Bermuda | 60 to 64 | 40.78 (15.74,105.67) | 3.48 (0.15,81.83) |
| High SDI | Bermuda | 65 to 69 | 48.7 (18.39,129.01) | 17.49 (2.58,118.47) |
| High SDI | Bermuda | 70 to 74 | 62.39 (19.67,197.88) | 27.47 (1.89,398.79) |
| High SDI | Bermuda | 75 to 79 | 82.94 (24.62,279.38) | 23.96 (1.49,384.86) |
| High SDI | Bermuda | 80 to 84 | 93.25 (23.15,375.71) | 23.43 (0.97,568.22) |
| High SDI | Bermuda | 85 to 89 | 106.46 (18.74,604.89) | 26.12 (0.54,1253.47) |
| High SDI | Canada | 5 to 9 | 3.65 (2.81,4.75) | 0.71 (0.4,1.27) |
| High SDI | Canada | 10 to 14 | 4.23 (3.39,5.28) | 0.69 (0.42,1.13) |
| High SDI | Canada | 15 to 19 | 8.21 (7.01,9.62) | 1 (0.68,1.48) |
| High SDI | Canada | 20 to 24 | 16.59 (14.73,18.69) | 1.3 (0.94,1.79) |
| High SDI | Canada | 25 to 29 | 23.06 (20.91,25.44) | 1.48 (1.13,1.93) |
| High SDI | Canada | 30 to 34 | 23.22 (21.31,25.29) | 1.71 (1.37,2.13) |
| High SDI | Canada | 35 to 39 | 18.16 (16.74,19.69) | 2.13 (1.78,2.54) |
| High SDI | Canada | 40 to 44 | 21.26 (19.87,22.75) | 2.82 (2.46,3.24) |
| High SDI | Canada | 45 to 49 | 27.76 (26.22,29.4) | 3.8 (3.39,4.26) |
| High SDI | Canada | 50 to 54 | 30.57 (28.97,32.25) | 5.68 (5.17,6.24) |
| High SDI | Canada | 55 to 59 | 45.55 (43.44,47.77) | 8.29 (7.63,9.01) |
| High SDI | Canada | 60 to 64 | 54.26 (51.79,56.84) | 11.68 (10.81,12.61) |
| High SDI | Canada | 65 to 69 | 73.8 (70.45,77.31) | 16.34 (15.17,17.6) |
| High SDI | Canada | 70 to 74 | 85.28 (80.79,90.02) | 23.47 (21.61,25.5) |
| High SDI | Canada | 75 to 79 | 92.88 (87.58,98.49) | 32.56 (29.91,35.44) |
| High SDI | Canada | 80 to 84 | 108.18 (101.44,115.35) | 45.53 (41.71,49.69) |
| High SDI | Canada | 85 to 89 | 129.46 (120.38,139.23) | 50.17 (45.64,55.15) |
| High SDI | Cyprus | 5 to 9 | 2.27 (1.04,4.98) | 0.35 (0.01,15.62) |
| High SDI | Cyprus | 10 to 14 | 1.35 (0.61,3.01) | 0.32 (0.01,9.41) |
| High SDI | Cyprus | 15 to 19 | 2.73 (1.45,5.11) | 0.28 (0.01,6.33) |
| High SDI | Cyprus | 20 to 24 | 2.5 (1.36,4.59) | 0.23 (0.01,4.19) |
| High SDI | Cyprus | 25 to 29 | 3.77 (2.23,6.4) | 1.35 (0.3,6) |
| High SDI | Cyprus | 30 to 34 | 5.55 (3.48,8.84) | 1.59 (0.48,5.22) |
| High SDI | Cyprus | 35 to 39 | 6.48 (4.22,9.93) | 1.44 (0.48,4.26) |
| High SDI | Cyprus | 40 to 44 | 8 (5.41,11.84) | 1.46 (0.54,3.9) |
| High SDI | Cyprus | 45 to 49 | 12.39 (8.83,17.41) | 2.52 (1.1,5.75) |
| High SDI | Cyprus | 50 to 54 | 17.85 (13.18,24.17) | 4.03 (2.05,7.93) |
| High SDI | Cyprus | 55 to 59 | 29.95 (23.1,38.84) | 6.45 (3.66,11.38) |
| High SDI | Cyprus | 60 to 64 | 51.51 (40.78,65.07) | 9.98 (5.98,16.65) |
| High SDI | Cyprus | 65 to 69 | 76.21 (60.19,96.49) | 14.86 (9.13,24.2) |
| High SDI | Cyprus | 70 to 74 | 112.82 (85.23,149.35) | 22.95 (13.16,40) |
| High SDI | Cyprus | 75 to 79 | 153.93 (113.88,208.07) | 31.61 (17.87,55.92) |
| High SDI | Cyprus | 80 to 84 | 184.08 (131.83,257.05) | 46.88 (26.01,84.49) |
| High SDI | Cyprus | 85 to 89 | 272.7 (186.1,399.59) | 62.21 (32.81,117.98) |
| High SDI | Czechia | 5 to 9 | 0.72 (0.48,1.06) | 0.38 (0.15,0.96) |
| High SDI | Czechia | 10 to 14 | 0.91 (0.65,1.27) | 0.38 (0.17,0.83) |
| High SDI | Czechia | 15 to 19 | 1.45 (1.1,1.91) | 0.61 (0.32,1.16) |
| High SDI | Czechia | 20 to 24 | 1.65 (1.28,2.13) | 0.73 (0.42,1.27) |
| High SDI | Czechia | 25 to 29 | 3.15 (2.56,3.88) | 0.84 (0.51,1.37) |
| High SDI | Czechia | 30 to 34 | 4.37 (3.64,5.24) | 0.92 (0.59,1.43) |
| High SDI | Czechia | 35 to 39 | 5.19 (4.4,6.12) | 1.35 (0.94,1.94) |
| High SDI | Czechia | 40 to 44 | 7.28 (6.36,8.33) | 1.86 (1.4,2.47) |
| High SDI | Czechia | 45 to 49 | 10.83 (9.67,12.12) | 2.75 (2.19,3.45) |
| High SDI | Czechia | 50 to 54 | 15.17 (13.74,16.75) | 4.14 (3.43,5.01) |
| High SDI | Czechia | 55 to 59 | 22.25 (20.36,24.31) | 6.46 (5.49,7.61) |
| High SDI | Czechia | 60 to 64 | 32.92 (30.32,35.73) | 9.54 (8.22,11.06) |
| High SDI | Czechia | 65 to 69 | 45.48 (41.91,49.34) | 14.66 (12.73,16.89) |
| High SDI | Czechia | 70 to 74 | 64.62 (58.76,71.08) | 21.1 (17.97,24.77) |
| High SDI | Czechia | 75 to 79 | 78.2 (70.5,86.74) | 28.46 (24.09,33.64) |
| High SDI | Czechia | 80 to 84 | 91.19 (81.21,102.4) | 36.37 (30.47,43.42) |
| High SDI | Czechia | 85 to 89 | 119.11 (104.11,136.28) | 39.86 (32.58,48.76) |
| High SDI | Denmark | 5 to 9 | 1.17 (0.68,2.02) | 0.84 (0.19,3.65) |
| High SDI | Denmark | 10 to 14 | 1.25 (0.76,2.05) | 0.84 (0.23,3.03) |
| High SDI | Denmark | 15 to 19 | 1.4 (0.89,2.21) | 0.88 (0.28,2.76) |
| High SDI | Denmark | 20 to 24 | 1.86 (1.25,2.75) | 0.73 (0.26,2.06) |
| High SDI | Denmark | 25 to 29 | 3.42 (2.53,4.62) | 0.93 (0.42,2.09) |
| High SDI | Denmark | 30 to 34 | 4.24 (3.26,5.5) | 1.15 (0.6,2.23) |
| High SDI | Denmark | 35 to 39 | 6.01 (4.84,7.46) | 1.64 (0.98,2.73) |
| High SDI | Denmark | 40 to 44 | 9.53 (8.04,11.3) | 2.03 (1.35,3.05) |
| High SDI | Denmark | 45 to 49 | 14.56 (12.67,16.73) | 3.15 (2.31,4.3) |
| High SDI | Denmark | 50 to 54 | 21.22 (18.85,23.9) | 4.68 (3.63,6.05) |
| High SDI | Denmark | 55 to 59 | 31.49 (28.35,34.99) | 6.87 (5.48,8.6) |
| High SDI | Denmark | 60 to 64 | 49.21 (44.65,54.23) | 9.93 (8.07,12.22) |
| High SDI | Denmark | 65 to 69 | 67.66 (61.41,74.54) | 14.39 (11.8,17.56) |
| High SDI | Denmark | 70 to 74 | 93.43 (83.48,104.56) | 20.78 (16.66,25.93) |
| High SDI | Denmark | 75 to 79 | 117.44 (104.05,132.54) | 28.6 (22.79,35.9) |
| High SDI | Denmark | 80 to 84 | 136.75 (119.59,156.38) | 40.06 (31.65,50.7) |
| High SDI | Denmark | 85 to 89 | 186.99 (160.94,217.26) | 46.32 (35.88,59.79) |
| High SDI | Estonia | 5 to 9 | 5.34 (3.02,9.45) | 0.24 (0.01,7.62) |
| High SDI | Estonia | 10 to 14 | 4.16 (2.38,7.28) | 0.22 (0.01,4.82) |
| High SDI | Estonia | 15 to 19 | 2.95 (1.66,5.23) | 0.59 (0.09,3.97) |
| High SDI | Estonia | 20 to 24 | 2.36 (1.34,4.16) | 1.22 (0.33,4.48) |
| High SDI | Estonia | 25 to 29 | 2.55 (1.47,4.41) | 1.41 (0.44,4.51) |
| High SDI | Estonia | 30 to 34 | 3.63 (2.25,5.85) | 1.26 (0.44,3.65) |
| High SDI | Estonia | 35 to 39 | 4.66 (3.06,7.1) | 1.58 (0.66,3.78) |
| High SDI | Estonia | 40 to 44 | 7.57 (5.36,10.69) | 1.89 (0.89,4.01) |
| High SDI | Estonia | 45 to 49 | 11.52 (8.5,15.62) | 2.94 (1.59,5.43) |
| High SDI | Estonia | 50 to 54 | 16.79 (12.77,22.07) | 4.73 (2.81,7.94) |
| High SDI | Estonia | 55 to 59 | 23.24 (18.04,29.95) | 7.38 (4.69,11.63) |
| High SDI | Estonia | 60 to 64 | 37.34 (29.71,46.92) | 10.55 (6.99,15.92) |
| High SDI | Estonia | 65 to 69 | 51.86 (41.44,64.89) | 16.05 (10.89,23.66) |
| High SDI | Estonia | 70 to 74 | 80.68 (61.7,105.48) | 22.47 (14.31,35.29) |
| High SDI | Estonia | 75 to 79 | 95.6 (71.34,128.11) | 29.4 (18.38,47.04) |
| High SDI | Estonia | 80 to 84 | 116.74 (84.17,161.91) | 40.93 (24.98,67.06) |
| High SDI | Estonia | 85 to 89 | 110.61 (72.1,169.69) | 39.85 (21.97,72.27) |
| High SDI | Finland | 5 to 9 | 1.45 (0.92,2.28) | 0.6 (0.18,2.03) |
| High SDI | Finland | 10 to 14 | 1.52 (1.01,2.29) | 0.58 (0.19,1.76) |
| High SDI | Finland | 15 to 19 | 2.17 (1.52,3.11) | 0.69 (0.26,1.79) |
| High SDI | Finland | 20 to 24 | 3.08 (2.26,4.2) | 1.12 (0.52,2.43) |
| High SDI | Finland | 25 to 29 | 4.18 (3.21,5.43) | 1.29 (0.66,2.53) |
| High SDI | Finland | 30 to 34 | 6.68 (5.36,8.33) | 1.55 (0.89,2.69) |
| High SDI | Finland | 35 to 39 | 9.3 (7.74,11.17) | 2.09 (1.34,3.26) |
| High SDI | Finland | 40 to 44 | 12.27 (10.54,14.28) | 2.75 (1.95,3.89) |
| High SDI | Finland | 45 to 49 | 17.49 (15.42,19.85) | 3.73 (2.83,4.93) |
| High SDI | Finland | 50 to 54 | 23.24 (20.78,26) | 5.47 (4.33,6.9) |
| High SDI | Finland | 55 to 59 | 31.96 (28.88,35.36) | 7.75 (6.31,9.51) |
| High SDI | Finland | 60 to 64 | 46.7 (42.5,51.32) | 10.94 (9.06,13.2) |
| High SDI | Finland | 65 to 69 | 59.42 (53.97,65.43) | 15.88 (13.25,19.04) |
| High SDI | Finland | 70 to 74 | 79 (70.68,88.29) | 23.23 (18.99,28.42) |
| High SDI | Finland | 75 to 79 | 97.04 (86.11,109.35) | 33.03 (26.88,40.59) |
| High SDI | Finland | 80 to 84 | 102.78 (90.07,117.29) | 45.4 (36.72,56.12) |
| High SDI | Finland | 85 to 89 | 124.7 (107.39,144.8) | 50.72 (40.36,63.75) |
| High SDI | France | 5 to 9 | 2.41 (2.13,2.72) | 0.66 (0.44,0.99) |
| High SDI | France | 10 to 14 | 2.33 (2.09,2.61) | 0.64 (0.45,0.91) |
| High SDI | France | 15 to 19 | 2.88 (2.61,3.17) | 0.89 (0.67,1.19) |
| High SDI | France | 20 to 24 | 3.73 (3.43,4.07) | 1.14 (0.91,1.45) |
| High SDI | France | 25 to 29 | 6.34 (5.91,6.79) | 1.35 (1.11,1.64) |
| High SDI | France | 30 to 34 | 8.43 (7.94,8.95) | 1.56 (1.32,1.84) |
| High SDI | France | 35 to 39 | 9.53 (9.03,10.06) | 2 (1.75,2.29) |
| High SDI | France | 40 to 44 | 12.09 (11.56,12.66) | 2.56 (2.3,2.85) |
| High SDI | France | 45 to 49 | 17.51 (16.85,18.2) | 3.53 (3.23,3.85) |
| High SDI | France | 50 to 54 | 22.54 (21.76,23.34) | 4.97 (4.61,5.36) |
| High SDI | France | 55 to 59 | 30.1 (29.15,31.08) | 7.06 (6.61,7.55) |
| High SDI | France | 60 to 64 | 44.97 (43.66,46.32) | 9.86 (9.28,10.48) |
| High SDI | France | 65 to 69 | 56.13 (54.47,57.84) | 13.98 (13.19,14.82) |
| High SDI | France | 70 to 74 | 79.89 (77.2,82.67) | 20.47 (19.19,21.84) |
| High SDI | France | 75 to 79 | 95.04 (91.65,98.56) | 28.72 (26.9,30.66) |
| High SDI | France | 80 to 84 | 113.15 (108.85,117.63) | 40.31 (37.71,43.1) |
| High SDI | France | 85 to 89 | 159.94 (153.39,166.76) | 52.62 (49.07,56.43) |
| High SDI | Germany | 5 to 9 | 3.45 (3.02,3.93) | 0.64 (0.42,0.96) |
| High SDI | Germany | 10 to 14 | 3.17 (2.82,3.57) | 0.61 (0.43,0.87) |
| High SDI | Germany | 15 to 19 | 3.6 (3.24,3.99) | 0.84 (0.63,1.12) |
| High SDI | Germany | 20 to 24 | 3.72 (3.39,4.08) | 0.95 (0.75,1.2) |
| High SDI | Germany | 25 to 29 | 4.97 (4.61,5.35) | 1.02 (0.84,1.24) |
| High SDI | Germany | 30 to 34 | 6.21 (5.83,6.61) | 1.14 (0.97,1.34) |
| High SDI | Germany | 35 to 39 | 7.36 (6.98,7.77) | 1.5 (1.31,1.7) |
| High SDI | Germany | 40 to 44 | 9.93 (9.5,10.38) | 2.06 (1.86,2.27) |
| High SDI | Germany | 45 to 49 | 13.5 (13,14.02) | 2.82 (2.6,3.07) |
| High SDI | Germany | 50 to 54 | 19.22 (18.61,19.86) | 4.22 (3.94,4.52) |
| High SDI | Germany | 55 to 59 | 26.41 (25.65,27.2) | 6.15 (5.79,6.52) |
| High SDI | Germany | 60 to 64 | 37.27 (36.25,38.32) | 8.84 (8.36,9.35) |
| High SDI | Germany | 65 to 69 | 49.09 (47.78,50.43) | 12.88 (12.22,13.58) |
| High SDI | Germany | 70 to 74 | 66.98 (64.96,69.07) | 19.04 (17.97,20.18) |
| High SDI | Germany | 75 to 79 | 82.85 (80.26,85.52) | 27.37 (25.82,29.02) |
| High SDI | Germany | 80 to 84 | 97.3 (94.02,100.68) | 37.7 (35.5,40.03) |
| High SDI | Germany | 85 to 89 | 118.35 (113.84,123.05) | 43.87 (41.11,46.82) |
| High SDI | Greenland | 5 to 9 | 1.75 (0.01,298.17) | - |
| High SDI | Greenland | 10 to 14 | 1.87 (0.02,232.5) | - |
| High SDI | Greenland | 15 to 19 | 2.12 (0.02,203.1) | - |
| High SDI | Greenland | 20 to 24 | 2.15 (0.03,160.66) | - |
| High SDI | Greenland | 25 to 29 | 2.13 (0.04,123.56) | - |
| High SDI | Greenland | 30 to 34 | 2.09 (0.05,87.51) | - |
| High SDI | Greenland | 35 to 39 | 2.21 (0.07,68.44) | - |
| High SDI | Greenland | 40 to 44 | 2.4 (0.11,53.96) | - |
| High SDI | Greenland | 45 to 49 | 2.67 (0.15,49.17) | - |
| High SDI | Greenland | 50 to 54 | 3.05 (0.19,49.79) | - |
| High SDI | Greenland | 55 to 59 | 25.88 (5.39,124.29) | - |
| High SDI | Greenland | 60 to 64 | 41.77 (10.42,167.43) | - |
| High SDI | Greenland | 65 to 69 | 56.37 (12.13,261.93) | - |
| High SDI | Greenland | 70 to 74 | 82.42 (10.29,660.09) | - |
| High SDI | Greenland | 75 to 79 | 64.66 (2.14,1956.03) | - |
| High SDI | Greenland | 80 to 84 | 164.13 (4.11,6561.38) | - |
| High SDI | Greenland | 85 to 89 | 374.28 (6.63,21113.49) | - |
| High SDI | Iceland | 5 to 9 | 1.95 (0.62,6.14) | 0.68 (0.01,90.48) |
| High SDI | Iceland | 10 to 14 | 3.31 (1.25,8.78) | 0.66 (0.01,64.38) |
| High SDI | Iceland | 15 to 19 | 3.68 (1.47,9.18) | 0.65 (0.01,48.04) |
| High SDI | Iceland | 20 to 24 | 3.55 (1.44,8.77) | 0.61 (0.01,35.4) |
| High SDI | Iceland | 25 to 29 | 7.14 (3.23,15.79) | 0.59 (0.01,24.45) |
| High SDI | Iceland | 30 to 34 | 9.55 (4.5,20.28) | 0.59 (0.02,17.81) |
| High SDI | Iceland | 35 to 39 | 8.64 (4.16,17.93) | 0.59 (0.03,13.82) |
| High SDI | Iceland | 40 to 44 | 12.93 (6.81,24.56) | 0.58 (0.03,10.65) |
| High SDI | Iceland | 45 to 49 | 16.69 (9.32,29.86) | 0.58 (0.04,9.19) |
| High SDI | Iceland | 50 to 54 | 20.05 (11.63,34.58) | 5.34 (1.34,21.32) |
| High SDI | Iceland | 55 to 59 | 28.17 (16.96,46.81) | 6.61 (2.09,20.93) |
| High SDI | Iceland | 60 to 64 | 40.52 (25.08,65.47) | 7.04 (2.2,22.5) |
| High SDI | Iceland | 65 to 69 | 56.02 (34.68,90.47) | 10.37 (3.6,29.92) |
| High SDI | Iceland | 70 to 74 | 91.27 (51.94,160.39) | 19.6 (5.84,65.76) |
| High SDI | Iceland | 75 to 79 | 96.81 (51.78,181.01) | 27.38 (7.92,94.72) |
| High SDI | Iceland | 80 to 84 | 108.45 (54.1,217.41) | 40.05 (11.31,141.89) |
| High SDI | Iceland | 85 to 89 | 159.71 (74.44,342.69) | 49.96 (12.83,194.46) |
| High SDI | Ireland | 5 to 9 | 1.65 (1.07,2.55) | 0.75 (0.21,2.75) |
| High SDI | Ireland | 10 to 14 | 1.86 (1.27,2.7) | 0.62 (0.19,2.01) |
| High SDI | Ireland | 15 to 19 | 2.44 (1.75,3.41) | 0.93 (0.37,2.34) |
| High SDI | Ireland | 20 to 24 | 2.9 (2.14,3.93) | 1.07 (0.47,2.45) |
| High SDI | Ireland | 25 to 29 | 4.95 (3.83,6.42) | 1.14 (0.54,2.42) |
| High SDI | Ireland | 30 to 34 | 6.37 (5.05,8.03) | 1.27 (0.66,2.44) |
| High SDI | Ireland | 35 to 39 | 7.16 (5.78,8.87) | 1.63 (0.94,2.82) |
| High SDI | Ireland | 40 to 44 | 10.03 (8.32,12.09) | 2.15 (1.37,3.36) |
| High SDI | Ireland | 45 to 49 | 14.99 (12.75,17.61) | 3.11 (2.15,4.51) |
| High SDI | Ireland | 50 to 54 | 21.63 (18.74,24.96) | 4.74 (3.47,6.48) |
| High SDI | Ireland | 55 to 59 | 30.57 (26.83,34.83) | 7 (5.33,9.21) |
| High SDI | Ireland | 60 to 64 | 50.57 (44.95,56.89) | 10.64 (8.31,13.63) |
| High SDI | Ireland | 65 to 69 | 66.59 (59.12,75.02) | 15.42 (12.17,19.56) |
| High SDI | Ireland | 70 to 74 | 93.83 (81.58,107.92) | 21.81 (16.68,28.51) |
| High SDI | Ireland | 75 to 79 | 112.34 (96.6,130.65) | 31.16 (23.71,40.95) |
| High SDI | Ireland | 80 to 84 | 134.14 (113.65,158.33) | 42.49 (32.01,56.38) |
| High SDI | Ireland | 85 to 89 | 169.65 (140.19,205.29) | 48.93 (35.91,66.68) |
| High SDI | Japan | 5 to 9 | 1.44 (1.18,1.77) | 0.66 (0.48,0.91) |
| High SDI | Japan | 10 to 14 | 1.39 (1.16,1.67) | 0.7 (0.53,0.92) |
| High SDI | Japan | 15 to 19 | 1.4 (1.19,1.65) | 0.9 (0.73,1.12) |
| High SDI | Japan | 20 to 24 | 1.63 (1.42,1.88) | 0.95 (0.79,1.15) |
| High SDI | Japan | 25 to 29 | 2.36 (2.1,2.65) | 0.96 (0.81,1.13) |
| High SDI | Japan | 30 to 34 | 3.43 (3.12,3.77) | 1.09 (0.95,1.25) |
| High SDI | Japan | 35 to 39 | 4.49 (4.15,4.86) | 1.35 (1.2,1.51) |
| High SDI | Japan | 40 to 44 | 6.5 (6.11,6.92) | 1.84 (1.69,2) |
| High SDI | Japan | 45 to 49 | 9.97 (9.48,10.47) | 2.61 (2.43,2.79) |
| High SDI | Japan | 50 to 54 | 14.88 (14.27,15.52) | 3.91 (3.69,4.13) |
| High SDI | Japan | 55 to 59 | 21.97 (21.18,22.79) | 5.87 (5.59,6.16) |
| High SDI | Japan | 60 to 64 | 33.35 (32.26,34.48) | 8.53 (8.16,8.91) |
| High SDI | Japan | 65 to 69 | 46.24 (44.76,47.76) | 12.79 (12.28,13.33) |
| High SDI | Japan | 70 to 74 | 68.34 (65.84,70.93) | 20.11 (19.21,21.05) |
| High SDI | Japan | 75 to 79 | 90.31 (86.85,93.9) | 30.59 (29.21,32.04) |
| High SDI | Japan | 80 to 84 | 119.58 (114.73,124.63) | 46.98 (44.81,49.25) |
| High SDI | Japan | 85 to 89 | 166.78 (159.47,174.42) | 64.68 (61.55,67.96) |
| High SDI | Kuwait | 5 to 9 | 1.58 (0.79,3.18) | 1.13 (0.3,4.34) |
| High SDI | Kuwait | 10 to 14 | 1.52 (0.79,2.91) | 1.14 (0.33,3.93) |
| High SDI | Kuwait | 15 to 19 | 1.79 (0.96,3.34) | 1.2 (0.38,3.84) |
| High SDI | Kuwait | 20 to 24 | 2.23 (1.29,3.84) | 1.4 (0.52,3.81) |
| High SDI | Kuwait | 25 to 29 | 3.32 (2.09,5.29) | 1.26 (0.52,3.02) |
| High SDI | Kuwait | 30 to 34 | 4.19 (2.73,6.42) | 1.38 (0.62,3.06) |
| High SDI | Kuwait | 35 to 39 | 4.67 (3.1,7.02) | 1.64 (0.78,3.43) |
| High SDI | Kuwait | 40 to 44 | 5.5 (3.75,8.07) | 2.17 (1.12,4.22) |
| High SDI | Kuwait | 45 to 49 | 8.52 (5.98,12.14) | 2.97 (1.61,5.49) |
| High SDI | Kuwait | 50 to 54 | 9.96 (6.97,14.24) | 3.66 (2.02,6.63) |
| High SDI | Kuwait | 55 to 59 | 13.84 (9.7,19.75) | 5.28 (2.92,9.54) |
| High SDI | Kuwait | 60 to 64 | 21.42 (15.16,30.26) | 7.13 (3.99,12.77) |
| High SDI | Kuwait | 65 to 69 | 26.02 (17.99,37.63) | 10.57 (5.98,18.69) |
| High SDI | Kuwait | 70 to 74 | 36.64 (23.58,56.92) | 15.61 (8.05,30.28) |
| High SDI | Kuwait | 75 to 79 | 34.85 (20.64,58.83) | 15.42 (7.3,32.56) |
| High SDI | Kuwait | 80 to 84 | 38.01 (20.84,69.31) | 20.54 (9.12,46.3) |
| High SDI | Kuwait | 85 to 89 | 46.08 (22.14,95.93) | 28.42 (11.5,70.24) |
| High SDI | Latvia | 5 to 9 | 0.22 (0.06,0.76) | 0.18 (0.02,1.5) |
| High SDI | Latvia | 10 to 14 | 0.23 (0.07,0.7) | 0.04 (0,0.78) |
| High SDI | Latvia | 15 to 19 | 0.24 (0.09,0.69) | 0.4 (0.11,1.42) |
| High SDI | Latvia | 20 to 24 | 0.51 (0.22,1.17) | 0.43 (0.14,1.34) |
| High SDI | Latvia | 25 to 29 | 0.74 (0.36,1.5) | 0.46 (0.16,1.32) |
| High SDI | Latvia | 30 to 34 | 1.17 (0.65,2.12) | 0.5 (0.19,1.35) |
| High SDI | Latvia | 35 to 39 | 1.74 (1.04,2.88) | 1.06 (0.51,2.21) |
| High SDI | Latvia | 40 to 44 | 2.8 (1.84,4.27) | 1.69 (0.94,3.04) |
| High SDI | Latvia | 45 to 49 | 5.12 (3.63,7.21) | 2.61 (1.59,4.28) |
| High SDI | Latvia | 50 to 54 | 9.03 (6.75,12.07) | 4.37 (2.88,6.64) |
| High SDI | Latvia | 55 to 59 | 15.02 (11.66,19.33) | 7.29 (5.09,10.46) |
| High SDI | Latvia | 60 to 64 | 22.97 (18.14,29.09) | 11.27 (8.07,15.73) |
| High SDI | Latvia | 65 to 69 | 36.09 (28.67,45.42) | 17.34 (12.54,23.98) |
| High SDI | Latvia | 70 to 74 | 49.84 (37.87,65.59) | 26.67 (18.37,38.72) |
| High SDI | Latvia | 75 to 79 | 60.2 (44.69,81.08) | 34.77 (23.47,51.51) |
| High SDI | Latvia | 80 to 84 | 74.07 (52.88,103.74) | 44.12 (28.66,67.91) |
| High SDI | Latvia | 85 to 89 | 80.8 (52.56,124.21) | 40.79 (23.57,70.59) |
| High SDI | Lithuania | 5 to 9 | 0.5 (0.24,1.05) | 0.19 (0.04,0.95) |
| High SDI | Lithuania | 10 to 14 | 0.49 (0.24,0.98) | 0.25 (0.07,0.89) |
| High SDI | Lithuania | 15 to 19 | 0.65 (0.35,1.2) | 0.27 (0.08,0.83) |
| High SDI | Lithuania | 20 to 24 | 0.9 (0.52,1.56) | 0.29 (0.1,0.83) |
| High SDI | Lithuania | 25 to 29 | 1.83 (1.18,2.82) | 0.49 (0.21,1.15) |
| High SDI | Lithuania | 30 to 34 | 2.5 (1.7,3.67) | 0.65 (0.31,1.38) |
| High SDI | Lithuania | 35 to 39 | 2.8 (1.95,4.01) | 0.99 (0.54,1.84) |
| High SDI | Lithuania | 40 to 44 | 3.57 (2.6,4.92) | 1.49 (0.9,2.47) |
| High SDI | Lithuania | 45 to 49 | 6.07 (4.63,7.95) | 2.39 (1.55,3.67) |
| High SDI | Lithuania | 50 to 54 | 9.6 (7.57,12.18) | 4 (2.77,5.75) |
| High SDI | Lithuania | 55 to 59 | 14.55 (11.74,18.04) | 6.48 (4.7,8.93) |
| High SDI | Lithuania | 60 to 64 | 23.06 (18.89,28.14) | 10.02 (7.45,13.48) |
| High SDI | Lithuania | 65 to 69 | 31.76 (25.97,38.83) | 15.23 (11.36,20.41) |
| High SDI | Lithuania | 70 to 74 | 45.11 (35.72,56.97) | 22.6 (16.21,31.51) |
| High SDI | Lithuania | 75 to 79 | 54.65 (42.55,70.2) | 31.09 (22.01,43.91) |
| High SDI | Lithuania | 80 to 84 | 63.18 (47.65,83.76) | 40.29 (27.74,58.51) |
| High SDI | Lithuania | 85 to 89 | 64.98 (45.31,93.2) | 39.64 (25.17,62.41) |
| High SDI | Luxembourg | 5 to 9 | 5.52 (1.28,23.88) | 1.58 (0.02,142.02) |
| High SDI | Luxembourg | 10 to 14 | 4.62 (1.32,16.2) | 1.54 (0.02,98.47) |
| High SDI | Luxembourg | 15 to 19 | 5.09 (1.66,15.55) | 1.56 (0.03,74.79) |
| High SDI | Luxembourg | 20 to 24 | 4.26 (1.5,12.05) | 1.21 (0.04,40.48) |
| High SDI | Luxembourg | 25 to 29 | 6.49 (2.91,14.46) | 0.81 (0.03,20.04) |
| High SDI | Luxembourg | 30 to 34 | 8.72 (4.51,16.87) | 0.6 (0.03,11.59) |
| High SDI | Luxembourg | 35 to 39 | 9.05 (4.93,16.64) | 2.75 (0.71,10.65) |
| High SDI | Luxembourg | 40 to 44 | 11.23 (6.65,18.96) | 3.18 (1.06,9.6) |
| High SDI | Luxembourg | 45 to 49 | 16.16 (10.26,25.45) | 3.2 (1.16,8.83) |
| High SDI | Luxembourg | 50 to 54 | 22.39 (14.95,33.54) | 4.84 (1.95,12.01) |
| High SDI | Luxembourg | 55 to 59 | 30.72 (21.26,44.38) | 6.68 (3.07,14.51) |
| High SDI | Luxembourg | 60 to 64 | 47.67 (33.7,67.44) | 10.66 (5.26,21.59) |
| High SDI | Luxembourg | 65 to 69 | 59.35 (41.65,84.56) | 14.08 (7.04,28.15) |
| High SDI | Luxembourg | 70 to 74 | 86.23 (57.4,129.55) | 20.3 (9.37,43.98) |
| High SDI | Luxembourg | 75 to 79 | 102.4 (66.3,158.16) | 29.58 (13.52,64.69) |
| High SDI | Luxembourg | 80 to 84 | 113.85 (70.67,183.42) | 39.42 (17.57,88.45) |
| High SDI | Luxembourg | 85 to 89 | 149.12 (87.64,253.75) | 46.09 (19.23,110.46) |
| High SDI | Monaco | 5 to 9 | 6.83 (0.06,767.43) | 8.62 (0.03,2332.57) |
| High SDI | Monaco | 10 to 14 | 6.57 (0.08,531.87) | 8.13 (0.04,1673.04) |
| High SDI | Monaco | 15 to 19 | 6.62 (0.11,409.42) | 7.77 (0.05,1269.08) |
| High SDI | Monaco | 20 to 24 | 6.06 (0.14,265.5) | 7.56 (0.06,982.58) |
| High SDI | Monaco | 25 to 29 | 5.11 (0.16,165.74) | 6.63 (0.06,684.3) |
| High SDI | Monaco | 30 to 34 | 4.55 (0.18,114.1) | 5.8 (0.07,474.55) |
| High SDI | Monaco | 35 to 39 | 4.08 (0.2,83.6) | 5.03 (0.08,330.49) |
| High SDI | Monaco | 40 to 44 | 19.76 (2.59,150.81) | 4.6 (0.11,187.25) |
| High SDI | Monaco | 45 to 49 | 30.01 (5.99,150.29) | 4.44 (0.13,151.93) |
| High SDI | Monaco | 50 to 54 | 39.11 (11.99,127.51) | 4.21 (0.13,132.88) |
| High SDI | Monaco | 55 to 59 | 41.29 (13.11,130) | 4.04 (0.13,128.32) |
| High SDI | Monaco | 60 to 64 | 43.75 (13.91,137.58) | 3.93 (0.11,134.94) |
| High SDI | Monaco | 65 to 69 | 66.68 (23.77,187.06) | 4.09 (0.11,158.33) |
| High SDI | Monaco | 70 to 74 | 87.81 (26.08,295.66) | 43.98 (1.65,1171.71) |
| High SDI | Monaco | 75 to 79 | 123.09 (35.24,429.98) | 47.83 (1.67,1371.18) |
| High SDI | Monaco | 80 to 84 | 100.5 (23.88,422.89) | 73.14 (2.4,2227.72) |
| High SDI | Monaco | 85 to 89 | 168.04 (36.55,772.44) | 87.68 (2.64,2906.28) |
| High SDI | Netherlands | 5 to 9 | 1.63 (1.24,2.16) | 0.6 (0.27,1.36) |
| High SDI | Netherlands | 10 to 14 | 1.78 (1.39,2.29) | 0.61 (0.29,1.24) |
| High SDI | Netherlands | 15 to 19 | 2.01 (1.6,2.51) | 0.67 (0.36,1.28) |
| High SDI | Netherlands | 20 to 24 | 2.39 (1.97,2.91) | 0.73 (0.43,1.25) |
| High SDI | Netherlands | 25 to 29 | 4.11 (3.53,4.79) | 0.84 (0.54,1.32) |
| High SDI | Netherlands | 30 to 34 | 6.33 (5.59,7.16) | 1.08 (0.75,1.55) |
| High SDI | Netherlands | 35 to 39 | 8.42 (7.59,9.35) | 1.46 (1.09,1.95) |
| High SDI | Netherlands | 40 to 44 | 12.62 (11.6,13.72) | 2.04 (1.62,2.57) |
| High SDI | Netherlands | 45 to 49 | 19.53 (18.23,20.91) | 2.88 (2.4,3.46) |
| High SDI | Netherlands | 50 to 54 | 27.6 (26,29.29) | 4.05 (3.47,4.73) |
| High SDI | Netherlands | 55 to 59 | 39.28 (37.21,41.46) | 5.79 (5.04,6.66) |
| High SDI | Netherlands | 60 to 64 | 61.19 (58.23,64.3) | 8.61 (7.58,9.78) |
| High SDI | Netherlands | 65 to 69 | 79.89 (75.99,84) | 12 (10.6,13.57) |
| High SDI | Netherlands | 70 to 74 | 118.13 (111.56,125.09) | 17.85 (15.57,20.48) |
| High SDI | Netherlands | 75 to 79 | 143.11 (134.59,152.17) | 24.78 (21.54,28.52) |
| High SDI | Netherlands | 80 to 84 | 164.27 (153.58,175.71) | 32.71 (28.28,37.84) |
| High SDI | Netherlands | 85 to 89 | 190.13 (175.96,205.45) | 37.02 (31.58,43.39) |
| High SDI | New Zealand | 5 to 9 | 2.97 (1.99,4.43) | 0.84 (0.22,3.28) |
| High SDI | New Zealand | 10 to 14 | 3.69 (2.6,5.24) | 0.81 (0.24,2.67) |
| High SDI | New Zealand | 15 to 19 | 4.52 (3.29,6.19) | 1.41 (0.58,3.44) |
| High SDI | New Zealand | 20 to 24 | 3.75 (2.76,5.09) | 1.17 (0.52,2.65) |
| High SDI | New Zealand | 25 to 29 | 6.25 (4.85,8.06) | 1.43 (0.72,2.84) |
| High SDI | New Zealand | 30 to 34 | 7.45 (5.93,9.35) | 1.59 (0.88,2.88) |
| High SDI | New Zealand | 35 to 39 | 9.05 (7.39,11.09) | 1.91 (1.15,3.15) |
| High SDI | New Zealand | 40 to 44 | 12.37 (10.41,14.7) | 2.65 (1.78,3.96) |
| High SDI | New Zealand | 45 to 49 | 17.9 (15.47,20.71) | 3.76 (2.71,5.22) |
| High SDI | New Zealand | 50 to 54 | 26.93 (23.75,30.53) | 5.71 (4.34,7.51) |
| High SDI | New Zealand | 55 to 59 | 40.39 (36.11,45.18) | 8.76 (6.9,11.12) |
| High SDI | New Zealand | 60 to 64 | 57.98 (52.23,64.37) | 12.3 (9.86,15.33) |
| High SDI | New Zealand | 65 to 69 | 76.13 (68.53,84.57) | 17.09 (13.78,21.19) |
| High SDI | New Zealand | 70 to 74 | 107.07 (94.89,120.81) | 23.99 (18.86,30.5) |
| High SDI | New Zealand | 75 to 79 | 124.19 (109.17,141.28) | 31.48 (24.61,40.27) |
| High SDI | New Zealand | 80 to 84 | 153.81 (133.96,176.61) | 42.99 (33.34,55.44) |
| High SDI | New Zealand | 85 to 89 | 211.95 (182.17,246.58) | 52.03 (39.59,68.37) |
| High SDI | Norway | 5 to 9 | 2.64 (1.69,4.12) | 1.02 (0.26,3.94) |
| High SDI | Norway | 10 to 14 | 2.26 (1.48,3.45) | 0.89 (0.26,3.1) |
| High SDI | Norway | 15 to 19 | 3.54 (2.5,5.03) | 0.89 (0.3,2.69) |
| High SDI | Norway | 20 to 24 | 4.02 (2.94,5.51) | 1.3 (0.56,3.04) |
| High SDI | Norway | 25 to 29 | 5.6 (4.3,7.3) | 1.18 (0.55,2.55) |
| High SDI | Norway | 30 to 34 | 7.09 (5.63,8.92) | 1.26 (0.65,2.43) |
| High SDI | Norway | 35 to 39 | 7.81 (6.34,9.62) | 1.61 (0.93,2.76) |
| High SDI | Norway | 40 to 44 | 11.75 (9.89,13.95) | 2.48 (1.65,3.73) |
| High SDI | Norway | 45 to 49 | 16.36 (14.15,18.92) | 3.32 (2.37,4.64) |
| High SDI | Norway | 50 to 54 | 21.79 (19.13,24.82) | 4.51 (3.39,6) |
| High SDI | Norway | 55 to 59 | 28.05 (24.89,31.62) | 6.17 (4.78,7.97) |
| High SDI | Norway | 60 to 64 | 44.74 (40.12,49.9) | 9.53 (7.58,11.98) |
| High SDI | Norway | 65 to 69 | 57.52 (51.32,64.47) | 13.65 (10.89,17.11) |
| High SDI | Norway | 70 to 74 | 75.46 (66.25,85.96) | 18.81 (14.67,24.12) |
| High SDI | Norway | 75 to 79 | 92.13 (80.18,105.85) | 28.05 (21.81,36.07) |
| High SDI | Norway | 80 to 84 | 97.1 (83.38,113.09) | 37.21 (28.73,48.17) |
| High SDI | Norway | 85 to 89 | 134.12 (113.68,158.24) | 48.23 (36.79,63.23) |
| High SDI | Poland | 5 to 9 | 0.82 (0.66,1.03) | 0.61 (0.41,0.93) |
| High SDI | Poland | 10 to 14 | 0.8 (0.65,0.98) | 0.6 (0.42,0.85) |
| High SDI | Poland | 15 to 19 | 1.06 (0.89,1.27) | 0.75 (0.55,1.01) |
| High SDI | Poland | 20 to 24 | 1.16 (0.98,1.36) | 0.86 (0.65,1.12) |
| High SDI | Poland | 25 to 29 | 1.61 (1.4,1.87) | 1.07 (0.85,1.36) |
| High SDI | Poland | 30 to 34 | 2.32 (2.05,2.63) | 1.16 (0.94,1.43) |
| High SDI | Poland | 35 to 39 | 3.14 (2.82,3.5) | 1.63 (1.38,1.94) |
| High SDI | Poland | 40 to 44 | 4.39 (4.01,4.8) | 2.24 (1.96,2.57) |
| High SDI | Poland | 45 to 49 | 6.75 (6.27,7.27) | 3.07 (2.75,3.44) |
| High SDI | Poland | 50 to 54 | 10.07 (9.44,10.73) | 4.92 (4.48,5.4) |
| High SDI | Poland | 55 to 59 | 14.77 (13.95,15.64) | 7.22 (6.66,7.84) |
| High SDI | Poland | 60 to 64 | 21.68 (20.54,22.88) | 10.22 (9.46,11.03) |
| High SDI | Poland | 65 to 69 | 31.03 (29.41,32.74) | 14.69 (13.63,15.84) |
| High SDI | Poland | 70 to 74 | 40.01 (37.47,42.72) | 19.92 (18.25,21.75) |
| High SDI | Poland | 75 to 79 | 49.35 (45.95,53) | 25.81 (23.53,28.31) |
| High SDI | Poland | 80 to 84 | 54.63 (50.37,59.24) | 31.64 (28.62,34.97) |
| High SDI | Poland | 85 to 89 | 64.82 (58.68,71.6) | 35.99 (31.98,40.51) |
| High SDI | Puerto Rico | 5 to 9 | 1.53 (0.9,2.59) | 0.72 (0.22,2.33) |
| High SDI | Puerto Rico | 10 to 14 | 1.1 (0.66,1.84) | 0.73 (0.28,1.95) |
| High SDI | Puerto Rico | 15 to 19 | 2.31 (1.57,3.4) | 1.49 (0.74,3.01) |
| High SDI | Puerto Rico | 20 to 24 | 3.8 (2.73,5.3) | 2.57 (1.44,4.58) |
| High SDI | Puerto Rico | 25 to 29 | 5.92 (4.43,7.91) | 2.84 (1.68,4.8) |
| High SDI | Puerto Rico | 30 to 34 | 8.28 (6.41,10.68) | 3.03 (1.89,4.86) |
| High SDI | Puerto Rico | 35 to 39 | 7.54 (5.89,9.64) | 3.17 (2.06,4.88) |
| High SDI | Puerto Rico | 40 to 44 | 8.21 (6.57,10.25) | 3.69 (2.56,5.32) |
| High SDI | Puerto Rico | 45 to 49 | 12.49 (10.32,15.13) | 4.81 (3.49,6.62) |
| High SDI | Puerto Rico | 50 to 54 | 17.83 (15.07,21.09) | 6.18 (4.65,8.23) |
| High SDI | Puerto Rico | 55 to 59 | 24.03 (20.58,28.05) | 8.35 (6.44,10.83) |
| High SDI | Puerto Rico | 60 to 64 | 32.81 (28.28,38.08) | 10.55 (8.21,13.56) |
| High SDI | Puerto Rico | 65 to 69 | 38.77 (33.32,45.11) | 13.71 (10.71,17.55) |
| High SDI | Puerto Rico | 70 to 74 | 55.34 (46.54,65.81) | 17.96 (13.6,23.73) |
| High SDI | Puerto Rico | 75 to 79 | 62.56 (51.86,75.46) | 21.75 (16.25,29.12) |
| High SDI | Puerto Rico | 80 to 84 | 64.48 (52.18,79.67) | 27.02 (19.82,36.84) |
| High SDI | Puerto Rico | 85 to 89 | 72.96 (57.08,93.27) | 27.54 (19.38,39.13) |
| High SDI | Qatar | 5 to 9 | 0.41 (0.07,2.45) | 0.5 (0.01,18.19) |
| High SDI | Qatar | 10 to 14 | 0.53 (0.11,2.52) | 0.49 (0.02,12.54) |
| High SDI | Qatar | 15 to 19 | 1.11 (0.31,4.04) | 0.48 (0.02,9.97) |
| High SDI | Qatar | 20 to 24 | 0.9 (0.29,2.72) | 1.12 (0.15,8.52) |
| High SDI | Qatar | 25 to 29 | 1.4 (0.55,3.59) | 1.42 (0.29,6.96) |
| High SDI | Qatar | 30 to 34 | 1.85 (0.8,4.27) | 1.44 (0.37,5.58) |
| High SDI | Qatar | 35 to 39 | 2.44 (1.15,5.19) | 1.78 (0.56,5.64) |
| High SDI | Qatar | 40 to 44 | 2.86 (1.41,5.8) | 1.87 (0.66,5.25) |
| High SDI | Qatar | 45 to 49 | 4.92 (2.57,9.42) | 2.81 (1.07,7.39) |
| High SDI | Qatar | 50 to 54 | 6.57 (3.55,12.19) | 2.99 (1.12,7.97) |
| High SDI | Qatar | 55 to 59 | 10.92 (6.04,19.75) | 5.26 (2.17,12.78) |
| High SDI | Qatar | 60 to 64 | 19.83 (10.94,35.94) | 7.52 (2.96,19.11) |
| High SDI | Qatar | 65 to 69 | 32.28 (16.56,62.92) | 13.57 (5,36.78) |
| High SDI | Qatar | 70 to 74 | 56.25 (23.54,134.38) | 21.87 (6.1,78.44) |
| High SDI | Qatar | 75 to 79 | 52.92 (16.74,167.27) | 22.71 (4.66,110.6) |
| High SDI | Qatar | 80 to 84 | 41.65 (8.81,196.93) | 24.22 (3.84,152.72) |
| High SDI | Qatar | 85 to 89 | 46.55 (1.76,1231.39) | 25.63 (0.85,770.6) |
| High SDI | Republic of Korea | 5 to 9 | 0.59 (0.47,0.74) | 1.15 (0.77,1.72) |
| High SDI | Republic of Korea | 10 to 14 | 0.54 (0.44,0.66) | 0.96 (0.67,1.37) |
| High SDI | Republic of Korea | 15 to 19 | 0.57 (0.48,0.69) | 1.03 (0.76,1.39) |
| High SDI | Republic of Korea | 20 to 24 | 0.61 (0.52,0.73) | 0.94 (0.72,1.24) |
| High SDI | Republic of Korea | 25 to 29 | 1.07 (0.93,1.23) | 0.99 (0.78,1.25) |
| High SDI | Republic of Korea | 30 to 34 | 1.69 (1.5,1.89) | 1.12 (0.92,1.37) |
| High SDI | Republic of Korea | 35 to 39 | 2.36 (2.13,2.6) | 1.37 (1.15,1.62) |
| High SDI | Republic of Korea | 40 to 44 | 3.52 (3.23,3.84) | 1.76 (1.52,2.02) |
| High SDI | Republic of Korea | 45 to 49 | 5.49 (5.09,5.91) | 2.27 (2,2.57) |
| High SDI | Republic of Korea | 50 to 54 | 8.19 (7.66,8.75) | 3.04 (2.71,3.4) |
| High SDI | Republic of Korea | 55 to 59 | 12.28 (11.57,13.04) | 4.23 (3.82,4.68) |
| High SDI | Republic of Korea | 60 to 64 | 19.44 (18.38,20.56) | 5.83 (5.29,6.42) |
| High SDI | Republic of Korea | 65 to 69 | 28.13 (26.59,29.75) | 8.42 (7.67,9.24) |
| High SDI | Republic of Korea | 70 to 74 | 43.87 (41.03,46.92) | 12.39 (11.14,13.78) |
| High SDI | Republic of Korea | 75 to 79 | 56.36 (52.39,60.63) | 17.02 (15.25,19) |
| High SDI | Republic of Korea | 80 to 84 | 74.56 (68.59,81.05) | 24.18 (21.48,27.21) |
| High SDI | Republic of Korea | 85 to 89 | 103.08 (93.11,114.12) | 29.02 (25.27,33.33) |
| High SDI | San Marino | 5 to 9 | 8.17 (0.09,736.99) | 8.57 (0.03,2316.63) |
| High SDI | San Marino | 10 to 14 | 8.18 (0.13,524.44) | 8.08 (0.04,1661.87) |
| High SDI | San Marino | 15 to 19 | 7.64 (0.17,344.81) | 7.73 (0.05,1262.97) |
| High SDI | San Marino | 20 to 24 | 6.8 (0.21,224.11) | 7.28 (0.06,946.87) |
| High SDI | San Marino | 25 to 29 | 6.13 (0.24,156.38) | 6.83 (0.07,704.34) |
| High SDI | San Marino | 30 to 34 | 5.8 (0.28,120.33) | 6.43 (0.08,526.18) |
| High SDI | San Marino | 35 to 39 | 31.12 (4.58,211.21) | 6.11 (0.09,401.25) |
| High SDI | San Marino | 40 to 44 | 42.97 (8.42,219.29) | 5.86 (0.14,238.52) |
| High SDI | San Marino | 45 to 49 | 47.93 (13.77,166.87) | 5.67 (0.17,194.09) |
| High SDI | San Marino | 50 to 54 | 59.45 (20.05,176.25) | 5.53 (0.18,174.33) |
| High SDI | San Marino | 55 to 59 | 66.52 (22.85,193.64) | 5.46 (0.17,173.68) |
| High SDI | San Marino | 60 to 64 | 95.22 (36.69,247.11) | 5.45 (0.16,187.13) |
| High SDI | San Marino | 65 to 69 | 107.03 (40.02,286.26) | 5.56 (0.14,215.32) |
| High SDI | San Marino | 70 to 74 | 176.07 (57.86,535.77) | 58.26 (2.19,1549.85) |
| High SDI | San Marino | 75 to 79 | 195.23 (60.35,631.58) | 63.86 (2.24,1820.6) |
| High SDI | San Marino | 80 to 84 | 175.92 (46.79,661.42) | 75.78 (2.55,2254.94) |
| High SDI | San Marino | 85 to 89 | 208.94 (48.58,898.74) | 102.76 (3.32,3180.28) |
| High SDI | Saudi Arabia | 5 to 9 | 0.14 (0.11,0.19) | 0.2 (0.13,0.31) |
| High SDI | Saudi Arabia | 10 to 14 | 0.16 (0.12,0.21) | 0.19 (0.12,0.28) |
| High SDI | Saudi Arabia | 15 to 19 | 0.26 (0.2,0.33) | 0.31 (0.22,0.45) |
| High SDI | Saudi Arabia | 20 to 24 | 0.42 (0.34,0.52) | 0.45 (0.33,0.62) |
| High SDI | Saudi Arabia | 25 to 29 | 0.81 (0.67,0.99) | 0.61 (0.46,0.82) |
| High SDI | Saudi Arabia | 30 to 34 | 1.17 (0.98,1.4) | 0.75 (0.58,0.97) |
| High SDI | Saudi Arabia | 35 to 39 | 1.73 (1.47,2.04) | 1.12 (0.89,1.42) |
| High SDI | Saudi Arabia | 40 to 44 | 2.74 (2.36,3.19) | 1.71 (1.38,2.1) |
| High SDI | Saudi Arabia | 45 to 49 | 4.95 (4.31,5.68) | 2.85 (2.36,3.44) |
| High SDI | Saudi Arabia | 50 to 54 | 7.92 (6.96,9) | 4.56 (3.84,5.42) |
| High SDI | Saudi Arabia | 55 to 59 | 13.24 (11.74,14.93) | 7.57 (6.46,8.88) |
| High SDI | Saudi Arabia | 60 to 64 | 22.1 (19.63,24.89) | 11.94 (10.19,13.98) |
| High SDI | Saudi Arabia | 65 to 69 | 34.69 (30.65,39.26) | 19.73 (16.82,23.15) |
| High SDI | Saudi Arabia | 70 to 74 | 51.71 (44,60.77) | 29.68 (24.32,36.22) |
| High SDI | Saudi Arabia | 75 to 79 | 64.26 (52.58,78.53) | 39.83 (31.54,50.3) |
| High SDI | Saudi Arabia | 80 to 84 | 85.65 (66.94,109.6) | 59.27 (45.32,77.51) |
| High SDI | Saudi Arabia | 85 to 89 | 110.19 (79.87,152.03) | 71.77 (51.01,100.98) |
| High SDI | Singapore | 5 to 9 | 0.63 (0.29,1.34) | 0.87 (0.21,3.62) |
| High SDI | Singapore | 10 to 14 | 0.93 (0.49,1.75) | 1.04 (0.32,3.43) |
| High SDI | Singapore | 15 to 19 | 0.81 (0.44,1.48) | 0.85 (0.28,2.52) |
| High SDI | Singapore | 20 to 24 | 0.89 (0.53,1.51) | 1.14 (0.49,2.67) |
| High SDI | Singapore | 25 to 29 | 1.36 (0.88,2.09) | 0.94 (0.43,2.04) |
| High SDI | Singapore | 30 to 34 | 1.99 (1.38,2.87) | 0.99 (0.5,1.94) |
| High SDI | Singapore | 35 to 39 | 2.58 (1.87,3.56) | 1.31 (0.74,2.31) |
| High SDI | Singapore | 40 to 44 | 4.03 (3.07,5.28) | 1.71 (1.06,2.76) |
| High SDI | Singapore | 45 to 49 | 6.4 (5.08,8.06) | 2.45 (1.64,3.64) |
| High SDI | Singapore | 50 to 54 | 10.14 (8.3,12.39) | 3.49 (2.47,4.94) |
| High SDI | Singapore | 55 to 59 | 16.16 (13.58,19.23) | 5.49 (4.07,7.4) |
| High SDI | Singapore | 60 to 64 | 25.25 (21.52,29.63) | 7.74 (5.87,10.22) |
| High SDI | Singapore | 65 to 69 | 36.27 (30.88,42.61) | 11.58 (8.87,15.12) |
| High SDI | Singapore | 70 to 74 | 52.95 (43.34,64.69) | 16.63 (12.12,22.83) |
| High SDI | Singapore | 75 to 79 | 65.45 (52.35,81.81) | 22.4 (16.04,31.28) |
| High SDI | Singapore | 80 to 84 | 76.27 (59.08,98.47) | 28.57 (19.94,40.94) |
| High SDI | Singapore | 85 to 89 | 87.07 (63.76,118.91) | 30.19 (19.84,45.92) |
| High SDI | Slovakia | 5 to 9 | 1.08 (0.64,1.83) | 0.67 (0.23,1.93) |
| High SDI | Slovakia | 10 to 14 | 0.86 (0.52,1.43) | 0.59 (0.22,1.58) |
| High SDI | Slovakia | 15 to 19 | 1 (0.64,1.58) | 0.64 (0.27,1.51) |
| High SDI | Slovakia | 20 to 24 | 1.26 (0.84,1.89) | 0.7 (0.33,1.5) |
| High SDI | Slovakia | 25 to 29 | 2.1 (1.5,2.94) | 0.86 (0.44,1.67) |
| High SDI | Slovakia | 30 to 34 | 2.95 (2.21,3.94) | 0.92 (0.51,1.67) |
| High SDI | Slovakia | 35 to 39 | 3.92 (3.05,5.03) | 1.44 (0.9,2.3) |
| High SDI | Slovakia | 40 to 44 | 5.73 (4.68,7.03) | 1.95 (1.34,2.84) |
| High SDI | Slovakia | 45 to 49 | 8.8 (7.39,10.47) | 2.96 (2.18,4.04) |
| High SDI | Slovakia | 50 to 54 | 13.85 (11.93,16.08) | 4.67 (3.6,6.06) |
| High SDI | Slovakia | 55 to 59 | 20.5 (17.94,23.42) | 6.81 (5.41,8.57) |
| High SDI | Slovakia | 60 to 64 | 32.29 (28.59,36.48) | 10.42 (8.44,12.85) |
| High SDI | Slovakia | 65 to 69 | 44.89 (39.67,50.79) | 15.16 (12.33,18.63) |
| High SDI | Slovakia | 70 to 74 | 58.3 (50.11,67.83) | 20.83 (16.35,26.54) |
| High SDI | Slovakia | 75 to 79 | 70.68 (59.87,83.43) | 28.25 (21.93,36.41) |
| High SDI | Slovakia | 80 to 84 | 75.99 (62.56,92.3) | 33.62 (25.42,44.46) |
| High SDI | Slovakia | 85 to 89 | 92 (72.44,116.86) | 35.2 (25.15,49.27) |
| High SDI | Slovenia | 5 to 9 | 0.68 (0.32,1.42) | 0.14 (0.01,3.7) |
| High SDI | Slovenia | 10 to 14 | 0.97 (0.53,1.78) | 0.13 (0.01,2.45) |
| High SDI | Slovenia | 15 to 19 | 1.22 (0.72,2.08) | 0.76 (0.19,3.04) |
| High SDI | Slovenia | 20 to 24 | 2.51 (1.64,3.82) | 1.01 (0.31,3.28) |
| High SDI | Slovenia | 25 to 29 | 4.61 (3.27,6.51) | 0.94 (0.32,2.73) |
| High SDI | Slovenia | 30 to 34 | 6.16 (4.56,8.33) | 0.87 (0.32,2.33) |
| High SDI | Slovenia | 35 to 39 | 8.72 (6.72,11.31) | 1.49 (0.7,3.18) |
| High SDI | Slovenia | 40 to 44 | 12.89 (10.39,15.98) | 1.94 (1.06,3.54) |
| High SDI | Slovenia | 45 to 49 | 19.95 (16.6,23.98) | 3.24 (2.01,5.22) |
| High SDI | Slovenia | 50 to 54 | 30.51 (25.97,35.84) | 5.05 (3.39,7.52) |
| High SDI | Slovenia | 55 to 59 | 42.3 (36.47,49.06) | 7.96 (5.66,11.2) |
| High SDI | Slovenia | 60 to 64 | 66.63 (58.21,76.27) | 11.81 (8.65,16.11) |
| High SDI | Slovenia | 65 to 69 | 93.27 (81.51,106.73) | 18.62 (13.93,24.89) |
| High SDI | Slovenia | 70 to 74 | 140.59 (119.36,165.59) | 28.43 (20.26,39.89) |
| High SDI | Slovenia | 75 to 79 | 186.06 (156.09,221.79) | 43.73 (30.96,61.75) |
| High SDI | Slovenia | 80 to 84 | 236.38 (194.61,287.11) | 70.63 (49.47,100.84) |
| High SDI | Slovenia | 85 to 89 | 313.21 (249.84,392.65) | 78.96 (53.09,117.43) |
| High SDI | Sweden | 5 to 9 | 1.88 (1.26,2.81) | 0.46 (0.14,1.51) |
| High SDI | Sweden | 10 to 14 | 2.02 (1.41,2.9) | 0.43 (0.15,1.29) |
| High SDI | Sweden | 15 to 19 | 2.19 (1.57,3.05) | 0.72 (0.3,1.71) |
| High SDI | Sweden | 20 to 24 | 2.37 (1.75,3.22) | 0.76 (0.36,1.62) |
| High SDI | Sweden | 25 to 29 | 3.89 (3.04,4.99) | 0.8 (0.41,1.56) |
| High SDI | Sweden | 30 to 34 | 5.66 (4.6,6.96) | 1.14 (0.68,1.92) |
| High SDI | Sweden | 35 to 39 | 6.76 (5.64,8.09) | 1.55 (1.02,2.36) |
| High SDI | Sweden | 40 to 44 | 8.74 (7.53,10.16) | 2.04 (1.47,2.83) |
| High SDI | Sweden | 45 to 49 | 13.02 (11.56,14.68) | 3 (2.33,3.85) |
| High SDI | Sweden | 50 to 54 | 17.99 (16.23,19.95) | 4.45 (3.62,5.46) |
| High SDI | Sweden | 55 to 59 | 23.03 (20.95,25.31) | 6.04 (5.04,7.25) |
| High SDI | Sweden | 60 to 64 | 34.26 (31.43,37.34) | 8.79 (7.45,10.36) |
| High SDI | Sweden | 65 to 69 | 44.85 (41.18,48.86) | 13.16 (11.25,15.41) |
| High SDI | Sweden | 70 to 74 | 59.5 (54.08,65.47) | 18.93 (15.95,22.46) |
| High SDI | Sweden | 75 to 79 | 67.65 (61.16,74.82) | 26.54 (22.32,31.55) |
| High SDI | Sweden | 80 to 84 | 70.5 (63.25,78.58) | 33.59 (28.14,40.09) |
| High SDI | Sweden | 85 to 89 | 90.65 (80.52,102.07) | 43.01 (35.73,51.77) |
| High SDI | Switzerland | 5 to 9 | 7.47 (5.62,9.94) | 1.01 (0.3,3.39) |
| High SDI | Switzerland | 10 to 14 | 6.76 (5.19,8.81) | 0.93 (0.31,2.77) |
| High SDI | Switzerland | 15 to 19 | 7.43 (5.85,9.44) | 1.36 (0.59,3.16) |
| High SDI | Switzerland | 20 to 24 | 8.6 (6.97,10.61) | 1.37 (0.67,2.81) |
| High SDI | Switzerland | 25 to 29 | 10.43 (8.7,12.51) | 1.4 (0.77,2.54) |
| High SDI | Switzerland | 30 to 34 | 12.62 (10.78,14.77) | 1.39 (0.83,2.31) |
| High SDI | Switzerland | 35 to 39 | 12.65 (10.93,14.63) | 1.63 (1.06,2.51) |
| High SDI | Switzerland | 40 to 44 | 13.56 (11.93,15.42) | 2.08 (1.48,2.93) |
| High SDI | Switzerland | 45 to 49 | 15.91 (14.18,17.85) | 2.72 (2.05,3.61) |
| High SDI | Switzerland | 50 to 54 | 23.03 (20.86,25.42) | 3.98 (3.15,5.04) |
| High SDI | Switzerland | 55 to 59 | 29.43 (26.85,32.26) | 5.63 (4.58,6.93) |
| High SDI | Switzerland | 60 to 64 | 41.4 (37.92,45.19) | 8.19 (6.76,9.92) |
| High SDI | Switzerland | 65 to 69 | 51.59 (47.2,56.4) | 12.2 (10.14,14.68) |
| High SDI | Switzerland | 70 to 74 | 73.5 (66.51,81.23) | 17.24 (14.06,21.13) |
| High SDI | Switzerland | 75 to 79 | 78.35 (70.36,87.25) | 24.83 (20.21,30.5) |
| High SDI | Switzerland | 80 to 84 | 98.39 (87.79,110.27) | 36.27 (29.4,44.73) |
| High SDI | Switzerland | 85 to 89 | 105.7 (92.93,120.23) | 42.23 (33.84,52.69) |
| High SDI | Taiwan (Province of China) | 5 to 9 | 0.9 (0.66,1.21) | 0.58 (0.34,0.98) |
| High SDI | Taiwan (Province of China) | 10 to 14 | 0.99 (0.76,1.28) | 0.62 (0.39,0.97) |
| High SDI | Taiwan (Province of China) | 15 to 19 | 1.21 (0.97,1.52) | 0.92 (0.64,1.31) |
| High SDI | Taiwan (Province of China) | 20 to 24 | 1.4 (1.14,1.71) | 0.96 (0.7,1.31) |
| High SDI | Taiwan (Province of China) | 25 to 29 | 2.09 (1.76,2.48) | 1.11 (0.84,1.46) |
| High SDI | Taiwan (Province of China) | 30 to 34 | 2.92 (2.52,3.38) | 1.28 (1,1.62) |
| High SDI | Taiwan (Province of China) | 35 to 39 | 3.71 (3.27,4.22) | 1.71 (1.4,2.09) |
| High SDI | Taiwan (Province of China) | 40 to 44 | 5.05 (4.53,5.64) | 2.32 (1.96,2.74) |
| High SDI | Taiwan (Province of China) | 45 to 49 | 6.96 (6.32,7.67) | 3.21 (2.78,3.71) |
| High SDI | Taiwan (Province of China) | 50 to 54 | 10.02 (9.18,10.93) | 4.64 (4.08,5.27) |
| High SDI | Taiwan (Province of China) | 55 to 59 | 13.96 (12.88,15.12) | 6.66 (5.93,7.48) |
| High SDI | Taiwan (Province of China) | 60 to 64 | 19.69 (18.26,21.24) | 9.36 (8.41,10.43) |
| High SDI | Taiwan (Province of China) | 65 to 69 | 24.77 (22.89,26.8) | 13.2 (11.86,14.69) |
| High SDI | Taiwan (Province of China) | 70 to 74 | 33.62 (30.62,36.91) | 18.58 (16.42,21.04) |
| High SDI | Taiwan (Province of China) | 75 to 79 | 42.52 (38.5,46.95) | 25.63 (22.55,29.13) |
| High SDI | Taiwan (Province of China) | 80 to 84 | 51.73 (46.38,57.7) | 34.89 (30.48,39.95) |
| High SDI | Taiwan (Province of China) | 85 to 89 | 60.42 (53.1,68.76) | 40.52 (34.74,47.26) |
| High SDI | United Arab Emirates | 5 to 9 | 0.52 (0.23,1.19) | 0.58 (0.18,1.92) |
| High SDI | United Arab Emirates | 10 to 14 | 0.82 (0.39,1.69) | 0.63 (0.21,1.88) |
| High SDI | United Arab Emirates | 15 to 19 | 1.7 (0.95,3.04) | 1.27 (0.53,3.01) |
| High SDI | United Arab Emirates | 20 to 24 | 1.75 (1.05,2.93) | 1.33 (0.64,2.74) |
| High SDI | United Arab Emirates | 25 to 29 | 2.47 (1.59,3.84) | 1.44 (0.77,2.7) |
| High SDI | United Arab Emirates | 30 to 34 | 2.41 (1.61,3.61) | 1.32 (0.75,2.33) |
| High SDI | United Arab Emirates | 35 to 39 | 3.13 (2.15,4.56) | 1.78 (1.06,2.99) |
| High SDI | United Arab Emirates | 40 to 44 | 3.63 (2.55,5.17) | 2.14 (1.34,3.43) |
| High SDI | United Arab Emirates | 45 to 49 | 5.61 (4.04,7.81) | 3.13 (2.01,4.86) |
| High SDI | United Arab Emirates | 50 to 54 | 6.22 (4.45,8.69) | 3.43 (2.19,5.36) |
| High SDI | United Arab Emirates | 55 to 59 | 9.94 (7.15,13.82) | 5.86 (3.81,9) |
| High SDI | United Arab Emirates | 60 to 64 | 17.18 (12.39,23.82) | 10.01 (6.54,15.32) |
| High SDI | United Arab Emirates | 65 to 69 | 21.94 (14.96,32.19) | 14.01 (8.69,22.59) |
| High SDI | United Arab Emirates | 70 to 74 | 54.21 (34.73,84.63) | 37.34 (21.78,64.01) |
| High SDI | United Arab Emirates | 75 to 79 | 57.18 (31.23,104.69) | 42.43 (21.33,84.39) |
| High SDI | United Arab Emirates | 80 to 84 | 64.2 (26.31,156.7) | 59.07 (24.15,144.5) |
| High SDI | United Arab Emirates | 85 to 89 | 88.24 (13.12,593.46) | 91.47 (25.45,328.81) |
| High SDI | United Kingdom | 5 to 9 | 2.54 (2.2,2.93) | 0.48 (0.31,0.74) |
| High SDI | United Kingdom | 10 to 14 | 2.57 (2.25,2.92) | 0.5 (0.34,0.72) |
| High SDI | United Kingdom | 15 to 19 | 3.22 (2.88,3.61) | 0.85 (0.64,1.12) |
| High SDI | United Kingdom | 20 to 24 | 3.4 (3.06,3.78) | 1.05 (0.83,1.33) |
| High SDI | United Kingdom | 25 to 29 | 5.95 (5.48,6.47) | 1.28 (1.05,1.54) |
| High SDI | United Kingdom | 30 to 34 | 7.66 (7.13,8.23) | 1.57 (1.34,1.85) |
| High SDI | United Kingdom | 35 to 39 | 10.06 (9.44,10.71) | 2.14 (1.88,2.44) |
| High SDI | United Kingdom | 40 to 44 | 13.29 (12.6,14.01) | 2.87 (2.59,3.19) |
| High SDI | United Kingdom | 45 to 49 | 17.89 (17.09,18.72) | 3.81 (3.5,4.16) |
| High SDI | United Kingdom | 50 to 54 | 23.84 (22.9,24.83) | 5.51 (5.12,5.92) |
| High SDI | United Kingdom | 55 to 59 | 32.25 (31.08,33.47) | 7.76 (7.27,8.28) |
| High SDI | United Kingdom | 60 to 64 | 46.13 (44.56,47.76) | 10.83 (10.2,11.5) |
| High SDI | United Kingdom | 65 to 69 | 58.23 (56.23,60.3) | 15.09 (14.24,15.99) |
| High SDI | United Kingdom | 70 to 74 | 78.98 (75.94,82.13) | 21.33 (20.02,22.72) |
| High SDI | United Kingdom | 75 to 79 | 94.05 (90.24,98.03) | 29.15 (27.33,31.09) |
| High SDI | United Kingdom | 80 to 84 | 107.65 (102.95,112.57) | 39.1 (36.59,41.77) |
| High SDI | United Kingdom | 85 to 89 | 128.75 (122.45,135.37) | 46.2 (43.04,49.6) |
| High SDI | United States of America | 5 to 9 | 4.3 (3.85,4.8) | 0.66 (0.53,0.81) |
| High SDI | United States of America | 10 to 14 | 4.4 (3.99,4.85) | 0.75 (0.64,0.89) |
| High SDI | United States of America | 15 to 19 | 5.29 (4.87,5.75) | 1.17 (1.02,1.33) |
| High SDI | United States of America | 20 to 24 | 6.99 (6.52,7.5) | 1.65 (1.49,1.83) |
| High SDI | United States of America | 25 to 29 | 10.4 (9.83,10.99) | 1.94 (1.78,2.11) |
| High SDI | United States of America | 30 to 34 | 13.57 (12.97,14.2) | 2.3 (2.15,2.46) |
| High SDI | United States of America | 35 to 39 | 14.21 (13.66,14.78) | 2.74 (2.59,2.89) |
| High SDI | United States of America | 40 to 44 | 17.06 (16.52,17.63) | 3.48 (3.33,3.63) |
| High SDI | United States of America | 45 to 49 | 22.54 (21.92,23.17) | 4.47 (4.31,4.63) |
| High SDI | United States of America | 50 to 54 | 29.35 (28.63,30.08) | 6.26 (6.07,6.45) |
| High SDI | United States of America | 55 to 59 | 38.27 (37.42,39.14) | 8.84 (8.6,9.09) |
| High SDI | United States of America | 60 to 64 | 52.32 (51.23,53.44) | 11.95 (11.65,12.26) |
| High SDI | United States of America | 65 to 69 | 62.07 (60.75,63.41) | 15.95 (15.56,16.36) |
| High SDI | United States of America | 70 to 74 | 80.93 (78.99,82.93) | 22.24 (21.63,22.86) |
| High SDI | United States of America | 75 to 79 | 90.82 (88.5,93.19) | 29.7 (28.87,30.56) |
| High SDI | United States of America | 80 to 84 | 100.17 (97.42,103) | 39.65 (38.51,40.82) |
| High SDI | United States of America | 85 to 89 | 137.07 (133,141.27) | 49.87 (48.35,51.44) |
| High SDI | United States Virgin Islands | 5 to 9 | 0.37 (0.01,23.58) | 0.72 (0,136.14) |
| High SDI | United States Virgin Islands | 10 to 14 | 0.41 (0.01,18.99) | 0.82 (0.01,114.81) |
| High SDI | United States Virgin Islands | 15 to 19 | 0.57 (0.02,20.89) | 1.05 (0.01,113.7) |
| High SDI | United States Virgin Islands | 20 to 24 | 0.85 (0.03,25.52) | 1.45 (0.02,123.24) |
| High SDI | United States Virgin Islands | 25 to 29 | 6.63 (0.49,89.5) | 1.6 (0.02,105.76) |
| High SDI | United States Virgin Islands | 30 to 34 | 9.07 (0.96,85.64) | 1.65 (0.03,84.18) |
| High SDI | United States Virgin Islands | 35 to 39 | 6.85 (1.1,42.48) | 1.58 (0.04,56.82) |
| High SDI | United States Virgin Islands | 40 to 44 | 4.43 (0.58,33.99) | 1.54 (0.06,38.78) |
| High SDI | United States Virgin Islands | 45 to 49 | 12.42 (3.71,41.59) | 1.55 (0.08,31.14) |
| High SDI | United States Virgin Islands | 50 to 54 | 12.49 (3.94,39.6) | 1.58 (0.09,27.29) |
| High SDI | United States Virgin Islands | 55 to 59 | 12.72 (4.08,39.71) | 12.38 (3.14,48.85) |
| High SDI | United States Virgin Islands | 60 to 64 | 14.6 (4.59,46.43) | 14.53 (3.72,56.86) |
| High SDI | United States Virgin Islands | 65 to 69 | 15.86 (4.79,52.45) | 20.15 (4.8,84.55) |
| High SDI | United States Virgin Islands | 70 to 74 | 18.85 (4.84,73.45) | 36.26 (6.18,212.62) |
| High SDI | United States Virgin Islands | 75 to 79 | 25.3 (5.89,108.68) | 22.17 (2.09,235.3) |
| High SDI | United States Virgin Islands | 80 to 84 | 17.48 (2.2,139.13) | 14.63 (0.48,449.63) |
| High SDI | United States Virgin Islands | 85 to 89 | 10.54 (0.39,286.81) | 25.93 (0.62,1076.06) |
| High-middle SDI | Antigua and Barbuda | 5 to 9 | 1.16 (0.01,196.51) | - |
| High-middle SDI | Antigua and Barbuda | 10 to 14 | 1.13 (0.01,140.08) | - |
| High-middle SDI | Antigua and Barbuda | 15 to 19 | 1.14 (0.01,109.39) | - |
| High-middle SDI | Antigua and Barbuda | 20 to 24 | 1.16 (0.02,86.7) | - |
| High-middle SDI | Antigua and Barbuda | 25 to 29 | 1.03 (0.02,56.99) | - |
| High-middle SDI | Antigua and Barbuda | 30 to 34 | 1.03 (0.02,43.79) | - |
| High-middle SDI | Antigua and Barbuda | 35 to 39 | 1.1 (0.03,35.88) | - |
| High-middle SDI | Antigua and Barbuda | 40 to 44 | 1.28 (0.05,30.07) | - |
| High-middle SDI | Antigua and Barbuda | 45 to 49 | 1.52 (0.08,29.84) | - |
| High-middle SDI | Antigua and Barbuda | 50 to 54 | 4.69 (0.32,68.22) | - |
| High-middle SDI | Antigua and Barbuda | 55 to 59 | 13.62 (1.42,130.66) | - |
| High-middle SDI | Antigua and Barbuda | 60 to 64 | 25.47 (5.4,120.11) | - |
| High-middle SDI | Antigua and Barbuda | 65 to 69 | 28.6 (5.14,159.02) | - |
| High-middle SDI | Antigua and Barbuda | 70 to 74 | 45.62 (4.41,472.2) | - |
| High-middle SDI | Antigua and Barbuda | 75 to 79 | 67.18 (3.78,1192.4) | - |
| High-middle SDI | Antigua and Barbuda | 80 to 84 | 75.77 (1.87,3077.87) | - |
| High-middle SDI | Antigua and Barbuda | 85 to 89 | 152.91 (2.71,8621.86) | - |
| High-middle SDI | Argentina | 5 to 9 | 1.09 (0.89,1.33) | 0.72 (0.52,1) |
| High-middle SDI | Argentina | 10 to 14 | 1.13 (0.94,1.36) | 0.67 (0.49,0.91) |
| High-middle SDI | Argentina | 15 to 19 | 1.69 (1.44,1.98) | 1.1 (0.86,1.41) |
| High-middle SDI | Argentina | 20 to 24 | 2.12 (1.84,2.45) | 1.36 (1.09,1.7) |
| High-middle SDI | Argentina | 25 to 29 | 3.45 (3.05,3.91) | 1.68 (1.38,2.04) |
| High-middle SDI | Argentina | 30 to 34 | 4.54 (4.06,5.07) | 2.01 (1.69,2.39) |
| High-middle SDI | Argentina | 35 to 39 | 4.98 (4.5,5.52) | 2.43 (2.08,2.83) |
| High-middle SDI | Argentina | 40 to 44 | 6.68 (6.12,7.3) | 3.36 (2.96,3.82) |
| High-middle SDI | Argentina | 45 to 49 | 8.8 (8.14,9.51) | 4.43 (3.97,4.95) |
| High-middle SDI | Argentina | 50 to 54 | 11.66 (10.87,12.51) | 6.3 (5.72,6.93) |
| High-middle SDI | Argentina | 55 to 59 | 16.37 (15.34,17.46) | 9.33 (8.56,10.16) |
| High-middle SDI | Argentina | 60 to 64 | 22.53 (21.17,23.97) | 12.74 (11.75,13.83) |
| High-middle SDI | Argentina | 65 to 69 | 28.65 (26.9,30.52) | 17.82 (16.45,19.32) |
| High-middle SDI | Argentina | 70 to 74 | 36.11 (33.56,38.85) | 23.75 (21.67,26.02) |
| High-middle SDI | Argentina | 75 to 79 | 38.36 (35.39,41.57) | 28.33 (25.72,31.2) |
| High-middle SDI | Argentina | 80 to 84 | 41.82 (38.21,45.76) | 34.54 (31.14,38.32) |
| High-middle SDI | Argentina | 85 to 89 | 45.47 (40.83,50.63) | 38.31 (34.04,43.12) |
| High-middle SDI | Bahamas | 5 to 9 | 1.54 (0.2,11.88) | 0.46 (0.01,16.98) |
| High-middle SDI | Bahamas | 10 to 14 | 0.25 (0.01,4.87) | 0.45 (0.02,11.62) |
| High-middle SDI | Bahamas | 15 to 19 | 1.92 (0.33,11) | 0.51 (0.02,10.58) |
| High-middle SDI | Bahamas | 20 to 24 | 2.37 (0.61,9.2) | 2.3 (0.22,23.71) |
| High-middle SDI | Bahamas | 25 to 29 | 2.48 (0.69,8.84) | 3.6 (0.68,18.97) |
| High-middle SDI | Bahamas | 30 to 34 | 4.85 (1.62,14.51) | 6.03 (1.51,24.08) |
| High-middle SDI | Bahamas | 35 to 39 | 4.77 (1.66,13.73) | 5.56 (1.52,20.25) |
| High-middle SDI | Bahamas | 40 to 44 | 5 (1.88,13.33) | 5.55 (1.77,17.43) |
| High-middle SDI | Bahamas | 45 to 49 | 8.22 (3.37,20.04) | 5.9 (1.98,17.65) |
| High-middle SDI | Bahamas | 50 to 54 | 10.84 (4.8,24.5) | 6.75 (2.31,19.66) |
| High-middle SDI | Bahamas | 55 to 59 | 14.92 (6.72,33.17) | 10.31 (3.73,28.5) |
| High-middle SDI | Bahamas | 60 to 64 | 18.73 (8.25,42.52) | 10.56 (3.67,30.34) |
| High-middle SDI | Bahamas | 65 to 69 | 26.7 (11.82,60.31) | 13.42 (4.88,36.89) |
| High-middle SDI | Bahamas | 70 to 74 | 31.65 (11.37,88.05) | 17.18 (5.1,57.89) |
| High-middle SDI | Bahamas | 75 to 79 | 41.33 (13.55,126.05) | 21.22 (5.52,81.56) |
| High-middle SDI | Bahamas | 80 to 84 | 48.17 (12.96,179.04) | 27.67 (6.43,119.1) |
| High-middle SDI | Bahamas | 85 to 89 | 11.63 (0.52,259.4) | 7.1 (0.3,168.67) |
| High-middle SDI | Bahrain | 5 to 9 | 0.92 (0.22,3.73) | 0.48 (0.01,15.86) |
| High-middle SDI | Bahrain | 10 to 14 | 0.83 (0.21,3.33) | 0.45 (0.02,10.54) |
| High-middle SDI | Bahrain | 15 to 19 | 1.21 (0.37,3.98) | 0.39 (0.02,7.47) |
| High-middle SDI | Bahrain | 20 to 24 | 1.36 (0.46,3.99) | 2.39 (0.42,13.58) |
| High-middle SDI | Bahrain | 25 to 29 | 2.08 (0.81,5.36) | 2.09 (0.54,8.05) |
| High-middle SDI | Bahrain | 30 to 34 | 2.58 (1.1,6.1) | 1.7 (0.48,5.98) |
| High-middle SDI | Bahrain | 35 to 39 | 3.03 (1.35,6.8) | 2.19 (0.69,6.94) |
| High-middle SDI | Bahrain | 40 to 44 | 3.51 (1.63,7.55) | 2.06 (0.72,5.9) |
| High-middle SDI | Bahrain | 45 to 49 | 5.06 (2.5,10.25) | 3.23 (1.22,8.54) |
| High-middle SDI | Bahrain | 50 to 54 | 6.36 (3.19,12.7) | 3.9 (1.54,9.84) |
| High-middle SDI | Bahrain | 55 to 59 | 9.54 (5,18.22) | 5.7 (2.44,13.28) |
| High-middle SDI | Bahrain | 60 to 64 | 14.04 (7.44,26.49) | 7.05 (2.96,16.8) |
| High-middle SDI | Bahrain | 65 to 69 | 20.14 (10.49,38.65) | 12.7 (5.56,28.98) |
| High-middle SDI | Bahrain | 70 to 74 | 28.55 (12.71,64.12) | 18.75 (6.97,50.43) |
| High-middle SDI | Bahrain | 75 to 79 | 32.61 (12.76,83.32) | 26.27 (8.97,76.95) |
| High-middle SDI | Bahrain | 80 to 84 | 45 (14.76,137.19) | 30.31 (8.24,111.58) |
| High-middle SDI | Bahrain | 85 to 89 | 43.06 (6.55,283.11) | 30.89 (4.28,222.76) |
| High-middle SDI | Barbados | 5 to 9 | 0.46 (0.02,11.2) | 1.31 (0.03,56.96) |
| High-middle SDI | Barbados | 10 to 14 | 1.47 (0.15,14.94) | 1.16 (0.04,34.18) |
| High-middle SDI | Barbados | 15 to 19 | 4.18 (1.12,15.53) | 1.1 (0.05,24.83) |
| High-middle SDI | Barbados | 20 to 24 | 4.59 (1.36,15.52) | 2.68 (0.36,20.16) |
| High-middle SDI | Barbados | 25 to 29 | 4.57 (1.46,14.29) | 3.83 (0.75,19.6) |
| High-middle SDI | Barbados | 30 to 34 | 8.99 (3.6,22.44) | 5.92 (1.53,22.99) |
| High-middle SDI | Barbados | 35 to 39 | 8.91 (3.8,20.89) | 6.12 (1.83,20.44) |
| High-middle SDI | Barbados | 40 to 44 | 8.83 (3.92,19.89) | 6.02 (2.05,17.69) |
| High-middle SDI | Barbados | 45 to 49 | 13.42 (6.76,26.64) | 5.92 (2.15,16.34) |
| High-middle SDI | Barbados | 50 to 54 | 17.16 (9.15,32.19) | 8.95 (3.5,22.87) |
| High-middle SDI | Barbados | 55 to 59 | 23.49 (13.15,41.97) | 11.52 (4.99,26.63) |
| High-middle SDI | Barbados | 60 to 64 | 36.29 (21.29,61.87) | 18.55 (8.77,39.25) |
| High-middle SDI | Barbados | 65 to 69 | 39.3 (22.37,69.04) | 23.72 (11.39,49.4) |
| High-middle SDI | Barbados | 70 to 74 | 56.34 (29.42,107.88) | 35.87 (15.16,84.91) |
| High-middle SDI | Barbados | 75 to 79 | 62.82 (30.87,127.86) | 41.83 (16.75,104.47) |
| High-middle SDI | Barbados | 80 to 84 | 63.56 (28.63,141.07) | 50.62 (19.02,134.75) |
| High-middle SDI | Barbados | 85 to 89 | 81.24 (32.36,203.95) | 55.64 (18.05,171.55) |
| High-middle SDI | Belarus | 5 to 9 | 0.26 (0.17,0.4) | 0.12 (0.04,0.35) |
| High-middle SDI | Belarus | 10 to 14 | 0.26 (0.17,0.39) | 0.09 (0.03,0.24) |
| High-middle SDI | Belarus | 15 to 19 | 0.27 (0.18,0.4) | 0.16 (0.08,0.36) |
| High-middle SDI | Belarus | 20 to 24 | 0.38 (0.27,0.54) | 0.25 (0.13,0.48) |
| High-middle SDI | Belarus | 25 to 29 | 0.75 (0.56,1) | 0.37 (0.22,0.64) |
| High-middle SDI | Belarus | 30 to 34 | 1.2 (0.93,1.54) | 0.51 (0.32,0.8) |
| High-middle SDI | Belarus | 35 to 39 | 1.71 (1.37,2.13) | 0.77 (0.53,1.13) |
| High-middle SDI | Belarus | 40 to 44 | 2.58 (2.13,3.13) | 1.14 (0.83,1.58) |
| High-middle SDI | Belarus | 45 to 49 | 4.6 (3.9,5.42) | 1.86 (1.42,2.43) |
| High-middle SDI | Belarus | 50 to 54 | 7.96 (6.9,9.17) | 3.19 (2.54,4.01) |
| High-middle SDI | Belarus | 55 to 59 | 12.97 (11.44,14.72) | 5.3 (4.36,6.45) |
| High-middle SDI | Belarus | 60 to 64 | 22.32 (19.89,25.06) | 8.38 (6.98,10.06) |
| High-middle SDI | Belarus | 65 to 69 | 33.03 (29.44,37.05) | 12.83 (10.73,15.34) |
| High-middle SDI | Belarus | 70 to 74 | 47.09 (40.81,54.34) | 18.33 (14.83,22.65) |
| High-middle SDI | Belarus | 75 to 79 | 51.8 (44.08,60.87) | 22.1 (17.6,27.74) |
| High-middle SDI | Belarus | 80 to 84 | 58.92 (48.75,71.21) | 27.62 (21.47,35.54) |
| High-middle SDI | Belarus | 85 to 89 | 51.2 (39.04,67.16) | 22.46 (16.01,31.51) |
| High-middle SDI | Brunei Darussalam | 5 to 9 | 0.37 (0.01,11.42) | 0.48 (0.01,25.1) |
| High-middle SDI | Brunei Darussalam | 10 to 14 | 1.31 (0.13,13.42) | 0.48 (0.01,17.14) |
| High-middle SDI | Brunei Darussalam | 15 to 19 | 0.31 (0.02,5.6) | 0.49 (0.02,13.02) |
| High-middle SDI | Brunei Darussalam | 20 to 24 | 2.08 (0.41,10.57) | 0.45 (0.02,9.77) |
| High-middle SDI | Brunei Darussalam | 25 to 29 | 2.3 (0.62,8.47) | 0.4 (0.02,7.43) |
| High-middle SDI | Brunei Darussalam | 30 to 34 | 2.45 (0.72,8.36) | 2.66 (0.45,15.85) |
| High-middle SDI | Brunei Darussalam | 35 to 39 | 3.5 (1.13,10.84) | 3.98 (1.01,15.72) |
| High-middle SDI | Brunei Darussalam | 40 to 44 | 4.05 (1.46,11.17) | 4.19 (1.05,16.79) |
| High-middle SDI | Brunei Darussalam | 45 to 49 | 6.69 (2.62,17.13) | 4.76 (1.42,15.97) |
| High-middle SDI | Brunei Darussalam | 50 to 54 | 9.64 (3.93,23.69) | 7.03 (2.28,21.62) |
| High-middle SDI | Brunei Darussalam | 55 to 59 | 15.1 (6.65,34.29) | 11.19 (4.03,31.07) |
| High-middle SDI | Brunei Darussalam | 60 to 64 | 24.35 (11.25,52.69) | 15.22 (5.89,39.31) |
| High-middle SDI | Brunei Darussalam | 65 to 69 | 34.45 (15.23,77.94) | 21.19 (7.85,57.21) |
| High-middle SDI | Brunei Darussalam | 70 to 74 | 46 (16.96,124.79) | 29.51 (9.02,96.59) |
| High-middle SDI | Brunei Darussalam | 75 to 79 | 53.63 (17.62,163.22) | 42.01 (12.13,145.52) |
| High-middle SDI | Brunei Darussalam | 80 to 84 | 67.35 (18.28,248.1) | 63.07 (16.52,240.8) |
| High-middle SDI | Brunei Darussalam | 85 to 89 | 126.51 (23.92,669) | 98.52 (20.2,480.38) |
| High-middle SDI | Bulgaria | 5 to 9 | 0.71 (0.44,1.16) | 0.37 (0.14,0.94) |
| High-middle SDI | Bulgaria | 10 to 14 | 0.68 (0.43,1.06) | 0.35 (0.15,0.82) |
| High-middle SDI | Bulgaria | 15 to 19 | 0.7 (0.46,1.06) | 0.49 (0.24,1) |
| High-middle SDI | Bulgaria | 20 to 24 | 0.74 (0.51,1.09) | 0.57 (0.31,1.06) |
| High-middle SDI | Bulgaria | 25 to 29 | 1.2 (0.87,1.66) | 0.72 (0.42,1.23) |
| High-middle SDI | Bulgaria | 30 to 34 | 1.67 (1.26,2.22) | 0.77 (0.47,1.26) |
| High-middle SDI | Bulgaria | 35 to 39 | 2.58 (2.04,3.27) | 1.09 (0.72,1.64) |
| High-middle SDI | Bulgaria | 40 to 44 | 3.86 (3.17,4.7) | 1.5 (1.07,2.11) |
| High-middle SDI | Bulgaria | 45 to 49 | 6.83 (5.81,8.02) | 2.35 (1.78,3.1) |
| High-middle SDI | Bulgaria | 50 to 54 | 10.82 (9.43,12.42) | 3.77 (2.99,4.76) |
| High-middle SDI | Bulgaria | 55 to 59 | 16.92 (14.98,19.12) | 5.93 (4.84,7.25) |
| High-middle SDI | Bulgaria | 60 to 64 | 23.96 (21.35,26.9) | 8.74 (7.25,10.55) |
| High-middle SDI | Bulgaria | 65 to 69 | 31.99 (28.43,36) | 13.2 (10.98,15.86) |
| High-middle SDI | Bulgaria | 70 to 74 | 40.97 (35.59,47.18) | 17.74 (14.32,21.98) |
| High-middle SDI | Bulgaria | 75 to 79 | 41.68 (35.52,48.92) | 23.93 (19.08,30.01) |
| High-middle SDI | Bulgaria | 80 to 84 | 42.04 (34.71,50.91) | 29.81 (23.25,38.22) |
| High-middle SDI | Bulgaria | 85 to 89 | 54.61 (42.63,69.96) | 39.88 (29.56,53.8) |
| High-middle SDI | Chile | 5 to 9 | 0.54 (0.37,0.79) | 0.62 (0.34,1.14) |
| High-middle SDI | Chile | 10 to 14 | 0.47 (0.33,0.68) | 0.51 (0.29,0.92) |
| High-middle SDI | Chile | 15 to 19 | 0.72 (0.53,0.99) | 0.78 (0.49,1.24) |
| High-middle SDI | Chile | 20 to 24 | 1.02 (0.78,1.33) | 1.05 (0.71,1.57) |
| High-middle SDI | Chile | 25 to 29 | 1.76 (1.4,2.2) | 1.33 (0.94,1.88) |
| High-middle SDI | Chile | 30 to 34 | 2.48 (2.04,3.02) | 1.54 (1.14,2.09) |
| High-middle SDI | Chile | 35 to 39 | 3.1 (2.6,3.7) | 1.89 (1.45,2.46) |
| High-middle SDI | Chile | 40 to 44 | 4.33 (3.71,5.04) | 2.43 (1.94,3.04) |
| High-middle SDI | Chile | 45 to 49 | 6.08 (5.31,6.96) | 3.04 (2.49,3.71) |
| High-middle SDI | Chile | 50 to 54 | 9.13 (8.11,10.29) | 4.4 (3.7,5.23) |
| High-middle SDI | Chile | 55 to 59 | 13.45 (12.07,14.99) | 6.4 (5.47,7.49) |
| High-middle SDI | Chile | 60 to 64 | 20.48 (18.51,22.67) | 9.03 (7.79,10.46) |
| High-middle SDI | Chile | 65 to 69 | 27.99 (25.25,31.03) | 12.95 (11.19,14.97) |
| High-middle SDI | Chile | 70 to 74 | 38.61 (34.21,43.57) | 17.98 (15.25,21.21) |
| High-middle SDI | Chile | 75 to 79 | 45.3 (39.67,51.74) | 23.35 (19.62,27.78) |
| High-middle SDI | Chile | 80 to 84 | 49.21 (42.34,57.19) | 27.78 (23.04,33.5) |
| High-middle SDI | Chile | 85 to 89 | 56.69 (47.52,67.62) | 30.94 (25.05,38.21) |
| High-middle SDI | Croatia | 5 to 9 | 1.21 (0.71,2.09) | 0.5 (0.15,1.73) |
| High-middle SDI | Croatia | 10 to 14 | 1.06 (0.64,1.78) | 0.49 (0.16,1.53) |
| High-middle SDI | Croatia | 15 to 19 | 1.7 (1.1,2.63) | 0.74 (0.3,1.85) |
| High-middle SDI | Croatia | 20 to 24 | 1.65 (1.08,2.5) | 0.94 (0.42,2.12) |
| High-middle SDI | Croatia | 25 to 29 | 2.39 (1.66,3.44) | 1.05 (0.52,2.12) |
| High-middle SDI | Croatia | 30 to 34 | 2.83 (2.04,3.9) | 1.04 (0.55,1.95) |
| High-middle SDI | Croatia | 35 to 39 | 3.6 (2.72,4.78) | 1.36 (0.81,2.29) |
| High-middle SDI | Croatia | 40 to 44 | 5.44 (4.34,6.83) | 1.92 (1.27,2.88) |
| High-middle SDI | Croatia | 45 to 49 | 8.3 (6.83,10.09) | 2.94 (2.1,4.11) |
| High-middle SDI | Croatia | 50 to 54 | 12.35 (10.45,14.6) | 4.59 (3.48,6.05) |
| High-middle SDI | Croatia | 55 to 59 | 18.62 (16.05,21.59) | 6.97 (5.47,8.87) |
| High-middle SDI | Croatia | 60 to 64 | 29.55 (25.8,33.85) | 10.46 (8.38,13.05) |
| High-middle SDI | Croatia | 65 to 69 | 42.73 (37.3,48.96) | 16.41 (13.24,20.34) |
| High-middle SDI | Croatia | 70 to 74 | 63.7 (54.43,74.55) | 24.59 (19.32,31.3) |
| High-middle SDI | Croatia | 75 to 79 | 80.35 (67.96,94.99) | 34.43 (26.86,44.12) |
| High-middle SDI | Croatia | 80 to 84 | 101.72 (84.52,122.43) | 46.23 (35.5,60.19) |
| High-middle SDI | Croatia | 85 to 89 | 146.76 (117.95,182.6) | 52.79 (38.74,71.94) |
| High-middle SDI | Dominica | 5 to 9 | 0.74 (0,110.26) | 1.11 (0.01,205.27) |
| High-middle SDI | Dominica | 10 to 14 | 0.77 (0.01,83.08) | 1.09 (0.01,149.29) |
| High-middle SDI | Dominica | 15 to 19 | 0.87 (0.01,72.08) | 1.19 (0.01,126.69) |
| High-middle SDI | Dominica | 20 to 24 | 1.09 (0.02,70.71) | 1.46 (0.02,120.71) |
| High-middle SDI | Dominica | 25 to 29 | 1.1 (0.02,51.68) | 1.65 (0.03,106.62) |
| High-middle SDI | Dominica | 30 to 34 | 1.15 (0.03,40.04) | 1.58 (0.03,76.19) |
| High-middle SDI | Dominica | 35 to 39 | 1.29 (0.05,34.77) | 1.73 (0.05,61.71) |
| High-middle SDI | Dominica | 40 to 44 | 1.54 (0.08,31.29) | 1.95 (0.08,48.96) |
| High-middle SDI | Dominica | 45 to 49 | 5.04 (0.43,59.45) | 2.45 (0.12,50.21) |
| High-middle SDI | Dominica | 50 to 54 | 12.27 (1.85,81.51) | 3.28 (0.18,60.25) |
| High-middle SDI | Dominica | 55 to 59 | 19.19 (4.26,86.32) | 9.72 (0.71,132.33) |
| High-middle SDI | Dominica | 60 to 64 | 40.79 (10.84,153.48) | 24.01 (4.46,129.24) |
| High-middle SDI | Dominica | 65 to 69 | 50.21 (12.47,202.21) | 47.62 (8.61,263.49) |
| High-middle SDI | Dominica | 70 to 74 | 61.11 (12.32,303.08) | 114.02 (14.87,874.11) |
| High-middle SDI | Dominica | 75 to 79 | 77.5 (14.65,409.85) | 137.02 (14.43,1300.91) |
| High-middle SDI | Dominica | 80 to 84 | 11.59 (0.55,246.31) | 23.72 (0.84,669.8) |
| High-middle SDI | Dominica | 85 to 89 | 22.45 (0.8,630.37) | 48.51 (1.34,1757.53) |
| High-middle SDI | Georgia | 5 to 9 | 2.41 (1.37,4.23) | 0.7 (0.27,1.82) |
| High-middle SDI | Georgia | 10 to 14 | 1.98 (1.16,3.37) | 0.64 (0.26,1.57) |
| High-middle SDI | Georgia | 15 to 19 | 1.87 (1.14,3.06) | 0.75 (0.34,1.63) |
| High-middle SDI | Georgia | 20 to 24 | 1.84 (1.16,2.91) | 0.87 (0.44,1.73) |
| High-middle SDI | Georgia | 25 to 29 | 2.46 (1.66,3.66) | 0.92 (0.49,1.72) |
| High-middle SDI | Georgia | 30 to 34 | 2.88 (2.01,4.11) | 1.05 (0.6,1.84) |
| High-middle SDI | Georgia | 35 to 39 | 3 (2.14,4.19) | 1.19 (0.72,1.97) |
| High-middle SDI | Georgia | 40 to 44 | 3.62 (2.7,4.86) | 1.6 (1.04,2.48) |
| High-middle SDI | Georgia | 45 to 49 | 5.05 (3.84,6.63) | 2.28 (1.54,3.39) |
| High-middle SDI | Georgia | 50 to 54 | 7.01 (5.43,9.04) | 3.4 (2.37,4.86) |
| High-middle SDI | Georgia | 55 to 59 | 9.9 (7.8,12.55) | 5.24 (3.79,7.25) |
| High-middle SDI | Georgia | 60 to 64 | 14.44 (11.63,17.92) | 7.94 (5.95,10.61) |
| High-middle SDI | Georgia | 65 to 69 | 19.7 (15.66,24.78) | 12.51 (9.3,16.84) |
| High-middle SDI | Georgia | 70 to 74 | 26.83 (20.33,35.42) | 18.57 (13.05,26.43) |
| High-middle SDI | Georgia | 75 to 79 | 29.08 (21.3,39.7) | 24.01 (16.46,35.02) |
| High-middle SDI | Georgia | 80 to 84 | 26.23 (17.78,38.7) | 24.91 (16.03,38.69) |
| High-middle SDI | Georgia | 85 to 89 | 18.71 (10.09,34.72) | 18.33 (9.55,35.19) |
| High-middle SDI | Greece | 5 to 9 | 1.33 (0.89,1.98) | 0.24 (0.07,0.83) |
| High-middle SDI | Greece | 10 to 14 | 1.31 (0.93,1.86) | 0.2 (0.07,0.6) |
| High-middle SDI | Greece | 15 to 19 | 1.77 (1.33,2.36) | 0.27 (0.12,0.63) |
| High-middle SDI | Greece | 20 to 24 | 2.3 (1.8,2.94) | 0.38 (0.19,0.76) |
| High-middle SDI | Greece | 25 to 29 | 3.7 (3.02,4.53) | 0.46 (0.25,0.82) |
| High-middle SDI | Greece | 30 to 34 | 4.7 (3.93,5.62) | 0.54 (0.32,0.9) |
| High-middle SDI | Greece | 35 to 39 | 4.96 (4.2,5.85) | 0.74 (0.48,1.14) |
| High-middle SDI | Greece | 40 to 44 | 6.53 (5.65,7.55) | 1.07 (0.75,1.53) |
| High-middle SDI | Greece | 45 to 49 | 9.13 (8.05,10.36) | 1.56 (1.16,2.11) |
| High-middle SDI | Greece | 50 to 54 | 12.1 (10.78,13.6) | 2.47 (1.91,3.19) |
| High-middle SDI | Greece | 55 to 59 | 15.91 (14.26,17.74) | 3.73 (2.98,4.67) |
| High-middle SDI | Greece | 60 to 64 | 23.91 (21.63,26.43) | 5.78 (4.73,7.07) |
| High-middle SDI | Greece | 65 to 69 | 32.27 (29.24,35.61) | 9.36 (7.76,11.3) |
| High-middle SDI | Greece | 70 to 74 | 44.72 (39.95,50.05) | 14.41 (11.65,17.81) |
| High-middle SDI | Greece | 75 to 79 | 52.16 (46.31,58.74) | 21.52 (17.36,26.66) |
| High-middle SDI | Greece | 80 to 84 | 59.98 (52.84,68.09) | 30.57 (24.57,38.05) |
| High-middle SDI | Greece | 85 to 89 | 76.72 (66.59,88.39) | 38.63 (30.59,48.78) |
| High-middle SDI | Guam | 5 to 9 | 0.23 (0.01,7.73) | 0.51 (0,110.88) |
| High-middle SDI | Guam | 10 to 14 | 2.49 (0.26,23.56) | 0.49 (0,79.67) |
| High-middle SDI | Guam | 15 to 19 | 2.79 (0.32,24.32) | 0.53 (0,67.66) |
| High-middle SDI | Guam | 20 to 24 | 2.45 (0.27,22.24) | 0.56 (0.01,56.54) |
| High-middle SDI | Guam | 25 to 29 | 3.21 (0.42,24.76) | 0.63 (0.01,49.59) |
| High-middle SDI | Guam | 30 to 34 | 3.4 (0.43,26.58) | 0.64 (0.01,37.93) |
| High-middle SDI | Guam | 35 to 39 | 4.08 (0.64,26.16) | 0.66 (0.01,30.67) |
| High-middle SDI | Guam | 40 to 44 | 3.19 (0.4,25.14) | 0.76 (0.02,23.55) |
| High-middle SDI | Guam | 45 to 49 | 4.66 (0.74,29.12) | 0.91 (0.03,24.12) |
| High-middle SDI | Guam | 50 to 54 | 4.14 (0.57,29.9) | 1.29 (0.05,31.8) |
| High-middle SDI | Guam | 55 to 59 | 7.62 (1.72,33.78) | 3.56 (0.18,68.74) |
| High-middle SDI | Guam | 60 to 64 | 9.29 (2.05,42.17) | 5.46 (0.28,104.99) |
| High-middle SDI | Guam | 65 to 69 | 25.16 (4.97,127.45) | 8.69 (1.22,62.09) |
| High-middle SDI | Guam | 70 to 74 | 55.82 (7.78,400.22) | 15 (0.61,371.08) |
| High-middle SDI | Guam | 75 to 79 | 66.37 (6.59,668.68) | 13.34 (0.29,606.29) |
| High-middle SDI | Guam | 80 to 84 | 25.41 (0.81,798.17) | 19.47 (0.34,1115.96) |
| High-middle SDI | Guam | 85 to 89 | 42.62 (0.99,1840.13) | 29.24 (0.37,2298.64) |
| High-middle SDI | Hungary | 5 to 9 | 1.11 (0.73,1.68) | 0.54 (0.21,1.37) |
| High-middle SDI | Hungary | 10 to 14 | 1.19 (0.82,1.73) | 0.7 (0.33,1.49) |
| High-middle SDI | Hungary | 15 to 19 | 1.39 (1,1.93) | 0.75 (0.39,1.43) |
| High-middle SDI | Hungary | 20 to 24 | 1.72 (1.29,2.31) | 1.01 (0.59,1.73) |
| High-middle SDI | Hungary | 25 to 29 | 2.98 (2.36,3.78) | 1.24 (0.78,1.96) |
| High-middle SDI | Hungary | 30 to 34 | 4.14 (3.39,5.05) | 1.44 (0.97,2.14) |
| High-middle SDI | Hungary | 35 to 39 | 5.68 (4.81,6.71) | 2.06 (1.51,2.8) |
| High-middle SDI | Hungary | 40 to 44 | 7.54 (6.59,8.64) | 2.75 (2.17,3.49) |
| High-middle SDI | Hungary | 45 to 49 | 11.62 (10.38,13.01) | 4.01 (3.29,4.88) |
| High-middle SDI | Hungary | 50 to 54 | 16.7 (15.15,18.41) | 5.83 (4.94,6.88) |
| High-middle SDI | Hungary | 55 to 59 | 22.29 (20.39,24.38) | 8.16 (7.05,9.45) |
| High-middle SDI | Hungary | 60 to 64 | 31.63 (29.05,34.44) | 11.19 (9.74,12.86) |
| High-middle SDI | Hungary | 65 to 69 | 40.63 (37.24,44.33) | 15.93 (13.89,18.27) |
| High-middle SDI | Hungary | 70 to 74 | 51.27 (46.27,56.81) | 20.95 (17.91,24.49) |
| High-middle SDI | Hungary | 75 to 79 | 56.45 (50.46,63.16) | 27.09 (23.01,31.88) |
| High-middle SDI | Hungary | 80 to 84 | 57.46 (50.47,65.42) | 31.7 (26.57,37.82) |
| High-middle SDI | Hungary | 85 to 89 | 64.81 (55.23,76.05) | 33.45 (27.17,41.18) |
| High-middle SDI | Israel | 5 to 9 | 2.93 (2.18,3.95) | 0.9 (0.38,2.13) |
| High-middle SDI | Israel | 10 to 14 | 2.84 (2.15,3.76) | 0.79 (0.36,1.76) |
| High-middle SDI | Israel | 15 to 19 | 3.49 (2.71,4.49) | 1.25 (0.66,2.4) |
| High-middle SDI | Israel | 20 to 24 | 4.24 (3.36,5.34) | 1.48 (0.84,2.61) |
| High-middle SDI | Israel | 25 to 29 | 6.54 (5.33,8.01) | 1.58 (0.94,2.64) |
| High-middle SDI | Israel | 30 to 34 | 8.62 (7.19,10.33) | 1.85 (1.18,2.9) |
| High-middle SDI | Israel | 35 to 39 | 9.59 (8.12,11.31) | 2.3 (1.57,3.38) |
| High-middle SDI | Israel | 40 to 44 | 12.36 (10.71,14.26) | 3.04 (2.23,4.15) |
| High-middle SDI | Israel | 45 to 49 | 17.88 (15.81,20.22) | 4.32 (3.34,5.59) |
| High-middle SDI | Israel | 50 to 54 | 25.08 (22.46,28.01) | 6.56 (5.27,8.17) |
| High-middle SDI | Israel | 55 to 59 | 35.96 (32.57,39.7) | 10.03 (8.31,12.12) |
| High-middle SDI | Israel | 60 to 64 | 55.77 (50.96,61.04) | 14.68 (12.38,17.41) |
| High-middle SDI | Israel | 65 to 69 | 73.13 (66.77,80.11) | 21.66 (18.4,25.48) |
| High-middle SDI | Israel | 70 to 74 | 103.89 (93.28,115.71) | 31.7 (26.31,38.19) |
| High-middle SDI | Israel | 75 to 79 | 125.75 (111.93,141.28) | 45.06 (37.21,54.57) |
| High-middle SDI | Israel | 80 to 84 | 149.81 (132.15,169.84) | 62.27 (51.14,75.82) |
| High-middle SDI | Israel | 85 to 89 | 186.38 (161.81,214.68) | 73.61 (59.52,91.02) |
| High-middle SDI | Italy | 5 to 9 | 6.4 (5.71,7.17) | 1.02 (0.69,1.52) |
| High-middle SDI | Italy | 10 to 14 | 7.66 (6.95,8.44) | 1.14 (0.82,1.59) |
| High-middle SDI | Italy | 15 to 19 | 9.85 (9.07,10.68) | 1.54 (1.19,2) |
| High-middle SDI | Italy | 20 to 24 | 9.2 (8.53,9.91) | 1.73 (1.39,2.14) |
| High-middle SDI | Italy | 25 to 29 | 14.48 (13.64,15.37) | 1.9 (1.59,2.26) |
| High-middle SDI | Italy | 30 to 34 | 15.14 (14.36,15.97) | 1.94 (1.66,2.26) |
| High-middle SDI | Italy | 35 to 39 | 15.22 (14.49,15.99) | 2.17 (1.91,2.48) |
| High-middle SDI | Italy | 40 to 44 | 16.98 (16.26,17.74) | 2.74 (2.46,3.05) |
| High-middle SDI | Italy | 45 to 49 | 24.37 (23.5,25.27) | 3.63 (3.32,3.96) |
| High-middle SDI | Italy | 50 to 54 | 30.24 (29.26,31.25) | 4.94 (4.58,5.33) |
| High-middle SDI | Italy | 55 to 59 | 39.63 (38.45,40.85) | 7.08 (6.62,7.57) |
| High-middle SDI | Italy | 60 to 64 | 53.29 (51.78,54.85) | 10.05 (9.45,10.68) |
| High-middle SDI | Italy | 65 to 69 | 63.55 (61.72,65.44) | 14.33 (13.51,15.2) |
| High-middle SDI | Italy | 70 to 74 | 81.26 (78.64,83.96) | 20.5 (19.22,21.86) |
| High-middle SDI | Italy | 75 to 79 | 90.39 (87.3,93.58) | 28.66 (26.86,30.58) |
| High-middle SDI | Italy | 80 to 84 | 95.9 (92.32,99.61) | 38.15 (35.69,40.77) |
| High-middle SDI | Italy | 85 to 89 | 110.45 (105.77,115.34) | 44.78 (41.72,48.07) |
| High-middle SDI | Jordan | 5 to 9 | 1.6 (1.06,2.4) | 1.55 (0.81,2.98) |
| High-middle SDI | Jordan | 10 to 14 | 1.15 (0.77,1.72) | 1.09 (0.58,2.08) |
| High-middle SDI | Jordan | 15 to 19 | 1 (0.68,1.49) | 1 (0.54,1.84) |
| High-middle SDI | Jordan | 20 to 24 | 1.38 (0.96,2) | 1.25 (0.71,2.18) |
| High-middle SDI | Jordan | 25 to 29 | 2.39 (1.71,3.35) | 1.53 (0.91,2.57) |
| High-middle SDI | Jordan | 30 to 34 | 3.31 (2.41,4.55) | 1.77 (1.08,2.91) |
| High-middle SDI | Jordan | 35 to 39 | 4.2 (3.12,5.66) | 2.4 (1.53,3.76) |
| High-middle SDI | Jordan | 40 to 44 | 5.38 (4.08,7.1) | 2.98 (1.98,4.48) |
| High-middle SDI | Jordan | 45 to 49 | 8.39 (6.51,10.82) | 4.22 (2.92,6.11) |
| High-middle SDI | Jordan | 50 to 54 | 10.86 (8.56,13.76) | 5.68 (4.07,7.93) |
| High-middle SDI | Jordan | 55 to 59 | 15.33 (12.33,19.05) | 8.14 (6.02,11) |
| High-middle SDI | Jordan | 60 to 64 | 24.54 (20.07,30.02) | 12.2 (9.23,16.13) |
| High-middle SDI | Jordan | 65 to 69 | 32.47 (26.46,39.85) | 17.11 (13.01,22.49) |
| High-middle SDI | Jordan | 70 to 74 | 40.33 (31.17,52.17) | 21.42 (15.34,29.89) |
| High-middle SDI | Jordan | 75 to 79 | 39.55 (28.99,53.95) | 22.85 (15.63,33.41) |
| High-middle SDI | Jordan | 80 to 84 | 47.84 (32.53,70.36) | 30.86 (19.89,47.88) |
| High-middle SDI | Jordan | 85 to 89 | 64.68 (38.43,108.86) | 41.3 (23.62,72.21) |
| High-middle SDI | Kazakhstan | 5 to 9 | 3.4 (2.43,4.75) | 3.11 (1.86,5.2) |
| High-middle SDI | Kazakhstan | 10 to 14 | 2.78 (2.02,3.81) | 2.4 (1.49,3.87) |
| High-middle SDI | Kazakhstan | 15 to 19 | 2.67 (1.99,3.59) | 2.63 (1.73,3.99) |
| High-middle SDI | Kazakhstan | 20 to 24 | 2.66 (2.02,3.51) | 2.57 (1.76,3.77) |
| High-middle SDI | Kazakhstan | 25 to 29 | 3.42 (2.67,4.37) | 2.5 (1.76,3.54) |
| High-middle SDI | Kazakhstan | 30 to 34 | 3.76 (3,4.71) | 2.38 (1.73,3.28) |
| High-middle SDI | Kazakhstan | 35 to 39 | 3.83 (3.1,4.74) | 2.4 (1.79,3.21) |
| High-middle SDI | Kazakhstan | 40 to 44 | 3.83 (3.18,4.61) | 2.4 (1.87,3.08) |
| High-middle SDI | Kazakhstan | 45 to 49 | 4.63 (3.87,5.53) | 2.78 (2.2,3.52) |
| High-middle SDI | Kazakhstan | 50 to 54 | 5.33 (4.47,6.34) | 3.2 (2.55,4.01) |
| High-middle SDI | Kazakhstan | 55 to 59 | 6.42 (5.42,7.61) | 3.87 (3.12,4.81) |
| High-middle SDI | Kazakhstan | 60 to 64 | 7.36 (6.21,8.72) | 4.32 (3.48,5.36) |
| High-middle SDI | Kazakhstan | 65 to 69 | 8.02 (6.7,9.59) | 4.95 (3.96,6.19) |
| High-middle SDI | Kazakhstan | 70 to 74 | 7.65 (6.12,9.58) | 4.86 (3.71,6.36) |
| High-middle SDI | Kazakhstan | 75 to 79 | 6.55 (4.99,8.6) | 4.46 (3.27,6.09) |
| High-middle SDI | Kazakhstan | 80 to 84 | 5.51 (3.83,7.93) | 4.11 (2.79,6.06) |
| High-middle SDI | Kazakhstan | 85 to 89 | 3.68 (1.95,6.95) | 2.62 (1.37,5.01) |
| High-middle SDI | Lebanon | 5 to 9 | 0.92 (0.57,1.49) | 0.72 (0.29,1.79) |
| High-middle SDI | Lebanon | 10 to 14 | 0.84 (0.53,1.32) | 0.65 (0.28,1.49) |
| High-middle SDI | Lebanon | 15 to 19 | 1.1 (0.72,1.68) | 0.97 (0.47,1.98) |
| High-middle SDI | Lebanon | 20 to 24 | 1.38 (0.93,2.02) | 1.15 (0.6,2.2) |
| High-middle SDI | Lebanon | 25 to 29 | 2.51 (1.8,3.5) | 1.33 (0.74,2.4) |
| High-middle SDI | Lebanon | 30 to 34 | 3.5 (2.58,4.76) | 1.76 (1.04,2.96) |
| High-middle SDI | Lebanon | 35 to 39 | 4.82 (3.64,6.37) | 2.47 (1.56,3.9) |
| High-middle SDI | Lebanon | 40 to 44 | 6.99 (5.46,8.95) | 3.36 (2.28,4.96) |
| High-middle SDI | Lebanon | 45 to 49 | 11.33 (9.15,14.03) | 5.12 (3.68,7.12) |
| High-middle SDI | Lebanon | 50 to 54 | 16.42 (13.58,19.85) | 7.62 (5.74,10.11) |
| High-middle SDI | Lebanon | 55 to 59 | 25.29 (21.36,29.95) | 11.81 (9.23,15.12) |
| High-middle SDI | Lebanon | 60 to 64 | 40.74 (34.88,47.59) | 17.73 (14.11,22.27) |
| High-middle SDI | Lebanon | 65 to 69 | 56.86 (48.55,66.6) | 26.87 (21.51,33.58) |
| High-middle SDI | Lebanon | 70 to 74 | 89.62 (74.74,107.47) | 42.59 (33.2,54.63) |
| High-middle SDI | Lebanon | 75 to 79 | 106.41 (87.41,129.54) | 56.64 (43.69,73.42) |
| High-middle SDI | Lebanon | 80 to 84 | 132.57 (106.52,164.99) | 78.72 (59.75,103.72) |
| High-middle SDI | Lebanon | 85 to 89 | 157.85 (121.6,204.9) | 90.53 (66.1,123.98) |
| High-middle SDI | Libya | 5 to 9 | 0.86 (0.57,1.31) | 0.5 (0.26,0.98) |
| High-middle SDI | Libya | 10 to 14 | 0.63 (0.41,0.95) | 0.35 (0.18,0.69) |
| High-middle SDI | Libya | 15 to 19 | 0.89 (0.61,1.3) | 0.57 (0.32,1.02) |
| High-middle SDI | Libya | 20 to 24 | 1.12 (0.78,1.6) | 0.73 (0.42,1.24) |
| High-middle SDI | Libya | 25 to 29 | 1.91 (1.38,2.65) | 0.94 (0.58,1.55) |
| High-middle SDI | Libya | 30 to 34 | 2.95 (2.19,3.98) | 1.33 (0.85,2.09) |
| High-middle SDI | Libya | 35 to 39 | 4.02 (3.04,5.31) | 1.99 (1.33,3) |
| High-middle SDI | Libya | 40 to 44 | 5.28 (4.06,6.86) | 2.74 (1.88,3.99) |
| High-middle SDI | Libya | 45 to 49 | 8.74 (6.88,11.1) | 4.4 (3.15,6.16) |
| High-middle SDI | Libya | 50 to 54 | 12.02 (9.61,15.03) | 6.5 (4.78,8.85) |
| High-middle SDI | Libya | 55 to 59 | 18.4 (14.93,22.69) | 10.44 (7.88,13.82) |
| High-middle SDI | Libya | 60 to 64 | 33.1 (27.3,40.13) | 18.33 (14.19,23.68) |
| High-middle SDI | Libya | 65 to 69 | 47.93 (39.35,58.39) | 28.75 (22.37,36.94) |
| High-middle SDI | Libya | 70 to 74 | 66.93 (52.63,85.12) | 42.57 (31.55,57.45) |
| High-middle SDI | Libya | 75 to 79 | 80.59 (61.63,105.39) | 57.57 (41.78,79.33) |
| High-middle SDI | Libya | 80 to 84 | 91.11 (66.65,124.55) | 71.69 (50.23,102.33) |
| High-middle SDI | Libya | 85 to 89 | 104.48 (70.4,155.05) | 87.56 (57.47,133.42) |
| High-middle SDI | Malaysia | 5 to 9 | 0.31 (0.22,0.43) | 0.28 (0.18,0.44) |
| High-middle SDI | Malaysia | 10 to 14 | 0.44 (0.33,0.58) | 0.37 (0.25,0.55) |
| High-middle SDI | Malaysia | 15 to 19 | 0.59 (0.45,0.76) | 0.56 (0.4,0.78) |
| High-middle SDI | Malaysia | 20 to 24 | 0.65 (0.51,0.83) | 0.64 (0.47,0.86) |
| High-middle SDI | Malaysia | 25 to 29 | 0.96 (0.77,1.19) | 0.71 (0.54,0.94) |
| High-middle SDI | Malaysia | 30 to 34 | 1.31 (1.07,1.59) | 0.85 (0.66,1.09) |
| High-middle SDI | Malaysia | 35 to 39 | 1.73 (1.45,2.07) | 1.16 (0.92,1.45) |
| High-middle SDI | Malaysia | 40 to 44 | 2.57 (2.2,3.01) | 1.86 (1.54,2.26) |
| High-middle SDI | Malaysia | 45 to 49 | 4.02 (3.49,4.63) | 2.83 (2.39,3.36) |
| High-middle SDI | Malaysia | 50 to 54 | 6.45 (5.69,7.3) | 4.72 (4.08,5.47) |
| High-middle SDI | Malaysia | 55 to 59 | 9.95 (8.88,11.15) | 7.33 (6.42,8.37) |
| High-middle SDI | Malaysia | 60 to 64 | 14.53 (13.03,16.19) | 10.59 (9.34,12.01) |
| High-middle SDI | Malaysia | 65 to 69 | 19.87 (17.76,22.24) | 15.23 (13.41,17.3) |
| High-middle SDI | Malaysia | 70 to 74 | 26.21 (22.81,30.12) | 20.88 (17.9,24.36) |
| High-middle SDI | Malaysia | 75 to 79 | 24.99 (21.2,29.46) | 21.57 (18.09,25.71) |
| High-middle SDI | Malaysia | 80 to 84 | 27.55 (22.56,33.63) | 25.83 (21.07,31.66) |
| High-middle SDI | Malaysia | 85 to 89 | 31.21 (23.85,40.83) | 30.3 (23.33,39.36) |
| High-middle SDI | Malta | 5 to 9 | 2.71 (0.92,8.01) | 0.2 (0,18.14) |
| High-middle SDI | Malta | 10 to 14 | 3.47 (1.28,9.43) | 0.2 (0,13.05) |
| High-middle SDI | Malta | 15 to 19 | 5.32 (2.3,12.33) | 0.18 (0,8.39) |
| High-middle SDI | Malta | 20 to 24 | 6.11 (2.78,13.41) | 0.19 (0.01,6.57) |
| High-middle SDI | Malta | 25 to 29 | 7.16 (3.46,14.81) | 0.21 (0.01,5.38) |
| High-middle SDI | Malta | 30 to 34 | 5.75 (2.78,11.86) | 0.23 (0.01,4.59) |
| High-middle SDI | Malta | 35 to 39 | 5.88 (2.92,11.85) | 0.25 (0.01,4.17) |
| High-middle SDI | Malta | 40 to 44 | 7.95 (4.3,14.69) | 0.68 (0.06,7.97) |
| High-middle SDI | Malta | 45 to 49 | 12.19 (7.25,20.51) | 3.26 (1.11,9.56) |
| High-middle SDI | Malta | 50 to 54 | 14.31 (8.7,23.55) | 3.51 (1.28,9.62) |
| High-middle SDI | Malta | 55 to 59 | 22.01 (14.27,33.94) | 6.21 (2.7,14.27) |
| High-middle SDI | Malta | 60 to 64 | 40.28 (27.63,58.71) | 9.93 (4.74,20.8) |
| High-middle SDI | Malta | 65 to 69 | 47.44 (31.81,70.73) | 14.67 (7.18,29.98) |
| High-middle SDI | Malta | 70 to 74 | 80.66 (51.4,126.6) | 21.78 (9.69,48.98) |
| High-middle SDI | Malta | 75 to 79 | 95.7 (58.55,156.41) | 29.14 (12.55,67.64) |
| High-middle SDI | Malta | 80 to 84 | 107.55 (62.81,184.17) | 38.41 (16.02,92.08) |
| High-middle SDI | Malta | 85 to 89 | 139.59 (75.5,258.11) | 42.02 (15.97,110.59) |
| High-middle SDI | Montenegro | 5 to 9 | 1.42 (0.3,6.62) | 0.3 (0,29.3) |
| High-middle SDI | Montenegro | 10 to 14 | 1.55 (0.38,6.38) | 0.29 (0,20.79) |
| High-middle SDI | Montenegro | 15 to 19 | 1.53 (0.41,5.69) | 0.31 (0.01,16.53) |
| High-middle SDI | Montenegro | 20 to 24 | 1.65 (0.48,5.62) | 0.32 (0.01,12.16) |
| High-middle SDI | Montenegro | 25 to 29 | 1.77 (0.56,5.53) | 0.33 (0.01,9.06) |
| High-middle SDI | Montenegro | 30 to 34 | 1.84 (0.63,5.35) | 0.31 (0.01,6.69) |
| High-middle SDI | Montenegro | 35 to 39 | 2.65 (1.05,6.72) | 0.3 (0.02,5.28) |
| High-middle SDI | Montenegro | 40 to 44 | 4.06 (1.94,8.49) | 1.9 (0.55,6.55) |
| High-middle SDI | Montenegro | 45 to 49 | 5.87 (3.04,11.33) | 2.68 (0.91,7.9) |
| High-middle SDI | Montenegro | 50 to 54 | 8.11 (4.57,14.4) | 2.68 (0.97,7.41) |
| High-middle SDI | Montenegro | 55 to 59 | 12.43 (7.43,20.81) | 4.21 (1.77,10.03) |
| High-middle SDI | Montenegro | 60 to 64 | 18.49 (11.41,29.98) | 6.22 (2.71,14.27) |
| High-middle SDI | Montenegro | 65 to 69 | 24.57 (15.01,40.24) | 8.48 (3.78,19) |
| High-middle SDI | Montenegro | 70 to 74 | 31.33 (17.26,56.88) | 12.95 (5.14,32.59) |
| High-middle SDI | Montenegro | 75 to 79 | 33.83 (17.34,66) | 15.66 (5.83,42.08) |
| High-middle SDI | Montenegro | 80 to 84 | 36.44 (16.52,80.38) | 21.22 (7.26,62.02) |
| High-middle SDI | Montenegro | 85 to 89 | 48.82 (16.54,144.11) | 22.62 (5.05,101.27) |
| High-middle SDI | North Macedonia | 5 to 9 | 0.75 (0.26,2.14) | 0.95 (0.16,5.81) |
| High-middle SDI | North Macedonia | 10 to 14 | 0.76 (0.29,2.01) | 0.91 (0.19,4.43) |
| High-middle SDI | North Macedonia | 15 to 19 | 0.73 (0.29,1.86) | 0.9 (0.22,3.79) |
| High-middle SDI | North Macedonia | 20 to 24 | 0.78 (0.32,1.86) | 0.92 (0.25,3.39) |
| High-middle SDI | North Macedonia | 25 to 29 | 1.27 (0.6,2.67) | 0.91 (0.28,2.98) |
| High-middle SDI | North Macedonia | 30 to 34 | 1.44 (0.73,2.81) | 0.86 (0.29,2.58) |
| High-middle SDI | North Macedonia | 35 to 39 | 1.81 (1,3.28) | 0.82 (0.29,2.27) |
| High-middle SDI | North Macedonia | 40 to 44 | 2.46 (1.47,4.11) | 1.32 (0.62,2.8) |
| High-middle SDI | North Macedonia | 45 to 49 | 3.9 (2.54,5.98) | 1.79 (0.92,3.49) |
| High-middle SDI | North Macedonia | 50 to 54 | 6.34 (4.43,9.09) | 2.77 (1.6,4.79) |
| High-middle SDI | North Macedonia | 55 to 59 | 9.45 (6.83,13.07) | 4.34 (2.69,6.99) |
| High-middle SDI | North Macedonia | 60 to 64 | 14.68 (10.88,19.81) | 6.32 (4.05,9.86) |
| High-middle SDI | North Macedonia | 65 to 69 | 19.5 (14.32,26.57) | 9.29 (5.99,14.39) |
| High-middle SDI | North Macedonia | 70 to 74 | 25.6 (17.69,37.05) | 12.66 (7.62,21.05) |
| High-middle SDI | North Macedonia | 75 to 79 | 31.94 (21.25,48) | 17.89 (10.47,30.57) |
| High-middle SDI | North Macedonia | 80 to 84 | 39.81 (24.57,64.51) | 24.5 (13.52,44.37) |
| High-middle SDI | North Macedonia | 85 to 89 | 68.13 (35.85,129.5) | 39.59 (18.69,83.85) |
| High-middle SDI | Oman | 5 to 9 | 0.56 (0.27,1.19) | 0.54 (0.15,1.92) |
| High-middle SDI | Oman | 10 to 14 | 0.63 (0.31,1.29) | 0.45 (0.13,1.56) |
| High-middle SDI | Oman | 15 to 19 | 0.77 (0.39,1.53) | 0.57 (0.18,1.8) |
| High-middle SDI | Oman | 20 to 24 | 0.92 (0.49,1.75) | 0.8 (0.29,2.19) |
| High-middle SDI | Oman | 25 to 29 | 1.45 (0.82,2.55) | 0.84 (0.33,2.11) |
| High-middle SDI | Oman | 30 to 34 | 1.93 (1.14,3.27) | 1.03 (0.45,2.35) |
| High-middle SDI | Oman | 35 to 39 | 2.61 (1.6,4.24) | 1.41 (0.67,2.97) |
| High-middle SDI | Oman | 40 to 44 | 3.53 (2.26,5.54) | 1.87 (0.95,3.68) |
| High-middle SDI | Oman | 45 to 49 | 6.22 (4.14,9.37) | 3.13 (1.73,5.68) |
| High-middle SDI | Oman | 50 to 54 | 9.49 (6.45,13.95) | 5.29 (3.1,9.02) |
| High-middle SDI | Oman | 55 to 59 | 15.81 (10.93,22.85) | 8.83 (5.34,14.6) |
| High-middle SDI | Oman | 60 to 64 | 26.22 (18.48,37.2) | 13.61 (8.46,21.89) |
| High-middle SDI | Oman | 65 to 69 | 39.03 (27.13,56.13) | 21.26 (13.1,34.5) |
| High-middle SDI | Oman | 70 to 74 | 48.87 (31.22,76.5) | 28.13 (15.86,49.89) |
| High-middle SDI | Oman | 75 to 79 | 50.76 (29.86,86.3) | 32.21 (16.94,61.27) |
| High-middle SDI | Oman | 80 to 84 | 58.66 (30.57,112.57) | 40.68 (19.4,85.28) |
| High-middle SDI | Oman | 85 to 89 | 68.61 (28.01,168.07) | 44.95 (17.43,115.92) |
| High-middle SDI | Portugal | 5 to 9 | 2.37 (1.78,3.14) | 1.02 (0.42,2.48) |
| High-middle SDI | Portugal | 10 to 14 | 2.53 (1.97,3.24) | 0.95 (0.44,2.04) |
| High-middle SDI | Portugal | 15 to 19 | 2.41 (1.93,3.01) | 1.15 (0.63,2.1) |
| High-middle SDI | Portugal | 20 to 24 | 3.86 (3.22,4.62) | 1.24 (0.75,2.06) |
| High-middle SDI | Portugal | 25 to 29 | 8.19 (7.1,9.44) | 1.38 (0.9,2.13) |
| High-middle SDI | Portugal | 30 to 34 | 11.62 (10.26,13.17) | 1.55 (1.07,2.23) |
| High-middle SDI | Portugal | 35 to 39 | 10.73 (9.52,12.09) | 1.98 (1.46,2.69) |
| High-middle SDI | Portugal | 40 to 44 | 13.26 (11.94,14.73) | 2.62 (2.04,3.35) |
| High-middle SDI | Portugal | 45 to 49 | 16.64 (15.12,18.3) | 3.49 (2.82,4.31) |
| High-middle SDI | Portugal | 50 to 54 | 18.91 (17.28,20.69) | 4.87 (4.06,5.84) |
| High-middle SDI | Portugal | 55 to 59 | 24.66 (22.68,26.81) | 7.13 (6.08,8.35) |
| High-middle SDI | Portugal | 60 to 64 | 43.98 (40.85,47.36) | 10.28 (8.89,11.89) |
| High-middle SDI | Portugal | 65 to 69 | 49.96 (46.21,54) | 14.94 (12.96,17.21) |
| High-middle SDI | Portugal | 70 to 74 | 76.61 (70.18,83.63) | 22.74 (19.45,26.6) |
| High-middle SDI | Portugal | 75 to 79 | 84.35 (76.72,92.74) | 31.67 (26.99,37.15) |
| High-middle SDI | Portugal | 80 to 84 | 107.19 (96.8,118.7) | 40.21 (34.05,47.49) |
| High-middle SDI | Portugal | 85 to 89 | 140.12 (124.77,157.36) | 48.72 (40.64,58.4) |
| High-middle SDI | Republic of Moldova | 5 to 9 | 1.73 (0.99,3.02) | 0.71 (0.25,2) |
| High-middle SDI | Republic of Moldova | 10 to 14 | 1.31 (0.76,2.26) | 0.5 (0.18,1.41) |
| High-middle SDI | Republic of Moldova | 15 to 19 | 0.85 (0.49,1.49) | 0.57 (0.23,1.44) |
| High-middle SDI | Republic of Moldova | 20 to 24 | 0.75 (0.43,1.3) | 0.55 (0.24,1.29) |
| High-middle SDI | Republic of Moldova | 25 to 29 | 1.11 (0.69,1.79) | 0.62 (0.29,1.33) |
| High-middle SDI | Republic of Moldova | 30 to 34 | 1.5 (0.98,2.28) | 0.64 (0.32,1.28) |
| High-middle SDI | Republic of Moldova | 35 to 39 | 1.83 (1.26,2.68) | 0.96 (0.54,1.7) |
| High-middle SDI | Republic of Moldova | 40 to 44 | 2.89 (2.12,3.95) | 1.47 (0.93,2.34) |
| High-middle SDI | Republic of Moldova | 45 to 49 | 4.89 (3.73,6.43) | 2.44 (1.65,3.62) |
| High-middle SDI | Republic of Moldova | 50 to 54 | 7.96 (6.28,10.09) | 3.85 (2.74,5.4) |
| High-middle SDI | Republic of Moldova | 55 to 59 | 12.17 (9.85,15.04) | 6.21 (4.64,8.32) |
| High-middle SDI | Republic of Moldova | 60 to 64 | 17.69 (14.46,21.64) | 8.86 (6.7,11.72) |
| High-middle SDI | Republic of Moldova | 65 to 69 | 23.42 (19.04,28.81) | 12.76 (9.64,16.89) |
| High-middle SDI | Republic of Moldova | 70 to 74 | 28.46 (21.87,37.04) | 15.66 (11.1,22.08) |
| High-middle SDI | Republic of Moldova | 75 to 79 | 28.62 (21.12,38.77) | 16.8 (11.49,24.56) |
| High-middle SDI | Republic of Moldova | 80 to 84 | 25.89 (17.59,38.08) | 17.32 (11.03,27.19) |
| High-middle SDI | Republic of Moldova | 85 to 89 | 18.4 (9.78,34.62) | 12.49 (6.26,24.92) |
| High-middle SDI | Romania | 5 to 9 | 1.1 (0.8,1.52) | 0.81 (0.5,1.32) |
| High-middle SDI | Romania | 10 to 14 | 0.9 (0.67,1.22) | 0.65 (0.42,1.02) |
| High-middle SDI | Romania | 15 to 19 | 0.78 (0.59,1.04) | 0.65 (0.43,0.97) |
| High-middle SDI | Romania | 20 to 24 | 0.86 (0.66,1.12) | 0.73 (0.51,1.05) |
| High-middle SDI | Romania | 25 to 29 | 1.26 (1,1.59) | 0.89 (0.65,1.21) |
| High-middle SDI | Romania | 30 to 34 | 1.34 (1.08,1.67) | 0.9 (0.67,1.2) |
| High-middle SDI | Romania | 35 to 39 | 1.97 (1.64,2.36) | 1.26 (0.99,1.6) |
| High-middle SDI | Romania | 40 to 44 | 2.05 (1.74,2.41) | 1.71 (1.4,2.08) |
| High-middle SDI | Romania | 45 to 49 | 3.45 (3.01,3.95) | 2.53 (2.14,2.99) |
| High-middle SDI | Romania | 50 to 54 | 5.89 (5.26,6.6) | 3.83 (3.32,4.42) |
| High-middle SDI | Romania | 55 to 59 | 10.19 (9.24,11.23) | 5.5 (4.83,6.26) |
| High-middle SDI | Romania | 60 to 64 | 15.59 (14.26,17.04) | 7.95 (7.05,8.97) |
| High-middle SDI | Romania | 65 to 69 | 23.09 (21.13,25.22) | 11.47 (10.18,12.93) |
| High-middle SDI | Romania | 70 to 74 | 34.55 (31.09,38.39) | 15.58 (13.55,17.92) |
| High-middle SDI | Romania | 75 to 79 | 44.62 (39.82,50) | 19.95 (17.2,23.14) |
| High-middle SDI | Romania | 80 to 84 | 43.04 (37.58,49.3) | 22.65 (19.16,26.78) |
| High-middle SDI | Romania | 85 to 89 | 56.67 (47.68,67.37) | 28.07 (22.78,34.59) |
| High-middle SDI | Russian Federation | 5 to 9 | 1.28 (1.09,1.49) | 0.77 (0.63,0.94) |
| High-middle SDI | Russian Federation | 10 to 14 | 1 (0.87,1.16) | 0.6 (0.5,0.72) |
| High-middle SDI | Russian Federation | 15 to 19 | 1 (0.88,1.14) | 0.67 (0.57,0.78) |
| High-middle SDI | Russian Federation | 20 to 24 | 0.98 (0.87,1.11) | 0.76 (0.67,0.87) |
| High-middle SDI | Russian Federation | 25 to 29 | 1.73 (1.57,1.92) | 0.9 (0.8,1.02) |
| High-middle SDI | Russian Federation | 30 to 34 | 2.45 (2.25,2.67) | 1.02 (0.91,1.13) |
| High-middle SDI | Russian Federation | 35 to 39 | 3.17 (2.93,3.42) | 1.23 (1.12,1.35) |
| High-middle SDI | Russian Federation | 40 to 44 | 4.54 (4.26,4.85) | 1.64 (1.52,1.78) |
| High-middle SDI | Russian Federation | 45 to 49 | 7.01 (6.62,7.42) | 2.37 (2.22,2.54) |
| High-middle SDI | Russian Federation | 50 to 54 | 10.58 (10.06,11.13) | 3.57 (3.37,3.78) |
| High-middle SDI | Russian Federation | 55 to 59 | 15.64 (14.95,16.37) | 5.25 (4.99,5.53) |
| High-middle SDI | Russian Federation | 60 to 64 | 23.13 (22.16,24.14) | 7.4 (7.05,7.77) |
| High-middle SDI | Russian Federation | 65 to 69 | 30.61 (29.33,31.94) | 10.34 (9.86,10.85) |
| High-middle SDI | Russian Federation | 70 to 74 | 39.11 (37.08,41.26) | 13.41 (12.67,14.2) |
| High-middle SDI | Russian Federation | 75 to 79 | 44.21 (41.64,46.94) | 16.84 (15.85,17.9) |
| High-middle SDI | Russian Federation | 80 to 84 | 45.82 (42.64,49.24) | 19.87 (18.55,21.27) |
| High-middle SDI | Russian Federation | 85 to 89 | 47.58 (42.94,52.73) | 18.95 (17.26,20.79) |
| High-middle SDI | Saint Kitts and Nevis | 5 to 9 | 5.47 (0.03,1086.56) | - |
| High-middle SDI | Saint Kitts and Nevis | 10 to 14 | 5.24 (0.04,761.19) | - |
| High-middle SDI | Saint Kitts and Nevis | 15 to 19 | 5.14 (0.05,583.97) | - |
| High-middle SDI | Saint Kitts and Nevis | 20 to 24 | 5.4 (0.06,481.38) | - |
| High-middle SDI | Saint Kitts and Nevis | 25 to 29 | 5.51 (0.08,385.62) | - |
| High-middle SDI | Saint Kitts and Nevis | 30 to 34 | 5.08 (0.1,266.61) | - |
| High-middle SDI | Saint Kitts and Nevis | 35 to 39 | 4.73 (0.12,185.46) | - |
| High-middle SDI | Saint Kitts and Nevis | 40 to 44 | 4.55 (0.17,122.76) | - |
| High-middle SDI | Saint Kitts and Nevis | 45 to 49 | 5.64 (0.24,130.25) | - |
| High-middle SDI | Saint Kitts and Nevis | 50 to 54 | 7.34 (0.35,153.36) | - |
| High-middle SDI | Saint Kitts and Nevis | 55 to 59 | 30.76 (2.7,349.84) | - |
| High-middle SDI | Saint Kitts and Nevis | 60 to 64 | 34.65 (6.14,195.66) | - |
| High-middle SDI | Saint Kitts and Nevis | 65 to 69 | 37.39 (5.93,235.67) | - |
| High-middle SDI | Saint Kitts and Nevis | 70 to 74 | 34.03 (1.1,1050.89) | - |
| High-middle SDI | Saint Kitts and Nevis | 75 to 79 | 47.2 (1.23,1818.5) | - |
| High-middle SDI | Saint Kitts and Nevis | 80 to 84 | 77.38 (1.55,3872.18) | - |
| High-middle SDI | Saint Kitts and Nevis | 85 to 89 | 167.8 (2.37,11856.01) | - |
| High-middle SDI | Serbia | 5 to 9 | 1.74 (1.09,2.78) | 0.92 (0.39,2.18) |
| High-middle SDI | Serbia | 10 to 14 | 1.31 (0.84,2.04) | 0.58 (0.25,1.35) |
| High-middle SDI | Serbia | 15 to 19 | 0.91 (0.59,1.41) | 0.58 (0.28,1.22) |
| High-middle SDI | Serbia | 20 to 24 | 0.94 (0.63,1.4) | 0.71 (0.38,1.32) |
| High-middle SDI | Serbia | 25 to 29 | 1.55 (1.12,2.15) | 0.86 (0.5,1.48) |
| High-middle SDI | Serbia | 30 to 34 | 1.95 (1.46,2.61) | 0.94 (0.58,1.5) |
| High-middle SDI | Serbia | 35 to 39 | 2.6 (2.04,3.32) | 1.12 (0.75,1.67) |
| High-middle SDI | Serbia | 40 to 44 | 3.61 (2.96,4.42) | 1.66 (1.22,2.26) |
| High-middle SDI | Serbia | 45 to 49 | 5.77 (4.86,6.84) | 2.68 (2.07,3.45) |
| High-middle SDI | Serbia | 50 to 54 | 9.08 (7.85,10.51) | 4.31 (3.49,5.32) |
| High-middle SDI | Serbia | 55 to 59 | 12.97 (11.41,14.75) | 6.69 (5.61,7.99) |
| High-middle SDI | Serbia | 60 to 64 | 19.56 (17.38,22.03) | 9.74 (8.27,11.48) |
| High-middle SDI | Serbia | 65 to 69 | 24.93 (22.08,28.15) | 14.32 (12.19,16.83) |
| High-middle SDI | Serbia | 70 to 74 | 32.3 (27.94,37.35) | 18.73 (15.52,22.61) |
| High-middle SDI | Serbia | 75 to 79 | 37.32 (31.82,43.78) | 25.27 (20.74,30.77) |
| High-middle SDI | Serbia | 80 to 84 | 44.68 (37.08,53.84) | 33.63 (27.05,41.82) |
| High-middle SDI | Serbia | 85 to 89 | 43.55 (33.4,56.79) | 32.35 (24.21,43.22) |
| High-middle SDI | Seychelles | 5 to 9 | 0.75 (0.01,103.53) | 1.15 (0.01,195.5) |
| High-middle SDI | Seychelles | 10 to 14 | 0.79 (0.01,78.77) | 1.17 (0.01,144.56) |
| High-middle SDI | Seychelles | 15 to 19 | 0.84 (0.01,63.63) | 1.17 (0.01,111.76) |
| High-middle SDI | Seychelles | 20 to 24 | 0.8 (0.01,44.97) | 1.18 (0.02,88.03) |
| High-middle SDI | Seychelles | 25 to 29 | 0.76 (0.02,32.15) | 1.06 (0.02,58.97) |
| High-middle SDI | Seychelles | 30 to 34 | 0.77 (0.02,24.99) | 1.05 (0.02,44.61) |
| High-middle SDI | Seychelles | 35 to 39 | 0.85 (0.03,22.08) | 1.11 (0.03,36.08) |
| High-middle SDI | Seychelles | 40 to 44 | 1.05 (0.05,20.86) | 1.29 (0.05,30.3) |
| High-middle SDI | Seychelles | 45 to 49 | 6.75 (0.76,60.21) | 1.72 (0.09,34.11) |
| High-middle SDI | Seychelles | 50 to 54 | 9.63 (1.43,64.65) | 5.97 (0.41,87.01) |
| High-middle SDI | Seychelles | 55 to 59 | 15.32 (3.44,68.22) | 12.61 (1.31,121.23) |
| High-middle SDI | Seychelles | 60 to 64 | 30 (7.58,118.68) | 19.27 (3.63,102.28) |
| High-middle SDI | Seychelles | 65 to 69 | 50.81 (11.51,224.26) | 38.99 (6.98,217.95) |
| High-middle SDI | Seychelles | 70 to 74 | 87.14 (13.61,558.09) | 130.67 (14.72,1160.28) |
| High-middle SDI | Seychelles | 75 to 79 | 75.17 (6.31,895.13) | 29.8 (0.88,1008.47) |
| High-middle SDI | Seychelles | 80 to 84 | 54.48 (1.61,1845.96) | 47.77 (1.14,2008.34) |
| High-middle SDI | Seychelles | 85 to 89 | 116.98 (2.48,5524.42) | 99.74 (1.72,5783.32) |
| High-middle SDI | Spain | 5 to 9 | 4.78 (3.88,5.88) | 1 (0.6,1.66) |
| High-middle SDI | Spain | 10 to 14 | 4.1 (3.4,4.94) | 0.98 (0.64,1.5) |
| High-middle SDI | Spain | 15 to 19 | 3.73 (3.15,4.41) | 1.28 (0.91,1.78) |
| High-middle SDI | Spain | 20 to 24 | 4.44 (3.85,5.11) | 1.34 (1.01,1.76) |
| High-middle SDI | Spain | 25 to 29 | 6.33 (5.66,7.09) | 1.47 (1.17,1.84) |
| High-middle SDI | Spain | 30 to 34 | 8.69 (7.92,9.54) | 1.64 (1.35,1.99) |
| High-middle SDI | Spain | 35 to 39 | 8.41 (7.72,9.16) | 2.01 (1.71,2.36) |
| High-middle SDI | Spain | 40 to 44 | 12.42 (11.58,13.33) | 2.61 (2.29,2.98) |
| High-middle SDI | Spain | 45 to 49 | 16.76 (15.76,17.82) | 3.29 (2.94,3.69) |
| High-middle SDI | Spain | 50 to 54 | 21.25 (20.11,22.47) | 4.41 (4,4.87) |
| High-middle SDI | Spain | 55 to 59 | 24.97 (23.68,26.34) | 6.2 (5.68,6.77) |
| High-middle SDI | Spain | 60 to 64 | 36.35 (34.6,38.19) | 8.42 (7.76,9.14) |
| High-middle SDI | Spain | 65 to 69 | 43.52 (41.38,45.76) | 11.83 (10.93,12.8) |
| High-middle SDI | Spain | 70 to 74 | 63.27 (59.84,66.89) | 17.09 (15.68,18.64) |
| High-middle SDI | Spain | 75 to 79 | 66.03 (62.21,70.08) | 23.69 (21.7,25.86) |
| High-middle SDI | Spain | 80 to 84 | 69.75 (65.39,74.39) | 31.85 (29.12,34.85) |
| High-middle SDI | Spain | 85 to 89 | 81.85 (76.16,87.95) | 38.76 (35.24,42.63) |
| High-middle SDI | Trinidad and Tobago | 5 to 9 | 1 (0.36,2.82) | 0.71 (0.16,3.18) |
| High-middle SDI | Trinidad and Tobago | 10 to 14 | 0.7 (0.26,1.92) | 0.71 (0.19,2.71) |
| High-middle SDI | Trinidad and Tobago | 15 to 19 | 1.25 (0.53,2.93) | 0.83 (0.24,2.8) |
| High-middle SDI | Trinidad and Tobago | 20 to 24 | 1.78 (0.83,3.83) | 1.16 (0.4,3.34) |
| High-middle SDI | Trinidad and Tobago | 25 to 29 | 2.89 (1.49,5.59) | 1.63 (0.64,4.14) |
| High-middle SDI | Trinidad and Tobago | 30 to 34 | 4.11 (2.3,7.35) | 1.87 (0.81,4.32) |
| High-middle SDI | Trinidad and Tobago | 35 to 39 | 4.02 (2.3,7.04) | 2.13 (0.98,4.65) |
| High-middle SDI | Trinidad and Tobago | 40 to 44 | 4.72 (2.86,7.79) | 2.93 (1.5,5.72) |
| High-middle SDI | Trinidad and Tobago | 45 to 49 | 7.19 (4.61,11.2) | 4.36 (2.44,7.79) |
| High-middle SDI | Trinidad and Tobago | 50 to 54 | 9.93 (6.64,14.87) | 5.38 (3.15,9.19) |
| High-middle SDI | Trinidad and Tobago | 55 to 59 | 13.29 (9.09,19.43) | 7.77 (4.74,12.74) |
| High-middle SDI | Trinidad and Tobago | 60 to 64 | 18.24 (12.67,26.26) | 10.07 (6.27,16.15) |
| High-middle SDI | Trinidad and Tobago | 65 to 69 | 19.04 (12.92,28.06) | 12.46 (7.69,20.19) |
| High-middle SDI | Trinidad and Tobago | 70 to 74 | 24.42 (15.37,38.81) | 15.23 (8.58,27.03) |
| High-middle SDI | Trinidad and Tobago | 75 to 79 | 25.37 (14.96,43.02) | 18.09 (9.65,33.91) |
| High-middle SDI | Trinidad and Tobago | 80 to 84 | 21.47 (11.16,41.3) | 18.16 (8.82,37.37) |
| High-middle SDI | Trinidad and Tobago | 85 to 89 | 21.5 (9.26,49.89) | 15.74 (6.17,40.14) |
| High-middle SDI | Ukraine | 5 to 9 | 0.77 (0.61,0.97) | 0.32 (0.22,0.46) |
| High-middle SDI | Ukraine | 10 to 14 | 0.77 (0.62,0.94) | 0.34 (0.24,0.47) |
| High-middle SDI | Ukraine | 15 to 19 | 0.88 (0.74,1.06) | 0.45 (0.35,0.59) |
| High-middle SDI | Ukraine | 20 to 24 | 1.11 (0.95,1.3) | 0.6 (0.48,0.76) |
[truncated: 588,894 more chars]
